# Supplementary material for: The Relationship between Serum Adipokines, miR-222-3p, miR-103a-3p and Glucose Regulation in Pregnancy and Two to Three Years Post-Delivery in Women with Gestational Diabetes Mellitus Adhering to Mediterranean Diet Recommendations
Source: Nutrients. 2022 Nov 8;14(22):4712. doi: 10.3390/nu14224712 (PMC9698999; doi:10.3390/nu14224712)
Supplement: Supplementary file 1 [file nutrients-14-04712-s001.zip › nutrients-1977670-supplementary.pdf]

| GDM | NAGT_AGT_2a | GROUPS |
|---|---|---|
| NGT | NGT | CONTROL |
| NGT | NGT | CONTROL |
| NGT | NGT | CONTROL |
| DMG | AGT | CONTROL |
| NGT | AGT | CONTROL |
| NGT | NGT | CONTROL |
| NGT | NGT | CONTROL |
| NGT | NGT | CONTROL |
| DMG | NGT | CONTROL |
| NGT | AGT | CONTROL |
| DMG | NGT | CONTROL |
| NGT | AGT | CONTROL |
| NGT | NGT | CONTROL |
| NGT | NGT | CONTROL |
| NGT | NGT | CONTROL |
| NGT | NGT | CONTROL |
| NGT | NGT | CONTROL |
| DMG | NGT | CONTROL |
| NGT | NGT | CONTROL |
| DMG | AGT | CONTROL |
| NGT | NGT | CONTROL |
| NGT | NGT | CONTROL |
| NGT | AGT | CONTROL |
| DMG | NGT | CONTROL |
| DMG | NGT | CONTROL |
| NGT | NGT | CONTROL |
| DMG | NGT | CONTROL |
| NGT | AGT | CONTROL |
| DMG | AGT | CONTROL |
| NGT | NGT | CONTROL |
| NGT | AGT | CONTROL |
| DMG | AGT | CONTROL |
| DMG | NGT | CONTROL |
| NGT | NGT | CONTROL |
| NGT | NGT | CONTROL |
| NGT | NGT | CONTROL |
| NGT | NGT | CONTROL |
| DMG | NGT | CONTROL |
| NGT | NGT | CONTROL |
| NGT | NGT | CONTROL |
| DMG | NGT | CONTROL |
| DMG | AGT | CONTROL |
| NGT | NGT | CONTROL |
| NGT | NGT | CONTROL |
| NGT | NGT | CONTROL |
| NGT | NGT | CONTROL |
| NGT | NGT | CONTROL |

| | | |
|---|---|---|
| NGT | NGT | CONTROL |
| NGT | NGT | CONTROL |
| NGT | NGT | CONTROL |
| NGT | NGT | CONTROL |
| NGT | NGT | CONTROL |
| DMG | NGT | CONTROL |
| NGT | NGT | CONTROL |
| NGT | NGT | CONTROL |
| NGT | NGT | CONTROL |
| NGT | NGT | CONTROL |
| NGT | NGT | CONTROL |
| NGT | NGT | CONTROL |
| NGT | NGT | CONTROL |
| NGT | NGT | CONTROL |
| NGT | NGT | CONTROL |
| NGT | NGT | CONTROL |
| NGT | AGT | CONTROL |
| NGT | NGT | CONTROL |
| NGT | NGT | CONTROL |
| NGT | NGT | CONTROL |
| NGT | NGT | CONTROL |
| NGT | NGT | CONTROL |
| NGT | NGT | CONTROL |
| NGT | NGT | CONTROL |
| NGT | NGT | CONTROL |
| NGT | AGT | CONTROL |
| DMG | NGT | CONTROL |
| DMG | AGT | CONTROL |
| NGT | NGT | CONTROL |
| NGT | NGT | CONTROL |
| NGT | NGT | CONTROL |
| NGT | NGT | CONTROL |
| DMG | AGT | CONTROL |
| NGT | NGT | CONTROL |
| NGT | NGT | CONTROL |
| NGT | NGT | CONTROL |
| NGT | NGT | CONTROL |
| DMG | AGT | CONTROL |
| NGT | NGT | CONTROL |
| DMG | AGT | CONTROL |
| NGT | NGT | CONTROL |
| DMG | AGT | CONTROL |
| NGT | NGT | CONTROL |
| NGT | AGT | CONTROL |
| NGT | NGT | CONTROL |
| NGT | NGT | CONTROL |
| NGT | NGT | CONTROL |
| DMG | NGT | CONTROL |
| NGT | NGT | CONTROL |
| NGT | NGT | CONTROL |

| | | |
|---|---|---|
| NGT | NGT | CONTROL |
| NGT | NGT | CONTROL |
| NGT | NGT | CONTROL |
| NGT | NGT | CONTROL |
| NGT | NGT | CONTROL |
| NGT | NGT | CONTROL |
| NGT | NGT | CONTROL |
| NGT | NGT | CONTROL |
| NGT | AGT | CONTROL |
| NGT | NGT | CONTROL |
| NGT | NGT | CONTROL |
| NGT | AGT | CONTROL |
| NGT | NGT | INTERVENCION |
| NGT | NGT | CONTROL |
| DMG | NGT | CONTROL |
| NGT | NGT | CONTROL |
| DMG | NGT | CONTROL |
| NGT | NGT | INTERVENCION |
| DMG | NGT | CONTROL |
| NGT | NGT | CONTROL |
| NGT | NGT | INTERVENCION |
| NGT | NGT | CONTROL |
| NGT | NGT | CONTROL |
| NGT | NGT | INTERVENCION |
| DMG | NGT | INTERVENCION |
| NGT | NGT | CONTROL |
| NGT | NGT | CONTROL |
| NGT | NGT | INTERVENCION |
| DMG | NGT | INTERVENCION |
| NGT | NGT | INTERVENCION |
| NGT | NGT | INTERVENCION |
| DMG | AGT | CONTROL |
| NGT | NGT | INTERVENCION |
| NGT | NGT | INTERVENCION |
| DMG | AGT | CONTROL |
| NGT | NGT | INTERVENCION |
| DMG | AGT | CONTROL |
| DMG | AGT | CONTROL |
| NGT | NGT | CONTROL |
| NGT | NGT | INTERVENCION |
| NGT | NGT | INTERVENCION |
| DMG | AGT | INTERVENCION |
| DMG | NGT | CONTROL |
| NGT | NGT | INTERVENCION |
| DMG | NGT | INTERVENCION |
| DMG | NGT | INTERVENCION |
| NGT | NGT | INTERVENCION |
| NGT | NGT | CONTROL |
| NGT | NGT | INTERVENCION |
| DMG | NGT | CONTROL |

| | | |
|---|---|---|
| DMG | NGT | INTERVENCION |
| DMG | NGT | INTERVENCION |
| NGT | NGT | INTERVENCION |
| NGT | NGT | INTERVENCION |
| DMG | NGT | INTERVENCION |
| DMG | NGT | CONTROL |
| NGT | NGT | INTERVENCION |
| NGT | NGT | INTERVENCION |
| NGT | NGT | INTERVENCION |
| NGT | AGT | INTERVENCION |
| NGT | NGT | INTERVENCION |
| NGT | NGT | INTERVENCION |
| NGT | NGT | INTERVENCION |
| NGT | AGT | INTERVENCION |
| NGT | NGT | INTERVENCION |
| DMG | NGT | INTERVENCION |
| NGT | NGT | INTERVENCION |
| NGT | NGT | INTERVENCION |
| DMG | NGT | INTERVENCION |
| NGT | NGT | INTERVENCION |
| NGT | NGT | INTERVENCION |
| NGT | NGT | INTERVENCION |
| NGT | NGT | INTERVENCION |
| NGT | NGT | INTERVENCION |
| DMG | AGT | INTERVENCION |
| NGT | NGT | INTERVENCION |
| NGT | NGT | INTERVENCION |
| NGT | NGT | INTERVENCION |
| NGT | NGT | INTERVENCION |
| NGT | NGT | INTERVENCION |
| NGT | NGT | INTERVENCION |
| NGT | NGT | INTERVENCION |
| NGT | NGT | INTERVENCION |
| NGT | AGT | INTERVENCION |
| DMG | NGT | INTERVENCION |
| DMG | NGT | INTERVENCION |
| NGT | NGT | INTERVENCION |
| NGT | NGT | INTERVENCION |
| NGT | NGT | INTERVENCION |
| DMG | NGT | INTERVENCION |
| NGT | NGT | INTERVENCION |
| DMG | NGT | INTERVENCION |
| NGT | NGT | INTERVENCION |
| DMG | NGT | INTERVENCION |
| NGT | NGT | CONTROL |
| NGT | NGT | INTERVENCION |
| NGT | NGT | INTERVENCION |
| NGT | NGT | INTERVENCION |
| NGT | NGT | INTERVENCION |
| NGT | NGT | INTERVENCION |

| | | |
|---|---|---|
| DMG | NGT | CONTROL |
| NGT | NGT | INTERVENCION |
| NGT | AGT | INTERVENCION |
| NGT | NGT | INTERVENCION |
| NGT | NGT | INTERVENCION |
| NGT | AGT | INTERVENCION |
| NGT | NGT | INTERVENCION |
| DMG | AGT | CONTROL |
| NGT | NGT | INTERVENCION |
| NGT | NGT | INTERVENCION |
| NGT | NGT | INTERVENCION |
| NGT | NGT | INTERVENCION |
| NGT | NGT | INTERVENCION |
| NGT | NGT | INTERVENCION |
| NGT | NGT | INTERVENCION |
| NGT | AGT | INTERVENCION |
| NGT | NGT | INTERVENCION |
| DMG | NGT | INTERVENCION |
| NGT | NGT | INTERVENCION |
| NGT | NGT | INTERVENCION |
| NGT | NGT | INTERVENCION |
| DMG | NGT | CONTROL |
| NGT | NGT | INTERVENCION |
| NGT | NGT | INTERVENCION |
| NGT | NGT | INTERVENCION |
| NGT | NGT | INTERVENCION |
| DMG | NGT | INTERVENCION |
| NGT | NGT | INTERVENCION |
| NGT | NGT | INTERVENCION |
| NGT | NGT | INTERVENCION |
| DMG | NGT | CONTROL |
| DMG | AGT | INTERVENCION |
| DMG | NGT | INTERVENCION |
| NGT | AGT | INTERVENCION |
| NGT | NGT | INTERVENCION |
| NGT | NGT | INTERVENCION |
| NGT | NGT | INTERVENCION |
| NGT | NGT | INTERVENCION |
| NGT | NGT | INTERVENCION |
| NGT | NGT | INTERVENCION |
| NGT | AGT | INTERVENCION |
| DMG | AGT | INTERVENCION |
| NGT | NGT | INTERVENCION |
| DMG | NGT | INTERVENCION |
| NGT | NGT | INTERVENCION |
| NGT | AGT | INTERVENCION |
| NGT | NGT | INTERVENCION |
| NGT | NGT | INTERVENCION |
| NGT | NGT | INTERVENCION |
| DMG | NGT | INTERVENCION |

| | | |
|---|---|---|
| NGT | NGT | INTERVENCION |
| NGT | NGT | INTERVENCION |
| DMG | AGT | INTERVENCION |
| DMG | AGT | INTERVENCION |
| DMG | NGT | INTERVENCION |
| NGT | NGT | CONTROL |
| DMG | NGT | INTERVENCION |
| NGT | NGT | INTERVENCION |
| NGT | NGT | INTERVENCION |
| NGT | NGT | INTERVENCION |
| DMG | AGT | CONTROL |
| NGT | NGT | INTERVENCION |
| DMG | NGT | INTERVENCION |
| DMG | AGT | INTERVENCION |
| NGT | NGT | INTERVENCION |
| NGT | NGT | INTERVENCION |
| NGT | NGT | INTERVENCION |
| NGT | NGT | INTERVENCION |
| NGT | AGT | INTERVENCION |
| NGT | NGT | INTERVENCION |
| NGT | NGT | INTERVENCION |
| NGT | NGT | INTERVENCION |
| DMG | NGT | INTERVENCION |
| NGT | NGT | INTERVENCION |
| DMG | NGT | INTERVENCION |
| NGT | NGT | INTERVENCION |
| NGT | NGT | INTERVENCION |
| NGT | NGT | INTERVENCION |
| NGT | NGT | INTERVENCION |
| NGT | NGT | INTERVENCION |
| NGT | NGT | INTERVENCION |
| DMG | AGT | INTERVENCION |
| DMG | AGT | INTERVENCION |
| NGT | NGT | INTERVENCION |
| NGT | NGT | INTERVENCION |
| NGT | NGT | CONTROL |
| NGT | NGT | INTERVENCION |
| NGT | NGT | INTERVENCION |
| NGT | NGT | INTERVENCION |
| NGT | NGT | INTERVENCION |
| NGT | NGT | CONTROL |
| NGT | NGT | INTERVENCION |
| NGT | NGT | CONTROL |
| NGT | NGT | INTERVENCION |
| NGT | NGT | INTERVENCION |
| NGT | NGT | INTERVENCION |
| NGT | AGT | INTERVENCION |
| NGT | NGT | INTERVENCION |
| NGT | NGT | INTERVENCION |
| DMG | AGT | CONTROL |

| | | |
|---|---|---|
| NGT | NGT | INTERVENCION |
| DMG | NGT | INTERVENCION |
| DMG | NGT | INTERVENCION |
| NGT | NGT | INTERVENCION |
| DMG | NGT | INTERVENCION |
| NGT | NGT | INTERVENCION |
| NGT | NGT | INTERVENCION |
| NGT | NGT | INTERVENCION |
| NGT | NGT | INTERVENCION |
| NGT | AGT | INTERVENCION |
| DMG | AGT | CONTROL |
| DMG | AGT | CONTROL |
| NGT | NGT | INTERVENCION |
| NGT | AGT | INTERVENCION |
| DMG | NGT | INTERVENCION |
| NGT | AGT | INTERVENCION |

| GROUPS INTERVENTION/GDM | Body Weight (Kg) 24 gw | Body Weight (Kg) 2 years PD |
|---|---|---|
| 1. Control (No intervencion No Diabetes) | 62,0 | 52,6 |
| 1. Control (No intervencion No Diabetes) | 62,5 | 61,1 |
| 1. Control (No intervencion No Diabetes) | NAN | 66,0 |
| 2. Controles que SI desarrollan DMG | 84,0 | 89,2 |
| 1. Control (No intervencion No Diabetes) | 59,6 | 61,5 |
| 1. Control (No intervencion No Diabetes) | 51,9 | 47,8 |
| 1. Control (No intervencion No Diabetes) | 52,7 | NAN |
| 1. Control (No intervencion No Diabetes) | 81,0 | 83,8 |
| 2. Controles que SI desarrollan DMG | 67,7 | 64,7 |
| 1. Control (No intervencion No Diabetes) | 86,0 | 91,6 |
| 2. Controles que SI desarrollan DMG | 49,4 | 40,0 |
| 1. Control (No intervencion No Diabetes) | 91,0 | 87,4 |
| 1. Control (No intervencion No Diabetes) | 56,0 | 52,4 |
| 1. Control (No intervencion No Diabetes) | 105,0 | 109,2 |
| 1. Control (No intervencion No Diabetes) | 63,0 | 54,6 |
| 1. Control (No intervencion No Diabetes) | 56,5 | 51,0 |
| 1. Control (No intervencion No Diabetes) | 60,0 | 53,5 |
| 2. Controles que SI desarrollan DMG | 62,0 | 56,5 |
| 1. Control (No intervencion No Diabetes) | 67,7 | 68,7 |
| 2. Controles que SI desarrollan DMG | 76,0 | 69,7 |
| 1. Control (No intervencion No Diabetes) | 57,3 | 54,3 |
| 1. Control (No intervencion No Diabetes) | 74,0 | 73,5 |
| 1. Control (No intervencion No Diabetes) | NAN | 81,4 |
| 2. Controles que SI desarrollan DMG | 65,8 | 58,4 |
| 2. Controles que SI desarrollan DMG | 86,9 | 82,0 |
| 1. Control (No intervencion No Diabetes) | 63,0 | NAN |
| 2. Controles que SI desarrollan DMG | 70,3 | 67,8 |
| 1. Control (No intervencion No Diabetes) | 83,7 | 87,3 |
| 2. Controles que SI desarrollan DMG | 57,0 | 48,2 |
| 1. Control (No intervencion No Diabetes) | 73,3 | 62,6 |
| 1. Control (No intervencion No Diabetes) | 71,8 | 69,7 |
| 2. Controles que SI desarrollan DMG | 62,0 | 58,1 |
| 2. Controles que SI desarrollan DMG | 67,1 | 66,2 |
| 1. Control (No intervencion No Diabetes) | 84,0 | 80,0 |
| 1. Control (No intervencion No Diabetes) | 62,3 | 60,6 |
| 1. Control (No intervencion No Diabetes) | 66,5 | 59,3 |
| 1. Control (No intervencion No Diabetes) | 57,0 | 51,5 |
| 2. Controles que SI desarrollan DMG | 88,6 | 92,7 |
| 1. Control (No intervencion No Diabetes) | 71,9 | NAN |
| 1. Control (No intervencion No Diabetes) | 68,2 | 68,2 |
| 2. Controles que SI desarrollan DMG | 76,5 | 64,6 |
| 2. Controles que SI desarrollan DMG | 67,5 | 64,8 |
| 1. Control (No intervencion No Diabetes) | 70,8 | 66,9 |
| 1. Control (No intervencion No Diabetes) | 53,8 | 55,6 |
| 1. Control (No intervencion No Diabetes) | 71,6 | 64,8 |
| 1. Control (No intervencion No Diabetes) | 69,2 | 66,5 |
| 1. Control (No intervencion No Diabetes) | 51,0 | 46,4 |

| | | |
|---|---|---|
| 1. Control (No intervencion No Diabetes) | 62,5 | 57,6 |
| 1. Control (No intervencion No Diabetes) | 72,1 | 66,8 |
| 1. Control (No intervencion No Diabetes) | 65,0 | 60,3 |
| 1. Control (No intervencion No Diabetes) | 78,3 | 78,3 |
| 1. Control (No intervencion No Diabetes) | 53,0 | 48,0 |
| 2. Controles que SI desarrollan DMG | 71,1 | 61,5 |
| 1. Control (No intervencion No Diabetes) | 66,7 | 63,0 |
| 1. Control (No intervencion No Diabetes) | 59,9 | NAN |
| 1. Control (No intervencion No Diabetes) | 74,9 | 65,0 |
| 1. Control (No intervencion No Diabetes) | 66,1 | 62,9 |
| 1. Control (No intervencion No Diabetes) | 79,0 | 65,0 |
| 1. Control (No intervencion No Diabetes) | 57,5 | 56,3 |
| 1. Control (No intervencion No Diabetes) | 76,6 | 71,0 |
| 1. Control (No intervencion No Diabetes) | 67,7 | 52,7 |
| 1. Control (No intervencion No Diabetes) | 72,0 | 70,6 |
| 1. Control (No intervencion No Diabetes) | 70,0 | 63,2 |
| 1. Control (No intervencion No Diabetes) | 72,0 | 70,0 |
| 1. Control (No intervencion No Diabetes) | 63,8 | 57,1 |
| 1. Control (No intervencion No Diabetes) | 69,1 | 67,6 |
| 1. Control (No intervencion No Diabetes) | 53,0 | 51,4 |
| 1. Control (No intervencion No Diabetes) | 57,2 | 57,0 |
| 1. Control (No intervencion No Diabetes) | 62,7 | 58,5 |
| 1. Control (No intervencion No Diabetes) | 68,3 | 70,5 |
| 1. Control (No intervencion No Diabetes) | 59,6 | 54,4 |
| 1. Control (No intervencion No Diabetes) | 136,3 | 157,9 |
| 1. Control (No intervencion No Diabetes) | 81,5 | 83,7 |
| 2. Controles que SI desarrollan DMG | 63,0 | 47,9 |
| 2. Controles que SI desarrollan DMG | 88,7 | NAN |
| 1. Control (No intervencion No Diabetes) | 67,1 | 65,7 |
| 1. Control (No intervencion No Diabetes) | 69,1 | 69,4 |
| 1. Control (No intervencion No Diabetes) | 67,1 | 58,4 |
| 1. Control (No intervencion No Diabetes) | 65,2 | 64,8 |
| 2. Controles que SI desarrollan DMG | 69,1 | 67,7 |
| 1. Control (No intervencion No Diabetes) | 82,3 | 73,3 |
| 1. Control (No intervencion No Diabetes) | 62,4 | NAN |
| 1. Control (No intervencion No Diabetes) | 57,0 | 59,0 |
| 1. Control (No intervencion No Diabetes) | 58,5 | 51,1 |
| 2. Controles que SI desarrollan DMG | 58,8 | 59,3 |
| 1. Control (No intervencion No Diabetes) | 74,5 | 74,9 |
| 2. Controles que SI desarrollan DMG | 55,0 | 50,6 |
| 1. Control (No intervencion No Diabetes) | 54,3 | 48,1 |
| 2. Controles que SI desarrollan DMG | 69,0 | 75,5 |
| 1. Control (No intervencion No Diabetes) | 79,3 | 79,0 |
| 1. Control (No intervencion No Diabetes) | 59,4 | 53,8 |
| 1. Control (No intervencion No Diabetes) | 62,1 | 60,6 |
| 1. Control (No intervencion No Diabetes) | 71,7 | NAN |
| 1. Control (No intervencion No Diabetes) | 69,7 | 53,8 |
| 2. Controles que SI desarrollan DMG | 61,9 | 55,6 |
| 1. Control (No intervencion No Diabetes) | 68,0 | 63,0 |
| 1. Control (No intervencion No Diabetes) | 47,8 | NAN |

| | | |
|---|---|---|
| 1. Control (No intervencion No Diabetes) | 58,6 | 53,6 |
| 1. Control (No intervencion No Diabetes) | 49,3 | 44,1 |
| 1. Control (No intervencion No Diabetes) | 66,5 | 60,2 |
| 1. Control (No intervencion No Diabetes) | 53,0 | 48,5 |
| 1. Control (No intervencion No Diabetes) | 66,0 | 58,3 |
| 1. Control (No intervencion No Diabetes) | 55,4 | 51,1 |
| 1. Control (No intervencion No Diabetes) | 71,1 | 57,7 |
| 1. Control (No intervencion No Diabetes) | 64,9 | 58,1 |
| 1. Control (No intervencion No Diabetes) | 61,2 | 57,9 |
| 1. Control (No intervencion No Diabetes) | 72,2 | 61,5 |
| 1. Control (No intervencion No Diabetes) | 65,3 | 70,0 |
| 1. Control (No intervencion No Diabetes) | 67,2 | 69,9 |
| 3. Intervencion que NO DMG | 66,3 | 60,8 |
| 1. Control (No intervencion No Diabetes) | 65,5 | 59,7 |
| 2. Controles que SI desarrollan DMG | 71,1 | 71,5 |
| 1. Control (No intervencion No Diabetes) | 69,5 | 59,0 |
| 2. Controles que SI desarrollan DMG | 58,9 | 49,7 |
| 3. Intervencion que NO DMG | 51,7 | NAN |
| 2. Controles que SI desarrollan DMG | 58,1 | 48,0 |
| 1. Control (No intervencion No Diabetes) | 65,8 | 57,6 |
| 3. Intervencion que NO DMG | 60,5 | 56,3 |
| 1. Control (No intervencion No Diabetes) | 94,6 | 82,0 |
| 1. Control (No intervencion No Diabetes) | 72,1 | 68,4 |
| 3. Intervencion que NO DMG | 60,4 | 52,0 |
| 4. Intervencion que SI hacen DMG | 72,6 | 66,4 |
| 1. Control (No intervencion No Diabetes) | 52,5 | NAN |
| 1. Control (No intervencion No Diabetes) | 57,7 | 47,0 |
| 3. Intervencion que NO DMG | 64,7 | 62,4 |
| 4. Intervencion que SI hacen DMG | 70,7 | 78,6 |
| 3. Intervencion que NO DMG | 57,8 | 51,5 |
| 3. Intervencion que NO DMG | 86,1 | 85,0 |
| 2. Controles que SI desarrollan DMG | 65,5 | 64,5 |
| 3. Intervencion que NO DMG | 64,5 | 58,0 |
| 3. Intervencion que NO DMG | 60,7 | 56,7 |
| 2. Controles que SI desarrollan DMG | 64,6 | 63,5 |
| 3. Intervencion que NO DMG | 59,6 | NAN |
| 2. Controles que SI desarrollan DMG | 79,0 | 80,8 |
| 2. Controles que SI desarrollan DMG | 63,7 | 57,7 |
| 1. Control (No intervencion No Diabetes) | 70,7 | 64,0 |
| 3. Intervencion que NO DMG | 91,8 | 92,9 |
| 3. Intervencion que NO DMG | 79,4 | NAN |
| 4. Intervencion que SI hacen DMG | 87,0 | 90,0 |
| 2. Controles que SI desarrollan DMG | 73,8 | 69,0 |
| 3. Intervencion que NO DMG | 62,9 | NAN |
| 4. Intervencion que SI hacen DMG | 91,3 | 81,0 |
| 4. Intervencion que SI hacen DMG | 64,6 | 60,8 |
| 3. Intervencion que NO DMG | 67,1 | 61,7 |
| 1. Control (No intervencion No Diabetes) | 59,1 | 57,1 |
| 3. Intervencion que NO DMG | 78,8 | 71,6 |
| 2. Controles que SI desarrollan DMG | 82,4 | 76,3 |

| | | |
|---|---|---|
| 4. Intervencion que SI hacen DMG | 49,6 | 46,0 |
| 4. Intervencion que SI hacen DMG | 69,2 | 64,7 |
| 3. Intervencion que NO DMG | 63,5 | 57,5 |
| 3. Intervencion que NO DMG | 57,4 | 49,3 |
| 4. Intervencion que SI hacen DMG | 72,5 | 75,3 |
| 2. Controles que SI desarrollan DMG | 62,3 | 52,5 |
| 3. Intervencion que NO DMG | 58,5 | 52,0 |
| 3. Intervencion que NO DMG | 63,1 | 54,6 |
| 3. Intervencion que NO DMG | 67,2 | 64,5 |
| 3. Intervencion que NO DMG | 66,9 | 62,0 |
| 3. Intervencion que NO DMG | 79,5 | NAN |
| 3. Intervencion que NO DMG | 58,1 | 56,1 |
| 3. Intervencion que NO DMG | 59,8 | 55,1 |
| 3. Intervencion que NO DMG | 67,8 | 68,9 |
| 3. Intervencion que NO DMG | 57,0 | 52,6 |
| 4. Intervencion que SI hacen DMG | 104,0 | 96,0 |
| 3. Intervencion que NO DMG | 69,4 | 64,9 |
| 3. Intervencion que NO DMG | 72,5 | 69,7 |
| 4. Intervencion que SI hacen DMG | 77,0 | 76,8 |
| 3. Intervencion que NO DMG | 62,7 | 64,6 |
| 3. Intervencion que NO DMG | 71,4 | 71,5 |
| 3. Intervencion que NO DMG | 66,5 | 59,5 |
| 3. Intervencion que NO DMG | 65,2 | NAN |
| 3. Intervencion que NO DMG | 54,5 | 53,0 |
| 4. Intervencion que SI hacen DMG | 71,0 | 68,0 |
| 3. Intervencion que NO DMG | 63,5 | 58,1 |
| 3. Intervencion que NO DMG | 63,3 | 54,1 |
| 3. Intervencion que NO DMG | 58,4 | 55,0 |
| 3. Intervencion que NO DMG | 72,4 | 74,4 |
| 3. Intervencion que NO DMG | 65,2 | 56,7 |
| 3. Intervencion que NO DMG | 52,1 | NAN |
| 3. Intervencion que NO DMG | 58,8 | 55,4 |
| 3. Intervencion que NO DMG | 68,2 | 64,5 |
| 3. Intervencion que NO DMG | 58,3 | 52,7 |
| 4. Intervencion que SI hacen DMG | 69,0 | 65,4 |
| 4. Intervencion que SI hacen DMG | 69,0 | 57,9 |
| 3. Intervencion que NO DMG | 74,1 | 67,8 |
| 3. Intervencion que NO DMG | 75,1 | 73,2 |
| 3. Intervencion que NO DMG | 75,8 | 63,3 |
| 4. Intervencion que SI hacen DMG | 60,6 | NAN |
| 3. Intervencion que NO DMG | 58,7 | 51,0 |
| 4. Intervencion que SI hacen DMG | 83,6 | 84,0 |
| 3. Intervencion que NO DMG | 62,2 | 57,9 |
| 4. Intervencion que SI hacen DMG | 63,6 | 54,0 |
| 1. Control (No intervencion No Diabetes) | 79,5 | 73,3 |
| 3. Intervencion que NO DMG | 59,1 | 54,9 |
| 3. Intervencion que NO DMG | 61,4 | 62,9 |
| 3. Intervencion que NO DMG | 61,4 | 55,7 |
| 3. Intervencion que NO DMG | 53,6 | 50,4 |
| 3. Intervencion que NO DMG | 67,0 | 57,5 |

| | | |
|---|---|---|
| 2. Controles que SI desarrollan DMG | 81,2 | 80,0 |
| 3. Intervencion que NO DMG | 64,8 | 57,3 |
| 3. Intervencion que NO DMG | 62,0 | 58,1 |
| 3. Intervencion que NO DMG | 73,4 | 63,9 |
| 3. Intervencion que NO DMG | 54,8 | 54,1 |
| 3. Intervencion que NO DMG | 68,2 | 61,5 |
| 3. Intervencion que NO DMG | 83,5 | 73,8 |
| 2. Controles que SI desarrollan DMG | 83,3 | 97,0 |
| 3. Intervencion que NO DMG | 65,1 | 54,6 |
| 3. Intervencion que NO DMG | 60,0 | 54,4 |
| 3. Intervencion que NO DMG | 58,8 | 55,2 |
| 3. Intervencion que NO DMG | 64,4 | 68,9 |
| 3. Intervencion que NO DMG | 65,7 | 61,0 |
| 3. Intervencion que NO DMG | 82,0 | NAN |
| 3. Intervencion que NO DMG | 52,3 | 50,6 |
| 3. Intervencion que NO DMG | 73,7 | NAN |
| 3. Intervencion que NO DMG | 63,5 | 65,5 |
| 4. Intervencion que SI hacen DMG | 61,9 | 56,1 |
| 3. Intervencion que NO DMG | 63,6 | NAN |
| 3. Intervencion que NO DMG | 90,0 | 77,2 |
| 3. Intervencion que NO DMG | 73,8 | 75,5 |
| 2. Controles que SI desarrollan DMG | 54,0 | 46,1 |
| 3. Intervencion que NO DMG | 74,8 | 69,3 |
| 3. Intervencion que NO DMG | 60,2 | 54,0 |
| 3. Intervencion que NO DMG | 76,2 | 82,5 |
| 3. Intervencion que NO DMG | 69,4 | 67,2 |
| 4. Intervencion que SI hacen DMG | 61,3 | 56,5 |
| 3. Intervencion que NO DMG | 79,8 | 89,3 |
| 3. Intervencion que NO DMG | 80,9 | 78,6 |
| 3. Intervencion que NO DMG | 59,6 | NAN |
| 2. Controles que SI desarrollan DMG | 58,7 | 54,2 |
| 4. Intervencion que SI hacen DMG | 65,0 | 59,4 |
| 4. Intervencion que SI hacen DMG | 66,1 | 60,5 |
| 3. Intervencion que NO DMG | 59,7 | NAN |
| 3. Intervencion que NO DMG | 66,3 | 56,9 |
| 3. Intervencion que NO DMG | 71,8 | 65,5 |
| 3. Intervencion que NO DMG | 64,6 | 59,6 |
| 3. Intervencion que NO DMG | 53,5 | 46,9 |
| 3. Intervencion que NO DMG | 63,3 | 63,0 |
| 3. Intervencion que NO DMG | 52,3 | 53,0 |
| 3. Intervencion que NO DMG | 64,5 | 55,0 |
| 4. Intervencion que SI hacen DMG | 60,2 | 55,8 |
| 3. Intervencion que NO DMG | 63,7 | 60,7 |
| 4. Intervencion que SI hacen DMG | 66,2 | 56,7 |
| 3. Intervencion que NO DMG | 75,4 | 67,1 |
| 3. Intervencion que NO DMG | 65,3 | NAN |
| 3. Intervencion que NO DMG | 100,0 | NAN |
| 3. Intervencion que NO DMG | 67,7 | 66,3 |
| 3. Intervencion que NO DMG | 63,2 | NAN |
| 4. Intervencion que SI hacen DMG | 75,7 | 75,0 |

| | | |
|---|---|---|
| 3. Intervencion que NO DMG | 57,1 | 56,9 |
| 3. Intervencion que NO DMG | 62,7 | 61,7 |
| 4. Intervencion que SI hacen DMG | 78,0 | 83,1 |
| 4. Intervencion que SI hacen DMG | 75,0 | 71,0 |
| 4. Intervencion que SI hacen DMG | 66,3 | NAN |
| 1. Control (No intervencion No Diabetes) | 73,8 | 63,3 |
| 4. Intervencion que SI hacen DMG | 57,8 | 56,9 |
| 3. Intervencion que NO DMG | 48,5 | 49,9 |
| 3. Intervencion que NO DMG | 67,1 | 63,0 |
| 3. Intervencion que NO DMG | 81,8 | 80,0 |
| 2. Controles que SI desarrollan DMG | NAN | 70,3 |
| 3. Intervencion que NO DMG | 63,5 | 71,8 |
| 4. Intervencion que SI hacen DMG | 60,1 | 53,5 |
| 4. Intervencion que SI hacen DMG | 55,0 | 54,4 |
| 3. Intervencion que NO DMG | 69,9 | NAN |
| 3. Intervencion que NO DMG | 73,2 | 77,0 |
| 3. Intervencion que NO DMG | 59,9 | 58,5 |
| 3. Intervencion que NO DMG | 68,4 | 62,2 |
| 3. Intervencion que NO DMG | 65,7 | 64,4 |
| 3. Intervencion que NO DMG | 56,0 | 53,0 |
| 3. Intervencion que NO DMG | 62,3 | NAN |
| 3. Intervencion que NO DMG | 65,9 | 64,0 |
| 4. Intervencion que SI hacen DMG | 99,0 | 73,9 |
| 3. Intervencion que NO DMG | 88,3 | 84,0 |
| 4. Intervencion que SI hacen DMG | 69,4 | 63,6 |
| 3. Intervencion que NO DMG | 67,5 | NAN |
| 3. Intervencion que NO DMG | 82,3 | 86,0 |
| 3. Intervencion que NO DMG | 51,2 | 46,7 |
| 3. Intervencion que NO DMG | 67,4 | 58,0 |
| 3. Intervencion que NO DMG | 66,0 | 66,9 |
| 3. Intervencion que NO DMG | 61,6 | 55,5 |
| 4. Intervencion que SI hacen DMG | 80,7 | NAN |
| 4. Intervencion que SI hacen DMG | 69,1 | 65,2 |
| 3. Intervencion que NO DMG | 75,2 | 75,8 |
| 3. Intervencion que NO DMG | 67,7 | NAN |
| 1. Control (No intervencion No Diabetes) | 72,2 | NAN |
| 3. Intervencion que NO DMG | 62,2 | NAN |
| 3. Intervencion que NO DMG | 67,9 | 66,2 |
| 3. Intervencion que NO DMG | 86,0 | 81,3 |
| 3. Intervencion que NO DMG | 83,9 | NAN |
| 1. Control (No intervencion No Diabetes) | NAN | NAN |
| 3. Intervencion que NO DMG | 75,6 | 62,2 |
| 1. Control (No intervencion No Diabetes) | 81,1 | NAN |
| 3. Intervencion que NO DMG | 65,9 | 59,9 |
| 3. Intervencion que NO DMG | 63,2 | NAN |
| 3. Intervencion que NO DMG | 59,0 | NAN |
| 3. Intervencion que NO DMG | 57,4 | 50,9 |
| 3. Intervencion que NO DMG | 75,6 | NAN |
| 3. Intervencion que NO DMG | 61,1 | 52,8 |
| 2. Controles que SI desarrollan DMG | 88,6 | NAN |

| | | |
|---|---|---|
| 3. Intervencion que NO DMG | 72,5 | 63,6 |
| 4. Intervencion que SI hacen DMG | 73,4 | 72,9 |
| 4. Intervencion que SI hacen DMG | 79,1 | 61,0 |
| 3. Intervencion que NO DMG | 62,9 | NAN |
| 4. Intervencion que SI hacen DMG | 71,7 | NAN |
| 3. Intervencion que NO DMG | 72,4 | 65,5 |
| 3. Intervencion que NO DMG | 92,4 | 92,9 |
| 3. Intervencion que NO DMG | 70,9 | 65,6 |
| 3. Intervencion que NO DMG | 64,7 | 65,8 |
| 3. Intervencion que NO DMG | 79,0 | 76,5 |
| 2. Controles que SI desarrollan DMG | 65,4 | NAN |
| 2. Controles que SI desarrollan DMG | 72,0 | NAN |
| 3. Intervencion que NO DMG | 62,6 | NAN |
| 3. Intervencion que NO DMG | 101,3 | 101,0 |
| 4. Intervencion que SI hacen DMG | 59,1 | 50,8 |
| 3. Intervencion que NO DMG | 83,6 | 86,0 |

| FP Glucose (mg/dl) 24 gw | 1 h GTT (mg/dl) 24 gw | 2 h GTT (mg/dl) 24 gw |
|---|---|---|
| 76,0 | 64,0 | 77,0 |
| 86,0 | 101,0 | 84,0 |
| 88,0 | 112,0 | 96,0 |
| 105,0 | NAN | NAN |
| 91,0 | 151,0 | 129,0 |
| 87,0 | 117,0 | 96,0 |
| 88,0 | 89,0 | 92,0 |
| 91,0 | 148,0 | 119,0 |
| 98,0 | NAN | NAN |
| 96,0 | NAN | NAN |
| 82,0 | 126,0 | 154,0 |
| 90,0 | 171,0 | 114,0 |
| 92,0 | 118,0 | 109,0 |
| 88,0 | 133,0 | 92,0 |
| 84,0 | 110,0 | 84,0 |
| 87,0 | 70,0 | 78,0 |
| 79,0 | 103,0 | 91,0 |
| 92,0 | NAN | NAN |
| 77,0 | 120,0 | 76,0 |
| 97,0 | NAN | NAN |
| 84,0 | 122,0 | 131,0 |
| 82,0 | 140,0 | 115,0 |
| 88,0 | 141,0 | 106,0 |
| 80,0 | 173,0 | 188,0 |
| 92,0 | 150,0 | 133,0 |
| 84,0 | 77,0 | 79,0 |
| 93,0 | 145,0 | 107,0 |
| 80,0 | 102,0 | 95,0 |
| 103,0 | NAN | NAN |
| 88,0 | 153,0 | 136,0 |
| 93,0 | 162,0 | 144,0 |
| 93,0 | NAN | NAN |
| 100,0 | 107,0 | 126,0 |
| 84,0 | 144,0 | 108,0 |
| 83,0 | 120,0 | 109,0 |
| 81,0 | 56,0 | 76,0 |
| 80,0 | 105,0 | 86,0 |
| 82,0 | 184,0 | 163,0 |
| 86,0 | 178,0 | 112,0 |
| 79,0 | 120,0 | 111,0 |
| 93,0 | 142,0 | 107,0 |
| 102,0 | NAN | NAN |
| 82,0 | 77,0 | 80,0 |
| 87,0 | 137,0 | 74,0 |
| 88,0 | 157,0 | 115,0 |
| 78,0 | 60,0 | 86,0 |
| 83,0 | 95,0 | 108,0 |

| | | |
|---|---|---|
| 81,0 | 104,0 | 110,0 |
| 79,0 | 78,0 | 60,0 |
| 81,0 | 101,0 | NAN |
| 91,0 | 115,0 | 111,0 |
| 77,0 | 148,0 | 112,0 |
| 93,0 | 145,0 | 127,0 |
| 81,0 | 77,0 | 83,0 |
| 80,0 | 153,0 | 126,0 |
| 81,0 | 101,0 | 100,0 |
| 79,0 | 144,0 | 77,0 |
| 87,0 | 111,0 | 105,0 |
| 72,0 | 104,0 | 98,0 |
| 82,0 | 97,0 | 69,0 |
| 90,0 | 134,0 | 101,0 |
| 90,0 | 151,0 | 121,0 |
| 87,0 | 89,0 | 91,0 |
| 87,0 | 155,0 | 150,0 |
| 83,0 | 161,0 | 118,0 |
| 82,0 | 131,0 | 95,0 |
| 83,0 | 96,0 | 118,0 |
| 82,0 | 128,0 | 100,0 |
| 88,0 | 127,0 | 121,0 |
| 86,0 | 119,0 | 100,0 |
| 80,0 | 87,0 | 120,0 |
| 77,0 | 94,0 | 103,0 |
| 82,0 | 124,0 | 110,0 |
| 94,0 | NAN | NAN |
| 96,0 | NAN | NAN |
| 81,0 | 84,0 | 99,0 |
| 83,0 | 63,0 | 91,0 |
| 77,0 | 83,0 | 67,0 |
| 91,0 | 80,0 | 88,0 |
| 96,0 | NAN | NAN |
| 70,0 | 138,0 | 101,0 |
| 84,0 | 119,0 | 98,0 |
| 85,0 | 110,0 | 95,0 |
| 69,0 | 137,0 | 102,0 |
| 81,0 | 194,0 | 156,0 |
| 82,0 | 132,0 | 120,0 |
| 86,0 | 203,0 | 165,0 |
| 91,0 | 127,0 | 139,0 |
| 99,0 | NAN | NAN |
| 84,0 | 168,0 | 93,0 |
| 85,0 | 106,0 | 108,0 |
| 81,0 | 75,0 | 86,0 |
| 85,0 | 148,0 | 145,0 |
| 84,0 | 84,0 | 108,0 |
| 95,0 | 152,0 | 179,0 |
| 90,0 | 135,0 | 102,0 |
| 82,0 | 123,0 | 132,0 |

| | | |
|---|---|---|
| 86,0 | 159,0 | 80,0 |
| 84,0 | 69,0 | 79,0 |
| 82,0 | 73,0 | 79,0 |
| 88,0 | 113,0 | 140,0 |
| 87,0 | 144,0 | 88,0 |
| 88,0 | 125,0 | 130,0 |
| 82,0 | 98,0 | 82,0 |
| 87,0 | 175,0 | 151,0 |
| 88,0 | 139,0 | 142,0 |
| 90,0 | 114,0 | 83,0 |
| 73,0 | 102,0 | 125,0 |
| 87,0 | 146,0 | 115,0 |
| 80,0 | 97,0 | 89,0 |
| 80,0 | 157,0 | 123,0 |
| 96,0 | NAN | NAN |
| 89,0 | 133,0 | 99,0 |
| 80,0 | 158,0 | 164,0 |
| 89,0 | 109,0 | 137,0 |
| 74,0 | 172,0 | 166,0 |
| 82,0 | 107,0 | 111,0 |
| 85,0 | 124,0 | 95,0 |
| 89,0 | 134,0 | 127,0 |
| 83,0 | 140,0 | 69,0 |
| 86,0 | 107,0 | 103,0 |
| 97,0 | NAN | NAN |
| 82,0 | 108,0 | 85,0 |
| 80,0 | 142,0 | 101,0 |
| 84,0 | 136,0 | 122,0 |
| 99,0 | NAN | NAN |
| 84,0 | 114,0 | 90,0 |
| 90,0 | 152,0 | 144,0 |
| 91,0 | 206,0 | 96,0 |
| 81,0 | 149,0 | 72,0 |
| 86,0 | 109,0 | 105,0 |
| 88,0 | 100,0 | 165,0 |
| 82,0 | 87,0 | 74,0 |
| 98,0 | NAN | NAN |
| 93,0 | 130,0 | 126,0 |
| 85,0 | 104,0 | 103,0 |
| 80,0 | 130,0 | 108,0 |
| 83,0 | 170,0 | 130,0 |
| 86,0 | 160,0 | 165,0 |
| 96,0 | NAN | NAN |
| 79,0 | 95,0 | 96,0 |
| 84,0 | 180,0 | 146,0 |
| 86,0 | 208,0 | 181,0 |
| 82,0 | 133,0 | 110,0 |
| 85,0 | 59,0 | 80,0 |
| 76,0 | 83,0 | 58,0 |
| 95,0 | 166,0 | 123,0 |

| | | |
|---|---|---|
| 93,0 | 156,0 | 136,0 |
| 97,0 | NAN | NAN |
| 82,0 | 109,0 | 83,0 |
| 82,0 | 150,0 | 145,0 |
| 98,0 | NAN | NAN |
| 73,0 | 167,0 | 167,0 |
| 81,0 | 138,0 | 87,0 |
| 86,0 | 110,0 | 88,0 |
| 87,0 | 110,0 | 110,0 |
| 80,0 | 68,0 | 82,0 |
| 76,0 | 108,0 | 73,0 |
| 83,0 | 127,0 | 115,0 |
| 90,0 | 129,0 | 112,0 |
| 85,0 | 170,0 | 136,0 |
| 76,0 | 132,0 | 128,0 |
| 87,0 | 205,0 | 63,0 |
| 79,0 | 133,0 | 103,0 |
| 77,0 | 122,0 | 86,0 |
| 95,0 | 152,0 | 116,0 |
| 81,0 | 128,0 | 90,0 |
| 79,0 | 101,0 | 97,0 |
| 86,0 | 149,0 | 107,0 |
| 87,0 | 129,0 | 134,0 |
| 89,0 | 153,0 | 116,0 |
| 93,0 | NAN | NAN |
| 87,0 | 99,0 | 78,0 |
| 83,0 | 99,0 | 91,0 |
| 75,0 | 130,0 | 136,0 |
| 90,0 | 112,0 | 102,0 |
| 82,0 | 113,0 | 103,0 |
| 81,0 | 126,0 | 114,0 |
| 78,0 | 109,0 | 99,0 |
| 90,0 | 179,0 | 124,0 |
| 81,0 | 90,0 | 82,0 |
| 93,0 | 147,0 | 114,0 |
| 97,0 | 168,0 | 131,0 |
| 83,0 | 114,0 | 119,0 |
| 80,0 | 96,0 | 130,0 |
| 76,0 | 127,0 | 116,0 |
| 100,0 | 214,0 | 133,0 |
| 81,0 | 105,0 | 75,0 |
| 93,0 | 155,0 | 112,0 |
| 83,0 | 108,0 | 92,0 |
| 84,0 | 181,0 | 139,0 |
| 88,0 | 145,0 | 107,0 |
| 81,0 | 101,0 | 90,0 |
| 85,0 | 117,0 | 126,0 |
| 85,0 | 173,0 | 120,0 |
| 77,0 | 130,0 | 112,0 |
| 87,0 | 138,0 | 133,0 |

| | | |
|---|---|---|
| 93,0 | 189,0 | 145,0 |
| 86,0 | 111,0 | 112,0 |
| 90,0 | 142,0 | 139,0 |
| 85,0 | 115,0 | 102,0 |
| 83,0 | 118,0 | 131,0 |
| 82,0 | 105,0 | 65,0 |
| 83,0 | 86,0 | 103,0 |
| 92,0 | 80,0 | 90,0 |
| 83,0 | 94,0 | 97,0 |
| 82,0 | 111,0 | 113,0 |
| 70,0 | 123,0 | 108,0 |
| 81,0 | 167,0 | 128,0 |
| 89,0 | 129,0 | 129,0 |
| 88,0 | 152,0 | 109,0 |
| 79,0 | 162,0 | 112,0 |
| 84,0 | 114,0 | 94,0 |
| 77,0 | 105,0 | 63,0 |
| 88,0 | 208,0 | 126,0 |
| 87,0 | 89,0 | 77,0 |
| 118,0 | NAN | NAN |
| 85,0 | 99,0 | 108,0 |
| 88,0 | 189,0 | 169,0 |
| 87,0 | 175,0 | 146,0 |
| 76,0 | 90,0 | 99,0 |
| 84,0 | 99,0 | 87,0 |
| 88,0 | 165,0 | 110,0 |
| 94,0 | 180,0 | 133,0 |
| 75,0 | 102,0 | 94,0 |
| 90,0 | 110,0 | 78,0 |
| 82,0 | 116,0 | 106,0 |
| 93,0 | 108,0 | 93,0 |
| 98,0 | 110,0 | 93,0 |
| 95,0 | 147,0 | 112,0 |
| 76,0 | 117,0 | 108,0 |
| 87,0 | 158,0 | 131,0 |
| 87,0 | 85,0 | 84,0 |
| 83,0 | 123,0 | 104,0 |
| 78,0 | 95,0 | 84,0 |
| 85,0 | 87,0 | 115,0 |
| 74,0 | 82,0 | 97,0 |
| 81,0 | 159,0 | 132,0 |
| 90,0 | 192,0 | 156,0 |
| 78,0 | 121,0 | 113,0 |
| 98,0 | NAN | NAN |
| 79,0 | 118,0 | 89,0 |
| 77,0 | 102,0 | 100,0 |
| 86,0 | 65,0 | 69,0 |
| 91,0 | 158,0 | 150,0 |
| 82,0 | 83,0 | 68,0 |
| 105,0 | NAN | NAN |

| | | |
|---|---|---|
| 82,0 | 96,0 | 108,0 |
| 84,0 | 83,0 | 96,0 |
| 98,0 | NAN | NAN |
| 94,0 | 158,0 | 69,0 |
| 89,0 | 158,0 | 153,0 |
| 80,0 | 88,0 | 65,0 |
| 79,0 | 154,0 | 155,0 |
| 83,0 | 135,0 | 113,0 |
| 82,0 | 101,0 | 118,0 |
| 82,0 | 151,0 | 109,0 |
| 94,0 | NAN | NAN |
| 79,0 | 133,0 | 106,0 |
| 93,0 | 179,0 | 133,0 |
| 100,0 | NAN | NAN |
| 85,0 | 119,0 | 111,0 |
| 82,0 | 108,0 | 116,0 |
| 80,0 | 78,0 | 74,0 |
| 84,0 | 81,0 | 103,0 |
| 86,0 | 123,0 | 91,0 |
| 81,0 | 91,0 | 88,0 |
| 80,0 | 126,0 | 145,0 |
| 83,0 | 127,0 | 103,0 |
| 94,0 | 125,0 | 94,0 |
| 75,0 | 88,0 | 102,0 |
| 97,0 | 89,0 | 94,0 |
| 82,0 | 165,0 | 144,0 |
| 85,0 | 110,0 | 117,0 |
| 82,0 | 102,0 | 104,0 |
| 81,0 | 151,0 | 122,0 |
| 88,0 | 120,0 | 123,0 |
| 85,0 | 125,0 | 112,0 |
| 85,0 | 166,0 | 173,0 |
| 100,0 | NAN | NAN |
| 79,0 | 71,0 | 73,0 |
| 82,0 | 146,0 | 141,0 |
| 84,0 | 133,0 | 129,0 |
| 81,0 | 86,0 | 91,0 |
| 73,0 | 142,0 | 64,0 |
| 89,0 | 126,0 | 100,0 |
| 87,0 | 109,0 | 152,0 |
| 89,0 | 125,0 | 91,0 |
| 86,0 | 98,0 | 88,0 |
| 86,0 | 108,0 | 93,0 |
| 80,0 | 160,0 | 140,0 |
| 91,0 | 177,0 | 105,0 |
| 81,0 | 110,0 | 84,0 |
| 82,0 | 133,0 | 93,0 |
| 85,0 | 138,0 | 106,0 |
| 79,0 | 100,0 | 105,0 |
| 92,0 | 106,0 | 105,0 |

| | | |
|---|---|---|
| 79,0 | 92,0 | 113,0 |
| 101,0 | NAN | NAN |
| 97,0 | NAN | NAN |
| 87,0 | 144,0 | 119,0 |
| 94,0 | NAN | NAN |
| 84,0 | 133,0 | 119,0 |
| 88,1 | 125,0 | 100,0 |
| 82,0 | 107,0 | 116,0 |
| 85,0 | 111,0 | 111,0 |
| 88,0 | 168,0 | 100,0 |
| 95,0 | NAN | NAN |
| 92,0 | 179,0 | 155,0 |
| 82,0 | 103,0 | 82,0 |
| 88,0 | 154,0 | 125,0 |
| 80,0 | 190,0 | 188,0 |
| 90,0 | 154,0 | 144,0 |

| FP Glucose (mg/dl) 2 years PD | 2 h GTT (mg/dl) 2 years PD | sBP (mm Hg) 24 GW | dBP (mm Hg) 24 GW | sBP (mm Hg) 2 Yrs PD |
|---|---|---|---|---|
| 82,0 | 63,0 | NAN | NAN | 99,0 |
| 92,0 | 108,0 | NAN | NAN | 117,0 |
| 96,0 | 114,0 | NAN | NAN | NAN |
| 101,0 | 110,0 | NAN | NAN | NAN |
| 103,0 | 74,0 | NAN | NAN | 108,0 |
| 98,0 | 99,0 | NAN | NAN | 116,0 |
| 91,0 | NAN | NAN | NAN | NAN |
| 90,0 | 96,0 | NAN | NAN | 103,0 |
| 93,0 | NAN | NAN | NAN | 122,0 |
| 101,0 | 128,0 | NAN | NAN | NAN |
| 88,0 | NAN | NAN | NAN | 106,0 |
| 105,0 | NAN | NAN | NAN | 113,0 |
| 89,0 | NAN | NAN | NAN | NAN |
| 96,0 | 86,0 | NAN | NAN | 111,0 |
| 79,0 | NAN | NAN | NAN | 113,0 |
| 89,0 | NAN | 99,0 | 64,0 | 105,0 |
| 75,0 | NAN | NAN | NAN | NAN |
| 90,0 | 100,0 | 99,0 | 58,0 | 111,0 |
| 94,0 | 102,0 | 107,0 | 72,0 | 111,0 |
| 100,0 | NAN | 106,0 | 61,0 | 105,0 |
| 91,0 | 121,0 | 102,0 | 68,0 | 116,0 |
| 97,0 | 91,0 | 104,0 | 57,0 | 115,0 |
| 101,0 | NAN | NAN | NAN | NAN |
| 87,0 | NAN | 101,0 | 76,0 | 99,0 |
| 98,0 | NAN | 129,0 | 73,0 | 113,0 |
| 97,0 | NAN | 94,0 | 53,0 | NAN |
| 97,0 | NAN | 110,0 | 69,0 | 111,0 |
| 100,0 | NAN | 94,0 | 55,0 | 115,0 |
| 104,0 | 80,0 | 106,0 | 64,0 | 120,0 |
| 94,0 | 85,0 | 114,0 | 71,0 | 106,0 |
| 105,0 | NAN | 100,0 | 74,0 | 115,0 |
| 102,0 | 112,0 | 100,0 | 60,0 | 102,0 |
| 96,0 | NAN | 98,0 | 47,0 | 106,0 |
| 94,0 | NAN | 117,0 | 72,0 | 116,0 |
| 90,0 | NAN | 107,0 | 59,0 | NAN |
| 86,0 | 99,0 | 88,0 | 53,0 | 103,0 |
| 98,0 | 94,0 | 89,0 | 53,0 | 101,0 |
| 87,0 | NAN | 103,0 | 59,0 | 130,0 |
| 96,0 | NAN | 91,0 | 56,0 | NAN |
| 84,0 | NAN | 108,0 | 63,0 | NAN |
| 97,0 | NAN | 110,0 | 63,0 | NAN |
| 101,0 | 90,0 | 106,0 | 65,0 | 125,0 |
| 92,0 | 83,0 | 104,0 | 62,0 | NAN |
| 88,0 | 76,0 | 112,0 | 56,0 | 111,0 |
| 94,0 | NAN | 90,0 | 64,0 | NAN |
| 86,0 | 90,0 | 94,0 | 49,0 | 97,0 |
| 94,0 | NAN | 100,0 | 53,0 | 106,0 |

| | | | | |
|---|---|---|---|---|
| 83,0 | 97,0 | 92,0 | 62,0 | 94,0 |
| 88,0 | NAN | 90,0 | 52,0 | 108,0 |
| 88,0 | NAN | 109,0 | 71,0 | 122,0 |
| 97,0 | NAN | 108,0 | 80,0 | 115,0 |
| 90,0 | NAN | 106,0 | 57,0 | 104,0 |
| 94,0 | 184,0 | 127,0 | 76,0 | 135,0 |
| 85,0 | 82,0 | 120,0 | 74,0 | 121,0 |
| 91,0 | NAN | 97,0 | 58,0 | NAN |
| 85,0 | NAN | 110,0 | 60,0 | 102,0 |
| 82,0 | NAN | 103,0 | 53,0 | 91,0 |
| 92,0 | 95,0 | 126,0 | 80,0 | 110,0 |
| 85,0 | 111,0 | 107,0 | 53,0 | NAN |
| 95,0 | 101,0 | 85,0 | 57,0 | 88,0 |
| 95,0 | NAN | 118,0 | 63,0 | 124,0 |
| 99,0 | 93,0 | 102,0 | 49,0 | 98,0 |
| 88,0 | 95,0 | 108,0 | 60,0 | NAN |
| 107,0 | NAN | 93,0 | 54,0 | 107,0 |
| 90,0 | 139,0 | 89,0 | 53,0 | 112,0 |
| 92,0 | NAN | 117,0 | 65,0 | 134,0 |
| 96,0 | 84,0 | 98,0 | 65,0 | 120,0 |
| 87,0 | NAN | 110,0 | 67,0 | 117,0 |
| 91,0 | 113,0 | 100,0 | 52,0 | 100,0 |
| 90,0 | NAN | 103,0 | 67,0 | 116,0 |
| 88,0 | NAN | 103,0 | 64,0 | 117,0 |
| 88,0 | 94,0 | NAN | NAN | NAN |
| 101,0 | NAN | 117,0 | 48,0 | NAN |
| 94,0 | 70,0 | 99,0 | 59,0 | 104,0 |
| 101,0 | NAN | 106,0 | 63,0 | NAN |
| 92,0 | 104,0 | 105,0 | 62,0 | 114,0 |
| 85,0 | NAN | 108,0 | 61,0 | 120,0 |
| 95,0 | NAN | 88,0 | 56,0 | 100,0 |
| 90,0 | 157,0 | 90,0 | 52,0 | 118,0 |
| 104,0 | 109,0 | 102,0 | 63,0 | 117,0 |
| 84,0 | 115,0 | 103,0 | 62,0 | NAN |
| 89,0 | 73,0 | 96,0 | 56,0 | 105,0 |
| 89,0 | 99,0 | 95,0 | 51,0 | 95,0 |
| 86,0 | 47,0 | 110,0 | 61,0 | 103,0 |
| 117,0 | NAN | 112,0 | 69,0 | 120,0 |
| 86,0 | NAN | 122,0 | 77,0 | 115,0 |
| 100,0 | 118,0 | 107,0 | 73,0 | 120,0 |
| 94,0 | 149,0 | 93,0 | 58,0 | 97,0 |
| 108,0 | 157,0 | 91,0 | 58,0 | 120,0 |
| 92,0 | NAN | 115,0 | 75,0 | 117,0 |
| 101,0 | NAN | 87,0 | 55,0 | 100,0 |
| 81,0 | NAN | 97,0 | 64,0 | 117,0 |
| 97,0 | 106,0 | 96,0 | 49,0 | NAN |
| 98,0 | NAN | 104,0 | 74,0 | 114,0 |
| 91,0 | 99,0 | 120,0 | 74,0 | 120,0 |
| 90,0 | NAN | 109,0 | 63,0 | NAN |
| 97,0 | 109,0 | 153,0 | 101,0 | 92,0 |

| | | | | |
|---|---|---|---|---|
| 94,0 | 89,0 | 102,0 | 53,0 | 100,0 |
| 84,0 | 70,0 | 98,0 | 57,0 | 107,0 |
| 94,0 | 76,0 | 88,0 | 67,0 | 111,0 |
| 89,0 | 78,0 | 90,0 | 56,0 | 100,0 |
| 88,0 | 98,0 | 112,0 | 68,0 | 113,0 |
| 91,0 | 94,0 | 117,0 | 76,0 | 105,0 |
| 94,0 | NAN | 92,0 | 55,0 | NAN |
| 91,0 | NAN | 86,0 | 46,0 | 102,0 |
| 101,0 | NAN | 104,0 | 62,0 | NAN |
| 92,0 | NAN | 96,0 | 68,0 | NAN |
| 87,0 | NAN | 100,0 | 67,0 | NAN |
| 102,0 | NAN | 120,0 | 63,0 | 127,0 |
| 93,0 | 75,0 | 99,0 | 59,0 | 117,0 |
| 94,0 | NAN | 100,0 | 52,0 | 108,0 |
| 96,0 | 99,0 | 115,0 | 79,0 | 113,0 |
| 94,0 | 80,0 | 86,0 | 54,0 | 98,0 |
| 88,0 | NAN | 113,0 | 60,0 | 104,0 |
| 87,0 | NAN | 103,0 | 66,0 | NAN |
| 82,0 | 87,0 | 102,0 | 68,0 | NAN |
| 90,0 | 77,0 | 103,0 | 64,0 | NAN |
| 90,0 | 87,0 | 101,0 | 62,0 | 93,0 |
| 86,0 | NAN | 107,0 | 72,0 | 120,0 |
| 93,0 | 49,0 | 96,0 | 59,0 | NAN |
| 79,0 | 93,0 | 98,0 | 59,0 | 94,0 |
| 96,0 | 117,0 | 99,0 | 62,0 | 119,0 |
| 87,0 | NAN | 97,0 | 50,0 | 119,0 |
| 89,0 | NAN | 91,0 | 57,0 | 96,0 |
| 93,0 | NAN | 146,0 | 83,0 | NAN |
| 96,0 | 96,0 | 93,0 | 53,0 | 108,0 |
| 92,0 | 81,0 | 99,0 | 62,0 | 117,0 |
| 93,0 | NAN | 121,0 | 73,0 | 123,0 |
| 102,0 | NAN | 116,0 | 52,0 | NAN |
| 96,0 | 64,0 | 116,0 | 64,0 | 122,0 |
| 94,0 | NAN | 90,0 | 64,0 | NAN |
| 112,0 | NAN | 104,0 | 63,0 | NAN |
| 86,0 | NAN | 105,0 | 65,0 | NAN |
| 101,0 | NAN | 94,0 | 55,0 | 132,0 |
| 107,0 | 91,0 | 108,0 | 74,0 | NAN |
| 94,0 | NAN | 107,0 | 70,0 | 118,0 |
| 89,0 | NAN | 130,0 | 78,0 | NAN |
| 91,0 | 87,0 | 110,0 | 65,0 | NAN |
| 105,0 | NAN | 136,0 | 89,0 | 138,0 |
| 99,0 | 93,0 | 101,0 | 61,0 | 113,0 |
| 97,0 | 73,0 | 97,0 | 59,0 | NAN |
| 90,0 | 104,0 | 117,0 | 68,0 | 118,0 |
| 94,0 | NAN | 108,0 | 64,0 | 110,0 |
| 93,0 | NAN | 110,0 | 73,0 | 95,0 |
| 96,0 | NAN | 88,0 | 54,0 | 104,0 |
| 82,0 | 89,0 | 119,0 | 68,0 | 113,0 |
| 93,0 | 75,0 | 110,0 | 64,0 | 107,0 |

| | | | | |
|---|---|---|---|---|
| 95,0 | 114,0 | 107,0 | 75,0 | NAN |
| 95,0 | NAN | 112,0 | 60,0 | 96,0 |
| 82,0 | 100,0 | 118,0 | 72,0 | 123,0 |
| 94,0 | NAN | 98,0 | 62,0 | 94,0 |
| 97,0 | 102,0 | 109,0 | 86,0 | 108,0 |
| 93,0 | NAN | 99,0 | 68,0 | 110,0 |
| 83,0 | 66,0 | 111,0 | 73,0 | 113,0 |
| 87,0 | NAN | 87,0 | 53,0 | 92,0 |
| 87,0 | 77,0 | 112,0 | 67,0 | 120,0 |
| 102,0 | NAN | 97,0 | 51,0 | 105,0 |
| 84,0 | NAN | 111,0 | 72,0 | NAN |
| 87,0 | NAN | 93,0 | 49,0 | 114,0 |
| 94,0 | NAN | 106,0 | 63,0 | 117,0 |
| 100,0 | NAN | 112,0 | 78,0 | 114,0 |
| 95,0 | 89,0 | 110,0 | 73,0 | 116,0 |
| 91,0 | NAN | 105,0 | 68,0 | NAN |
| 97,0 | 107,0 | 137,0 | 81,0 | 129,0 |
| 88,0 | NAN | 90,0 | 57,0 | 113,0 |
| 94,0 | 82,0 | 107,0 | 66,0 | 101,0 |
| 93,0 | NAN | 113,0 | 74,0 | 119,0 |
| 87,0 | NAN | 97,0 | 60,0 | 117,0 |
| 87,0 | 92,0 | 101,0 | 58,0 | 110,0 |
| 96,0 | 93,0 | 104,0 | 69,0 | NAN |
| 92,0 | NAN | 85,0 | 50,0 | 101,0 |
| 109,0 | 118,0 | 88,0 | 54,0 | NAN |
| 95,0 | NAN | 102,0 | 55,0 | 119,0 |
| 80,0 | NAN | 101,0 | 61,0 | 93,0 |
| 99,0 | NAN | 107,0 | 65,0 | 112,0 |
| 95,0 | 102,0 | 129,0 | 77,0 | 132,0 |
| 93,0 | 97,0 | 105,0 | 47,0 | NAN |
| 89,0 | 64,0 | 86,0 | 46,0 | NAN |
| 88,0 | NAN | 86,0 | 50,0 | 99,0 |
| 86,0 | NAN | 106,0 | 60,0 | 121,0 |
| 103,0 | NAN | 99,0 | 72,0 | 97,0 |
| 97,0 | 141,0 | 111,0 | 63,0 | 117,0 |
| 95,0 | 68,0 | 82,0 | 55,0 | 98,0 |
| 86,0 | NAN | 96,0 | 64,0 | 116,0 |
| 84,0 | NAN | 102,0 | 58,0 | NAN |
| 83,0 | NAN | 110,0 | 66,0 | 93,0 |
| 93,0 | 97,0 | 98,0 | 55,0 | NAN |
| 93,0 | 141,0 | 85,0 | 51,0 | NAN |
| 85,0 | NAN | 122,0 | 59,0 | 129,0 |
| 91,0 | 62,0 | 92,0 | 59,0 | NAN |
| 94,0 | 71,0 | 130,0 | 63,0 | 129,0 |
| 95,0 | NAN | 122,0 | 57,0 | 142,0 |
| 89,0 | NAN | 102,0 | 59,0 | 107,0 |
| 91,0 | NAN | 94,0 | 57,0 | NAN |
| 93,0 | NAN | 94,0 | 56,0 | 115,0 |
| 86,0 | NAN | 94,0 | 73,0 | 101,0 |
| 97,0 | 100,0 | 96,0 | 61,0 | NAN |

| | | | | |
|---|---|---|---|---|
| 96,0 | 77,0 | 120,0 | 74,0 | 110,0 |
| 86,0 | NAN | 105,0 | 60,0 | 123,0 |
| 100,0 | 91,0 | 95,0 | 84,0 | 107,0 |
| 82,0 | NAN | 105,0 | 64,0 | 99,0 |
| 99,0 | 116,0 | 96,0 | 56,0 | 108,0 |
| 101,0 | NAN | 107,0 | 69,0 | 123,0 |
| 89,0 | 89,0 | 109,0 | 58,0 | 107,0 |
| 100,0 | 87,0 | 127,0 | 81,0 | 135,0 |
| 88,0 | 98,0 | 134,0 | 74,0 | 130,0 |
| 92,0 | NAN | 104,0 | 59,0 | 100,0 |
| 96,0 | 105,0 | 90,0 | 55,0 | NAN |
| 87,0 | NAN | 107,0 | 64,0 | 120,0 |
| 97,0 | NAN | 103,0 | 60,0 | 114,0 |
| 91,0 | 102,0 | 118,0 | 73,0 | NAN |
| 88,0 | NAN | 99,0 | 62,0 | 134,0 |
| 107,0 | 92,0 | 115,0 | 71,0 | NAN |
| 80,0 | 93,0 | 94,0 | 63,0 | 110,0 |
| 87,0 | NAN | 101,0 | 69,0 | NAN |
| 99,0 | 105,0 | 97,0 | 57,0 | NAN |
| 96,0 | 99,0 | 122,0 | 76,0 | 135,0 |
| 95,0 | 92,0 | 109,0 | 54,0 | 110,0 |
| 93,0 | NAN | 92,0 | 65,0 | 97,0 |
| 88,0 | NAN | 120,0 | 71,0 | 113,0 |
| 76,0 | NAN | 96,0 | 85,0 | 120,0 |
| 95,0 | NAN | 95,0 | 62,0 | NAN |
| 97,0 | 87,0 | 103,0 | 57,0 | 108,0 |
| 97,0 | 97,0 | 106,0 | 59,0 | 110,0 |
| 94,0 | 110,0 | NAN | NAN | 112,0 |
| 93,0 | 60,0 | 99,0 | 65,0 | 115,0 |
| 92,0 | NAN | 104,0 | 57,0 | NAN |
| 94,0 | 95,0 | 107,0 | 68,0 | 119,0 |
| 100,0 | 87,0 | 99,0 | 59,0 | 118,0 |
| 98,0 | NAN | 108,0 | 61,0 | 109,0 |
| 103,0 | 118,0 | 99,0 | 55,0 | 111,0 |
| 91,0 | NAN | 97,0 | 56,0 | 106,0 |
| 98,0 | 93,0 | 115,0 | 71,0 | 117,0 |
| 89,0 | NAN | 109,0 | 70,0 | 119,0 |
| 87,0 | 84,0 | 102,0 | 57,0 | 91,0 |
| 98,0 | 90,0 | 96,0 | 57,0 | 87,0 |
| 83,0 | 106,0 | 103,0 | 60,0 | 121,0 |
| 101,0 | 107,0 | 108,0 | 72,0 | 119,0 |
| 110,0 | 140,0 | 105,0 | 52,0 | 113,0 |
| 91,0 | 128,0 | 102,0 | 43,0 | 101,0 |
| 94,0 | NAN | 118,0 | 68,0 | 112,0 |
| 87,0 | NAN | 96,0 | 62,0 | 108,0 |
| 104,0 | NAN | 97,0 | 57,0 | NAN |
| 92,0 | 77,0 | 113,0 | 62,0 | 104,0 |
| 98,0 | NAN | 112,0 | 66,0 | NAN |
| 84,0 | NAN | 106,0 | 62,0 | NAN |
| 97,0 | 128,0 | 110,0 | 69,0 | 117,0 |

| | | | | |
|---|---|---|---|---|
| 93,0 | NAN | 88,0 | 57,0 | 111,0 |
| 80,0 | 84,0 | 106,0 | 60,0 | 103,0 |
| 111,0 | 153,0 | 112,0 | 71,0 | NAN |
| 104,0 | 131,0 | 103,0 | 61,0 | NAN |
| 89,0 | 62,0 | 112,0 | 62,0 | 109,0 |
| 87,0 | NAN | 106,0 | 60,0 | 109,0 |
| 85,0 | 102,0 | 100,0 | 67,0 | 116,0 |
| 86,0 | NAN | 98,0 | 64,0 | 107,0 |
| 99,0 | 115,0 | 94,0 | 61,0 | 117,0 |
| 96,0 | 80,0 | 110,0 | 65,0 | 118,0 |
| 103,0 | NAN | NAN | NAN | 108,0 |
| 93,0 | 103,0 | 90,0 | 47,0 | 106,0 |
| 87,0 | NAN | 88,0 | 50,0 | 100,0 |
| 102,0 | 115,0 | 116,0 | 81,0 | 103,0 |
| 96,0 | 126,0 | 122,0 | 75,0 | 137,0 |
| 93,0 | 117,0 | 113,0 | 73,0 | 119,0 |
| 99,0 | NAN | 100,0 | 59,0 | NAN |
| 82,0 | 77,0 | 101,0 | 48,0 | 117,0 |
| 100,0 | 104,0 | 112,0 | 66,0 | NAN |
| 89,0 | 88,0 | 101,0 | 47,0 | 86,0 |
| 92,0 | NAN | 101,0 | 57,0 | 103,0 |
| 93,0 | NAN | 111,0 | 68,0 | 122,0 |
| 96,0 | 82,0 | 93,0 | 62,0 | 103,0 |
| 90,0 | NAN | 107,0 | 68,0 | 113,0 |
| 97,0 | 132,0 | 96,0 | 54,0 | NAN |
| 77,0 | NAN | 96,0 | 60,0 | NAN |
| 95,0 | 119,0 | 109,0 | 73,0 | 120,0 |
| 91,0 | 108,0 | 100,0 | 59,0 | 99,0 |
| 90,0 | NAN | 95,0 | 73,0 | 90,0 |
| 95,0 | 100,0 | 106,0 | 54,0 | 101,0 |
| 84,0 | 79,0 | 103,0 | 64,0 | 135,0 |
| 103,0 | 250,0 | 110,0 | 66,0 | NAN |
| 108,0 | 76,0 | NAN | NAN | 104,0 |
| 85,0 | 69,0 | 112,0 | 66,0 | 124,0 |
| 87,0 | 106,0 | 91,0 | 64,0 | NAN |
| 93,0 | NAN | 86,0 | 48,0 | NAN |
| 96,0 | NAN | 106,0 | 47,0 | NAN |
| 89,0 | 67,0 | 88,0 | 61,0 | 104,0 |
| 91,0 | 94,0 | 127,0 | 61,0 | 116,0 |
| 96,0 | 110,0 | 100,0 | 60,0 | NAN |
| 92,0 | 115,0 | NAN | NAN | 118,0 |
| 95,0 | 121,0 | 125,0 | 70,0 | 112,0 |
| 89,0 | 91,0 | 98,0 | 53,0 | NAN |
| 99,0 | 73,0 | 89,0 | 73,0 | 103,0 |
| 95,0 | NAN | 104,0 | 66,0 | NAN |
| 90,0 | 100,0 | 103,0 | 67,0 | NAN |
| 101,0 | NAN | 104,0 | 55,0 | 123,0 |
| 86,0 | NAN | 108,0 | 57,0 | NAN |
| 92,0 | 83,0 | 98,0 | 66,0 | 107,0 |
| 107,0 | 105,0 | 134,0 | 74,0 | NAN |

| | | | | |
|---|---|---|---|---|
| 92,0 | 127,0 | 115,0 | 81,0 | 122,0 |
| 97,0 | 146,0 | 117,0 | 70,0 | 122,0 |
| 85,0 | NAN | 129,0 | 76,0 | 112,0 |
| 98,0 | 100,0 | 84,0 | 49,0 | 85,0 |
| 96,0 | 85,0 | 127,0 | 74,0 | NAN |
| 87,0 | 118,0 | 106,0 | 55,0 | NAN |
| 98,0 | 102,0 | 120,0 | 74,0 | 125,0 |
| 98,0 | 66,0 | 100,0 | 41,0 | 113,0 |
| 92,0 | 88,0 | 110,0 | 61,0 | 117,0 |
| 110,0 | NAN | 142,0 | 89,0 | 168,0 |
| 108,0 | 136,0 | 90,0 | 59,0 | NAN |
| 105,0 | NAN | 98,0 | 62,0 | 108,0 |
| 95,0 | 126,0 | 114,0 | 82,0 | NAN |
| 103,0 | NAN | 100,0 | 50,0 | NAN |
| 99,0 | 129,0 | 112,0 | 60,0 | 117,0 |
| 106,0 | 78,0 | 100,0 | 60,0 | NAN |

| dBP (mm Hg) 2 Yrs PD | T-Chol (mg/dl) 24 gw | T-Chol (mg/dl) 2 years PD | Triglycerides (g/L) 24 gw | Triglycerides (g/L) 2 Yrs PD |
|---|---|---|---|---|
| 64,0 | 295,0 | 152,0 | 151,0 | 46,0 |
| 73,0 | NAN | 193,0 | NAN | 76,0 |
| NAN | 300,0 | 206,0 | 158,0 | 101,0 |
| NAN | NAN | 210,0 | NAN | 187,0 |
| 72,0 | 210,0 | 148,0 | 83,0 | 35,0 |
| 75,0 | 133,0 | 105,0 | 142,0 | 50,0 |
| NAN | 251,0 | 170,0 | 118,0 | 58,0 |
| 71,0 | NAN | 225,0 | NAN | 67,0 |
| 77,0 | 237,0 | 196,0 | 125,0 | 83,0 |
| NAN | 210,0 | 134,0 | 147,0 | 183,0 |
| 72,0 | NAN | 179,0 | NAN | 82,0 |
| 73,0 | 282,0 | 208,0 | 165,0 | 62,0 |
| NAN | 233,0 | 183,0 | 124,0 | 48,0 |
| 70,0 | 219,0 | 167,0 | 163,0 | 93,0 |
| 69,0 | 222,0 | 138,0 | 149,0 | 58,0 |
| 71,0 | 288,0 | 194,0 | 146,0 | 52,0 |
| NAN | 244,0 | 114,0 | 99,0 | 56,0 |
| 65,0 | 160,0 | 102,0 | 182,0 | 54,0 |
| 74,0 | NAN | 172,0 | NAN | 72,0 |
| 75,0 | 268,0 | 223,0 | 259,0 | 136,0 |
| 70,0 | 300,0 | 168,0 | 166,0 | 55,0 |
| 63,0 | 218,0 | 165,0 | 168,0 | 75,0 |
| NAN | 259,0 | 226,0 | 159,0 | 150,0 |
| 66,0 | NAN | 150,0 | 146,0 | 37,0 |
| 80,0 | 292,0 | 205,0 | 253,0 | 134,0 |
| NAN | 248,0 | 150,0 | 245,0 | 81,0 |
| 78,0 | 362,0 | 202,0 | 217,0 | 166,0 |
| 74,0 | NAN | 188,0 | NAN | 190,0 |
| 80,0 | 301,0 | 237,0 | 125,0 | 73,0 |
| 64,0 | 279,0 | 196,0 | 212,0 | 118,0 |
| 74,0 | 256,0 | 213,0 | 354,0 | 138,0 |
| 64,0 | NAN | 184,0 | NAN | 51,0 |
| 73,0 | 293,0 | 158,0 | 125,0 | 35,0 |
| 68,0 | 150,0 | 123,0 | 256,0 | 65,0 |
| NAN | 234,0 | 177,0 | 161,0 | 83,0 |
| 67,0 | NAN | 137,0 | NAN | 82,0 |
| 68,0 | NAN | 172,0 | NAN | 45,0 |
| 75,0 | 191,0 | 148,0 | 161,0 | 112,0 |
| NAN | 212,0 | 213,0 | 210,0 | 109,0 |
| NAN | 257,0 | 155,0 | 177,0 | 69,0 |
| NAN | 246,0 | 163,0 | 171,0 | 94,0 |
| 75,0 | 301,0 | 217,0 | 160,0 | 151,0 |
| NAN | 168,0 | 148,0 | 62,0 | 40,0 |
| 66,0 | 345,0 | 164,0 | 268,0 | 122,0 |
| NAN | 298,0 | 176,0 | 127,0 | 90,0 |
| 61,0 | 285,0 | 183,0 | 155,0 | 52,0 |
| 63,0 | 289,0 | 187,0 | 255,0 | 66,0 |

| | | | | |
|---|---|---|---|---|
| 54,0 | 360,0 | 151,0 | 112,0 | 71,0 |
| 61,0 | 282,0 | 181,0 | 214,0 | 59,0 |
| 81,0 | 207,0 | 173,0 | 198,0 | 106,0 |
| 84,0 | 237,0 | 180,0 | 123,0 | 69,0 |
| 69,0 | 197,0 | 181,0 | 92,0 | 60,0 |
| 83,0 | 221,0 | 185,0 | 178,0 | 104,0 |
| 79,0 | 231,0 | 124,0 | 133,0 | 68,0 |
| NAN | 258,0 | 174,0 | 146,0 | 54,0 |
| 71,0 | 244,0 | 149,0 | 183,0 | 39,0 |
| 60,0 | NAN | 223,0 | NAN | 80,0 |
| 69,0 | NAN | 159,0 | NAN | 51,0 |
| NAN | 238,0 | 155,0 | 118,0 | 42,0 |
| 56,0 | 172,0 | 118,0 | 130,0 | 44,0 |
| 78,0 | 256,0 | 213,0 | 183,0 | 93,0 |
| 55,0 | NAN | 151,0 | NAN | 155,0 |
| NAN | 324,0 | 203,0 | 202,0 | 87,0 |
| 74,0 | 338,0 | 239,0 | 240,0 | 65,0 |
| 71,0 | 295,0 | 212,0 | 159,0 | 114,0 |
| 85,0 | 259,0 | 191,0 | 170,0 | 79,0 |
| 79,0 | 281,0 | 184,0 | 103,0 | 109,0 |
| 78,0 | 227,0 | 132,0 | 290,0 | 64,0 |
| 58,0 | 247,0 | 181,0 | 140,0 | 74,0 |
| 79,0 | NAN | 172,0 | NAN | 201,0 |
| 82,0 | 248,0 | 132,0 | 110,0 | 38,0 |
| NAN | NAN | 161,0 | NAN | 117,0 |
| NAN | 229,0 | 115,0 | 214,0 | 41,0 |
| 70,0 | 230,0 | 187,0 | 150,0 | 38,0 |
| NAN | 235,0 | 160,0 | 254,0 | 75,0 |
| 71,0 | 255,0 | 221,0 | 128,0 | 100,0 |
| 79,0 | 272,0 | 171,0 | 100,0 | 65,0 |
| 70,0 | 217,0 | 160,0 | 152,0 | 58,0 |
| 75,0 | 174,0 | 177,0 | 138,0 | 103,0 |
| 78,0 | 214,0 | 167,0 | 111,0 | 84,0 |
| NAN | NAN | 223,0 | NAN | 80,0 |
| 70,0 | 246,0 | 181,0 | 163,0 | 55,0 |
| 69,0 | 323,0 | 192,0 | 211,0 | 95,0 |
| 63,0 | 312,0 | 170,0 | 241,0 | 81,0 |
| 80,0 | 212,0 | 152,0 | 139,0 | 87,0 |
| 63,0 | 252,0 | 198,0 | 182,0 | 122,0 |
| 65,0 | NAN | 158,0 | NAN | 62,0 |
| 66,0 | 304,0 | 203,0 | 178,0 | 54,0 |
| 70,0 | NAN | 239,0 | NAN | 130,0 |
| 86,0 | 282,0 | 183,0 | 294,0 | 219,0 |
| 58,0 | NAN | 179,0 | NAN | 65,0 |
| 80,0 | 179,0 | 142,0 | 50,0 | 30,0 |
| NAN | 279,0 | 183,0 | 207,0 | 78,0 |
| 70,0 | 274,0 | 179,0 | 124,0 | 41,0 |
| 97,0 | 309,0 | 224,0 | 220,0 | 67,0 |
| NAN | NAN | 165,0 | NAN | 76,0 |
| 51,0 | 348,0 | 169,0 | 186,0 | 45,0 |

| | | | | |
|---|---|---|---|---|
| 61,0 | 245,0 | 160,0 | 128,0 | 47,0 |
| 56,0 | 254,0 | 181,0 | 161,0 | 204,0 |
| 82,0 | NAN | 129,0 | NAN | 54,0 |
| 64,0 | 168,0 | 111,0 | 117,0 | 41,0 |
| 78,0 | 148,0 | 148,0 | 115,0 | 111,0 |
| 66,0 | 223,0 | 168,0 | 118,0 | 56,0 |
| NAN | 273,0 | 182,0 | 104,0 | 54,0 |
| 66,0 | 294,0 | 242,0 | 153,0 | 79,0 |
| NAN | NAN | 167,0 | NAN | 199,0 |
| NAN | 208,0 | 165,0 | 197,0 | 69,0 |
| NAN | 254,0 | 159,0 | 86,0 | 70,0 |
| 79,0 | 222,0 | 176,0 | 128,0 | 93,0 |
| 73,0 | 280,0 | 200,0 | 115,0 | 66,0 |
| 74,0 | 253,0 | 168,0 | 109,0 | 56,0 |
| 66,0 | 266,0 | 171,0 | 167,0 | 98,0 |
| 68,0 | 249,0 | 188,0 | 154,0 | 74,0 |
| 70,0 | 239,0 | 143,0 | 134,0 | 38,0 |
| NAN | NAN | 164,0 | NAN | 62,0 |
| NAN | NAN | 135,0 | NAN | 71,0 |
| NAN | NAN | 163,0 | NAN | 47,0 |
| 59,0 | 263,0 | 188,0 | 121,0 | 60,0 |
| 76,0 | NAN | 166,0 | NAN | 44,0 |
| NAN | 276,0 | 181,0 | 227,0 | 104,0 |
| 64,0 | 219,0 | 145,0 | 166,0 | 78,0 |
| 75,0 | 249,0 | 214,0 | 249,0 | 228,0 |
| 64,0 | 214,0 | 166,0 | 170,0 | 76,0 |
| 67,0 | 175,0 | 174,0 | 100,0 | 57,0 |
| NAN | 212,0 | 189,0 | 144,0 | 61,0 |
| 65,0 | 143,0 | 103,0 | 117,0 | 110,0 |
| 72,0 | 267,0 | 201,0 | 154,0 | 53,0 |
| 78,0 | 208,0 | 181,0 | 241,0 | 112,0 |
| NAN | 249,0 | 225,0 | 145,0 | 79,0 |
| 76,0 | 184,0 | 162,0 | 125,0 | 54,0 |
| NAN | 252,0 | 182,0 | 76,0 | 47,0 |
| NAN | NAN | 186,0 | NAN | 271,0 |
| NAN | 220,0 | 163,0 | 202,0 | 65,0 |
| 82,0 | 206,0 | 203,0 | 180,0 | 103,0 |
| NAN | 305,0 | 189,0 | 163,0 | 65,0 |
| 84,0 | 225,0 | 200,0 | 116,0 | 58,0 |
| NAN | 208,0 | 122,0 | 147,0 | 52,0 |
| NAN | 243,0 | 154,0 | 138,0 | 77,0 |
| 93,0 | NAN | 304,0 | NAN | 359,0 |
| 81,0 | 181,0 | 121,0 | 92,0 | 45,0 |
| NAN | NAN | 164,0 | NAN | 63,0 |
| 82,0 | 251,0 | 211,0 | 147,0 | 79,0 |
| 68,0 | 340,0 | 182,0 | 122,0 | 77,0 |
| 62,0 | 254,0 | 174,0 | 95,0 | 39,0 |
| 61,0 | 327,0 | 205,0 | 181,0 | 80,0 |
| 66,0 | NAN | 195,0 | NAN | 34,0 |
| 74,0 | 250,0 | 202,0 | 222,0 | 72,0 |

| | | | | |
|---|---|---|---|---|
| NAN | 263,0 | 191,0 | 141,0 | 51,0 |
| 64,0 | NAN | 153,0 | NAN | 50,0 |
| 83,0 | NAN | 176,0 | NAN | 68,0 |
| 75,0 | NAN | 204,0 | NAN | 66,0 |
| 68,0 | 250,0 | 172,0 | 161,0 | 73,0 |
| 64,0 | 314,0 | 172,0 | 202,0 | 59,0 |
| 76,0 | 305,0 | 208,0 | 176,0 | 74,0 |
| 72,0 | 229,0 | 130,0 | 110,0 | 40,0 |
| 70,0 | 250,0 | 116,0 | 154,0 | 34,0 |
| 64,0 | 294,0 | 230,0 | 97,0 | 57,0 |
| NAN | 188,0 | 119,0 | 116,0 | 64,0 |
| 75,0 | 288,0 | 171,0 | 195,0 | 50,0 |
| 76,0 | 336,0 | 186,0 | 127,0 | 51,0 |
| 74,0 | 312,0 | 245,0 | 170,0 | 51,0 |
| 83,0 | NAN | 220,0 | NAN | 60,0 |
| NAN | NAN | 142,0 | NAN | 59,0 |
| 80,0 | 325,0 | 202,0 | 238,0 | 102,0 |
| 67,0 | 244,0 | 183,0 | 224,0 | 99,0 |
| 79,0 | 323,0 | 215,0 | 241,0 | 102,0 |
| 68,0 | 283,0 | 196,0 | 226,0 | 64,0 |
| 76,0 | 234,0 | 160,0 | 198,0 | 95,0 |
| 80,0 | 315,0 | 175,0 | 165,0 | 53,0 |
| NAN | 223,0 | 172,0 | 193,0 | 154,0 |
| 63,0 | 323,0 | 222,0 | 234,0 | 76,0 |
| NAN | 268,0 | 208,0 | 193,0 | 92,0 |
| 77,0 | 247,0 | 173,0 | 118,0 | 42,0 |
| 55,0 | 243,0 | 146,0 | 99,0 | 60,0 |
| 83,0 | NAN | 185,0 | NAN | 69,0 |
| 84,0 | 260,0 | 194,0 | 208,0 | 84,0 |
| NAN | 270,0 | 200,0 | 179,0 | 43,0 |
| NAN | 226,0 | 170,0 | 165,0 | 70,0 |
| 68,0 | 225,0 | 162,0 | 168,0 | 60,0 |
| 64,0 | 264,0 | 200,0 | 162,0 | 60,0 |
| 63,0 | 257,0 | 159,0 | 103,0 | 56,0 |
| 79,0 | 220,0 | 209,0 | 200,0 | 114,0 |
| 67,0 | 199,0 | 128,0 | 184,0 | 102,0 |
| 62,0 | 267,0 | 176,0 | 241,0 | 68,0 |
| NAN | 198,0 | 146,0 | 155,0 | 52,0 |
| 65,0 | 226,0 | 163,0 | 191,0 | 55,0 |
| NAN | 253,0 | 208,0 | 287,0 | 68,0 |
| NAN | 269,0 | 193,0 | 119,0 | 52,0 |
| 75,0 | 216,0 | 153,0 | 119,0 | 54,0 |
| NAN | NAN | 140,0 | NAN | 49,0 |
| 77,0 | 267,0 | 192,0 | 131,0 | 55,0 |
| 61,0 | 250,0 | 143,0 | 97,0 | 51,0 |
| 62,0 | NAN | 204,0 | NAN | 51,0 |
| NAN | 239,0 | 205,0 | 132,0 | 82,0 |
| 79,0 | 279,0 | 184,0 | 135,0 | 57,0 |
| 66,0 | 232,0 | 168,0 | 122,0 | 57,0 |
| NAN | 295,0 | 200,0 | 141,0 | 48,0 |

| | | | | |
|---|---|---|---|---|
| 77,0 | 263,0 | 193,0 | 179,0 | 125,0 |
| 83,0 | 200,0 | 154,0 | 207,0 | 58,0 |
| 59,0 | NAN | 185,0 | NAN | 77,0 |
| 62,0 | 242,0 | 179,0 | 109,0 | 54,0 |
| 70,0 | 257,0 | 219,0 | 150,0 | 62,0 |
| 76,0 | 297,0 | 169,0 | 155,0 | 52,0 |
| 62,0 | 230,0 | 148,0 | 243,0 | 56,0 |
| 75,0 | 241,0 | 204,0 | 117,0 | 87,0 |
| 70,0 | 222,0 | 178,0 | 228,0 | 84,0 |
| 59,0 | 302,0 | 180,0 | 161,0 | 93,0 |
| NAN | 244,0 | 169,0 | 112,0 | 49,0 |
| 72,0 | 267,0 | 152,0 | 92,0 | 42,0 |
| 74,0 | NAN | 184,0 | NAN | 47,0 |
| NAN | 245,0 | 186,0 | 197,0 | 107,0 |
| 81,0 | 263,0 | 234,0 | 101,0 | 64,0 |
| NAN | 289,0 | 185,0 | 155,0 | 62,0 |
| 74,0 | 264,0 | 189,0 | 206,0 | 79,0 |
| NAN | 307,0 | 183,0 | 140,0 | 50,0 |
| NAN | 190,0 | 168,0 | 114,0 | 54,0 |
| 79,0 | NAN | 133,0 | NAN | 71,0 |
| 65,0 | 251,0 | 201,0 | 175,0 | 81,0 |
| 58,0 | NAN | 166,0 | NAN | 75,0 |
| 78,0 | 299,0 | 229,0 | 222,0 | 138,0 |
| 78,0 | 260,0 | 161,0 | 123,0 | 54,0 |
| NAN | 207,0 | 129,0 | 144,0 | 59,0 |
| 63,0 | 248,0 | 175,0 | 161,0 | 106,0 |
| 74,0 | NAN | 175,0 | NAN | 62,0 |
| 73,0 | 271,0 | 182,0 | 170,0 | 84,0 |
| 81,0 | 244,0 | 163,0 | 113,0 | 45,0 |
| NAN | 301,0 | 196,0 | 130,0 | 87,0 |
| 81,0 | 210,0 | 169,0 | 159,0 | 69,0 |
| 67,0 | NAN | 152,0 | NAN | 48,0 |
| 71,0 | 245,0 | 155,0 | 135,0 | 60,0 |
| 68,0 | NAN | 214,0 | NAN | 58,0 |
| 67,0 | NAN | 177,0 | NAN | 39,0 |
| 73,0 | 272,0 | 173,0 | 243,0 | 63,0 |
| 79,0 | 231,0 | 170,0 | 138,0 | 54,0 |
| 59,0 | 261,0 | 140,0 | 171,0 | 48,0 |
| 60,0 | 278,0 | 173,0 | 150,0 | 83,0 |
| 86,0 | 338,0 | 177,0 | 221,0 | 93,0 |
| 77,0 | 338,0 | 199,0 | 229,0 | 77,0 |
| 60,0 | 222,0 | 189,0 | 200,0 | 99,0 |
| 58,0 | 276,0 | 182,0 | 162,0 | 71,0 |
| 69,0 | 224,0 | 180,0 | 176,0 | 55,0 |
| 73,0 | 247,0 | 148,0 | 153,0 | 49,0 |
| NAN | NAN | 170,0 | NAN | 63,0 |
| 63,0 | 195,0 | 162,0 | 50,0 | 40,0 |
| NAN | 289,0 | 241,0 | 270,0 | 372,0 |
| NAN | 249,0 | 158,0 | 107,0 | 43,0 |
| 71,0 | 241,0 | 181,0 | 239,0 | 117,0 |

| | | | | |
|---|---|---|---|---|
| 71,0 | NAN | 232,0 | NAN | 111,0 |
| 65,0 | 254,0 | 182,0 | 140,0 | 72,0 |
| NAN | 230,0 | 196,0 | 161,0 | 63,0 |
| NAN | NAN | 199,0 | NAN | 145,0 |
| 67,0 | 280,0 | 181,0 | 175,0 | 91,0 |
| 65,0 | 235,0 | 160,0 | 102,0 | 42,0 |
| 74,0 | 180,0 | 146,0 | 136,0 | 44,0 |
| 67,0 | 231,0 | 136,0 | 181,0 | 89,0 |
| 77,0 | 188,0 | 136,0 | 67,0 | 40,0 |
| 83,0 | 231,0 | 184,0 | 173,0 | 82,0 |
| 68,0 | NAN | 183,0 | NAN | 56,0 |
| 69,0 | NAN | 208,0 | NAN | 62,0 |
| 58,0 | 266,0 | 209,0 | 145,0 | 67,0 |
| 60,0 | 250,0 | 233,0 | 150,0 | 99,0 |
| 85,0 | NAN | 215,0 | NAN | 121,0 |
| 80,0 | 315,0 | 247,0 | 257,0 | 195,0 |
| NAN | 250,0 | 149,0 | 147,0 | 37,0 |
| 70,0 | 339,0 | 225,0 | 154,0 | 77,0 |
| NAN | 236,0 | 195,0 | 89,0 | 53,0 |
| 58,0 | NAN | 185,0 | NAN | 54,0 |
| 59,0 | NAN | 114,0 | NAN | 58,0 |
| 73,0 | 246,0 | 173,0 | 119,0 | 71,0 |
| 68,0 | 318,0 | 170,0 | 165,0 | 48,0 |
| 74,0 | NAN | 195,0 | NAN | 68,0 |
| NAN | 216,0 | 210,0 | 94,0 | 67,0 |
| NAN | 232,0 | 170,0 | 185,0 | 68,0 |
| 83,0 | 236,0 | 176,0 | 142,0 | 78,0 |
| 58,0 | 317,0 | 223,0 | 247,0 | 86,0 |
| 54,0 | NAN | 171,0 | NAN | 67,0 |
| 56,0 | 368,0 | 200,0 | 211,0 | 78,0 |
| 89,0 | 258,0 | 200,0 | 143,0 | 85,0 |
| NAN | 309,0 | 237,0 | 149,0 | 151,0 |
| 61,0 | NAN | 167,0 | NAN | 36,0 |
| 75,0 | 260,0 | 192,0 | 140,0 | 75,0 |
| NAN | 217,0 | 173,0 | 218,0 | 111,0 |
| NAN | 305,0 | 182,0 | 147,0 | 53,0 |
| NAN | NAN | 203,0 | NAN | 62,0 |
| 72,0 | 231,0 | 149,0 | 213,0 | 68,0 |
| 60,0 | 307,0 | 180,0 | 185,0 | 64,0 |
| NAN | 269,0 | 194,0 | 94,0 | 49,0 |
| 74,0 | 247,0 | 162,0 | 211,0 | 86,0 |
| 75,0 | NAN | 160,0 | NAN | 69,0 |
| NAN | 202,0 | 138,0 | 138,0 | 58,0 |
| 64,0 | 346,0 | 163,0 | 208,0 | 54,0 |
| NAN | NAN | 183,0 | NAN | 48,0 |
| NAN | 240,0 | 177,0 | 92,0 | 51,0 |
| 78,0 | 244,0 | 164,0 | 93,0 | 79,0 |
| NAN | 367,0 | 184,0 | 232,0 | 71,0 |
| 73,0 | 286,0 | 178,0 | 183,0 | 60,0 |
| NAN | 174,0 | 157,0 | 167,0 | 94,0 |

| | | | | |
|---|---|---|---|---|
| 77,0 | 243,0 | 143,0 | 242,0 | 61,0 |
| 78,0 | 220,0 | 195,0 | 220,0 | 195,0 |
| 85,0 | 318,0 | 214,0 | 205,0 | 90,0 |
| 62,0 | 201,0 | 141,0 | 137,0 | 71,0 |
| NAN | NAN | 241,0 | NAN | 67,0 |
| NAN | 279,0 | 209,0 | 166,0 | 84,0 |
| 83,0 | 175,0 | 136,0 | 112,0 | 69,0 |
| 63,0 | 278,0 | 131,0 | 121,0 | 38,0 |
| 67,0 | 355,0 | 238,0 | 222,0 | 70,0 |
| 111,0 | 236,0 | 214,0 | 454,0 | 383,0 |
| NAN | NAN | 162,0 | NAN | 188,0 |
| 68,0 | 230,0 | 162,0 | 117,0 | 41,0 |
| NAN | 219,0 | 117,0 | 164,0 | 97,0 |
| NAN | 250,0 | 178,0 | 106,0 | 43,0 |
| 77,0 | 208,0 | 122,0 | 130,0 | 40,0 |
| NAN | 192,0 | 155,0 | 155,0 | 87,0 |

| HbA1c-IFCC % 24 gw | HbA1c-IFCC % 2 Yrs PD | TSH mcUI/mL 24 gw | FT4 (ng/dl) 24 gw | TSH mcUI/mL 2 Yrs PD |
|---|---|---|---|---|
| 5,1 | 5,2 | 3,20 | 9,02 | 2,43 |
| 4,9 | 5,3 | NAN | NAN | 2,57 |
| 5,3 | 5,4 | 2,08 | 6,47 | 1,63 |
| 5,6 | 5,4 | NAN | NAN | 2,93 |
| 4,7 | 5,3 | 1,70 | 6,78 | 1,56 |
| 5,0 | 5,3 | 2,97 | 8,30 | 3,24 |
| 5,2 | 5,5 | 2,52 | 6,22 | 1,84 |
| 4,9 | 5,5 | NAN | NAN | 2,78 |
| 5,1 | NAN | NAN | NAN | 1,28 |
| 5,1 | 6,0 | 3,71 | 6,71 | 2,99 |
| 4,6 | 5,1 | NAN | NAN | 1,50 |
| 5,2 | 5,5 | 0,83 | 6,59 | 0,81 |
| 5,2 | 5,6 | 1,00 | 6,57 | 1,13 |
| 5,1 | 5,4 | 0,88 | 5,37 | NAN |
| 5,0 | 5,3 | 1,68 | 6,72 | 1,36 |
| 4,7 | 5,5 | 1,29 | 8,87 | 1,10 |
| 4,8 | 5,2 | 1,34 | 7,17 | 1,19 |
| 4,5 | 4,7 | 0,66 | 6,78 | 0,18 |
| 5,4 | 5,5 | 1,13 | 8,80 | 2,87 |
| 5,7 | 5,8 | 1,06 | 6,71 | 2,84 |
| 4,6 | 4,9 | 2,71 | 7,56 | 0,85 |
| 5,4 | 5,6 | 3,05 | 5,49 | 4,65 |
| 5,4 | NAN | 1,45 | 5,92 | 4,40 |
| 4,7 | 5,2 | 2,55 | 6,36 | 0,79 |
| 5,1 | 5,3 | 3,36 | 5,75 | 2,81 |
| 4,9 | 4,7 | 5,32 | 7,24 | 4,87 |
| 5,2 | 5,1 | 3,15 | 5,21 | 3,96 |
| 5,0 | 5,5 | 3,36 | 6,17 | 2,28 |
| 5,1 | 5,3 | 3,66 | 6,31 | 1,11 |
| 5,3 | 5,2 | NAN | NAN | 0,57 |
| 5,0 | 5,1 | NAN | NAN | 1,00 |
| 5,0 | 5,4 | NAN | NAN | 0,93 |
| 5,7 | 5,8 | 2,65 | 4,33 | 2,95 |
| 4,7 | 5,1 | 3,18 | 7,97 | 2,68 |
| 4,8 | 4,9 | 0,61 | 6,45 | 0,52 |
| 4,8 | 5,4 | NAN | NAN | 1,13 |
| 4,9 | 5,4 | NAN | NAN | 0,58 |
| 5,1 | 5,5 | 2,57 | 9,16 | 2,31 |
| 5,2 | 5,4 | 1,36 | 5,99 | 1,11 |
| 5,0 | 5,3 | 0,88 | 6,92 | 1,07 |
| 4,7 | 5,0 | 1,61 | 7,55 | 1,28 |
| 5,4 | 5,7 | 1,97 | 6,90 | 1,65 |
| 4,7 | 5,1 | 0,47 | 7,37 | 1,79 |
| 5,1 | 5,3 | 3,70 | 6,86 | 1,94 |
| 5,3 | 5,5 | 1,80 | 6,78 | 1,81 |
| 4,7 | 5,0 | 1,78 | 8,57 | 1,05 |
| 5,4 | 4,9 | 2,16 | 7,38 | 1,82 |

| | | | | |
|---|---|---|---|---|
| 5,1 | 5,8 | 1,13 | 7,49 | 4,98 |
| 4,7 | 5,1 | 1,52 | 8,33 | 1,81 |
| 5,1 | 5,5 | 1,45 | 6,87 | 1,11 |
| 5,4 | 5,5 | 1,97 | 7,44 | 2,38 |
| 5,0 | 5,2 | 0,18 | 9,36 | 0,87 |
| 5,3 | 5,5 | 2,44 | 6,02 | 0,99 |
| 5,7 | 5,3 | 0,62 | 6,48 | 0,81 |
| 5,3 | 5,1 | 0,77 | 8,18 | NAN |
| 5,1 | 5,2 | 3,96 | 5,98 | 2,29 |
| 5,0 | NAN | 1,79 | 5,65 | 3,81 |
| 5,5 | 5,5 | NAN | NAN | 1,85 |
| 4,9 | 5,2 | 1,06 | 7,14 | 1,11 |
| 4,9 | 5,4 | 1,71 | 7,73 | 1,55 |
| 5,3 | 5,1 | 1,79 | 7,73 | 2,30 |
| 5,0 | 5,5 | NAN | NAN | 0,81 |
| 5,1 | 5,1 | 2,10 | 7,43 | 1,56 |
| 4,8 | 5,3 | 3,28 | 6,47 | 2,38 |
| 5,1 | 5,4 | 1,37 | 8,19 | 1,33 |
| 4,6 | 4,9 | 0,54 | 8,00 | 4,34 |
| 4,9 | 4,7 | 1,61 | 6,85 | 2,38 |
| 4,8 | 5,1 | 2,18 | 6,40 | 1,14 |
| 5,4 | 5,4 | 2,22 | 7,41 | 4,19 |
| 5,8 | 5,9 | NAN | NAN | 1,78 |
| NAN | 5,5 | 0,95 | 6,84 | 1,26 |
| 4,2 | 5,1 | 0,70 | 7,09 | 2,86 |
| 4,9 | 5,4 | 3,14 | 6,95 | 4,77 |
| 4,8 | 5,1 | 1,47 | 6,45 | 0,70 |
| 5,2 | 5,3 | NAN | NAN | 4,00 |
| 5,0 | 5,6 | 2,44 | 8,58 | 1,76 |
| 5,2 | 5,6 | 1,03 | 7,20 | 0,64 |
| 4,9 | 5,3 | 1,36 | 6,78 | 0,92 |
| 5,1 | 5,5 | 3,32 | 5,82 | 2,62 |
| 5,3 | 5,6 | 2,22 | 7,00 | 1,06 |
| 5,2 | 5,3 | NAN | NAN | 2,40 |
| 5,0 | 5,4 | 2,15 | 7,86 | 3,29 |
| 4,9 | 5,2 | 2,50 | 6,99 | 1,70 |
| 4,5 | 4,9 | 2,66 | 5,39 | 2,39 |
| 5,4 | 6,0 | 2,21 | 6,07 | 1,69 |
| 5,0 | 5,5 | 0,15 | 7,74 | 3,16 |
| 4,9 | 5,1 | NAN | NAN | 2,36 |
| 5,3 | 5,6 | 2,24 | 7,85 | 1,81 |
| 5,1 | 5,6 | NAN | NAN | 2,68 |
| 5,2 | 5,7 | 0,55 | 7,66 | 0,77 |
| 5,3 | 4,5 | NAN | NAN | 1,54 |
| 5,1 | 5,3 | 4,35 | 7,80 | 2,21 |
| 5,3 | 5,0 | 1,75 | 6,11 | 1,98 |
| 4,6 | 5,2 | NAN | NAN | 0,90 |
| 5,2 | 5,7 | 2,39 | 6,86 | 2,05 |
| 4,9 | 5,2 | NAN | NAN | 0,68 |
| 5,0 | 5,5 | 2,54 | 6,58 | 3,12 |

| | | | | |
|---|---|---|---|---|
| 4,8 | NAN | 3,05 | 7,17 | 1,33 |
| 5,0 | 5,4 | 1,71 | 8,18 | 1,27 |
| 5,2 | 5,3 | NAN | NAN | 0,94 |
| 4,3 | 4,6 | 1,53 | 8,36 | 1,39 |
| 4,9 | 5,0 | 0,23 | 11,63 | 1,81 |
| 4,9 | 5,2 | 2,01 | 6,39 | 1,88 |
| 5,0 | 5,2 | 2,59 | 6,30 | 2,84 |
| 4,9 | 5,3 | 1,73 | 7,87 | 1,46 |
| 5,4 | 5,6 | NAN | NAN | 2,37 |
| 5,1 | 5,3 | 0,78 | 7,98 | 1,33 |
| 5,3 | 5,5 | 1,70 | 6,63 | 1,09 |
| 5,2 | 5,3 | NAN | NAN | 3,10 |
| 4,9 | 5,1 | 2,52 | 7,12 | 1,93 |
| 4,8 | 5,3 | 1,51 | 8,34 | 5,60 |
| 5,1 | 5,5 | 1,56 | 7,03 | 1,73 |
| 5,1 | 5,4 | 0,21 | 7,98 | 2,88 |
| 5,3 | 5,5 | NAN | NAN | 0,71 |
| 5,1 | 5,3 | NAN | NAN | 0,95 |
| 5,3 | 5,4 | NAN | NAN | 0,60 |
| 4,8 | 5,0 | NAN | NAN | 2,71 |
| 5,0 | 5,5 | 1,21 | 7,52 | 1,56 |
| 5,0 | 5,3 | NAN | NAN | 1,03 |
| 4,9 | 5,5 | 2,07 | 6,23 | 1,76 |
| 4,6 | 5,1 | 2,38 | 7,37 | 1,32 |
| 5,4 | 5,4 | 2,84 | 5,88 | 1,29 |
| 5,0 | 5,2 | 2,64 | 7,96 | 0,97 |
| 4,9 | 5,5 | 2,69 | 8,35 | 2,03 |
| 5,1 | 5,3 | 1,25 | 7,04 | 1,91 |
| 5,4 | 6,0 | 2,40 | 5,25 | 3,77 |
| 4,8 | 5,2 | 2,64 | 6,88 | 2,19 |
| 5,7 | 5,2 | 3,20 | 7,57 | 1,56 |
| 5,8 | 6,2 | 1,97 | 6,19 | 2,31 |
| 4,8 | 5,4 | 2,41 | 7,35 | 1,93 |
| 5,0 | 5,2 | 3,20 | 8,51 | 2,93 |
| 5,5 | 5,9 | NAN | NAN | 3,72 |
| 4,8 | 5,3 | 1,92 | 7,64 | 1,62 |
| 5,3 | 5,4 | 0,03 | 9,12 | 2,61 |
| 5,1 | 5,4 | 3,75 | 6,64 | 5,71 |
| 4,8 | 5,2 | 2,54 | 7,60 | 1,01 |
| 4,8 | 5,2 | 1,64 | 5,27 | 2,14 |
| 5,0 | 5,3 | 2,63 | 7,40 | 2,02 |
| 5,6 | 5,8 | NAN | NAN | 2,75 |
| 5,3 | 5,3 | 0,20 | 10,39 | 1,95 |
| 5,1 | 5,2 | NAN | NAN | 3,77 |
| 5,2 | 5,1 | 3,18 | 6,35 | NAN |
| 5,3 | 5,3 | 3,43 | 6,19 | 2,10 |
| 5,3 | 5,4 | 2,89 | 8,10 | 3,30 |
| 4,8 | 5,5 | 3,59 | 7,30 | 1,62 |
| 4,8 | 5,0 | NAN | NAN | 1,53 |
| 5,3 | 5,5 | NAN | NAN | 3,16 |

| | | | | |
|---|---|---|---|---|
| 5,4 | 5,2 | 0,39 | 7,41 | 0,55 |
| 5,5 | 5,3 | NAN | NAN | 1,72 |
| 5,2 | 5,7 | NAN | NAN | 1,20 |
| 5,1 | 5,2 | NAN | NAN | 1,82 |
| 5,4 | 5,8 | 1,84 | 6,32 | 1,29 |
| 4,6 | 4,7 | 0,10 | 8,71 | 0,43 |
| 4,6 | 5,1 | NAN | 7,17 | 3,13 |
| 5,2 | 5,5 | NAN | NAN | 1,99 |
| 5,2 | 5,4 | 1,81 | 5,93 | 0,93 |
| 5,0 | 5,5 | 1,96 | 7,17 | 5,45 |
| 4,8 | 5,3 | 0,73 | 9,15 | 3,15 |
| 4,2 | 4,6 | 3,22 | 6,67 | 2,33 |
| 5,3 | 5,6 | 3,44 | 6,40 | 2,21 |
| 5,0 | 5,0 | 2,20 | 5,44 | 2,10 |
| 4,4 | 5,2 | NAN | NAN | 2,30 |
| 5,3 | 5,6 | NAN | NAN | 2,91 |
| 5,2 | 5,4 | 1,08 | 6,10 | 1,18 |
| 4,8 | 5,1 | 2,86 | 5,10 | 2,47 |
| NAN | 5,4 | 3,19 | NAN | 1,59 |
| 4,4 | 5,1 | 3,35 | 6,74 | 4,04 |
| 4,9 | 5,4 | 0,22 | 10,04 | 3,62 |
| 5,5 | 5,5 | 0,70 | 7,07 | 1,11 |
| 4,9 | 5,2 | 0,84 | 7,28 | 1,15 |
| 5,8 | 6,0 | 2,35 | 6,27 | 1,37 |
| 5,4 | 5,6 | NAN | NAN | 1,78 |
| 5,1 | 5,9 | 2,46 | 7,19 | 1,53 |
| NAN | 5,8 | 3,49 | 7,66 | 1,21 |
| 4,9 | 5,4 | NAN | NAN | 2,19 |
| 5,1 | 5,6 | NAN | NAN | 0,76 |
| 5,2 | 5,7 | 2,90 | 5,52 | 1,09 |
| 4,6 | NAN | 1,67 | 8,62 | 1,97 |
| 5,4 | 5,4 | 1,35 | 7,66 | 0,44 |
| 5,1 | 5,5 | 2,68 | 8,05 | 1,99 |
| 4,6 | 5,1 | NAN | NAN | 1,36 |
| 5,1 | 5,2 | 1,67 | 7,60 | 1,09 |
| 4,7 | 5,4 | 1,84 | 6,71 | 1,60 |
| 4,6 | 4,9 | 2,05 | 5,91 | 1,69 |
| 4,9 | 5,1 | 2,64 | 6,69 | 1,52 |
| 4,8 | 5,1 | 2,42 | 6,58 | 1,66 |
| 5,1 | 5,2 | 1,73 | 7,37 | 1,72 |
| 5,1 | 5,5 | 2,49 | 7,26 | 2,15 |
| 4,8 | 5,2 | 2,73 | 6,66 | 2,82 |
| 5,7 | 5,8 | NAN | NAN | 0,97 |
| 5,4 | 5,8 | 1,47 | 7,97 | 1,62 |
| 4,7 | 4,8 | 1,39 | 6,38 | 1,36 |
| 4,9 | 5,4 | NAN | NAN | 1,66 |
| 4,7 | 5,0 | 1,50 | 6,99 | 0,65 |
| 5,0 | 5,6 | 1,06 | 6,02 | 1,20 |
| 5,2 | 5,6 | 3,86 | 7,16 | 1,25 |
| 5,1 | 5,5 | 1,47 | 8,80 | 1,40 |

| | | | | |
|---|---|---|---|---|
| 5,0 | 5,6 | NAN | NAN | 1,69 |
| 5,4 | 5,5 | 1,79 | 6,22 | 2,05 |
| 5,1 | 5,7 | NAN | NAN | 0,83 |
| 5,1 | 5,5 | 0,60 | 8,05 | 2,48 |
| 5,0 | 5,5 | 2,18 | 6,03 | 1,80 |
| 5,0 | 5,5 | 1,30 | 6,06 | 0,21 |
| 4,7 | 5,2 | NAN | NAN | 2,33 |
| 5,0 | 5,8 | 1,06 | 7,27 | 1,06 |
| 5,0 | 5,1 | 1,11 | 5,65 | 1,98 |
| 4,9 | 5,5 | 0,87 | 7,27 | 2,16 |
| 4,9 | 5,2 | 1,17 | 7,68 | 2,53 |
| 5,1 | 5,6 | 2,40 | 7,46 | 0,81 |
| 4,4 | 5,4 | 2,69 | 7,26 | 1,37 |
| 4,9 | 5,1 | 1,71 | 6,56 | 1,02 |
| 4,3 | 5,2 | 2,72 | 6,73 | NAN |
| 4,4 | 5,0 | NAN | NAN | 3,75 |
| 5,4 | 5,3 | 2,85 | 5,65 | 1,19 |
| 4,7 | 5,1 | 2,19 | 6,15 | 1,20 |
| 5,0 | 5,4 | NAN | NAN | 1,70 |
| 5,3 | 5,5 | NAN | NAN | 2,66 |
| NAN | 5,4 | 2,61 | 6,49 | 1,63 |
| 4,9 | 5,5 | NAN | NAN | 1,35 |
| 4,8 | 5,0 | 2,04 | 7,27 | 3,70 |
| 4,4 | NAN | 2,69 | 7,06 | 1,38 |
| 5,0 | 5,7 | 2,01 | 6,87 | 1,05 |
| 5,0 | 5,7 | 1,90 | 6,33 | 1,88 |
| 5,0 | 5,5 | NAN | NAN | 1,68 |
| 4,4 | 5,0 | 2,54 | 6,70 | 1,61 |
| 4,8 | 5,3 | 2,35 | 6,43 | 1,85 |
| 5,0 | 5,2 | 2,13 | 7,63 | 1,30 |
| 5,2 | 5,5 | 0,36 | 6,59 | 0,58 |
| 4,6 | 5,3 | 2,43 | 7,63 | 0,75 |
| 5,1 | 5,5 | 2,03 | 6,57 | 1,11 |
| 5,2 | 5,7 | NAN | NAN | 1,42 |
| 4,9 | 4,6 | NAN | NAN | 1,33 |
| 5,1 | 5,7 | 2,06 | 8,01 | 1,56 |
| 4,4 | 5,0 | 1,45 | 8,58 | 1,15 |
| 5,2 | 5,4 | 1,73 | 6,89 | 1,39 |
| 4,9 | 5,4 | 2,04 | 6,27 | 1,58 |
| 4,9 | 5,3 | 2,88 | 6,24 | 2,69 |
| 5,0 | 5,2 | NAN | NAN | 2,34 |
| 5,2 | 5,3 | 2,92 | 6,49 | 1,30 |
| 5,1 | 5,7 | 4,43 | 5,99 | 2,32 |
| 5,3 | 5,3 | 2,54 | 6,24 | 2,15 |
| 5,1 | 5,8 | NAN | NAN | 1,24 |
| 4,9 | 5,0 | NAN | NAN | 4,08 |
| 5,0 | 5,2 | NAN | NAN | 1,93 |
| 4,8 | 5,4 | 0,46 | 7,57 | 2,77 |
| 5,0 | 5,5 | 0,04 | 9,69 | 3,98 |
| 5,7 | 5,8 | 2,31 | 6,95 | 1,42 |

| | | | | |
|---|---|---|---|---|
| 4,9 | 5,4 | NAN | NAN | 2,44 |
| 4,1 | 4,0 | NAN | NAN | 1,81 |
| 5,3 | 6,2 | NAN | NAN | 2,87 |
| 4,5 | 5,1 | NAN | NAN | 2,98 |
| 4,9 | 5,2 | NAN | NAN | 1,36 |
| 4,8 | 5,1 | 2,12 | 6,95 | 1,18 |
| 4,3 | 4,9 | 1,32 | 6,22 | 0,74 |
| 5,1 | 5,4 | 1,70 | 8,36 | 0,55 |
| 4,9 | 5,4 | 1,52 | 7,73 | 1,68 |
| NAN | 5,4 | 0,46 | 8,65 | 0,31 |
| 4,7 | 5,2 | NAN | NAN | 3,83 |
| 5,1 | 5,5 | NAN | NAN | 2,72 |
| 4,9 | 5,4 | 1,68 | 7,15 | 1,80 |
| 5,2 | 5,6 | 2,70 | 7,42 | 3,16 |
| NAN | 5,5 | NAN | NAN | 0,67 |
| 4,9 | 5,2 | 3,67 | 5,20 | 2,77 |
| 5,0 | 5,6 | 1,93 | 5,80 | 1,85 |
| 5,2 | 5,8 | NAN | NAN | 3,42 |
| 4,8 | 5,3 | 2,01 | 6,94 | 2,04 |
| 4,7 | 5,2 | NAN | NAN | 2,33 |
| 5,1 | 5,4 | NAN | NAN | 2,27 |
| 4,5 | 4,9 | 1,21 | 7,37 | 1,25 |
| 4,9 | 5,4 | 4,49 | 6,26 | 2,38 |
| 4,9 | 5,4 | NAN | NAN | 2,43 |
| 5,0 | 5,5 | 1,83 | 6,09 | 2,05 |
| 4,1 | 5,1 | 2,07 | 6,31 | 1,36 |
| 5,0 | 5,6 | 1,88 | 7,71 | 2,34 |
| 5,0 | 5,4 | 2,43 | 6,44 | 0,78 |
| 4,4 | 5,2 | NAN | NAN | 1,06 |
| 5,2 | 5,5 | 1,76 | 5,81 | 1,89 |
| 4,8 | 5,2 | 2,17 | 7,06 | 2,32 |
| 5,2 | 5,9 | 1,84 | 7,69 | 2,87 |
| 4,9 | 5,4 | NAN | NAN | 0,69 |
| 4,8 | 5,1 | 1,21 | 10,81 | 2,31 |
| 4,7 | 5,2 | 2,40 | 6,74 | 3,00 |
| 4,7 | 5,0 | 2,20 | 5,43 | 1,67 |
| 5,1 | 5,1 | NAN | NAN | 1,28 |
| 5,0 | 5,6 | 1,44 | 7,89 | 2,02 |
| 4,4 | 4,7 | 0,56 | 10,99 | 1,22 |
| 5,0 | 5,3 | 1,88 | 6,81 | 2,24 |
| 5,2 | 5,5 | 1,98 | 6,59 | 2,54 |
| 5,2 | 5,9 | NAN | NAN | 1,43 |
| 4,9 | 5,0 | 3,16 | 7,50 | 1,69 |
| 5,0 | 5,5 | 1,85 | 5,20 | 1,68 |
| 5,0 | 4,1 | NAN | NAN | 2,15 |
| 5,0 | 5,2 | 1,51 | 7,40 | 3,77 |
| NAN | 5,5 | 1,95 | 8,21 | 2,03 |
| 5,1 | 5,4 | 1,83 | 7,35 | 1,99 |
| 4,9 | 5,6 | 1,00 | 7,24 | 1,48 |
| 5,1 | 5,6 | 2,01 | 6,58 | 1,66 |

| | | | | |
|---|---|---|---|---|
| 5,2 | 5,7 | 1,48 | 6,59 | 0,89 |
| 5,4 | 5,7 | 1,78 | 7,13 | 1,05 |
| 5,1 | 5,2 | 3,99 | 5,27 | 3,22 |
| 5,2 | 5,9 | 2,34 | 5,31 | 1,72 |
| 5,6 | 5,9 | NAN | NAN | 1,05 |
| 4,7 | 5,2 | NAN | NAN | 0,90 |
| 4,4 | 4,9 | 0,63 | 6,66 | 1,03 |
| 5,0 | 5,3 | 0,90 | 6,95 | 1,73 |
| 5,1 | 5,7 | 1,30 | 6,79 | 2,61 |
| 5,1 | 5,7 | 1,33 | 5,47 | 0,69 |
| 5,2 | 5,9 | NAN | NAN | 1,56 |
| 4,9 | 5,4 | 2,14 | 6,96 | 1,45 |
| 4,6 | 5,2 | 3,70 | 7,09 | 2,67 |
| 4,6 | 5,3 | 1,89 | 7,48 | 3,13 |
| 5,2 | 5,4 | 2,35 | 7,59 | 1,67 |
| 5,0 | 5,5 | 3,26 | 5,70 | 1,91 |

| FT4 (ng/dl) 2 Yrs PD | FP Insulin (mcU/ml) 24 gw | FP Insulin (mcU/ml) 2 years PD | Insulin Increase from baseline 24 GW |
|---|---|---|---|
| 13,52 | 6,55 | 5,93 | -5,35 |
| 8,96 | 7,24 | 8,55 | -4,36 |
| 8,28 | 6,27 | 10,39 | -3,87 |
| 8,49 | 16,39 | 11,68 | -12,91 |
| 8,36 | 13,39 | 13,23 | -0,67 |
| 10,23 | 19,81 | 9,82 | 22,95 |
| 7,66 | 5,69 | 5,11 | -4,49 |
| 7,76 | 7,69 | 5,75 | -4,89 |
| 9,37 | 8,88 | 6,41 | -2,24 |
| 8,60 | 12,90 | 24,65 | -3,34 |
| 7,89 | 7,10 | 6,07 | -4,34 |
| 7,30 | 9,73 | 37,05 | -1,49 |
| 8,79 | 5,31 | 3,49 | -4,11 |
| NAN | 12,81 | 8,30 | -1,21 |
| 7,46 | 8,11 | 6,56 | -5,03 |
| 9,15 | 8,10 | 7,72 | -6,50 |
| 9,25 | 9,66 | 5,99 | -4,54 |
| 9,08 | 15,03 | 42,89 | -9,87 |
| 10,09 | 7,05 | 7,48 | -5,69 |
| 7,38 | 11,79 | 13,16 | -7,51 |
| 7,91 | 10,77 | 8,37 | -8,37 |
| 6,90 | 16,06 | 16,53 | -12,90 |
| 8,08 | 19,80 | 20,83 | -16,64 |
| 6,62 | 4,96 | 8,00 | -3,36 |
| 7,11 | 16,48 | 16,51 | -13,04 |
| 7,00 | 10,61 | 14,21 | -9,41 |
| 6,56 | 10,02 | 9,48 | -8,66 |
| 7,31 | 10,24 | 140,69 | -5,88 |
| 9,62 | 17,61 | 13,28 | -14,17 |
| 8,51 | 21,48 | 9,84 | -17,08 |
| 10,05 | 12,05 | 13,64 | -8,61 |
| 8,32 | 6,26 | 9,56 | -3,86 |
| 7,90 | 18,33 | 7,82 | -16,97 |
| 8,52 | 12,84 | 14,88 | -10,40 |
| 7,33 | 6,80 | 7,68 | -0,04 |
| 8,14 | 10,76 | 30,32 | -6,00 |
| 9,36 | 5,75 | 8,22 | -4,55 |
| 11,22 | 14,20 | 15,63 | -7,92 |
| 7,84 | 18,36 | 19,63 | -14,12 |
| 8,65 | 9,22 | 8,78 | 16,78 |
| 8,61 | 13,86 | 8,33 | -9,10 |
| 6,92 | 17,08 | 13,14 | -13,92 |
| 7,20 | 11,96 | 11,99 | -8,80 |
| 9,60 | 7,57 | 5,89 | -0,29 |
| 9,16 | 10,29 | 10,37 | -8,49 |
| 8,75 | 9,36 | 5,23 | -0,68 |
| 8,00 | 11,55 | 6,90 | -6,75 |

| | | | |
|---|---|---|---|
| 6,93 | 7,37 | 4,48 | -5,93 |
| 8,64 | 11,41 | 49,17 | 1,47 |
| 7,58 | 16,44 | 10,79 | -11,84 |
| 7,28 | 12,82 | 16,59 | -7,58 |
| 10,59 | 8,06 | 13,96 | -5,02 |
| 8,03 | 26,36 | 14,40 | -24,32 |
| 8,97 | 13,24 | 45,36 | -2,28 |
| NAN | NAN | 6,81 | NAN |
| 9,49 | 15,87 | 10,28 | -11,75 |
| 9,36 | 5,77 | 5,51 | 11,23 |
| 10,53 | 20,91 | 9,13 | 3,85 |
| 8,98 | 10,69 | 9,04 | 6,11 |
| 6,89 | 15,91 | 9,77 | -13,07 |
| 9,54 | 9,25 | 7,30 | -2,49 |
| 9,02 | 27,75 | 11,33 | 14,37 |
| 8,74 | NAN | 49,31 | NAN |
| 8,63 | 13,40 | 10,80 | -11,40 |
| 11,82 | 10,46 | 6,79 | 5,22 |
| 7,40 | 12,96 | 10,96 | 3,84 |
| 9,43 | 12,43 | 9,83 | -9,47 |
| 8,45 | 8,48 | 10,26 | -0,64 |
| 8,63 | 11,64 | 7,64 | -9,56 |
| 7,56 | NAN | 113,81 | NAN |
| 9,10 | 7,26 | 5,53 | -6,02 |
| 9,27 | 18,28 | 48,49 | 9,08 |
| 9,43 | 53,36 | 7,00 | -52,00 |
| 8,27 | 11,30 | 8,26 | 3,10 |
| 8,29 | 15,90 | 17,88 | -9,58 |
| 7,73 | 10,38 | 11,02 | -7,10 |
| 7,86 | 8,76 | 7,65 | -7,20 |
| 8,06 | 8,03 | 10,90 | -5,59 |
| 7,84 | 7,03 | 8,56 | -5,83 |
| 7,45 | 14,87 | 12,47 | -12,51 |
| 9,28 | 9,25 | 6,36 | -7,45 |
| 8,70 | 8,88 | 8,51 | -7,68 |
| 7,78 | 19,44 | 11,46 | -5,76 |
| 8,32 | 10,06 | 10,42 | -2,98 |
| 8,23 | 10,14 | 17,22 | -6,10 |
| 8,27 | 10,83 | 13,95 | -3,27 |
| 9,82 | 9,88 | 8,77 | -8,68 |
| 9,12 | 8,98 | 5,68 | 6,38 |
| 8,90 | 21,55 | 14,28 | -17,55 |
| 8,29 | 14,45 | 14,34 | -8,13 |
| 7,13 | 6,59 | 5,40 | -5,39 |
| 9,43 | 7,03 | 7,30 | -4,87 |
| 7,54 | 10,80 | 9,84 | 3,76 |
| 7,68 | 54,80 | 7,68 | -53,60 |
| 8,97 | 10,66 | 3,77 | -8,58 |
| 8,14 | 17,03 | 10,66 | -4,19 |
| 8,08 | 6,03 | 6,48 | -2,99 |

| | | | |
|---|---|---|---|
| 9,18 | 8,64 | 6,17 | -7,28 |
| 6,96 | 13,83 | 13,73 | -10,99 |
| 8,03 | 6,16 | 5,87 | 5,56 |
| 8,25 | 86,55 | 31,00 | -82,87 |
| 8,37 | 16,52 | 8,50 | -3,52 |
| 8,18 | 14,19 | 33,43 | -12,91 |
| 8,57 | 13,36 | 13,93 | -5,48 |
| 8,37 | 8,34 | 7,78 | 6,22 |
| 5,99 | 14,29 | 11,85 | 7,03 |
| 8,17 | 6,27 | NAN | 0,77 |
| 6,90 | 10,54 | 10,91 | -8,22 |
| 7,71 | 8,15 | 12,83 | -5,03 |
| 7,97 | 5,00 | 5,29 | -3,80 |
| 7,97 | 7,06 | 23,81 | -5,06 |
| 8,32 | 18,89 | 46,56 | -14,57 |
| 7,51 | 18,81 | 9,32 | -16,29 |
| 8,89 | 8,54 | NAN | -5,02 |
| 9,44 | 7,14 | 3,98 | -1,82 |
| 8,10 | 10,74 | 15,08 | -9,06 |
| 9,44 | 12,88 | 24,99 | -11,08 |
| 6,86 | 5,21 | 2,78 | 5,39 |
| 8,27 | 9,51 | 10,40 | -0,59 |
| 9,07 | 16,36 | 11,97 | 17,72 |
| 6,70 | 11,15 | 6,68 | -9,23 |
| 8,25 | 21,64 | 11,58 | -17,52 |
| 9,99 | 8,52 | 17,01 | -6,16 |
| 8,82 | 12,33 | 8,90 | 14,83 |
| 9,09 | 12,28 | 7,18 | -10,36 |
| 5,87 | 18,63 | 69,61 | -10,83 |
| 8,28 | 8,12 | 7,85 | -2,96 |
| 7,35 | 83,45 | 15,66 | -74,45 |
| 7,62 | 6,07 | 4,15 | NAN |
| 8,41 | 4,35 | 7,35 | -3,15 |
| 8,16 | 10,43 | 10,93 | -7,47 |
| 6,51 | 21,29 | 132,12 | 12,75 |
| 10,10 | 16,09 | 7,84 | -4,29 |
| 3,41 | 23,00 | 52,51 | -20,44 |
| 8,95 | 8,35 | 30,05 | -5,95 |
| 10,41 | 65,14 | 13,02 | -62,66 |
| 7,27 | 11,94 | 9,38 | -8,82 |
| 9,31 | 137,27 | 7,63 | -135,87 |
| 7,15 | 19,38 | 20,32 | -15,94 |
| 10,90 | 16,98 | 10,22 | 6,58 |
| 9,17 | 10,79 | 43,04 | -9,59 |
| NAN | 14,90 | 8,21 | 4,18 |
| 8,90 | 7,16 | 5,15 | -5,96 |
| 9,78 | 7,97 | 7,75 | -6,77 |
| 6,93 | 11,79 | 7,07 | -10,23 |
| 8,27 | 9,45 | 10,04 | -6,25 |
| 5,81 | 14,23 | 8,83 | -10,03 |

| | | | |
|---|---|---|---|
| 9,87 | 7,95 | 8,79 | -2,35 |
| 9,52 | 17,68 | 7,70 | -11,28 |
| 9,79 | 10,05 | 5,65 | -8,61 |
| 7,94 | 14,73 | 12,11 | -6,49 |
| 8,28 | 8,10 | 4,36 | 0,34 |
| 8,78 | 8,06 | 6,52 | -5,54 |
| 7,90 | 11,39 | 5,78 | 16,13 |
| 10,03 | 10,56 | 9,21 | -3,92 |
| 9,35 | 12,98 | 7,94 | -2,86 |
| 8,85 | 4,19 | 5,93 | -2,79 |
| 7,73 | 4,23 | 9999,00 | -3,03 |
| 7,95 | 8,60 | 5,89 | 0,68 |
| 7,83 | 7,98 | 6,58 | -6,22 |
| 7,04 | 7,46 | 5,89 | 0,22 |
| 8,51 | 9,12 | 10,07 | -7,24 |
| 9,09 | 18,87 | 10,35 | -14,35 |
| 7,59 | 11,16 | 41,86 | -7,84 |
| 7,17 | 8,68 | 8,72 | -7,08 |
| 7,26 | 19,99 | 12,29 | -5,75 |
| 6,76 | 6,37 | 7,92 | -4,61 |
| 8,02 | 12,59 | 56,04 | -3,31 |
| 9,48 | 5,41 | 7,90 | 14,59 |
| 8,10 | 23,85 | 19,54 | -17,73 |
| 9,53 | 8,45 | 6,37 | 11,95 |
| 6,70 | 15,67 | 28,77 | -14,03 |
| 7,40 | 10,51 | 7,83 | -8,99 |
| 8,80 | 6,18 | 4,59 | 2,98 |
| 8,29 | 72,85 | 13,20 | -70,25 |
| 7,81 | 14,35 | 10,25 | -10,47 |
| 7,65 | 4,55 | 3,44 | 2,97 |
| 8,46 | 7,70 | 5,03 | -6,50 |
| 8,29 | 8,65 | 9,61 | -4,69 |
| 9,91 | 10,48 | 6,71 | -8,16 |
| 6,90 | 6,78 | 11,34 | -4,58 |
| 9,33 | 12,01 | 69,33 | 2,31 |
| 7,80 | 10,35 | 9,02 | -7,95 |
| 8,37 | 14,39 | 14,91 | -12,35 |
| 8,92 | 8,08 | 8,79 | -5,00 |
| 8,57 | 10,29 | 9,04 | 12,47 |
| 6,58 | 8,34 | 14,13 | -4,94 |
| 9,10 | 8,69 | 8,83 | 7,83 |
| 7,60 | 8,67 | 9,94 | -6,95 |
| 7,32 | 5,85 | 5,87 | -4,57 |
| 11,84 | 7,15 | 4,15 | -2,87 |
| 7,75 | 11,12 | 12,19 | 8,92 |
| 8,47 | 11,65 | 8,61 | 14,59 |
| 8,76 | 6,52 | 5,15 | -4,08 |
| 9,80 | 10,14 | 7,49 | -8,46 |
| 7,26 | 6,42 | 4,89 | 1,54 |
| 8,57 | 8,53 | 8,20 | -1,25 |

| | | | |
|---|---|---|---|
| 8,10 | 13,71 | 39,22 | -10,67 |
| 7,91 | 9,21 | 9,70 | 6,35 |
| 7,49 | 7,19 | 6,92 | -1,35 |
| 8,41 | 12,48 | 13,39 | -8,44 |
| 8,14 | 5,27 | 7,46 | 4,69 |
| 7,45 | 11,40 | 14,49 | -9,12 |
| 7,59 | 8,44 | 5,43 | -7,24 |
| 8,03 | 9,16 | 10,55 | -5,80 |
| 7,91 | 5,39 | 16,36 | -3,03 |
| 7,08 | 8,70 | 7,97 | -6,22 |
| 8,16 | 6,56 | 5,75 | -4,36 |
| 8,36 | 9,14 | 8,70 | -4,50 |
| 7,89 | 4,90 | 2,68 | -1,54 |
| 8,63 | 16,79 | 17,61 | -11,43 |
| 7,81 | 52,08 | 6,18 | -30,40 |
| 7,35 | 13,77 | 10,51 | -12,49 |
| 6,72 | 11,61 | 9,85 | -2,33 |
| 7,73 | 9,16 | 5,55 | -7,12 |
| 7,39 | 5,13 | 8,62 | 1,51 |
| 7,64 | 27,07 | 6,67 | -22,23 |
| 8,70 | 15,32 | 7,64 | -12,44 |
| 7,33 | 12,11 | 10,55 | 4,33 |
| 6,54 | 7,58 | 7,28 | -6,30 |
| 7,93 | 9,74 | 6,13 | 12,10 |
| 6,93 | 5,79 | 10,42 | -2,35 |
| 5,48 | 12,44 | 20,72 | -9,64 |
| 6,47 | 9,42 | 7,91 | -6,98 |
| 6,81 | 7,41 | 10,98 | -4,61 |
| 8,60 | 10,00 | 8,07 | -8,32 |
| 9,36 | 10,08 | 6,23 | -2,36 |
| 7,35 | 19,21 | 9,40 | -18,01 |
| 8,34 | 7,72 | 6,76 | -5,88 |
| 8,81 | 8,61 | 12,42 | -6,69 |
| 9,30 | 8,54 | 11,26 | -1,22 |
| 8,36 | 9,66 | 8,61 | 3,98 |
| 9,91 | 14,52 | 14,58 | -11,52 |
| 7,66 | 7,89 | 4,34 | -5,57 |
| 7,74 | 6,66 | 5,76 | 6,98 |
| 7,14 | 6,66 | 16,10 | -2,26 |
| 6,03 | 9,54 | 12,93 | -5,26 |
| 7,30 | 32,55 | 7,90 | -30,51 |
| 7,68 | 10,30 | 6,86 | -8,46 |
| 8,10 | 8,08 | 8,77 | -6,48 |
| 7,92 | 12,88 | 7,43 | -9,72 |
| 6,77 | 4,53 | 4,41 | -2,93 |
| 8,83 | 4,21 | 6,69 | -2,89 |
| 8,22 | 11,93 | 7,30 | -10,21 |
| 6,76 | 13,56 | 12,81 | -4,32 |
| 8,05 | 7,03 | 3,99 | 4,77 |
| 10,32 | 8,71 | 9,48 | -4,15 |

| | | | |
|---|---|---|---|
| 8,39 | 10,80 | 8,04 | -9,04 |
| 7,38 | 9,45 | 6,42 | -2,45 |
| 8,36 | 16,54 | 30,10 | -13,02 |
| 7,74 | 14,00 | 17,21 | -4,20 |
| 8,38 | 11,53 | 14,42 | -6,69 |
| 7,42 | 7,24 | 6,23 | 3,80 |
| 9,27 | 7,18 | 7,52 | -5,06 |
| 12,11 | 7,22 | 23,56 | 7,26 |
| 8,85 | 6,02 | 6,99 | -1,02 |
| 10,21 | 7,95 | 8,79 | -6,59 |
| 7,67 | NAN | 8,37 | NAN |
| 7,62 | 6,73 | 10,59 | -5,01 |
| 8,10 | 9,05 | 8,79 | -7,73 |
| 8,48 | 28,47 | 6,91 | -13,31 |
| 6,06 | 19,28 | 13,65 | -14,64 |
| 6,94 | 24,90 | 20,50 | -19,42 |
| 8,94 | 5,05 | 9,90 | -1,25 |
| 6,51 | 8,22 | 6,09 | 0,94 |
| 7,17 | 4,60 | 6,55 | -3,24 |
| 9,06 | 13,77 | 8,41 | -0,77 |
| 7,37 | 7,81 | 5,68 | -6,53 |
| 10,17 | 11,34 | 8,71 | -7,46 |
| 7,87 | 12,04 | 25,09 | -10,56 |
| 8,63 | 14,63 | 9,34 | -7,99 |
| 9,07 | 14,88 | 12,24 | -5,20 |
| 8,89 | 8,58 | 5,93 | -3,18 |
| 7,28 | 15,14 | 131,23 | -9,78 |
| 7,18 | 6,74 | 8,58 | -3,78 |
| 7,71 | 31,05 | 4,68 | -29,53 |
| 8,77 | 14,38 | 9,90 | -12,14 |
| 6,51 | 8,91 | 7,19 | 7,25 |
| 7,79 | 9,14 | 19,48 | -6,26 |
| 6,99 | 10,31 | 8,80 | -8,55 |
| 9,39 | 9,03 | 8,56 | -3,39 |
| 7,23 | 6,76 | 77,28 | 3,72 |
| 7,76 | 8,06 | 6,77 | -4,38 |
| 8,54 | 4,94 | 9,92 | -3,74 |
| 9,14 | 5,13 | 6,47 | 3,07 |
| 10,42 | 6,96 | 9,21 | 5,24 |
| 5,94 | 3,62 | 7,80 | -1,78 |
| 7,45 | NAN | 6,52 | NAN |
| 8,69 | 9,80 | 38,37 | 9,00 |
| 7,51 | 45,75 | 9,20 | -37,27 |
| 8,89 | 9,44 | 8,83 | -7,12 |
| 8,51 | 4,66 | 11,46 | 3,46 |
| 9,10 | 6,58 | 7,21 | -5,22 |
| 7,68 | 4,95 | 11,09 | -2,39 |
| 8,56 | 6,28 | 7,34 | -3,96 |
| 9,13 | 6,30 | 6,54 | -1,22 |
| 8,38 | 10,05 | 30,88 | 19,99 |

| | | | |
|---|---|---|---|
| 7,95 | 10,08 | 11,74 | 15,28 |
| 8,12 | 7,84 | 12,78 | -4,52 |
| 5,85 | 13,84 | 19,19 | 0,08 |
| 7,40 | 5,21 | 6,77 | 8,11 |
| 7,10 | 8,17 | 10,63 | 0,63 |
| 7,85 | 4,11 | 5,56 | -0,83 |
| 6,37 | 10,99 | 18,00 | -7,19 |
| 7,11 | 4,00 | 11,35 | -1,64 |
| 6,86 | 5,01 | 6,51 | -2,93 |
| 8,10 | 11,88 | 28,99 | 4,12 |
| 6,62 | 10,12 | 10,14 | -4,12 |
| 6,61 | 3,38 | 8,67 | -1,02 |
| 8,76 | 4,51 | 9999,00 | -1,03 |
| 7,37 | 8,41 | 11,07 | -4,13 |
| 8,66 | 7,11 | 5,79 | -5,31 |
| 7,14 | 4,40 | 15,39 | -2,36 |

| Insulin Increase from baseline 2 yrs PD | Insulin change from baseline 24 GW |
|---|---|
| -4,73 | -4,46 |
| -5,67 | -1,51 |
| -7,99 | -1,61 |
| -8,20 | -3,71 |
| -0,51 | -0,05 |
| 32,94 | 0,54 |
| -3,91 | -3,74 |
| -2,95 | -1,75 |
| 0,23 | -0,34 |
| -15,09 | -0,35 |
| -3,31 | -1,57 |
| -28,81 | -0,18 |
| -2,29 | -3,43 |
| 3,30 | -0,10 |
| -3,48 | -1,63 |
| -6,12 | -4,06 |
| -0,87 | -0,89 |
| -37,73 | -1,91 |
| -6,12 | -4,18 |
| -8,88 | -1,75 |
| -5,97 | -3,49 |
| -13,37 | -4,08 |
| -17,67 | -5,27 |
| -6,40 | -2,10 |
| -13,07 | -3,79 |
| -13,01 | -7,84 |
| -8,12 | -6,37 |
| -136,33 | -1,35 |
| -9,84 | -4,12 |
| -5,44 | -3,88 |
| -10,20 | -2,50 |
| -7,16 | -1,61 |
| -6,46 | -12,48 |
| -12,44 | -4,26 |
| -0,92 | -0,01 |
| -25,56 | -1,26 |
| -7,02 | -3,79 |
| -9,35 | -1,26 |
| -15,39 | -3,33 |
| 17,22 | 0,65 |
| -3,57 | -1,91 |
| -9,98 | -4,41 |
| -8,83 | -2,78 |
| 1,39 | -0,04 |
| -8,57 | -4,72 |
| 3,45 | -0,08 |
| -2,10 | -1,41 |

| | |
|---|---|
| -3,04 | -4,12 |
| -36,29 | 0,11 |
| -6,19 | -2,57 |
| -11,35 | -1,45 |
| -10,92 | -1,65 |
| -12,36 | -11,92 |
| -34,40 | -0,21 |
| -4,37 | NAN |
| -6,16 | -2,85 |
| 11,49 | 0,66 |
| 15,63 | 0,16 |
| 7,76 | 0,36 |
| -6,93 | -4,60 |
| -0,54 | -0,37 |
| 30,79 | 0,34 |
| -18,19 | NAN |
| -8,80 | -5,70 |
| 8,89 | 0,33 |
| 5,84 | 0,23 |
| -6,87 | -3,20 |
| -2,42 | -0,08 |
| -5,56 | -4,60 |
| -111,09 | NAN |
| -4,29 | -4,85 |
| -21,13 | 0,33 |
| -5,64 | -38,24 |
| 6,14 | 0,22 |
| -11,56 | -1,52 |
| -7,74 | -2,16 |
| -6,09 | -4,62 |
| -8,46 | -2,29 |
| -7,36 | -4,86 |
| -10,11 | -5,30 |
| -4,56 | -4,14 |
| -7,31 | -6,40 |
| 2,22 | -0,42 |
| -3,34 | -0,42 |
| -13,18 | -1,51 |
| -6,39 | -0,43 |
| -7,57 | -7,23 |
| 9,68 | 0,42 |
| -10,28 | -4,39 |
| -8,02 | -1,29 |
| -4,20 | -4,49 |
| -5,14 | -2,25 |
| 4,72 | 0,26 |
| -6,48 | -44,67 |
| -1,69 | -4,13 |
| 2,18 | -0,33 |
| -3,44 | -0,98 |

| | |
|---|---|
| -4,81 | -5,35 |
| -10,89 | -3,87 |
| 5,85 | 0,47 |
| -27,32 | -22,52 |
| 4,50 | -0,27 |
| -32,15 | -10,09 |
| -6,05 | -0,70 |
| 6,78 | 0,43 |
| 9,47 | 0,33 |
| NAN | 0,11 |
| -8,59 | -3,54 |
| -9,71 | -1,61 |
| -4,09 | -3,17 |
| -21,81 | -2,53 |
| -42,24 | -3,37 |
| -6,80 | -6,46 |
| NAN | -1,43 |
| 1,34 | -0,34 |
| -13,40 | -5,39 |
| -23,19 | -6,16 |
| 7,82 | 0,51 |
| -1,48 | -0,07 |
| 22,11 | 0,52 |
| -4,76 | -4,81 |
| -7,46 | -4,25 |
| -14,65 | -2,61 |
| 18,26 | 0,55 |
| -5,26 | -5,40 |
| -61,81 | -1,39 |
| -2,69 | -0,57 |
| -6,66 | -8,27 |
| NAN | NAN |
| -6,15 | -2,63 |
| -7,97 | -2,52 |
| -98,08 | 0,37 |
| 3,96 | -0,36 |
| -49,95 | -7,98 |
| -27,65 | -2,48 |
| -10,54 | -25,27 |
| -6,26 | -2,83 |
| -6,23 | -97,05 |
| -16,88 | -4,63 |
| 13,34 | 0,28 |
| -41,84 | -7,99 |
| 10,87 | 0,22 |
| -3,95 | -4,97 |
| -6,55 | -5,64 |
| -5,51 | -6,56 |
| -6,84 | -1,95 |
| -4,63 | -2,39 |

| | |
|---|---|
| -3,19 | -0,42 |
| -1,30 | -1,76 |
| -4,21 | -5,98 |
| -3,87 | -0,79 |
| 4,08 | 0,04 |
| -4,00 | -2,20 |
| 21,74 | 0,59 |
| -2,57 | -0,59 |
| 2,18 | -0,28 |
| -4,53 | -1,99 |
| NAN | -2,53 |
| 3,39 | 0,07 |
| -4,82 | -3,53 |
| 1,79 | 0,03 |
| -8,19 | -3,85 |
| -5,83 | -3,17 |
| -38,54 | -2,36 |
| -7,12 | -4,43 |
| 1,95 | -0,40 |
| -6,16 | -2,62 |
| -46,76 | -0,36 |
| 12,10 | 0,73 |
| -13,42 | -2,90 |
| 14,03 | 0,59 |
| -27,13 | -8,55 |
| -6,31 | -5,91 |
| 4,57 | 0,33 |
| -10,60 | -27,02 |
| -6,37 | -2,70 |
| 4,08 | 0,39 |
| -3,83 | -5,42 |
| -5,65 | -1,18 |
| -4,39 | -3,52 |
| -9,14 | -2,08 |
| -55,01 | 0,16 |
| -6,62 | -3,31 |
| -12,87 | -6,05 |
| -5,71 | -1,62 |
| 13,72 | 0,55 |
| -10,73 | -1,45 |
| 7,69 | 0,47 |
| -8,22 | -4,04 |
| -4,59 | -3,57 |
| 0,13 | -0,67 |
| 7,85 | 0,45 |
| 17,63 | 0,56 |
| -2,71 | -1,67 |
| -5,81 | -5,04 |
| 3,07 | 0,19 |
| -0,92 | -0,17 |

| | |
|---|---|
| -36,18 | -3,51 |
| 5,86 | 0,41 |
| -1,08 | -0,23 |
| -9,35 | -2,09 |
| 2,50 | 0,47 |
| -12,21 | -4,00 |
| -4,23 | -6,03 |
| -7,19 | -1,73 |
| -14,00 | -1,28 |
| -5,49 | -2,51 |
| -3,55 | -1,98 |
| -4,06 | -0,97 |
| 0,68 | -0,46 |
| -12,25 | -2,13 |
| 15,50 | -1,40 |
| -9,23 | -9,76 |
| -0,57 | -0,25 |
| -3,51 | -3,49 |
| -1,98 | 0,23 |
| -1,83 | -4,59 |
| -4,76 | -4,32 |
| 5,89 | 0,26 |
| -6,00 | -4,92 |
| 15,71 | 0,55 |
| -6,98 | -0,68 |
| -17,92 | -3,44 |
| -5,47 | -2,86 |
| -8,18 | -1,65 |
| -6,39 | -4,95 |
| 1,49 | -0,31 |
| -8,20 | -15,01 |
| -4,92 | -3,20 |
| -10,50 | -3,48 |
| -3,94 | -0,17 |
| 5,03 | 0,29 |
| -11,58 | -3,84 |
| -2,02 | -2,40 |
| 7,88 | 0,51 |
| -11,70 | -0,51 |
| -8,65 | -1,23 |
| -5,86 | -14,96 |
| -5,02 | -4,60 |
| -7,17 | -4,05 |
| -4,27 | -3,08 |
| -2,81 | -1,83 |
| -5,37 | -2,19 |
| -5,58 | -5,94 |
| -3,57 | -0,47 |
| 7,81 | 0,40 |
| -4,92 | -0,91 |

| | |
|---|---|
| -6,28 | -5,14 |
| 0,58 | -0,35 |
| -26,58 | -3,70 |
| -7,41 | -0,43 |
| -9,58 | -1,38 |
| 4,81 | 0,34 |
| -5,40 | -2,39 |
| -9,08 | 0,50 |
| -1,99 | -0,20 |
| -7,43 | -4,85 |
| -5,57 | NAN |
| -8,87 | -2,91 |
| -7,47 | -5,86 |
| 8,25 | -0,88 |
| -9,01 | -3,16 |
| -15,02 | -3,54 |
| -6,10 | -0,33 |
| 3,07 | 0,10 |
| -5,19 | -2,38 |
| 4,59 | -0,06 |
| -4,40 | -5,10 |
| -4,83 | -1,92 |
| -23,61 | -7,14 |
| -2,70 | -1,20 |
| -2,56 | -0,54 |
| -0,53 | -0,59 |
| -125,87 | -1,82 |
| -5,62 | -1,28 |
| -3,16 | -19,43 |
| -7,66 | -5,42 |
| 8,97 | 0,45 |
| -16,60 | -2,17 |
| -7,04 | -4,86 |
| -2,92 | -0,60 |
| -66,80 | 0,35 |
| -3,09 | -1,19 |
| -8,72 | -3,12 |
| 1,73 | 0,37 |
| 2,99 | 0,43 |
| -5,96 | -0,97 |
| -4,40 | NAN |
| -19,57 | 0,48 |
| -0,72 | -4,40 |
| -6,51 | -3,07 |
| -3,34 | 0,43 |
| -5,85 | -3,84 |
| -8,53 | -0,93 |
| -5,02 | -1,71 |
| -1,46 | -0,24 |
| -0,84 | 0,67 |

| | |
|---|---|
| 13,62 | 0,60 |
| -9,46 | -1,36 |
| -5,27 | 0,01 |
| 6,55 | 0,61 |
| -1,83 | 0,07 |
| -2,28 | -0,25 |
| -14,20 | -1,89 |
| -8,99 | -0,69 |
| -4,43 | -1,41 |
| -12,99 | 0,26 |
| -4,14 | -0,69 |
| -6,31 | -0,43 |
| NAN | -0,30 |
| -6,79 | -0,96 |
| -3,99 | -2,95 |
| -13,35 | -1,16 |

| Insulin change from baseline 2 yrs PD | HOMA-IR 24 gw | HOMA-IR 2 years PD |
|---|---|---|
| -3,94 | 1,23 | 1,20 |
| -1,97 | 1,54 | 1,94 |
| -3,33 | 1,36 | 2,46 |
| -2,36 | 4,25 | 2,91 |
| -0,04 | 3,01 | 3,36 |
| 0,77 | 4,26 | 2,38 |
| -3,26 | 1,24 | 1,15 |
| -1,05 | 1,73 | 1,28 |
| 0,03 | 2,15 | 1,47 |
| -1,58 | 3,06 | 6,15 |
| -1,20 | 1,44 | 1,32 |
| -3,50 | 2,16 | 9,61 |
| -1,91 | 1,21 | 0,77 |
| 0,28 | 2,78 | 1,97 |
| -1,13 | 1,68 | 1,28 |
| -3,83 | 1,74 | 1,70 |
| -0,17 | 1,88 | 1,11 |
| -7,31 | 3,41 | 9,53 |
| -4,50 | 1,34 | 1,74 |
| -2,07 | 2,82 | 3,25 |
| -2,49 | 2,23 | 1,88 |
| -4,23 | 3,25 | 3,96 |
| -5,59 | 4,30 | 5,19 |
| -4,00 | 0,98 | 1,72 |
| -3,80 | 3,74 | 4,00 |
| -10,84 | 2,20 | 3,40 |
| -5,97 | 2,30 | 2,27 |
| -31,27 | 2,02 | 34,74 |
| -2,86 | 4,48 | 3,41 |
| -1,24 | 4,67 | 2,28 |
| -2,97 | 2,77 | 3,54 |
| -2,98 | 1,44 | 2,41 |
| -4,75 | 4,53 | 1,85 |
| -5,10 | 2,66 | 3,45 |
| -0,14 | 1,39 | 1,71 |
| -5,37 | 2,15 | 6,44 |
| -5,85 | 1,14 | 1,99 |
| -1,49 | 2,88 | 3,36 |
| -3,63 | 3,90 | 4,65 |
| 0,66 | 1,80 | 1,82 |
| -0,75 | 3,18 | 2,00 |
| -3,16 | 4,30 | 3,28 |
| -2,79 | 2,42 | 2,72 |
| 0,19 | 1,63 | 1,28 |
| -4,76 | 2,24 | 2,41 |
| 0,40 | 1,80 | 1,11 |
| -0,44 | 2,37 | 1,60 |

| | | |
|---|---|---|
| -2,11 | 1,47 | 0,92 |
| -2,82 | 2,23 | 10,68 |
| -1,35 | 3,29 | 2,34 |
| -2,17 | 2,88 | 3,97 |
| -3,59 | 1,53 | 3,10 |
| -6,06 | 6,05 | 3,34 |
| -3,14 | 2,65 | 9,52 |
| -1,79 | NAN | 1,53 |
| -1,50 | 3,17 | 2,16 |
| 0,68 | 1,13 | 1,12 |
| 0,63 | 4,49 | 2,07 |
| 0,46 | 1,90 | 1,90 |
| -2,44 | 3,22 | 2,29 |
| -0,08 | 2,06 | 1,71 |
| 0,73 | 6,17 | 2,77 |
| -0,58 | NAN | 10,71 |
| -4,40 | 2,88 | 2,85 |
| 0,57 | 2,14 | 1,51 |
| 0,35 | 2,62 | 2,49 |
| -2,32 | 2,55 | 2,33 |
| -0,31 | 1,72 | 2,20 |
| -2,67 | 2,53 | 1,72 |
| -40,84 | NAN | 25,29 |
| -3,46 | 1,43 | 1,20 |
| -0,77 | 3,48 | 10,54 |
| -4,15 | 10,80 | 1,75 |
| 0,43 | 2,62 | 1,92 |
| -1,83 | 3,77 | 4,46 |
| -2,36 | 2,08 | 2,50 |
| -3,90 | 1,80 | 1,61 |
| -3,47 | 1,53 | 2,56 |
| -6,13 | 1,58 | 1,90 |
| -4,28 | 3,52 | 3,20 |
| -2,53 | 1,60 | 1,32 |
| -6,09 | 1,84 | 1,87 |
| 0,16 | 4,08 | 2,52 |
| -0,47 | 1,71 | 2,21 |
| -3,26 | 2,03 | 4,97 |
| -0,85 | 2,19 | 2,96 |
| -6,31 | 2,10 | 2,17 |
| 0,63 | 2,02 | 1,32 |
| -2,57 | 5,27 | 3,81 |
| -1,27 | 3,00 | 3,26 |
| -3,50 | 1,38 | 1,35 |
| -2,38 | 1,41 | 1,46 |
| 0,32 | 2,27 | 2,36 |
| -5,40 | 11,37 | 1,86 |
| -0,81 | 2,50 | 0,85 |
| 0,17 | 3,78 | 2,37 |
| -1,13 | 1,22 | 1,55 |

| | | |
|---|---|---|
| -3,54 | 1,83 | 1,43 |
| -3,83 | 2,87 | 2,85 |
| 0,50 | 1,25 | 1,36 |
| -7,42 | 18,81 | 6,81 |
| 0,35 | 3,55 | 1,85 |
| -25,12 | 3,08 | 7,51 |
| -0,77 | 2,70 | 3,23 |
| 0,47 | 1,79 | 1,75 |
| 0,44 | 3,10 | 2,96 |
| NAN | 1,39 | NAN |
| -3,70 | 1,90 | 2,34 |
| -3,11 | 1,75 | 3,23 |
| -3,41 | 0,99 | 1,21 |
| -10,91 | 1,39 | 5,47 |
| -9,78 | 4,48 | 10,81 |
| -2,70 | 4,13 | 2,16 |
| NAN | 1,69 | NAN |
| 0,25 | 1,57 | 0,85 |
| -7,98 | 1,96 | 3,05 |
| -12,88 | 2,61 | 5,55 |
| 0,74 | 1,09 | 0,62 |
| -0,17 | 2,09 | 2,21 |
| 0,65 | 3,35 | 2,75 |
| -2,48 | 2,37 | 1,30 |
| -1,81 | 5,18 | 2,74 |
| -6,21 | 1,73 | 3,65 |
| 0,67 | 2,44 | 1,96 |
| -2,74 | 2,55 | 1,65 |
| -7,92 | 4,55 | 16,50 |
| -0,52 | 1,68 | 1,78 |
| -0,74 | 18,54 | 3,60 |
| NAN | 1,36 | 1,05 |
| -5,13 | 0,87 | 1,74 |
| -2,69 | 2,21 | 2,54 |
| -2,88 | 4,63 | 36,54 |
| 0,34 | 3,26 | 1,66 |
| -19,51 | 5,57 | 13,10 |
| -11,52 | 1,92 | 7,94 |
| -4,25 | 13,67 | 3,02 |
| -2,01 | 2,36 | 2,06 |
| -4,45 | 28,13 | 1,71 |
| -4,91 | 4,12 | 5,27 |
| 0,57 | 4,02 | 2,50 |
| -34,87 | 2,10 | 10,31 |
| 0,57 | 3,09 | 1,82 |
| -3,29 | 1,52 | 1,20 |
| -5,46 | 1,61 | 1,78 |
| -3,53 | 2,47 | 1,68 |
| -2,14 | 1,77 | 2,03 |
| -1,10 | 3,34 | 2,03 |

| | | |
|---|---|---|
| -0,57 | 1,83 | 2,06 |
| -0,20 | 4,23 | 1,81 |
| -2,92 | 2,03 | 1,14 |
| -0,47 | 2,98 | 2,81 |
| 0,48 | 1,96 | 1,04 |
| -1,59 | 1,45 | 1,50 |
| 0,79 | 2,28 | 1,18 |
| -0,39 | 2,24 | 1,98 |
| 0,22 | 2,79 | 1,71 |
| -3,24 | 0,83 | 1,49 |
| NAN | 0,79 | NAN |
| 0,37 | 1,76 | 1,27 |
| -2,74 | 1,77 | 1,53 |
| 0,23 | 1,57 | 1,45 |
| -4,36 | 1,71 | 2,36 |
| -1,29 | 4,05 | 2,33 |
| -11,61 | 2,18 | 10,03 |
| -4,45 | 1,65 | 1,89 |
| 0,14 | 4,69 | 2,85 |
| -3,50 | 1,27 | 1,82 |
| -5,04 | 2,46 | 12,04 |
| 0,61 | 1,15 | 1,70 |
| -2,19 | 5,12 | 4,63 |
| 0,69 | 1,86 | 1,45 |
| -16,54 | 3,60 | 7,74 |
| -4,15 | 2,26 | 1,84 |
| 0,50 | 1,27 | 0,91 |
| -4,08 | 13,49 | 3,23 |
| -1,64 | 3,19 | 2,40 |
| 0,54 | 0,92 | 0,79 |
| -3,19 | 1,54 | 1,11 |
| -1,43 | 1,67 | 2,09 |
| -1,89 | 2,33 | 1,42 |
| -4,15 | 1,36 | 2,88 |
| -3,84 | 2,76 | 16,60 |
| -2,76 | 2,48 | 2,12 |
| -6,31 | 2,95 | 3,17 |
| -1,85 | 1,60 | 1,82 |
| 0,60 | 1,93 | 1,85 |
| -3,16 | 2,06 | 3,24 |
| 0,47 | 1,74 | 2,03 |
| -4,78 | 1,99 | 2,09 |
| -3,59 | 1,20 | 1,32 |
| 0,03 | 1,48 | 0,96 |
| 0,39 | 2,42 | 2,86 |
| 0,67 | 2,33 | 1,89 |
| -1,11 | 1,37 | 1,16 |
| -3,46 | 2,13 | 1,72 |
| 0,39 | 1,22 | 1,04 |
| -0,13 | 1,83 | 1,96 |

| | | |
|---|---|---|
| -11,90 | 3,15 | 9,30 |
| 0,38 | 1,96 | 2,06 |
| -0,18 | 1,60 | 1,71 |
| -2,31 | 2,62 | 2,71 |
| 0,25 | 1,08 | 1,82 |
| -5,36 | 2,31 | 3,61 |
| -3,53 | 1,73 | 1,19 |
| -2,14 | 2,08 | 2,60 |
| -5,93 | 1,10 | 3,55 |
| -2,21 | 1,76 | 1,81 |
| -1,61 | 1,13 | 1,36 |
| -0,88 | 1,83 | 1,87 |
| 0,20 | 1,08 | 0,64 |
| -2,29 | 3,65 | 3,96 |
| 0,71 | 10,16 | 1,34 |
| -7,21 | 2,86 | 2,78 |
| -0,06 | 2,21 | 1,95 |
| -1,72 | 1,99 | 1,19 |
| -0,30 | 1,10 | 2,11 |
| -0,38 | 7,89 | 1,58 |
| -1,65 | 3,22 | 1,79 |
| 0,36 | 2,63 | 2,42 |
| -4,69 | 1,63 | 1,58 |
| 0,72 | 1,83 | 1,15 |
| -2,03 | 1,20 | 2,44 |
| -6,40 | 2,70 | 4,96 |
| -2,24 | 2,19 | 1,89 |
| -2,92 | 1,37 | 2,55 |
| -3,80 | 2,22 | 1,85 |
| 0,19 | 2,04 | 1,42 |
| -6,83 | 4,41 | 2,18 |
| -2,67 | 1,87 | 1,67 |
| -5,47 | 2,02 | 3,01 |
| -0,54 | 1,60 | 2,86 |
| 0,37 | 2,08 | 1,93 |
| -3,86 | 3,12 | 3,53 |
| -0,87 | 1,62 | 0,95 |
| 0,58 | 1,28 | 1,24 |
| -2,66 | 1,40 | 3,90 |
| -2,02 | 1,74 | 2,65 |
| -2,87 | 6,51 | 1,97 |
| -2,73 | 2,29 | 1,86 |
| -4,48 | 1,56 | 1,97 |
| -1,35 | 3,12 | 1,72 |
| -1,76 | 0,88 | 0,95 |
| -4,07 | 0,80 | 1,72 |
| -3,24 | 2,53 | 1,66 |
| -0,39 | 3,05 | 3,10 |
| 0,66 | 1,42 | 0,83 |
| -1,08 | 2,26 | 2,27 |

| | | |
|---|---|---|
| -3,57 | 2,19 | 1,85 |
| 0,08 | 1,96 | 1,27 |
| -7,55 | 4,00 | 8,25 |
| -0,76 | 3,25 | 4,42 |
| -1,98 | 2,53 | 3,17 |
| 0,44 | 1,43 | 1,34 |
| -2,55 | 1,40 | 1,58 |
| -0,63 | 1,48 | 5,00 |
| -0,40 | 1,22 | 1,71 |
| -5,46 | 1,61 | 2,08 |
| -1,99 | NAN | 2,13 |
| -5,16 | 1,31 | 2,43 |
| -5,66 | 2,08 | 1,89 |
| 0,54 | 7,03 | 1,74 |
| -1,94 | 4,05 | 3,24 |
| -2,74 | 5,04 | 4,71 |
| -1,61 | 1,00 | 2,42 |
| 0,34 | 1,70 | 1,23 |
| -3,82 | 0,98 | 1,62 |
| 0,35 | 2,75 | 1,85 |
| -3,44 | 1,54 | 1,29 |
| -1,24 | 2,32 | 2,00 |
| -15,95 | 2,79 | 5,95 |
| -0,41 | 2,71 | 2,08 |
| -0,26 | 3,56 | 2,93 |
| -0,10 | 1,74 | 1,13 |
| -23,48 | 3,18 | 30,78 |
| -1,90 | 1,36 | 1,93 |
| -2,08 | 6,21 | 1,04 |
| -3,42 | 3,12 | 2,32 |
| 0,56 | 1,87 | 1,49 |
| -5,76 | 1,92 | 4,95 |
| -4,00 | 2,55 | 2,35 |
| -0,52 | 1,76 | 1,80 |
| -6,37 | 1,37 | 16,60 |
| -0,84 | 1,67 | 1,55 |
| -7,27 | 0,99 | 2,35 |
| 0,21 | 0,92 | 1,42 |
| 0,25 | 1,53 | 2,07 |
| -3,24 | 0,78 | 1,85 |
| -2,08 | NAN | 1,48 |
| -1,04 | 2,08 | 9,00 |
| -0,08 | 9,71 | 2,02 |
| -2,81 | 1,86 | 2,16 |
| -0,41 | 1,05 | 2,69 |
| -4,30 | 1,32 | 1,60 |
| -3,33 | 1,00 | 2,77 |
| -2,16 | 1,32 | 1,56 |
| -0,29 | 1,23 | 1,49 |
| -0,03 | 2,28 | 8,16 |

| | | |
|---|---|---|
| 0,54 | 1,97 | 2,67 |
| -2,85 | 1,96 | 3,06 |
| -0,38 | 3,31 | 4,03 |
| 0,49 | 1,12 | 1,64 |
| -0,21 | 1,90 | 2,52 |
| -0,70 | 0,85 | 1,19 |
| -3,74 | 2,39 | 4,36 |
| -3,81 | 0,81 | 2,75 |
| -2,13 | 1,05 | 1,48 |
| -0,81 | 2,58 | 7,87 |
| -0,69 | 2,37 | 2,70 |
| -2,67 | 0,77 | 2,25 |
| NAN | 0,91 | NAN |
| -1,59 | 1,83 | 2,82 |
| -2,22 | 1,40 | 1,42 |
| -6,54 | 0,98 | 4,03 |

| HOMA-IR increase 24 gw | HOMA-IR change 24 gw |
|---|---|
| -0,66 | -1,15 |
| -0,08 | -0,05 |
| -0,15 | -0,12 |
| -2,34 | -1,22 |
| 2,96 | 0,50 |
| 18,44 | 0,81 |
| -0,57 | -0,85 |
| -0,26 | -0,18 |
| 1,50 | 0,41 |
| 2,02 | 0,40 |
| -0,01 | 0,00 |
| 1,75 | 0,45 |
| -0,54 | -0,81 |
| 3,45 | 0,55 |
| -0,14 | -0,09 |
| -0,86 | -0,98 |
| 0,68 | 0,26 |
| -0,74 | -0,28 |
| -0,65 | -0,95 |
| -0,53 | -0,23 |
| -1,20 | -1,15 |
| -1,57 | -0,94 |
| -2,59 | -1,51 |
| -0,13 | -0,15 |
| -1,96 | -1,10 |
| -1,57 | -2,50 |
| -1,59 | -2,22 |
| -0,03 | -0,02 |
| -2,63 | -1,42 |
| -2,79 | -1,49 |
| -0,81 | -0,42 |
| -0,18 | -0,14 |
| -3,92 | -6,49 |
| -1,49 | -1,27 |
| 2,28 | 0,62 |
| 0,26 | 0,11 |
| -0,54 | -0,89 |
| 0,03 | 0,01 |
| -1,91 | -0,96 |
| 8,95 | 0,83 |
| -1,07 | -0,50 |
| -2,53 | -1,42 |
| -0,70 | -0,41 |
| 1,74 | 0,52 |
| -1,28 | -1,34 |
| 2,48 | 0,58 |
| 0,12 | 0,05 |

| | |
|---|---|
| -0,79 | -1,15 |
| 3,82 | 0,63 |
| -0,76 | -0,30 |
| -0,52 | -0,22 |
| -0,07 | -0,05 |
| -5,08 | -5,24 |
| 2,90 | 0,52 |
| NAN | NAN |
| -1,06 | -0,50 |
| 6,12 | 0,84 |
| 8,04 | 0,64 |
| 6,40 | 0,77 |
| -1,78 | -1,24 |
| 1,32 | 0,39 |
| 15,41 | 0,71 |
| NAN | NAN |
| -1,83 | -1,74 |
| 5,41 | 0,72 |
| 4,53 | 0,63 |
| -0,90 | -0,55 |
| 1,82 | 0,51 |
| -1,48 | -1,40 |
| NAN | NAN |
| -0,79 | -1,23 |
| 11,05 | 0,76 |
| -10,17 | -16,16 |
| 5,20 | 0,66 |
| -0,41 | -0,12 |
| -0,70 | -0,51 |
| -0,95 | -1,12 |
| -0,44 | -0,41 |
| -0,96 | -1,54 |
| -2,45 | -2,27 |
| -0,84 | -1,12 |
| -1,36 | -2,83 |
| 2,34 | 0,36 |
| 1,21 | 0,41 |
| -0,11 | -0,06 |
| 1,31 | 0,37 |
| -1,59 | -3,10 |
| 6,33 | 0,76 |
| -3,12 | -1,45 |
| -0,31 | -0,11 |
| -0,77 | -1,25 |
| -0,25 | -0,21 |
| 5,46 | 0,71 |
| -10,83 | -20,31 |
| -1,37 | -1,21 |
| 2,64 | 0,41 |
| 0,22 | 0,16 |

| | |
|---|---|
| -1,12 | -1,57 |
| -1,57 | -1,21 |
| 4,83 | 0,79 |
| -16,74 | -8,10 |
| 3,27 | 0,48 |
| -2,43 | -3,70 |
| 1,24 | 0,31 |
| 5,49 | 0,75 |
| 8,48 | 0,73 |
| 2,39 | 0,63 |
| -0,78 | -0,70 |
| -0,27 | -0,18 |
| -0,38 | -0,63 |
| -0,27 | -0,24 |
| -2,21 | -0,98 |
| -2,72 | -1,92 |
| -0,04 | -0,02 |
| 1,16 | 0,42 |
| -1,26 | -1,78 |
| -1,77 | -2,13 |
| 3,75 | 0,77 |
| 2,65 | 0,56 |
| 15,58 | 0,82 |
| -1,30 | -1,22 |
| -2,87 | -1,24 |
| -0,63 | -0,58 |
| 9,80 | 0,80 |
| -1,62 | -1,76 |
| -1,04 | -0,30 |
| 1,02 | 0,38 |
| -13,82 | -2,93 |
| NAN | NAN |
| -0,31 | -0,57 |
| -0,79 | -0,55 |
| 12,60 | 0,73 |
| 2,93 | 0,47 |
| -4,35 | -3,57 |
| -0,60 | -0,45 |
| -12,42 | -9,89 |
| -0,84 | -0,55 |
| -27,45 | -40,21 |
| -2,46 | -1,48 |
| 8,34 | 0,67 |
| -1,50 | -2,47 |
| 5,74 | 0,65 |
| -0,88 | -1,36 |
| -1,03 | -1,76 |
| -1,67 | -2,06 |
| -0,39 | -0,28 |
| -1,29 | -0,63 |

| | |
|---|---|
| 0,87 | 0,32 |
| -1,51 | -0,55 |
| -1,39 | -2,18 |
| 0,78 | 0,21 |
| 2,68 | 0,58 |
| -0,24 | -0,20 |
| 10,97 | 0,83 |
| 1,12 | 0,33 |
| 2,02 | 0,42 |
| -0,15 | -0,23 |
| -0,23 | -0,41 |
| 2,93 | 0,62 |
| -0,99 | -1,27 |
| 2,08 | 0,57 |
| -0,75 | -0,78 |
| -1,68 | -0,71 |
| -0,56 | -0,34 |
| -0,83 | -1,01 |
| 2,08 | 0,31 |
| -0,30 | -0,30 |
| 1,84 | 0,43 |
| 7,37 | 0,87 |
| -2,44 | -0,91 |
| 8,34 | 0,82 |
| -2,86 | -3,87 |
| -1,59 | -2,39 |
| 2,69 | 0,68 |
| -12,37 | -11,01 |
| -1,15 | -0,57 |
| 2,75 | 0,75 |
| -0,97 | -1,70 |
| 0,19 | 0,10 |
| -1,14 | -0,96 |
| -0,23 | -0,20 |
| 4,76 | 0,63 |
| -1,25 | -1,02 |
| -1,97 | -2,00 |
| -0,15 | -0,10 |
| 9,45 | 0,83 |
| -0,28 | -0,15 |
| 6,52 | 0,79 |
| -1,14 | -1,34 |
| -0,58 | -0,95 |
| 0,47 | 0,24 |
| 7,48 | 0,76 |
| 8,85 | 0,79 |
| -0,30 | -0,28 |
| -1,24 | -1,39 |
| 2,12 | 0,63 |
| 2,08 | 0,53 |

| | |
|---|---|
| -1,65 | -1,10 |
| 4,77 | 0,71 |
| 1,75 | 0,52 |
| -0,75 | -0,40 |
| 4,51 | 0,81 |
| -1,14 | -0,98 |
| -1,20 | -2,24 |
| -0,55 | -0,36 |
| 0,00 | 0,00 |
| -0,61 | -0,53 |
| -0,06 | -0,06 |
| 0,43 | 0,19 |
| 0,73 | 0,40 |
| -0,80 | -0,28 |
| 0,82 | 0,07 |
| -2,15 | -3,06 |
| 1,92 | 0,46 |
| -1,05 | -1,11 |
| 2,63 | 0,70 |
| -5,38 | -2,14 |
| -1,85 | -1,35 |
| 5,28 | 0,67 |
| -0,93 | -1,32 |
| 8,42 | 0,82 |
| 0,69 | 0,36 |
| -1,37 | -1,03 |
| -1,06 | -0,94 |
| 0,10 | 0,07 |
| -1,32 | -1,46 |
| 1,91 | 0,48 |
| -3,77 | -5,84 |
| -0,89 | -0,91 |
| -0,99 | -0,96 |
| 1,83 | 0,53 |
| 4,66 | 0,69 |
| -1,49 | -0,91 |
| -0,39 | -0,31 |
| 5,45 | 0,81 |
| 0,61 | 0,30 |
| 0,42 | 0,20 |
| -5,58 | -5,99 |
| -1,26 | -1,21 |
| -0,79 | -1,02 |
| -1,40 | -0,82 |
| -0,09 | -0,12 |
| -0,19 | -0,31 |
| -1,63 | -1,81 |
| 1,69 | 0,36 |
| 5,06 | 0,78 |
| 0,19 | 0,08 |

| | |
|---|---|
| -1,42 | -1,83 |
| 1,76 | 0,47 |
| -2,29 | -1,33 |
| 1,59 | 0,33 |
| -0,11 | -0,05 |
| 3,68 | 0,72 |
| -0,45 | -0,47 |
| 5,49 | 0,79 |
| 1,47 | 0,55 |
| -0,96 | -1,49 |
| NAN | NAN |
| -0,40 | -0,44 |
| -1,34 | -1,83 |
| 1,49 | 0,17 |
| -1,81 | -0,81 |
| -2,06 | -0,69 |
| 1,07 | 0,52 |
| 2,65 | 0,61 |
| -0,21 | -0,28 |
| 4,31 | 0,61 |
| -0,96 | -1,64 |
| -0,36 | -0,18 |
| -2,03 | -2,64 |
| 0,49 | 0,15 |
| 0,80 | 0,18 |
| 0,83 | 0,32 |
| -0,60 | -0,23 |
| -0,14 | -0,11 |
| -5,44 | -7,07 |
| -2,02 | -1,82 |
| 7,11 | 0,79 |
| -0,41 | -0,27 |
| -1,61 | -1,72 |
| 1,09 | 0,38 |
| 4,32 | 0,76 |
| 0,26 | 0,13 |
| -0,41 | -0,71 |
| 2,82 | 0,75 |
| 5,10 | 0,77 |
| 0,18 | 0,18 |
| NAN | NAN |
| 8,13 | 0,80 |
| -5,47 | -1,29 |
| -0,82 | -0,78 |
| 3,41 | 0,77 |
| -0,69 | -1,09 |
| 0,34 | 0,25 |
| -0,19 | -0,16 |
| 1,37 | 0,53 |
| 13,29 | 0,85 |

| | |
|---|---|
| 10,71 | 0,84 |
| -0,13 | -0,07 |
| 4,42 | 0,57 |
| 6,12 | 0,85 |
| 2,67 | 0,58 |
| 0,81 | 0,49 |
| -0,26 | -0,12 |
| 0,43 | 0,35 |
| -0,09 | -0,09 |
| 6,21 | 0,71 |
| 0,92 | 0,28 |
| 0,41 | 0,35 |
| 0,68 | 0,43 |
| 0,55 | 0,23 |
| -0,56 | -0,66 |
| 0,17 | 0,15 |

| HOMA-IR increase 2 yrs PD | HOMA-IR change 2 yrs PD |
|---|---|
| -0,63 | -0,52 |
| -0,48 | -0,25 |
| -1,25 | -0,51 |
| -1,00 | -0,34 |
| 2,60 | 0,77 |
| 20,32 | 8,55 |
| -0,48 | -0,42 |
| 0,19 | 0,15 |
| 2,18 | 1,48 |
| -1,07 | -0,17 |
| 0,11 | 0,09 |
| -5,69 | -0,59 |
| -0,10 | -0,13 |
| 4,26 | 2,17 |
| 0,26 | 0,20 |
| -0,82 | -0,48 |
| 1,45 | 1,31 |
| -6,86 | -0,72 |
| -1,05 | -0,60 |
| -0,95 | -0,29 |
| -0,84 | -0,45 |
| -2,28 | -0,58 |
| -3,48 | -0,67 |
| -0,87 | -0,51 |
| -2,21 | -0,55 |
| -2,77 | -0,81 |
| -1,56 | -0,69 |
| -32,75 | -0,94 |
| -1,56 | -0,46 |
| -0,41 | -0,18 |
| -1,58 | -0,45 |
| -1,15 | -0,48 |
| -1,25 | -0,67 |
| -2,28 | -0,66 |
| 1,97 | 1,15 |
| -4,03 | -0,63 |
| -1,39 | -0,70 |
| -0,45 | -0,13 |
| -2,66 | -0,57 |
| 8,93 | 4,90 |
| 0,12 | 0,06 |
| -1,50 | -0,46 |
| -1,01 | -0,37 |
| 2,09 | 1,63 |
| -1,45 | -0,60 |
| 3,18 | 2,86 |
| 0,89 | 0,55 |

| | |
|---|---|
| -0,23 | -0,25 |
| -4,64 | -0,43 |
| 0,18 | 0,08 |
| -1,61 | -0,41 |
| -1,64 | -0,53 |
| -2,37 | -0,71 |
| -3,97 | -0,42 |
| -0,42 | -0,27 |
| -0,05 | -0,02 |
| 6,13 | 5,49 |
| 10,46 | 5,04 |
| 6,40 | 3,37 |
| -0,85 | -0,37 |
| 1,67 | 0,97 |
| 18,81 | 6,79 |
| 4,27 | 0,40 |
| -1,80 | -0,63 |
| 6,04 | 4,00 |
| 4,67 | 1,87 |
| -0,69 | -0,29 |
| 1,33 | 0,60 |
| -0,66 | -0,39 |
| -23,96 | -0,95 |
| -0,56 | -0,46 |
| 3,99 | 0,38 |
| -1,12 | -0,64 |
| 5,91 | 3,08 |
| -1,10 | -0,25 |
| -1,13 | -0,45 |
| -0,76 | -0,47 |
| -1,47 | -0,58 |
| -1,28 | -0,67 |
| -2,12 | -0,66 |
| -0,56 | -0,43 |
| -1,39 | -0,74 |
| 3,90 | 1,55 |
| 0,72 | 0,32 |
| -3,05 | -0,61 |
| 0,54 | 0,18 |
| -1,65 | -0,76 |
| 7,03 | 5,33 |
| -1,66 | -0,44 |
| -0,57 | -0,17 |
| -0,73 | -0,54 |
| -0,30 | -0,21 |
| 5,37 | 2,28 |
| -1,33 | -0,71 |
| 0,28 | 0,33 |
| 4,05 | 1,71 |
| -0,11 | -0,07 |

| | |
|---|---|
| -0,72 | -0,50 |
| -1,55 | -0,54 |
| 4,71 | 3,46 |
| -4,75 | -0,70 |
| 4,97 | 2,69 |
| -6,86 | -0,91 |
| 0,71 | 0,22 |
| 5,53 | 3,16 |
| 8,63 | 2,92 |
| NAN | NAN |
| -1,23 | -0,52 |
| -1,75 | -0,54 |
| -0,61 | -0,50 |
| -4,34 | -0,79 |
| -8,54 | -0,79 |
| -0,75 | -0,35 |
| NAN | NAN |
| 1,87 | 2,19 |
| -2,35 | -0,77 |
| -4,72 | -0,85 |
| 4,22 | 6,84 |
| 2,53 | 1,14 |
| 16,18 | 5,89 |
| -0,24 | -0,18 |
| -0,43 | -0,16 |
| -2,56 | -0,70 |
| 10,28 | 5,26 |
| -0,72 | -0,44 |
| -12,99 | -0,79 |
| 0,92 | 0,52 |
| 1,13 | 0,31 |
| NAN | NAN |
| -1,19 | -0,68 |
| -1,11 | -0,44 |
| -19,31 | -0,53 |
| 4,53 | 2,72 |
| -11,88 | -0,91 |
| -6,62 | -0,83 |
| -1,77 | -0,58 |
| -0,54 | -0,26 |
| -1,03 | -0,60 |
| -3,61 | -0,69 |
| 9,86 | 3,95 |
| -9,70 | -0,94 |
| 7,01 | 3,84 |
| -0,55 | -0,46 |
| -1,19 | -0,67 |
| -0,87 | -0,52 |
| -0,65 | -0,32 |
| 0,02 | 0,01 |

| | |
|---|---|
| 0,63 | 0,31 |
| 0,92 | 0,51 |
| -0,50 | -0,44 |
| 0,95 | 0,34 |
| 3,59 | 3,44 |
| -0,28 | -0,19 |
| 12,07 | 10,19 |
| 1,38 | 0,70 |
| 3,10 | 1,82 |
| -0,82 | -0,55 |
| NAN | NAN |
| 3,43 | 2,71 |
| -0,74 | -0,49 |
| 2,20 | 1,51 |
| -1,40 | -0,59 |
| 0,05 | 0,02 |
| -8,41 | -0,84 |
| -1,07 | -0,57 |
| 3,92 | 1,37 |
| -0,84 | -0,46 |
| -7,74 | -0,64 |
| 6,82 | 4,02 |
| -1,95 | -0,42 |
| 8,75 | 6,05 |
| -7,00 | -0,90 |
| -1,17 | -0,64 |
| 3,05 | 3,37 |
| -2,10 | -0,65 |
| -0,37 | -0,15 |
| 2,88 | 3,64 |
| -0,53 | -0,48 |
| -0,23 | -0,11 |
| -0,24 | -0,17 |
| -1,76 | -0,61 |
| -9,09 | -0,55 |
| -0,89 | -0,42 |
| -2,18 | -0,69 |
| -0,38 | -0,21 |
| 9,53 | 5,14 |
| -1,46 | -0,45 |
| 6,23 | 3,07 |
| -1,24 | -0,59 |
| -0,70 | -0,53 |
| 0,99 | 1,03 |
| 7,04 | 2,46 |
| 9,28 | 4,91 |
| -0,09 | -0,08 |
| -0,83 | -0,48 |
| 2,30 | 2,22 |
| 1,95 | 0,99 |

| | |
|---|---|
| -7,80 | -0,84 |
| 4,66 | 2,26 |
| 1,64 | 0,96 |
| -0,84 | -0,31 |
| 3,77 | 2,07 |
| -2,45 | -0,68 |
| -0,66 | -0,55 |
| -1,07 | -0,41 |
| -2,45 | -0,69 |
| -0,66 | -0,37 |
| -0,29 | -0,21 |
| 0,39 | 0,21 |
| 1,16 | 1,81 |
| -1,11 | -0,28 |
| 9,63 | 7,17 |
| -2,07 | -0,75 |
| 2,18 | 1,12 |
| -0,25 | -0,21 |
| 1,62 | 0,77 |
| 0,93 | 0,59 |
| -0,42 | -0,24 |
| 5,49 | 2,27 |
| -0,88 | -0,56 |
| 9,10 | 7,91 |
| -0,55 | -0,23 |
| -3,63 | -0,73 |
| -0,76 | -0,40 |
| -1,08 | -0,42 |
| -0,95 | -0,51 |
| 2,54 | 1,79 |
| -1,54 | -0,70 |
| -0,69 | -0,41 |
| -1,97 | -0,66 |
| 0,57 | 0,20 |
| 4,80 | 2,48 |
| -1,90 | -0,54 |
| 0,28 | 0,29 |
| 5,50 | 4,44 |
| -1,89 | -0,48 |
| -0,48 | -0,18 |
| -1,04 | -0,53 |
| -0,83 | -0,45 |
| -1,20 | -0,61 |
| -0,01 | 0,00 |
| -0,16 | -0,17 |
| -1,11 | -0,64 |
| -0,76 | -0,46 |
| 1,63 | 0,53 |
| 5,66 | 6,83 |
| 0,18 | 0,08 |

| | |
|---|---|
| -1,07 | -0,58 |
| 2,45 | 1,93 |
| -6,53 | -0,79 |
| 0,42 | 0,10 |
| -0,75 | -0,24 |
| 3,77 | 2,82 |
| -0,62 | -0,39 |
| 1,97 | 0,39 |
| 0,98 | 0,57 |
| -1,44 | -0,69 |
| -0,69 | -0,33 |
| -1,52 | -0,62 |
| -1,15 | -0,61 |
| 6,78 | 3,89 |
| -1,00 | -0,31 |
| -1,73 | -0,37 |
| -0,36 | -0,15 |
| 3,12 | 2,53 |
| -0,85 | -0,53 |
| 5,21 | 2,82 |
| -0,71 | -0,55 |
| -0,04 | -0,02 |
| -5,18 | -0,87 |
| 1,12 | 0,54 |
| 1,43 | 0,49 |
| 1,44 | 1,28 |
| -28,20 | -0,92 |
| -0,70 | -0,36 |
| -0,27 | -0,26 |
| -1,22 | -0,52 |
| 7,49 | 5,02 |
| -3,44 | -0,69 |
| -1,41 | -0,60 |
| 1,06 | 0,59 |
| -10,91 | -0,66 |
| 0,38 | 0,24 |
| -1,77 | -0,75 |
| 2,32 | 1,63 |
| 4,56 | 2,20 |
| -0,89 | -0,48 |
| -0,54 | -0,36 |
| 1,21 | 0,13 |
| 2,22 | 1,10 |
| -1,11 | -0,52 |
| 1,77 | 0,66 |
| -0,97 | -0,61 |
| -1,42 | -0,51 |
| -0,43 | -0,27 |
| 1,12 | 0,75 |
| 7,42 | 0,91 |

| | |
|---|---|
| 10,01 | 3,75 |
| -1,24 | -0,40 |
| 3,71 | 0,92 |
| 5,60 | 3,42 |
| 2,04 | 0,81 |
| 0,47 | 0,39 |
| -2,22 | -0,51 |
| -1,51 | -0,55 |
| -0,52 | -0,35 |
| 0,92 | 0,12 |
| 0,59 | 0,22 |
| -1,07 | -0,48 |
| NAN | NAN |
| -0,44 | -0,16 |
| -0,57 | -0,40 |
| -2,88 | -0,72 |

| Adiponectin (mcg/mL) 24 gw | Adiponectin (mcg/mL) 2 yrs. PD |
|---|---|
| 18,11 | 15,80 |
| 12,14 | 11,99 |
| 18,07 | 12,98 |
| 15,42 | 17,95 |
| 22,42 | 12,60 |
| 14,46 | 15,93 |
| 14,57 | 10,10 |
| 10,02 | 5,42 |
| 20,33 | 14,55 |
| 14,89 | 9,40 |
| 21,44 | 26,57 |
| 54,62 | 14,88 |
| 14,50 | 9,66 |
| 23,04 | 10,80 |
| 17,70 | 23,31 |
| 42,82 | 26,46 |
| 16,18 | 22,82 |
| 14,74 | 10,98 |
| 29,11 | 30,97 |
| 4,42 | 3,46 |
| 25,10 | 29,90 |
| 20,65 | 16,31 |
| 20,57 | 24,16 |
| 24,79 | 19,63 |
| 23,86 | 15,23 |
| 29,41 | 21,01 |
| 23,69 | 30,30 |
| 32,63 | 17,86 |
| 30,72 | 31,49 |
| 19,83 | 10,25 |
| 5,55 | 7,86 |
| 21,98 | 13,64 |
| 19,74 | 9,43 |
| 11,43 | 23,58 |
| 13,07 | 20,59 |
| 23,04 | 19,30 |
| 38,18 | 36,45 |
| 9,94 | 8,69 |
| 6,93 | 7,18 |
| 16,98 | 9,44 |
| 6,49 | 9,02 |
| 25,28 | 15,49 |
| 19,07 | 18,18 |
| 18,51 | 19,65 |
| 34,93 | 20,77 |
| 31,36 | 28,52 |
| 23,88 | 21,96 |

| | |
|---|---|
| 23,91 | 34,59 |
| 35,06 | 26,10 |
| 24,81 | 28,51 |
| 22,27 | 15,73 |
| 18,20 | 18,36 |
| 10,92 | 10,99 |
| 20,69 | 23,11 |
| NAN | NAN |
| 22,80 | 12,08 |
| 31,31 | 24,11 |
| 18,56 | 35,52 |
| 28,69 | 22,12 |
| 27,77 | 28,14 |
| 13,83 | 20,12 |
| 15,23 | 17,87 |
| NAN | NAN |
| 20,14 | 15,32 |
| 26,79 | 21,82 |
| 10,33 | 5,29 |
| 15,58 | 18,05 |
| 10,19 | 8,50 |
| 32,56 | 16,53 |
| 7,53 | 6,96 |
| 20,53 | 16,29 |
| 12,08 | 11,54 |
| 16,57 | 17,75 |
| 12,33 | 15,18 |
| 9,33 | 11,89 |
| 19,99 | 13,87 |
| 17,32 | 20,86 |
| 19,85 | 17,27 |
| 17,40 | 24,40 |
| 18,49 | 14,17 |
| 28,63 | 21,07 |
| 21,60 | 29,57 |
| 11,77 | 9,65 |
| 24,73 | 27,79 |
| 5,97 | 9,04 |
| 16,01 | 16,20 |
| 14,46 | 11,46 |
| 19,83 | 22,94 |
| 16,76 | 23,71 |
| 2,30 | 7,08 |
| 24,72 | 22,93 |
| 30,91 | 26,51 |
| 6,69 | 9,08 |
| 23,74 | 38,92 |
| 14,40 | 22,39 |
| 8,06 | 9,08 |
| 8,97 | 17,01 |

| | |
|---|---|
| 20,81 | 19,10 |
| 13,26 | 19,75 |
| 3,10 | 3,90 |
| 8,31 | 8,72 |
| 13,05 | 15,14 |
| 12,92 | 25,32 |
| 22,34 | 28,12 |
| 16,66 | 21,09 |
| 13,82 | 15,25 |
| NAN | NAN |
| 32,58 | 17,42 |
| 5,02 | 4,37 |
| 14,34 | 10,42 |
| 39,62 | 35,23 |
| 12,71 | 16,16 |
| 21,43 | 23,07 |
| NAN | NAN |
| 15,72 | 17,94 |
| 8,57 | 11,92 |
| 17,42 | 14,13 |
| 15,35 | 15,70 |
| 16,87 | 15,84 |
| 12,67 | 16,94 |
| 13,74 | 32,57 |
| 11,76 | 11,14 |
| 14,35 | 14,40 |
| 15,13 | 11,79 |
| 15,53 | 21,19 |
| 14,63 | 8,40 |
| 21,89 | 39,63 |
| 11,93 | 20,07 |
| 9,52 | 9,01 |
| 14,71 | 15,12 |
| 21,89 | 15,03 |
| 7,35 | 9,47 |
| 17,71 | 18,33 |
| 11,31 | 9,41 |
| 11,73 | 14,92 |
| 14,17 | 12,89 |
| 16,17 | 12,77 |
| 12,71 | 14,37 |
| 6,97 | 9,38 |
| 8,99 | 8,18 |
| 12,45 | 9,51 |
| NAN | NAN |
| 16,34 | 14,83 |
| 16,32 | 19,69 |
| 10,52 | 12,14 |
| 16,93 | 10,36 |
| 9,16 | 10,25 |

| | |
|---|---|
| 9,54 | 9,67 |
| 11,76 | 7,65 |
| 13,80 | 15,60 |
| 14,57 | 13,54 |
| 12,09 | 17,63 |
| 9,89 | 18,44 |
| 21,36 | 14,39 |
| 23,38 | 15,41 |
| 17,44 | 17,30 |
| 21,31 | 21,38 |
| 17,83 | 18,31 |
| 18,47 | 18,30 |
| 41,68 | 22,84 |
| 9,29 | 12,29 |
| 18,08 | 13,47 |
| 9,88 | 17,21 |
| 14,92 | 13,64 |
| 12,47 | 10,83 |
| 8,15 | 8,28 |
| 24,97 | 20,11 |
| 17,23 | 24,78 |
| 14,06 | 22,71 |
| 10,50 | 9,32 |
| 16,50 | 22,09 |
| 11,70 | 14,74 |
| 27,33 | 27,05 |
| 18,08 | 15,17 |
| 16,33 | 11,47 |
| 9,64 | 8,28 |
| 13,86 | 15,81 |
| 13,30 | 26,47 |
| 23,22 | 19,68 |
| 22,11 | 21,80 |
| 11,20 | 11,55 |
| 9,46 | 14,14 |
| 15,53 | 18,18 |
| 13,94 | 19,00 |
| 9,22 | 12,19 |
| 10,83 | 16,94 |
| 18,35 | 16,07 |
| 28,72 | 20,44 |
| 16,13 | 6,27 |
| 13,88 | 18,77 |
| 14,14 | 18,23 |
| 10,68 | 9,43 |
| 12,03 | 11,19 |
| 16,48 | 15,53 |
| 18,28 | 9,82 |
| 27,83 | 18,97 |
| 16,55 | 16,86 |

| | |
|---|---|
| 15,01 | 11,40 |
| 12,25 | 10,27 |
| 8,28 | 10,20 |
| 14,30 | 15,80 |
| 18,04 | 10,74 |
| 18,92 | 18,64 |
| 9,52 | 9,57 |
| 13,88 | 13,24 |
| 11,49 | 16,16 |
| 11,22 | 10,83 |
| 25,61 | 14,77 |
| 22,69 | 13,76 |
| 15,29 | 15,08 |
| 13,81 | 9,32 |
| 22,20 | 18,66 |
| 30,53 | 36,34 |
| 26,79 | 20,44 |
| 37,45 | 31,51 |
| 15,83 | 17,26 |
| 15,89 | 12,00 |
| 23,85 | 17,71 |
| 12,16 | 12,09 |
| 12,58 | 13,47 |
| 20,01 | 18,15 |
| 14,83 | 8,80 |
| 10,33 | 9,04 |
| 10,46 | 12,83 |
| 8,15 | 5,51 |
| 15,43 | 8,62 |
| 22,17 | 26,06 |
| 25,42 | 31,95 |
| 16,86 | 14,03 |
| 13,13 | 13,74 |
| 28,80 | 31,77 |
| 28,51 | 33,65 |
| 17,53 | 11,08 |
| 27,09 | 25,28 |
| NAN | NAN |
| NAN | NAN |
| NAN | NAN |
| NAN | NAN |
| NAN | NAN |
| NAN | NAN |
| NAN | NAN |
| NAN | NAN |
| NAN | NAN |
| NAN | NAN |
| NAN | NAN |
| NAN | NAN |
| NAN | NAN |

| | |
|---|---|
| NAN | NAN |
| NAN | NAN |
| NAN | NAN |
| NAN | NAN |
| NAN | NAN |
| NAN | NAN |
| NAN | NAN |
| NAN | NAN |
| NAN | NAN |
| NAN | NAN |
| NAN | NAN |
| NAN | NAN |
| NAN | NAN |
| NAN | NAN |
| NAN | NAN |
| NAN | NAN |
| NAN | NAN |
| NAN | NAN |
| NAN | NAN |
| NAN | NAN |
| NAN | NAN |
| NAN | NAN |
| NAN | NAN |
| NAN | NAN |
| NAN | NAN |
| NAN | NAN |
| NAN | NAN |
| NAN | NAN |
| NAN | NAN |
| NAN | NAN |
| NAN | NAN |
| NAN | NAN |
| NAN | NAN |
| NAN | NAN |
| NAN | NAN |
| NAN | NAN |
| NAN | NAN |
| NAN | NAN |
| NAN | NAN |
| NAN | NAN |
| NAN | NAN |
| NAN | NAN |
| NAN | NAN |
| NAN | NAN |
| NAN | NAN |
| NAN | NAN |
| NAN | NAN |
| NAN | NAN |
| NAN | NAN |
| NAN | NAN |

| | |
|---|---|
| NAN | NAN |
| NAN | NAN |
| NAN | NAN |
| NAN | NAN |
| NAN | NAN |
| NAN | NAN |
| NAN | NAN |
| NAN | NAN |
| NAN | NAN |
| NAN | NAN |
| NAN | NAN |
| NAN | NAN |
| NAN | NAN |
| NAN | NAN |
| NAN | NAN |
| NAN | NAN |

| Adiponectin increase 24 gw | Adiponectin increase 2 yrs. PD |
|---|---|
| 5,29 | 7,60 |
| -6,99 | -6,84 |
| 2,30 | 7,39 |
| 11,01 | 8,48 |
| 1,73 | 11,55 |
| 4,64 | 3,17 |
| -2,56 | 1,91 |
| -2,46 | 2,14 |
| -0,94 | 4,84 |
| 5,83 | 11,32 |
| 5,29 | 0,16 |
| -21,09 | 18,65 |
| 0,31 | 5,15 |
| -8,22 | 4,02 |
| 10,71 | 5,10 |
| -7,77 | 8,59 |
| 9,77 | 3,13 |
| -0,63 | 3,13 |
| 5,83 | 3,97 |
| 2,00 | 2,96 |
| 3,98 | -0,82 |
| 5,78 | 10,12 |
| 1,32 | -2,27 |
| 5,94 | 11,10 |
| 4,23 | 12,86 |
| -7,24 | 1,16 |
| 3,66 | -2,95 |
| 14,14 | 28,91 |
| 12,35 | 11,58 |
| 7,55 | 17,13 |
| 3,52 | 1,21 |
| -1,29 | 7,05 |
| 7,10 | 17,41 |
| 6,81 | -5,34 |
| 3,73 | -3,79 |
| 5,86 | 9,60 |
| -2,96 | -1,23 |
| 1,38 | 2,63 |
| 0,24 | -0,01 |
| -0,77 | 6,77 |
| -0,35 | -2,88 |
| -7,13 | 2,66 |
| 0,34 | 1,23 |
| -4,32 | -5,46 |
| -0,71 | 13,45 |
| -14,26 | -11,42 |
| 7,16 | 9,08 |

| | |
|---|---|
| 6,54 | -4,14 |
| -4,05 | 4,91 |
| -4,38 | -8,08 |
| 5,42 | 11,96 |
| -5,01 | -5,17 |
| 10,68 | 10,61 |
| 16,91 | 14,49 |
| NAN | NAN |
| -2,37 | 8,35 |
| -5,04 | 2,16 |
| 2,74 | -14,22 |
| 9,43 | 16,00 |
| NAN | NAN |
| -0,65 | -6,94 |
| 3,05 | 0,41 |
| NAN | NAN |
| -8,42 | -3,60 |
| -0,82 | 4,15 |
| 0,21 | 5,25 |
| 5,41 | 2,94 |
| 1,31 | 3,00 |
| 5,35 | 21,38 |
| 4,32 | 4,89 |
| 6,59 | 10,83 |
| 0,03 | 0,57 |
| 9,90 | 8,72 |
| 7,43 | 4,58 |
| 22,10 | 19,54 |
| -1,86 | 4,26 |
| 16,41 | 12,87 |
| 5,65 | 8,23 |
| 9,06 | 2,06 |
| 3,10 | 7,42 |
| -5,80 | 1,76 |
| 11,97 | 4,00 |
| 7,60 | 9,72 |
| 9,16 | 6,10 |
| -0,55 | -3,62 |
| -6,39 | -6,58 |
| -0,36 | 2,64 |
| 9,01 | 5,90 |
| 1,54 | -5,41 |
| 8,37 | 3,59 |
| 6,51 | 8,30 |
| -1,75 | 2,65 |
| 3,59 | 1,20 |
| -4,26 | -19,44 |
| 13,39 | 5,40 |
| 5,17 | 4,15 |
| 10,73 | 2,69 |

| | |
|---|---|
| 2,41 | 4,12 |
| 7,50 | 1,01 |
| 19,70 | 18,90 |
| 20,84 | 20,43 |
| 4,83 | 2,74 |
| 6,71 | -5,69 |
| -0,76 | -6,54 |
| -1,69 | -6,12 |
| 2,66 | 1,23 |
| NAN | NAN |
| -7,37 | 7,79 |
| 2,11 | 2,76 |
| 4,39 | 8,31 |
| -6,44 | -2,05 |
| -1,17 | -4,62 |
| 4,91 | 3,27 |
| NAN | NAN |
| 13,23 | 11,01 |
| 2,73 | -0,62 |
| -0,78 | 2,51 |
| 6,05 | 5,70 |
| 0,26 | 1,29 |
| 17,18 | 12,91 |
| 32,55 | 13,72 |
| -2,23 | -1,61 |
| 4,40 | 4,35 |
| 1,28 | 4,62 |
| 6,15 | 0,49 |
| -0,01 | 6,22 |
| 0,39 | -17,35 |
| 5,91 | -2,23 |
| 32,88 | 33,39 |
| 0,10 | -0,31 |
| -7,48 | -0,62 |
| 3,71 | 1,59 |
| -4,81 | -5,43 |
| -1,11 | 0,79 |
| -4,62 | -7,81 |
| 0,47 | 1,75 |
| -0,32 | 3,08 |
| 7,50 | 5,84 |
| 1,70 | -0,71 |
| -1,56 | -0,75 |
| 2,79 | 5,73 |
| NAN | NAN |
| 4,38 | 5,89 |
| 8,07 | 4,70 |
| 1,13 | -0,49 |
| -1,03 | 5,54 |
| 2,21 | 1,12 |

| | |
|---|---|
| 5,78 | 5,65 |
| 2,34 | 6,45 |
| -1,39 | -3,19 |
| 8,92 | 9,95 |
| 15,59 | 10,05 |
| 4,31 | -4,24 |
| 6,58 | 13,55 |
| -14,90 | -6,93 |
| 3,33 | 3,47 |
| 4,28 | 4,21 |
| 0,19 | -0,29 |
| 2,32 | 2,49 |
| 2,25 | 21,09 |
| 4,15 | 1,15 |
| 4,32 | 8,93 |
| 4,35 | -2,98 |
| 3,86 | 5,14 |
| 3,94 | 5,58 |
| 4,41 | 4,28 |
| 6,60 | 11,46 |
| -0,28 | -7,83 |
| 10,13 | 1,48 |
| 3,72 | 4,90 |
| 1,23 | -4,36 |
| 6,07 | 3,03 |
| -17,66 | -17,38 |
| 13,32 | 16,23 |
| 2,32 | 7,18 |
| 3,21 | 4,57 |
| 2,14 | 0,19 |
| 27,84 | 14,67 |
| -5,32 | -1,78 |
| 0,47 | 0,78 |
| 1,34 | 0,99 |
| 6,34 | 1,66 |
| 9,34 | 6,69 |
| 5,00 | -0,06 |
| 2,23 | -0,74 |
| 2,01 | -4,10 |
| -2,85 | -0,57 |
| -3,78 | 4,50 |
| -9,26 | 0,60 |
| 7,37 | 2,48 |
| 2,71 | -1,38 |
| 0,91 | 2,16 |
| 0,01 | 0,85 |
| 0,17 | 1,12 |
| -6,51 | 1,95 |
| -5,46 | 3,40 |
| 2,31 | 2,00 |

| | |
|---|---|
| -1,27 | 2,34 |
| -0,99 | 0,99 |
| 0,36 | -1,56 |
| 0,28 | -1,22 |
| -2,06 | 5,24 |
| 1,86 | 2,14 |
| -2,75 | -2,80 |
| 0,81 | 1,45 |
| 1,66 | -3,01 |
| 4,79 | 5,18 |
| -9,33 | 1,51 |
| 0,14 | 9,07 |
| 5,75 | 5,96 |
| 1,25 | 5,74 |
| -1,67 | 1,87 |
| 11,81 | 6,00 |
| -5,66 | 0,69 |
| -2,41 | 3,53 |
| 11,17 | 9,74 |
| 17,61 | 21,50 |
| 1,91 | 8,05 |
| 3,01 | 3,08 |
| -0,18 | -1,07 |
| 10,34 | 12,20 |
| 1,03 | 7,06 |
| 2,61 | 3,90 |
| 1,99 | -0,38 |
| -0,36 | 2,28 |
| -0,34 | 6,47 |
| -0,87 | -4,76 |
| 3,69 | -2,84 |
| 2,07 | 4,90 |
| -2,51 | -3,12 |
| 2,18 | -0,79 |
| 6,52 | 1,38 |
| -6,27 | 0,18 |
| 3,89 | 5,70 |
| NAN | NAN |
| NAN | NAN |
| NAN | NAN |
| NAN | NAN |
| NAN | NAN |
| NAN | NAN |
| NAN | NAN |
| NAN | NAN |
| NAN | NAN |
| NAN | NAN |
| NAN | NAN |
| NAN | NAN |
| NAN | NAN |

| | |
|---|---|
| NAN | NAN |
| NAN | NAN |
| NAN | NAN |
| NAN | NAN |
| NAN | NAN |
| NAN | NAN |
| NAN | NAN |
| NAN | NAN |
| NAN | NAN |
| NAN | NAN |
| NAN | NAN |
| NAN | NAN |
| NAN | NAN |
| NAN | NAN |
| NAN | NAN |
| NAN | NAN |
| NAN | NAN |
| NAN | NAN |
| NAN | NAN |
| NAN | NAN |
| NAN | NAN |
| NAN | NAN |
| NAN | NAN |
| NAN | NAN |
| NAN | NAN |
| NAN | NAN |
| NAN | NAN |
| NAN | NAN |
| NAN | NAN |
| NAN | NAN |
| NAN | NAN |
| NAN | NAN |
| NAN | NAN |
| NAN | NAN |
| NAN | NAN |
| NAN | NAN |
| NAN | NAN |
| NAN | NAN |
| NAN | NAN |
| NAN | NAN |
| NAN | NAN |
| NAN | NAN |
| NAN | NAN |
| NAN | NAN |
| NAN | NAN |
| NAN | NAN |
| NAN | NAN |
| NAN | NAN |
| NAN | NAN |
| NAN | NAN |

| | |
|---|---|
| NAN | NAN |
| NAN | NAN |
| NAN | NAN |
| NAN | NAN |
| NAN | NAN |
| NAN | NAN |
| NAN | NAN |
| NAN | NAN |
| NAN | NAN |
| NAN | NAN |
| NAN | NAN |
| NAN | NAN |
| NAN | NAN |
| NAN | NAN |
| NAN | NAN |
| NAN | NAN |

| Adiponectin change 24 gw | Adiponectin change 2 yrs PD | IL-6 (pg/mL) 24 gw | IL-6 (pg/mL) 2 Yrs PD |
|---|---|---|---|
| 0,23 | 0,32 | 3,25 | 2,17 |
| -1,36 | -1,33 | 3,78 | 2,98 |
| 0,11 | 0,36 | 11,36 | 6,19 |
| 0,42 | 0,32 | 1,90 | 3,11 |
| 0,07 | 0,48 | 3,51 | 3,52 |
| 0,24 | 0,17 | 5,59 | 9,11 |
| -0,21 | 0,16 | 1,03 | 0,87 |
| -0,33 | 0,28 | 1,97 | 2,78 |
| -0,05 | 0,25 | 2,31 | 2,31 |
| 0,28 | 0,55 | 2,71 | 3,72 |
| 0,20 | 0,01 | 1,90 | 2,84 |
| -0,63 | 0,56 | 2,84 | 1,70 |
| 0,02 | 0,35 | 2,24 | 2,44 |
| -0,55 | 0,27 | 2,31 | 1,90 |
| 0,38 | 0,18 | 2,14 | 1,60 |
| -0,22 | 0,25 | 11,85 | 7,85 |
| 0,38 | 0,12 | 7,04 | 10,21 |
| -0,04 | 0,22 | 9,93 | 16,22 |
| 0,17 | 0,11 | 3,31 | 3,61 |
| 0,31 | 0,46 | 3,51 | 2,14 |
| 0,14 | -0,03 | 17,22 | 5,16 |
| 0,22 | 0,38 | 8,08 | 11,14 |
| 0,06 | -0,10 | 4,12 | 6,00 |
| 0,19 | 0,36 | 13,10 | 5,58 |
| 0,15 | 0,46 | 4,95 | 5,79 |
| -0,33 | 0,05 | 4,95 | 13,67 |
| 0,13 | -0,11 | 2,62 | 4,33 |
| 0,30 | 0,62 | 3,51 | 2,14 |
| 0,29 | 0,27 | 2,56 | 3,06 |
| 0,28 | 0,63 | 7,79 | 1,87 |
| 0,39 | 0,13 | 9,02 | 21,07 |
| -0,06 | 0,34 | 7,67 | 2,53 |
| 0,26 | 0,65 | 1,61 | 3,25 |
| 0,37 | -0,29 | 13,74 | 2,96 |
| 0,22 | -0,23 | 1,88 | 2,96 |
| 0,20 | 0,33 | 2,21 | 2,63 |
| -0,08 | -0,03 | 5,91 | 6,80 |
| 0,12 | 0,23 | 8,45 | 7,77 |
| 0,03 | 0,00 | 3,27 | 5,21 |
| -0,05 | 0,42 | 12,51 | 2,74 |
| -0,06 | -0,47 | 2,10 | 1,35 |
| -0,39 | 0,15 | 7,04 | 10,86 |
| 0,02 | 0,06 | 0,01 | 8,45 |
| -0,30 | -0,38 | 1,88 | 1,35 |
| -0,02 | 0,39 | 0,01 | 0,01 |
| -0,83 | -0,67 | 1,35 | 1,54 |
| 0,23 | 0,29 | 39,67 | 31,01 |

| | | | |
|---|---|---|---|
| 0,21 | -0,14 | 2,93 | 1,92 |
| -0,13 | 0,16 | 3,15 | 6,23 |
| -0,21 | -0,40 | 21,25 | 1,45 |
| 0,20 | 0,43 | 3,00 | 2,31 |
| -0,38 | -0,39 | 0,01 | 0,01 |
| 0,49 | 0,49 | 12,11 | 10,32 |
| 0,45 | 0,39 | 2,16 | 3,15 |
| NAN | NAN | NAN | NAN |
| -0,12 | 0,41 | 8,17 | 9,19 |
| -0,19 | 0,08 | 1,53 | 10,39 |
| 0,13 | -0,67 | 1,29 | 5,81 |
| 0,25 | 0,42 | 2,24 | 3,00 |
| NAN | NAN | 11,50 | 1,45 |
| -0,05 | -0,53 | 7,37 | 2,56 |
| 0,17 | 0,02 | 1,13 | 2,89 |
| NAN | NAN | NAN | NAN |
| -0,72 | -0,31 | 6,52 | 0,01 |
| -0,03 | 0,16 | 2,22 | 2,56 |
| 0,02 | 0,50 | 4,97 | 14,09 |
| 0,26 | 0,14 | 3,55 | 3,31 |
| 0,11 | 0,26 | 13,19 | 5,19 |
| 0,14 | 0,56 | 0,01 | 1,70 |
| 0,36 | 0,41 | 2,65 | 2,81 |
| 0,24 | 0,40 | 4,34 | 11,21 |
| 0,00 | 0,05 | 3,47 | 11,07 |
| 0,37 | 0,33 | 0,01 | 0,01 |
| 0,38 | 0,23 | 0,01 | 0,01 |
| 0,70 | 0,62 | 2,98 | 4,11 |
| -0,10 | 0,23 | 1,87 | 0,93 |
| 0,49 | 0,38 | 1,96 | 8,81 |
| 0,22 | 0,32 | 0,01 | 0,01 |
| 0,34 | 0,08 | 0,01 | 1,70 |
| 0,14 | 0,34 | 1,32 | 3,20 |
| -0,25 | 0,08 | 13,06 | 9,19 |
| 0,36 | 0,12 | 8,87 | 0,87 |
| 0,39 | 0,50 | 8,09 | 7,54 |
| 0,27 | 0,18 | 2,60 | 3,19 |
| -0,10 | -0,67 | 2,31 | 1,66 |
| -0,66 | -0,68 | 1,32 | 4,86 |
| -0,03 | 0,19 | 0,95 | 2,54 |
| 0,31 | 0,20 | 3,49 | 2,31 |
| 0,08 | -0,30 | 2,88 | 4,85 |
| 0,78 | 0,34 | 3,20 | 3,20 |
| 0,21 | 0,27 | 3,38 | 1,68 |
| -0,06 | 0,09 | 3,18 | 0,01 |
| 0,35 | 0,12 | NAN | NAN |
| -0,22 | -1,00 | 5,90 | 16,78 |
| 0,48 | 0,19 | 2,88 | 4,27 |
| 0,39 | 0,31 | 10,86 | 12,61 |
| 0,54 | 0,14 | 0,01 | 0,01 |

| | | | |
|---|---|---|---|
| 0,10 | 0,18 | 4,66 | 4,66 |
| 0,36 | 0,05 | 3,25 | 2,90 |
| 0,86 | 0,83 | 2,90 | 2,18 |
| 0,71 | 0,70 | 4,93 | 5,64 |
| 0,27 | 0,15 | 2,11 | 0,99 |
| 0,34 | -0,29 | 1,89 | 1,59 |
| -0,04 | -0,30 | 13,60 | 15,17 |
| -0,11 | -0,41 | 0,01 | 0,99 |
| 0,16 | 0,07 | 2,83 | 0,98 |
| NAN | NAN | NAN | NAN |
| -0,29 | 0,31 | 0,01 | 0,01 |
| 0,30 | 0,39 | 2,04 | 3,86 |
| 0,23 | 0,44 | 10,11 | 13,94 |
| -0,19 | -0,06 | 5,06 | 1,89 |
| -0,10 | -0,40 | 7,23 | 11,42 |
| 0,19 | 0,12 | 1,84 | 2,77 |
| NAN | NAN | NAN | NAN |
| 0,46 | 0,38 | 2,71 | 14,59 |
| 0,24 | -0,05 | 7,09 | 17,34 |
| -0,05 | 0,15 | 2,41 | 3,18 |
| 0,28 | 0,27 | 107,31 | 198,48 |
| 0,02 | 0,08 | 1,84 | 0,84 |
| 0,58 | 0,43 | 5,14 | 7,49 |
| 0,70 | 0,30 | 4,27 | 4,03 |
| -0,23 | -0,17 | 13,87 | 19,64 |
| 0,23 | 0,23 | 16,92 | 13,23 |
| 0,08 | 0,28 | 4,76 | 7,07 |
| 0,28 | 0,02 | 2,12 | 4,83 |
| 0,00 | 0,43 | 11,12 | 9,50 |
| 0,02 | -0,78 | 8,90 | 10,98 |
| 0,33 | -0,13 | 22,23 | 8,30 |
| 0,78 | 0,79 | 7,54 | 11,94 |
| 0,01 | -0,02 | 7,44 | 0,01 |
| -0,52 | -0,04 | 0,01 | 6,16 |
| 0,34 | 0,14 | 2,81 | 0,96 |
| -0,37 | -0,42 | 0,01 | 7,25 |
| -0,11 | 0,08 | 3,51 | 3,62 |
| -0,65 | -1,10 | 1,95 | 13,33 |
| 0,03 | 0,12 | 2,10 | 2,05 |
| -0,02 | 0,19 | 9,97 | 10,33 |
| 0,37 | 0,29 | 5,34 | 8,49 |
| 0,20 | -0,08 | 4,83 | 12,12 |
| -0,21 | -0,10 | 35,31 | 17,69 |
| 0,18 | 0,38 | 10,43 | 4,28 |
| NAN | NAN | 1,08 | 1,87 |
| 0,21 | 0,28 | 10,33 | 8,86 |
| 0,33 | 0,19 | 0,87 | 0,85 |
| 0,10 | -0,04 | 34,36 | 84,28 |
| -0,06 | 0,35 | 7,16 | 7,64 |
| 0,19 | 0,10 | 1,57 | 2,23 |

| | | | |
|---|---|---|---|
| 0,38 | 0,37 | 2,36 | 0,87 |
| 0,17 | 0,46 | 15,80 | 36,60 |
| -0,11 | -0,26 | 0,96 | 0,01 |
| 0,38 | 0,42 | 3,94 | 2,35 |
| 0,56 | 0,36 | 2,30 | 1,50 |
| 0,30 | -0,30 | 0,96 | 23,67 |
| 0,24 | 0,48 | 0,96 | 1,02 |
| -1,76 | -0,82 | 0,01 | 0,01 |
| 0,16 | 0,17 | 0,01 | 0,87 |
| 0,17 | 0,16 | 0,01 | 0,01 |
| 0,01 | -0,02 | 0,01 | 1,77 |
| 0,11 | 0,12 | 3,80 | 1,77 |
| 0,05 | 0,48 | 0,01 | 0,01 |
| 0,31 | 0,09 | 0,89 | 0,90 |
| 0,19 | 0,40 | 0,01 | 0,01 |
| 0,31 | -0,21 | 2,55 | 6,37 |
| 0,21 | 0,27 | 1,02 | 0,01 |
| 0,24 | 0,34 | 0,01 | 0,01 |
| 0,35 | 0,34 | 11,67 | 23,71 |
| 0,21 | 0,36 | 3,09 | 2,62 |
| -0,02 | -0,46 | 1,48 | 0,95 |
| 0,42 | 0,06 | 0,87 | 1,42 |
| 0,26 | 0,34 | 1,24 | 1,10 |
| 0,07 | -0,25 | 1,42 | 2,76 |
| 0,34 | 0,17 | 4,07 | 0,01 |
| -1,83 | -1,80 | 0,01 | 4,72 |
| 0,42 | 0,52 | 0,01 | 3,47 |
| 0,12 | 0,38 | 0,01 | 0,01 |
| 0,25 | 0,36 | 0,01 | 0,01 |
| 0,13 | 0,01 | 1,40 | 0,83 |
| 0,68 | 0,36 | 2,75 | 5,00 |
| -0,30 | -0,10 | 0,01 | 9,35 |
| 0,02 | 0,03 | 67,42 | 30,77 |
| 0,11 | 0,08 | 1,34 | 1,47 |
| 0,40 | 0,11 | 8,34 | 26,11 |
| 0,38 | 0,27 | 4,16 | 3,52 |
| 0,26 | 0,00 | 2,52 | 0,01 |
| 0,19 | -0,06 | 1,40 | 2,47 |
| 0,16 | -0,32 | 4,90 | 0,01 |
| -0,18 | -0,04 | 0,86 | 2,30 |
| -0,15 | 0,18 | 0,01 | 8,19 |
| -1,35 | 0,09 | 3,30 | 1,83 |
| 0,35 | 0,12 | NAN | NAN |
| 0,16 | -0,08 | NAN | NAN |
| 0,08 | 0,19 | NAN | NAN |
| 0,00 | 0,07 | NAN | NAN |
| 0,01 | 0,07 | NAN | NAN |
| -0,55 | 0,17 | NAN | NAN |
| -0,24 | 0,15 | NAN | NAN |
| 0,12 | 0,11 | NAN | NAN |

| | | | |
|---|---|---|---|
| -0,09 | 0,17 | NAN | NAN |
| -0,09 | 0,09 | NAN | NAN |
| 0,04 | -0,18 | NAN | NAN |
| 0,02 | -0,08 | NAN | NAN |
| -0,13 | 0,33 | NAN | NAN |
| 0,09 | 0,10 | 0,01 | 0,01 |
| -0,41 | -0,41 | NAN | NAN |
| 0,06 | 0,10 | 0,01 | 0,01 |
| 0,13 | -0,23 | 0,01 | 0,01 |
| 0,30 | 0,32 | NAN | NAN |
| -0,57 | 0,09 | 15,64 | 2,97 |
| 0,01 | 0,40 | NAN | NAN |
| 0,27 | 0,28 | NAN | NAN |
| 0,08 | 0,38 | 0,01 | 11,02 |
| -0,08 | 0,09 | NAN | NAN |
| 0,28 | 0,14 | 34,38 | 27,43 |
| -0,27 | 0,03 | 7,83 | 27,07 |
| -0,07 | 0,10 | NAN | NAN |
| 0,41 | 0,36 | NAN | NAN |
| 0,53 | 0,64 | NAN | NAN |
| 0,07 | 0,31 | NAN | NAN |
| 0,20 | 0,20 | 2,79 | 0,86 |
| -0,01 | -0,09 | 0,01 | 0,01 |
| 0,34 | 0,40 | 87,19 | 107,32 |
| 0,06 | 0,45 | 0,01 | 12,15 |
| 0,20 | 0,30 | 0,01 | 0,01 |
| 0,16 | -0,03 | 3,30 | 13,11 |
| -0,05 | 0,29 | 0,01 | 6,75 |
| -0,02 | 0,43 | 28,15 | 4,91 |
| -0,04 | -0,22 | 0,97 | 0,01 |
| 0,13 | -0,10 | 0,90 | 0,01 |
| 0,11 | 0,26 | 32,41 | 36,46 |
| -0,24 | -0,29 | 2,79 | 5,47 |
| 0,07 | -0,03 | 0,01 | 0,01 |
| 0,19 | 0,04 | 0,01 | 0,01 |
| -0,56 | 0,02 | 32,85 | 0,80 |
| 0,13 | 0,18 | 0,01 | 0,01 |
| NAN | NAN | 0,01 | 0,01 |
| NAN | NAN | 4,72 | 0,01 |
| NAN | NAN | 1,56 | 8,88 |
| NAN | NAN | 0,01 | 0,01 |
| NAN | NAN | 0,01 | 0,01 |
| NAN | NAN | NAN | NAN |
| NAN | NAN | 0,01 | 0,01 |
| NAN | NAN | 0,80 | 0,01 |
| NAN | NAN | 0,01 | 3,42 |
| NAN | NAN | 29,43 | 4,20 |
| NAN | NAN | 16,88 | 3,55 |
| NAN | NAN | 0,01 | 0,91 |
| NAN | NAN | NAN | NAN |

| | | | |
|---|---|---|---|
| NAN | NAN | 1,43 | 0,88 |
| NAN | NAN | 1,60 | 4,76 |
| NAN | NAN | 1,43 | 0,01 |
| NAN | NAN | 0,01 | 0,01 |
| NAN | NAN | NAN | NAN |
| NAN | NAN | 0,49 | 0,43 |
| NAN | NAN | 43,36 | 23,63 |
| NAN | NAN | 0,01 | 0,01 |
| NAN | NAN | 0,01 | 3,45 |
| NAN | NAN | 1,00 | 0,91 |
| NAN | NAN | NAN | NAN |
| NAN | NAN | 0,01 | 0,01 |
| NAN | NAN | 5,30 | 0,89 |
| NAN | NAN | 1,13 | 0,90 |
| NAN | NAN | 0,01 | 0,01 |
| NAN | NAN | NAN | NAN |
| NAN | NAN | NAN | NAN |
| NAN | NAN | NAN | NAN |
| NAN | NAN | NAN | NAN |
| NAN | NAN | NAN | NAN |
| NAN | NAN | NAN | NAN |
| NAN | NAN | NAN | NAN |
| NAN | NAN | NAN | NAN |
| NAN | NAN | 30,26 | 46,92 |
| NAN | NAN | 0,01 | 7,79 |
| NAN | NAN | 22,59 | 9,03 |
| NAN | NAN | 0,01 | 0,01 |
| NAN | NAN | NAN | NAN |
| NAN | NAN | NAN | NAN |
| NAN | NAN | 7,16 | 3,54 |
| NAN | NAN | 0,01 | 0,01 |
| NAN | NAN | 0,01 | 0,01 |
| NAN | NAN | NAN | NAN |
| NAN | NAN | NAN | NAN |
| NAN | NAN | 0,01 | 0,01 |
| NAN | NAN | 0,01 | 0,01 |
| NAN | NAN | 0,01 | 0,01 |
| NAN | NAN | 1,99 | 2,36 |
| NAN | NAN | 2,15 | 2,92 |
| NAN | NAN | 0,01 | 0,01 |
| NAN | NAN | NAN | NAN |
| NAN | NAN | 7,44 | 0,01 |
| NAN | NAN | 0,82 | 0,01 |
| NAN | NAN | 0,01 | 0,01 |
| NAN | NAN | 0,01 | 0,01 |
| NAN | NAN | 0,01 | 0,01 |
| NAN | NAN | NAN | NAN |
| NAN | NAN | NAN | NAN |
| NAN | NAN | NAN | NAN |
| NAN | NAN | NAN | NAN |

| | | | |
|---|---|---|---|
| NAN | NAN | 0,01 | 2,81 |
| NAN | NAN | 0,89 | 0,01 |
| NAN | NAN | 3,90 | 3,90 |
| NAN | NAN | 0,01 | 0,01 |
| NAN | NAN | 1,31 | 0,01 |
| NAN | NAN | 1,46 | 0,01 |
| NAN | NAN | 0,01 | 0,01 |
| NAN | NAN | 0,01 | 8,74 |
| NAN | NAN | 1,31 | 0,01 |
| NAN | NAN | 3,78 | 4,62 |
| NAN | NAN | 12,32 | 8,62 |
| NAN | NAN | 1,31 | 0,01 |
| NAN | NAN | NAN | NAN |
| NAN | NAN | NAN | NAN |
| NAN | NAN | NAN | NAN |
| NAN | NAN | NAN | NAN |

| IL-6 increase 24 gw | IL-6 increase 2 Yrs PD | IL-6 change 24 gw | IL-6 change 2Yrs PD |
|---|---|---|---|
| -0,21 | 0,87 | -0,07 | 0,29 |
| 1,21 | 2,01 | 0,24 | 0,40 |
| -6,17 | -1,00 | -1,19 | -0,19 |
| 5,29 | 4,08 | 0,74 | 0,57 |
| 1,21 | 1,20 | 0,26 | 0,25 |
| -2,21 | -5,73 | -0,65 | -1,70 |
| 0,54 | 0,70 | 0,34 | 0,45 |
| 0,20 | -0,61 | 0,09 | -0,28 |
| -0,88 | -0,88 | -0,62 | -0,62 |
| 0,07 | -0,94 | 0,03 | -0,34 |
| 0,07 | -0,87 | 0,04 | -0,44 |
| -1,55 | -0,41 | -1,20 | -0,32 |
| 1,81 | 1,61 | 0,45 | 0,40 |
| -0,68 | -0,27 | -0,42 | -0,17 |
| 0,57 | 1,11 | 0,21 | 0,41 |
| 13,66 | 17,66 | 0,54 | 0,69 |
| 3,08 | -0,09 | 0,30 | -0,01 |
| -3,31 | -9,60 | -0,50 | -1,45 |
| 0,00 | -0,30 | 0,00 | -0,09 |
| -0,99 | 0,38 | -0,39 | 0,15 |
| -7,70 | 4,36 | -0,81 | 0,46 |
| 10,31 | 7,25 | 0,56 | 0,39 |
| -1,31 | -3,19 | -0,47 | -1,14 |
| -6,79 | 0,73 | -1,08 | 0,12 |
| -1,24 | -2,08 | -0,33 | -0,56 |
| 4,47 | -4,25 | 0,47 | -0,45 |
| -1,11 | -2,82 | -0,74 | -1,87 |
| -0,20 | 1,17 | -0,06 | 0,35 |
| -0,29 | -0,79 | -0,13 | -0,35 |
| 1,35 | 7,27 | 0,15 | 0,80 |
| 9,53 | -2,52 | 0,51 | -0,14 |
| -1,17 | 3,97 | -0,18 | 0,61 |
| -1,60 | -3,24 | -160,00 | -324,00 |
| 1,27 | 12,05 | 0,08 | 0,80 |
| 0,43 | -0,65 | 0,19 | -0,28 |
| 0,10 | -0,32 | 0,04 | -0,14 |
| 2,25 | 1,36 | 0,28 | 0,17 |
| -3,04 | -2,36 | -0,56 | -0,44 |
| 0,42 | -1,52 | 0,11 | -0,41 |
| -8,41 | 1,36 | -2,05 | 0,33 |
| -0,11 | 0,64 | -0,06 | 0,32 |
| 6,79 | 2,97 | 0,49 | 0,21 |
| 0,00 | -8,44 | 0,00 | -844,00 |
| 9,79 | 10,32 | 0,84 | 0,88 |
| 0,00 | 0,00 | 0,00 | 0,00 |
| 0,19 | 0,00 | 0,12 | 0,00 |
| 14,72 | 23,38 | 0,27 | 0,43 |

| | | | |
|---|---|---|---|
| -0,08 | 0,93 | -0,03 | 0,33 |
| -0,76 | -3,84 | -0,32 | -1,61 |
| -3,48 | 16,32 | -0,20 | 0,92 |
| 0,75 | 1,44 | 0,20 | 0,38 |
| 2,86 | 2,86 | 1,00 | 1,00 |
| -11,24 | -9,45 | -12,92 | -10,86 |
| 0,23 | -0,76 | 0,10 | -0,32 |
| NAN | NAN | NAN | NAN |
| 1,90 | 0,88 | 0,19 | 0,09 |
| 0,39 | -8,47 | 0,20 | -4,41 |
| 0,00 | -4,52 | 0,00 | -3,50 |
| -0,47 | -1,23 | -0,27 | -0,69 |
| -9,42 | 0,63 | -4,53 | 0,30 |
| -1,47 | 3,34 | -0,25 | 0,57 |
| -0,26 | -2,02 | -0,30 | -2,32 |
| NAN | NAN | NAN | NAN |
| -1,14 | 5,37 | -0,21 | 1,00 |
| -0,17 | -0,51 | -0,08 | -0,25 |
| 0,99 | -8,13 | 0,17 | -1,36 |
| 4,03 | 4,27 | 0,53 | 0,56 |
| -4,42 | 3,58 | -0,50 | 0,41 |
| 0,00 | -1,69 | 0,00 | -169,00 |
| -1,56 | -1,72 | -1,43 | -1,58 |
| 3,61 | -3,26 | 0,45 | -0,41 |
| -0,91 | -8,51 | -0,36 | -3,32 |
| 0,00 | 0,00 | 0,00 | 0,00 |
| 0,00 | 0,00 | 0,00 | 0,00 |
| 9,20 | 8,07 | 0,76 | 0,66 |
| 0,99 | 1,93 | 0,35 | 0,67 |
| -1,95 | -8,80 | -195,00 | -880,00 |
| 0,00 | 0,00 | 0,00 | 0,00 |
| 0,00 | -1,69 | 0,00 | -169,00 |
| 1,29 | -0,59 | 0,49 | -0,23 |
| 0,41 | 4,28 | 0,03 | 0,32 |
| -7,01 | 0,99 | -3,77 | 0,53 |
| 6,04 | 6,59 | 0,43 | 0,47 |
| -0,39 | -0,98 | -0,18 | -0,44 |
| -2,30 | -1,65 | -230,00 | -165,00 |
| -1,31 | -4,85 | -131,00 | -485,00 |
| 0,00 | -1,59 | 0,00 | -1,67 |
| 2,54 | 3,72 | 0,42 | 0,62 |
| 0,70 | -1,27 | 0,20 | -0,35 |
| -3,19 | -3,19 | -319,00 | -319,00 |
| -2,15 | -0,45 | -1,75 | -0,37 |
| 0,15 | 3,32 | 0,05 | 1,00 |
| NAN | NAN | NAN | NAN |
| 1,00 | -9,88 | 0,14 | -1,43 |
| -2,04 | -3,43 | -2,43 | -4,08 |
| 1,75 | 0,00 | 0,14 | 0,00 |
| 0,00 | 0,00 | 0,00 | 0,00 |

| | | | |
|---|---|---|---|
| 3,19 | 3,19 | 0,41 | 0,41 |
| -0,92 | -0,57 | -0,39 | -0,24 |
| 45,10 | 45,82 | 0,94 | 0,95 |
| -1,61 | -2,32 | -0,48 | -0,70 |
| 0,58 | 1,70 | 0,22 | 0,63 |
| 5,40 | 5,70 | 0,74 | 0,78 |
| -1,85 | -3,42 | -0,16 | -0,29 |
| 0,00 | -0,98 | 0,00 | -98,00 |
| -0,50 | 1,35 | -0,21 | 0,58 |
| NAN | NAN | NAN | NAN |
| 1,58 | 1,58 | 0,99 | 0,99 |
| -1,01 | -2,83 | -0,98 | -2,75 |
| 10,53 | 6,70 | 0,51 | 0,32 |
| 3,84 | 7,01 | 0,43 | 0,79 |
| 8,21 | 4,02 | 0,53 | 0,26 |
| -0,17 | -1,10 | -0,10 | -0,66 |
| NAN | NAN | NAN | NAN |
| -0,25 | -12,13 | -0,10 | -4,93 |
| 1,29 | -8,96 | 0,15 | -1,07 |
| -1,57 | -2,34 | -1,87 | -2,79 |
| -53,34 | -144,51 | -0,99 | -2,68 |
| 1,64 | 2,64 | 0,47 | 0,76 |
| -1,66 | -4,01 | -0,48 | -1,15 |
| -0,40 | -0,16 | -0,10 | -0,04 |
| 14,73 | 8,96 | 0,52 | 0,31 |
| 7,51 | 11,20 | 0,31 | 0,46 |
| 1,43 | -0,88 | 0,23 | -0,14 |
| -1,16 | -3,87 | -1,21 | -4,03 |
| 2,54 | 4,16 | 0,19 | 0,30 |
| -4,46 | -6,54 | -1,00 | -1,47 |
| -4,22 | 9,71 | -0,23 | 0,54 |
| 15,71 | 11,31 | 0,68 | 0,49 |
| -2,10 | 5,33 | -0,39 | 1,00 |
| 1,08 | -5,07 | 0,99 | -4,65 |
| 0,81 | 2,66 | 0,22 | 0,73 |
| 0,00 | -7,24 | 0,00 | -724,00 |
| 4,22 | 4,11 | 0,55 | 0,53 |
| -1,94 | -13,32 | -194,00 | -1332,00 |
| 1,53 | 1,58 | 0,42 | 0,44 |
| -1,01 | -1,37 | -0,11 | -0,15 |
| -4,37 | -7,52 | -4,51 | -7,75 |
| -0,44 | -7,73 | -0,10 | -1,76 |
| -1,95 | 15,67 | -0,06 | 0,47 |
| -2,41 | 3,74 | -0,30 | 0,47 |
| -1,07 | -1,86 | -107,00 | -186,00 |
| -3,57 | -2,10 | -0,53 | -0,31 |
| -0,86 | -0,84 | -86,00 | -84,00 |
| -0,46 | -50,38 | -0,01 | -1,49 |
| -2,90 | -3,38 | -0,68 | -0,79 |
| -0,59 | -1,25 | -0,60 | -1,28 |

| | | | |
|---|---|---|---|
| -0,06 | 1,43 | -0,03 | 0,62 |
| -3,92 | -24,72 | -0,33 | -2,08 |
| -0,95 | 0,00 | -95,00 | 0,00 |
| -2,47 | -0,88 | -1,68 | -0,60 |
| 6,16 | 6,96 | 0,73 | 0,82 |
| 2,30 | -20,41 | 0,71 | -6,26 |
| -0,95 | -1,01 | -95,00 | -101,00 |
| 0,00 | 0,00 | 0,00 | 0,00 |
| 0,00 | -0,86 | 0,00 | -86,00 |
| 0,00 | 0,00 | 0,00 | 0,00 |
| 0,00 | -1,76 | 0,00 | -176,00 |
| 1,21 | 3,24 | 0,24 | 0,65 |
| 0,00 | 0,00 | 0,00 | 0,00 |
| 1,34 | 1,33 | 0,60 | 0,60 |
| 0,00 | 0,00 | 0,00 | 0,00 |
| -0,98 | -4,80 | -0,62 | -3,06 |
| -1,01 | 0,00 | -101,00 | 0,00 |
| 0,00 | 0,00 | 0,00 | 0,00 |
| -3,29 | -15,33 | -0,39 | -1,83 |
| -1,05 | -0,58 | -0,51 | -0,28 |
| -0,67 | -0,14 | -0,83 | -0,17 |
| -0,04 | -0,59 | -0,05 | -0,71 |
| 0,35 | 0,49 | 0,22 | 0,31 |
| -0,23 | -1,57 | -0,19 | -1,32 |
| -2,63 | 1,43 | -1,83 | 0,99 |
| 0,80 | -3,91 | 0,99 | -4,83 |
| 0,00 | -3,46 | 0,00 | -346,00 |
| 1,87 | 1,87 | 0,99 | 0,99 |
| 0,00 | 0,00 | 0,00 | 0,00 |
| -0,37 | 0,20 | -0,36 | 0,19 |
| -0,26 | -2,51 | -0,10 | -1,01 |
| 0,00 | -9,34 | 0,00 | -934,00 |
| 7,59 | 44,24 | 0,10 | 0,59 |
| 3,76 | 3,63 | 0,74 | 0,71 |
| 8,07 | -9,70 | 0,49 | -0,59 |
| 2,51 | 3,15 | 0,38 | 0,47 |
| -2,51 | 0,00 | -251,00 | 0,00 |
| 0,55 | -0,52 | 0,28 | -0,27 |
| -1,49 | 3,40 | -0,44 | 1,00 |
| 3,78 | 2,34 | 0,81 | 0,50 |
| 0,00 | -8,18 | 0,00 | -818,00 |
| 1,34 | 2,81 | 0,29 | 0,61 |
| NAN | NAN | NAN | NAN |
| NAN | NAN | NAN | NAN |
| NAN | NAN | NAN | NAN |
| NAN | NAN | NAN | NAN |
| NAN | NAN | NAN | NAN |
| NAN | NAN | NAN | NAN |
| NAN | NAN | NAN | NAN |
| NAN | NAN | NAN | NAN |

| | | | |
|---|---|---|---|
| NAN | NAN | NAN | NAN |
| NAN | NAN | NAN | NAN |
| NAN | NAN | NAN | NAN |
| NAN | NAN | NAN | NAN |
| NAN | NAN | NAN | NAN |
| 0,00 | 0,00 | 0,00 | 0,00 |
| NAN | NAN | NAN | NAN |
| 0,00 | 0,00 | 0,00 | 0,00 |
| 0,00 | 0,00 | 0,00 | 0,00 |
| NAN | NAN | NAN | NAN |
| 11,15 | 23,82 | 0,42 | 0,89 |
| NAN | NAN | NAN | NAN |
| NAN | NAN | NAN | NAN |
| 0,00 | -11,01 | 0,00 | -1101,00 |
| NAN | NAN | NAN | NAN |
| 10,63 | 17,58 | 0,24 | 0,39 |
| 21,89 | 2,65 | 0,74 | 0,09 |
| NAN | NAN | NAN | NAN |
| NAN | NAN | NAN | NAN |
| NAN | NAN | NAN | NAN |
| NAN | NAN | NAN | NAN |
| -0,49 | 1,44 | -0,21 | 0,63 |
| 0,00 | 0,00 | 0,00 | 0,00 |
| 2,58 | -17,55 | 0,03 | -0,20 |
| 0,00 | -12,14 | 0,00 | -1214,00 |
| 0,00 | 0,00 | 0,00 | 0,00 |
| 2,17 | -7,64 | 0,40 | -1,40 |
| 0,86 | -5,88 | 0,99 | -6,76 |
| -25,85 | -2,61 | -11,24 | -1,13 |
| 0,52 | 1,48 | 0,35 | 0,99 |
| 0,48 | 1,37 | 0,35 | 0,99 |
| 6,53 | 2,48 | 0,17 | 0,06 |
| -2,01 | -4,69 | -2,58 | -6,01 |
| 0,00 | 0,00 | 0,00 | 0,00 |
| 0,00 | 0,00 | 0,00 | 0,00 |
| -6,96 | 25,09 | -0,27 | 0,97 |
| 0,00 | 0,00 | 0,00 | 0,00 |
| 0,00 | 0,00 | 0,00 | 0,00 |
| 16,04 | 20,75 | 0,77 | 1,00 |
| 0,89 | -6,43 | 0,36 | -2,62 |
| 0,00 | 0,00 | 0,00 | 0,00 |
| 0,79 | 0,79 | 0,99 | 0,99 |
| NAN | NAN | NAN | NAN |
| 0,00 | 0,00 | 0,00 | 0,00 |
| 0,04 | 0,83 | 0,05 | 0,99 |
| 0,61 | -2,80 | 0,98 | -4,52 |
| -29,42 | -4,19 | -2942,00 | -419,00 |
| -5,92 | 7,41 | -0,54 | 0,68 |
| 0,00 | -0,90 | 0,00 | -90,00 |
| NAN | NAN | NAN | NAN |

| | | | |
|---|---|---|---|
| -1,42 | -0,87 | -142,00 | -87,00 |
| 6,11 | 2,95 | 0,79 | 0,38 |
| 3,44 | 4,86 | 0,71 | 1,00 |
| 0,00 | 0,00 | 0,00 | 0,00 |
| NAN | NAN | NAN | NAN |
| 5,54 | 5,60 | 0,92 | 0,93 |
| 8,26 | 27,99 | 0,16 | 0,54 |
| 0,00 | 0,00 | 0,00 | 0,00 |
| 0,00 | -3,44 | 0,00 | -344,00 |
| -0,99 | -0,90 | -99,00 | -90,00 |
| NAN | NAN | NAN | NAN |
| 0,00 | 0,00 | 0,00 | 0,00 |
| -0,62 | 3,79 | -0,13 | 0,81 |
| 3,45 | 3,68 | 0,75 | 0,80 |
| 0,00 | 0,00 | 0,00 | 0,00 |
| NAN | NAN | NAN | NAN |
| NAN | NAN | NAN | NAN |
| NAN | NAN | NAN | NAN |
| NAN | NAN | NAN | NAN |
| NAN | NAN | NAN | NAN |
| NAN | NAN | NAN | NAN |
| NAN | NAN | NAN | NAN |
| NAN | NAN | NAN | NAN |
| 10,54 | -6,12 | 0,26 | -0,15 |
| 0,00 | -7,78 | 0,00 | -778,00 |
| 10,15 | 23,71 | 0,31 | 0,72 |
| 0,00 | 0,00 | 0,00 | 0,00 |
| NAN | NAN | NAN | NAN |
| NAN | NAN | NAN | NAN |
| -1,46 | 2,16 | -0,26 | 0,38 |
| 0,00 | 0,00 | 0,00 | 0,00 |
| 0,00 | 0,00 | 0,00 | 0,00 |
| NAN | NAN | NAN | NAN |
| NAN | NAN | NAN | NAN |
| 0,00 | 0,00 | 0,00 | 0,00 |
| 0,00 | 0,00 | 0,00 | 0,00 |
| 0,81 | 0,81 | 0,99 | 0,99 |
| 0,59 | 0,22 | 0,23 | 0,09 |
| -0,05 | -0,82 | -0,02 | -0,39 |
| 0,00 | 0,00 | 0,00 | 0,00 |
| NAN | NAN | NAN | NAN |
| -3,70 | 3,73 | -0,99 | 1,00 |
| -0,81 | 0,00 | -81,00 | 0,00 |
| 0,00 | 0,00 | 0,00 | 0,00 |
| 0,00 | 0,00 | 0,00 | 0,00 |
| 0,81 | 0,81 | 0,99 | 0,99 |
| NAN | NAN | NAN | NAN |
| NAN | NAN | NAN | NAN |
| NAN | NAN | NAN | NAN |
| NAN | NAN | NAN | NAN |

| | | | |
|---|---|---|---|
| 3,89 | 1,09 | 1,00 | 0,28 |
| -0,88 | 0,00 | -88,00 | 0,00 |
| 4,72 | 4,72 | 0,55 | 0,55 |
| 0,00 | 0,00 | 0,00 | 0,00 |
| -1,30 | 0,00 | -130,00 | 0,00 |
| -0,11 | 1,34 | -0,08 | 0,99 |
| 0,00 | 0,00 | 0,00 | 0,00 |
| 0,00 | -8,73 | 0,00 | -873,00 |
| -1,30 | 0,00 | -130,00 | 0,00 |
| 1,07 | 0,23 | 0,22 | 0,05 |
| -9,15 | -5,45 | -2,89 | -1,72 |
| -1,30 | 0,00 | -130,00 | 0,00 |
| NAN | NAN | NAN | NAN |
| NAN | NAN | NAN | NAN |
| NAN | NAN | NAN | NAN |
| NAN | NAN | NAN | NAN |

| Leptin (ng/ml) 24 gw | Leptin (ng/ml) 2 yrs. PD | Leptin increase 24 gw |
|---|---|---|
| 8,43 | 2,28 | -5,58 |
| 25,84 | 14,57 | 3,34 |
| 15,17 | 7,90 | -0,83 |
| 18,40 | 16,99 | 1,76 |
| 11,21 | 13,60 | -3,49 |
| 6,13 | 5,75 | -4,48 |
| 3,27 | 2,17 | -1,68 |
| 38,16 | 25,33 | -9,35 |
| 16,72 | 11,44 | -5,15 |
| 27,42 | 28,02 | -7,77 |
| 15,32 | 3,58 | -10,10 |
| 24,04 | 16,27 | -1,88 |
| 5,76 | 3,11 | -3,41 |
| 18,70 | 16,49 | -3,84 |
| 3,58 | 2,26 | 0,02 |
| 5,17 | 4,87 | -0,34 |
| 11,79 | 2,54 | -7,26 |
| 7,02 | 4,19 | -1,83 |
| 6,04 | 14,41 | -2,28 |
| 8,78 | 9,76 | 4,88 |
| 4,29 | 5,25 | -1,37 |
| 18,84 | 16,07 | -10,76 |
| 30,78 | 29,84 | -6,59 |
| 20,06 | 3,05 | -12,77 |
| 29,46 | 16,25 | -2,92 |
| 11,03 | 8,93 | -7,64 |
| 20,43 | 16,24 | -12,36 |
| 26,24 | 20,61 | -10,09 |
| 8,05 | 1,30 | -4,25 |
| 10,63 | 5,12 | -4,46 |
| 14,98 | 19,07 | -0,42 |
| 14,89 | 10,13 | -8,55 |
| 15,47 | 7,36 | -11,78 |
| 9,76 | 10,93 | -1,38 |
| 29,06 | 17,06 | -9,43 |
| 21,26 | 5,31 | -16,48 |
| 3,17 | 1,98 | -1,30 |
| 27,94 | 21,61 | -2,53 |
| 24,91 | 27,28 | -5,79 |
| 17,75 | 22,59 | -4,66 |
| 26,96 | 8,66 | -8,50 |
| 15,33 | 10,73 | -1,50 |
| 34,06 | 25,25 | -13,21 |
| 8,07 | 10,39 | -3,27 |
| 8,39 | 4,68 | -2,21 |
| 34,64 | 16,46 | -16,69 |
| 10,17 | 4,59 | -8,17 |

| | | |
|---|---|---|
| 5,99 | 4,62 | -0,97 |
| 17,46 | 5,66 | -8,08 |
| 25,81 | 11,09 | -11,76 |
| 36,88 | 24,64 | -9,24 |
| 4,11 | 3,12 | 27,09 |
| 33,99 | 27,78 | -31,15 |
| 15,28 | 5,37 | -9,23 |
| NAN | NAN | NAN |
| 10,91 | 4,95 | -5,12 |
| 5,06 | 7,03 | 0,80 |
| 15,29 | 2,09 | -7,71 |
| 5,06 | 10,69 | -0,82 |
| 22,47 | 14,55 | -4,56 |
| 6,45 | 1,00 | -0,99 |
| 11,09 | 6,02 | -3,40 |
| NAN | NAN | NAN |
| 14,38 | 2,99 | -3,10 |
| 7,23 | 3,03 | -1,08 |
| 19,32 | 16,14 | -5,23 |
| 13,95 | 10,64 | -6,05 |
| 11,81 | 22,58 | -0,54 |
| 7,68 | 5,89 | -0,86 |
| 19,48 | 16,39 | -6,02 |
| 5,01 | 3,64 | -2,66 |
| 49,38 | 42,69 | -20,24 |
| 13,89 | 16,23 | 5,75 |
| 17,66 | 1,87 | -5,62 |
| 41,19 | 51,92 | -20,52 |
| 3,92 | 2,62 | -1,05 |
| 33,03 | 15,95 | -19,62 |
| 5,27 | 1,74 | -2,89 |
| 13,43 | 13,96 | -7,50 |
| 20,72 | 12,01 | -9,71 |
| 21,38 | 10,31 | -11,48 |
| 16,84 | 13,51 | -7,97 |
| 7,65 | 6,32 | -3,96 |
| 7,15 | 8,24 | -2,23 |
| 12,26 | 9,85 | -5,45 |
| 12,51 | 7,54 | -6,39 |
| 6,65 | 2,90 | -3,92 |
| 7,28 | 3,68 | -2,52 |
| 10,55 | 9,89 | -3,64 |
| 15,61 | 12,71 | -1,96 |
| 4,85 | 2,17 | -2,88 |
| 7,57 | 3,57 | -5,10 |
| NAN | NAN | NAN |
| 7,41 | 2,38 | 1,07 |
| 8,92 | 2,80 | -3,10 |
| 10,82 | 5,80 | -1,33 |
| 13,42 | 3,27 | -3,04 |

| | | |
|---|---|---|
| 1,68 | 2,18 | 2,29 |
| 3,70 | 1,25 | -0,80 |
| 32,95 | 10,30 | -31,37 |
| 7,69 | 2,88 | -2,91 |
| 24,23 | 7,74 | -15,48 |
| 23,03 | 7,93 | -12,25 |
| 9,65 | 4,88 | -3,18 |
| 11,60 | 6,82 | -5,47 |
| 10,35 | 5,54 | -3,05 |
| NAN | NAN | NAN |
| 11,86 | 5,95 | -5,51 |
| 6,22 | 6,21 | -3,82 |
| 2,43 | 0,89 | -1,12 |
| 5,74 | 1,31 | -2,50 |
| 17,16 | 12,49 | -4,57 |
| 7,56 | 2,63 | -3,91 |
| NAN | NAN | NAN |
| 16,68 | 1,13 | -11,24 |
| 17,01 | 3,56 | -6,22 |
| 0,79 | 0,46 | -0,34 |
| 15,10 | 9,13 | -7,55 |
| 13,89 | 6,50 | -1,96 |
| 8,23 | 2,69 | -1,81 |
| 25,88 | 5,07 | -10,68 |
| 16,16 | 8,61 | -6,89 |
| 8,77 | 3,53 | -2,86 |
| 3,68 | 0,55 | -2,23 |
| 17,07 | 7,24 | -1,56 |
| 30,66 | 23,74 | -17,06 |
| 3,99 | 2,58 | -1,27 |
| 24,27 | 23,71 | 0,99 |
| 27,21 | 19,84 | -20,00 |
| 8,76 | 6,00 | -2,39 |
| 9,74 | 7,81 | -4,78 |
| 23,40 | 18,94 | -10,26 |
| 9,47 | 4,77 | -5,20 |
| 29,81 | 23,96 | -8,94 |
| 19,88 | 26,83 | -11,14 |
| 13,71 | 8,12 | -1,57 |
| 17,27 | 9,64 | -5,91 |
| 13,05 | 9,61 | -3,14 |
| 14,56 | 17,93 | -1,89 |
| 10,64 | 19,32 | -0,46 |
| 11,52 | 6,39 | -5,17 |
| 13,45 | 4,72 | -8,88 |
| 15,28 | 10,46 | -8,22 |
| 10,02 | 7,85 | -4,55 |
| 7,50 | 3,36 | -5,83 |
| 14,73 | 8,53 | -8,41 |
| 9,99 | 9,16 | -2,01 |

| | | |
|---|---|---|
| 14,54 | 6,83 | -7,59 |
| 22,37 | 13,69 | -12,32 |
| 10,68 | 2,82 | -7,82 |
| 7,89 | 3,12 | -5,72 |
| 23,88 | 17,93 | -16,08 |
| 8,14 | 2,05 | -3,67 |
| 5,06 | 1,12 | -2,46 |
| 4,09 | 3,56 | -3,07 |
| 17,49 | 10,10 | -9,72 |
| 5,10 | 2,97 | -3,86 |
| 3,51 | 6,73 | -0,84 |
| 7,31 | 4,83 | -5,25 |
| 6,06 | 3,96 | -4,19 |
| 13,03 | 13,97 | -8,27 |
| 6,85 | 5,09 | -5,15 |
| 10,81 | 9,57 | -6,49 |
| 11,43 | 5,82 | -7,85 |
| 6,77 | 7,17 | 0,86 |
| 16,09 | 10,28 | -0,58 |
| 6,59 | 8,12 | -0,18 |
| 4,68 | 3,33 | 0,25 |
| 3,83 | 1,14 | 0,21 |
| 9999,00 | 7,45 | NAN |
| 3,13 | 4,57 | 3,20 |
| 19,08 | 9,42 | -2,68 |
| 7,03 | 4,19 | -1,85 |
| 2,45 | 0,67 | 0,13 |
| 1,69 | 2,07 | -0,58 |
| 11,36 | 10,75 | 1,39 |
| 9,48 | 5,10 | -2,24 |
| 9,76 | 5,02 | -4,20 |
| 9,26 | 3,18 | -3,74 |
| 5,83 | 4,50 | 2,44 |
| 10,65 | 5,47 | -5,06 |
| 7,42 | 8,31 | 8,48 |
| 10,12 | 5,53 | -1,36 |
| 11,54 | 9,74 | -5,14 |
| 8,79 | 11,80 | -3,06 |
| 28,49 | 10,08 | -3,40 |
| 14,13 | 13,29 | 1,14 |
| 4,86 | 3,01 | 0,47 |
| 19,26 | 11,37 | -2,13 |
| NAN | NAN | NAN |
| NAN | NAN | NAN |
| NAN | NAN | NAN |
| NAN | NAN | NAN |
| NAN | NAN | NAN |
| NAN | NAN | NAN |
| NAN | NAN | NAN |
| NAN | NAN | NAN |

| | | |
|---|---|---|
| NAN | NAN | NAN |
| NAN | NAN | NAN |
| NAN | NAN | NAN |
| NAN | NAN | NAN |
| NAN | NAN | NAN |
| 14,68 | 8,27 | -6,18 |
| NAN | NAN | NAN |
| 6,25 | 15,36 | 3,76 |
| 14,84 | 7,73 | -0,02 |
| NAN | NAN | NAN |
| 3,73 | 3,16 | -0,18 |
| NAN | NAN | NAN |
| NAN | NAN | NAN |
| 19,97 | 13,41 | -2,15 |
| NAN | NAN | NAN |
| 25,47 | 17,13 | -14,63 |
| 6,49 | 14,56 | 2,36 |
| NAN | NAN | NAN |
| NAN | NAN | NAN |
| NAN | NAN | NAN |
| NAN | NAN | NAN |
| 7,59 | 3,31 | -2,49 |
| 11,40 | 9,20 | -4,10 |
| 3,42 | 1,35 | -2,38 |
| 13,41 | 18,22 | 0,63 |
| 10,92 | 15,55 | 0,49 |
| 13,57 | 9,51 | -6,53 |
| 14,65 | 20,85 | 8,24 |
| 12,59 | 9,96 | -2,64 |
| 14,80 | 7,21 | -9,32 |
| 7,85 | 6,94 | -4,78 |
| 9,71 | 4,16 | -4,09 |
| 9,17 | 5,59 | -4,02 |
| 1,78 | 1,94 | -0,47 |
| 6,99 | 8,86 | 0,90 |
| 16,78 | 11,93 | -9,32 |
| 4,48 | 2,42 | -2,06 |
| 6,53 | 6,08 | -2,63 |
| 2,54 | 8,29 | -1,02 |
| 8,74 | 9,18 | 2,06 |
| 7,82 | 3,73 | 0,52 |
| 9,53 | 4,67 | -4,24 |
| NAN | NAN | NAN |
| 19,08 | 3,15 | -8,67 |
| 8,03 | 5,77 | -1,55 |
| 7,05 | 4,59 | -6,03 |
| 26,99 | 17,64 | -17,95 |
| 10,72 | 12,59 | -3,24 |
| 7,89 | 1,88 | -5,85 |
| NAN | NAN | NAN |

| | | |
|---|---|---|
| 7,92 | 7,89 | -5,74 |
| 14,20 | 12,00 | -10,23 |
| 18,35 | 13,17 | -8,93 |
| 6,75 | 5,93 | -5,29 |
| NAN | NAN | NAN |
| 8,03 | 1,62 | -4,84 |
| 3,88 | 13,46 | -1,09 |
| 2,27 | 2,10 | -1,61 |
| 5,18 | 2,21 | -2,33 |
| 23,82 | 30,28 | -14,79 |
| NAN | NAN | NAN |
| 12,50 | 12,60 | -8,76 |
| 9,16 | 2,97 | -7,95 |
| 6,99 | 6,82 | -5,12 |
| 7,76 | 3,47 | -5,03 |
| NAN | NAN | NAN |
| NAN | NAN | NAN |
| NAN | NAN | NAN |
| NAN | NAN | NAN |
| NAN | NAN | NAN |
| NAN | NAN | NAN |
| NAN | NAN | NAN |
| NAN | NAN | NAN |
| 21,71 | 17,76 | -9,04 |
| 9,15 | 10,09 | -3,91 |
| 8,18 | 8,71 | 3,38 |
| 25,16 | 20,11 | -0,88 |
| NAN | NAN | NAN |
| NAN | NAN | NAN |
| 26,75 | 21,28 | -15,40 |
| 8,58 | 1,56 | -5,20 |
| 23,95 | 18,06 | -5,93 |
| NAN | NAN | NAN |
| NAN | NAN | NAN |
| 9,35 | 4,84 | 0,78 |
| 5,41 | 3,81 | 4,26 |
| 2,45 | 13,87 | 5,22 |
| 1,39 | 3,84 | 0,44 |
| 7,88 | 15,63 | 8,87 |
| 8,27 | 15,83 | 7,87 |
| NAN | NAN | NAN |
| 5,19 | 4,95 | 7,42 |
| 13,83 | 7,62 | 0,09 |
| 6,10 | 6,12 | 1,85 |
| 8,01 | 3,28 | 4,09 |
| 2,77 | 1,59 | 0,21 |
| NAN | NAN | NAN |
| NAN | NAN | NAN |
| NAN | NAN | NAN |
| NAN | NAN | NAN |

| | | |
|---|---|---|
| 4,80 | 7,36 | 6,32 |
| 7,72 | 15,23 | 2,01 |
| 6,17 | 5,62 | 6,80 |
| 2,16 | 4,05 | -0,07 |
| 9,94 | 8,33 | -3,78 |
| 12,73 | 14,05 | 3,58 |
| 24,82 | 17,13 | -0,70 |
| 5,45 | 9,73 | 1,01 |
| 4,61 | 7,09 | 0,40 |
| 15,43 | 9,21 | -1,14 |
| 10,83 | 11,61 | 2,41 |
| 13,22 | 15,25 | -0,65 |
| NAN | NAN | NAN |
| NAN | NAN | NAN |
| NAN | NAN | NAN |
| NAN | NAN | NAN |

| Leptin increase 2 yrs PD | Leptin change 24 gw | Leptin change 2 yrs PD |
|---|---|---|
| 0,57 | -1,96 | 0,20 |
| 14,61 | 0,11 | 0,50 |
| 6,44 | -0,06 | 0,45 |
| 3,17 | 0,09 | 0,16 |
| -5,88 | -0,45 | -0,76 |
| -4,10 | -2,72 | -2,48 |
| -0,58 | -1,06 | -0,36 |
| 3,48 | -0,32 | 0,12 |
| 0,13 | -0,45 | 0,01 |
| -8,37 | -0,40 | -0,43 |
| 1,64 | -1,93 | 0,31 |
| 5,89 | -0,08 | 0,27 |
| -0,76 | -1,45 | -0,32 |
| -1,63 | -0,26 | -0,11 |
| 1,34 | 0,01 | 0,37 |
| -0,04 | -0,07 | -0,01 |
| 1,99 | -1,60 | 0,44 |
| 1,00 | -0,35 | 0,19 |
| -10,65 | -0,61 | -2,83 |
| 3,90 | 0,36 | 0,29 |
| -2,33 | -0,47 | -0,80 |
| -7,99 | -1,33 | -0,99 |
| -5,65 | -0,27 | -0,23 |
| 4,24 | -1,75 | 0,58 |
| 10,29 | -0,11 | 0,39 |
| -5,54 | -2,25 | -1,63 |
| -8,17 | -1,53 | -1,01 |
| -4,46 | -0,62 | -0,28 |
| 2,50 | -1,12 | 0,66 |
| 1,05 | -0,72 | 0,17 |
| -4,51 | -0,03 | -0,31 |
| -3,79 | -1,35 | -0,60 |
| -3,67 | -3,19 | -0,99 |
| -2,55 | -0,16 | -0,30 |
| 2,57 | -0,48 | 0,13 |
| -0,53 | -3,45 | -0,11 |
| -0,11 | -0,70 | -0,06 |
| 3,80 | -0,10 | 0,15 |
| -8,16 | -0,30 | -0,43 |
| -9,50 | -0,36 | -0,73 |
| 9,80 | -0,46 | 0,53 |
| 3,10 | -0,11 | 0,22 |
| -4,40 | -0,63 | -0,21 |
| -5,59 | -0,68 | -1,16 |
| 1,50 | -0,36 | 0,24 |
| 1,49 | -0,93 | 0,08 |
| -2,59 | -4,09 | -1,30 |

| | | |
|---|---|---|
| 0,40 | -0,19 | 0,08 |
| 3,72 | -0,86 | 0,40 |
| 2,96 | -0,84 | 0,21 |
| 3,00 | -0,33 | 0,11 |
| 28,08 | 0,87 | 0,90 |
| -24,94 | -10,97 | -8,78 |
| 0,68 | -1,53 | 0,11 |
| NAN | NAN | NAN |
| 0,84 | -0,88 | 0,15 |
| -1,17 | 0,14 | -0,20 |
| 5,49 | -1,02 | 0,72 |
| -6,45 | -0,19 | -1,52 |
| 3,36 | -0,25 | 0,19 |
| 4,46 | -0,18 | 0,82 |
| 1,67 | -0,44 | 0,22 |
| NAN | NAN | NAN |
| 8,29 | -0,27 | 0,73 |
| 3,12 | -0,18 | 0,51 |
| -2,05 | -0,37 | -0,15 |
| -2,74 | -0,77 | -0,35 |
| -11,31 | -0,05 | -1,00 |
| 0,93 | -0,13 | 0,14 |
| -2,93 | -0,45 | -0,22 |
| -1,29 | -1,13 | -0,55 |
| -13,55 | -0,69 | -0,46 |
| 3,41 | 0,29 | 0,17 |
| 10,17 | -0,47 | 0,84 |
| -31,25 | -0,99 | -1,51 |
| 0,25 | -0,37 | 0,09 |
| -2,54 | -1,46 | -0,19 |
| 0,64 | -1,21 | 0,27 |
| -8,03 | -1,26 | -1,35 |
| -1,00 | -0,88 | -0,09 |
| -0,41 | -1,16 | -0,04 |
| -4,64 | -0,90 | -0,52 |
| -2,63 | -1,07 | -0,71 |
| -3,32 | -0,45 | -0,67 |
| -3,04 | -0,80 | -0,45 |
| -1,42 | -1,04 | -0,23 |
| -0,17 | -1,44 | -0,06 |
| 1,08 | -0,53 | 0,23 |
| -2,98 | -0,53 | -0,43 |
| 0,94 | -0,14 | 0,07 |
| -0,20 | -1,46 | -0,10 |
| -1,10 | -2,06 | -0,45 |
| NAN | NAN | NAN |
| 6,10 | 0,13 | 0,72 |
| 3,02 | -0,53 | 0,52 |
| 3,69 | -0,14 | 0,39 |
| 7,11 | -0,29 | 0,68 |

| | | |
|---|---|---|
| 1,79 | 0,58 | 0,45 |
| 1,65 | -0,28 | 0,57 |
| -8,72 | -19,85 | -5,52 |
| 1,90 | -0,61 | 0,40 |
| 1,01 | -1,77 | 0,12 |
| 2,85 | -1,14 | 0,26 |
| 1,59 | -0,49 | 0,25 |
| -0,69 | -0,89 | -0,11 |
| 1,76 | -0,42 | 0,24 |
| NAN | NAN | NAN |
| 0,40 | -0,87 | 0,06 |
| -3,81 | -1,59 | -1,59 |
| 0,42 | -0,85 | 0,32 |
| 1,93 | -0,77 | 0,60 |
| 0,10 | -0,36 | 0,01 |
| 1,02 | -1,07 | 0,28 |
| NAN | NAN | NAN |
| 4,31 | -2,07 | 0,79 |
| 7,23 | -0,58 | 0,67 |
| -0,01 | -0,76 | -0,02 |
| -1,58 | -1,00 | -0,21 |
| 5,43 | -0,16 | 0,46 |
| 3,73 | -0,28 | 0,58 |
| 10,13 | -0,70 | 0,67 |
| 0,66 | -0,74 | 0,07 |
| 2,38 | -0,48 | 0,40 |
| 0,90 | -1,54 | 0,62 |
| 8,27 | -0,10 | 0,53 |
| -10,14 | -1,25 | -0,75 |
| 0,14 | -0,47 | 0,05 |
| 1,55 | 0,04 | 0,06 |
| -12,63 | -2,77 | -1,75 |
| 0,37 | -0,38 | 0,06 |
| -2,85 | -0,96 | -0,57 |
| -5,80 | -0,78 | -0,44 |
| -0,50 | -1,22 | -0,12 |
| -3,09 | -0,43 | -0,15 |
| -18,09 | -1,27 | -2,07 |
| 4,02 | -0,13 | 0,33 |
| 1,72 | -0,52 | 0,15 |
| 0,30 | -0,32 | 0,03 |
| -5,26 | -0,15 | -0,42 |
| -9,14 | -0,05 | -0,90 |
| -0,04 | -0,81 | -0,01 |
| -0,15 | -1,94 | -0,03 |
| -3,40 | -1,16 | -0,48 |
| -2,38 | -0,83 | -0,44 |
| -1,69 | -3,49 | -1,01 |
| -2,21 | -1,33 | -0,35 |
| -1,18 | -0,25 | -0,15 |

| | | |
|---|---|---|
| 0,12 | -1,09 | 0,02 |
| -3,64 | -1,23 | -0,36 |
| 0,04 | -2,73 | 0,01 |
| -0,95 | -2,64 | -0,44 |
| -10,13 | -2,06 | -1,30 |
| 2,42 | -0,82 | 0,54 |
| 1,48 | -0,95 | 0,57 |
| -2,54 | -3,01 | -2,49 |
| -2,33 | -1,25 | -0,30 |
| -1,73 | -3,11 | -1,40 |
| -4,06 | -0,31 | -1,52 |
| -2,77 | -2,55 | -1,34 |
| -2,09 | -2,24 | -1,12 |
| -9,21 | -1,74 | -1,93 |
| -3,39 | -3,03 | -1,99 |
| -5,25 | -1,50 | -1,22 |
| -2,24 | -2,19 | -0,63 |
| 0,46 | 0,11 | 0,06 |
| 5,23 | -0,04 | 0,34 |
| -1,71 | -0,03 | -0,27 |
| 1,60 | 0,05 | 0,32 |
| 2,90 | 0,05 | 0,72 |
| -0,06 | NAN | -0,01 |
| 1,76 | 0,51 | 0,28 |
| 6,98 | -0,16 | 0,43 |
| 0,99 | -0,36 | 0,19 |
| 1,91 | 0,05 | 0,74 |
| -0,96 | -0,52 | -0,86 |
| 2,00 | 0,11 | 0,16 |
| 2,14 | -0,31 | 0,30 |
| 0,54 | -0,76 | 0,10 |
| 2,34 | -0,68 | 0,42 |
| 3,77 | 0,30 | 0,46 |
| 0,12 | -0,91 | 0,02 |
| 7,59 | 0,53 | 0,48 |
| 3,23 | -0,16 | 0,37 |
| -3,34 | -0,80 | -0,52 |
| -6,07 | -0,53 | -1,06 |
| 15,01 | -0,14 | 0,60 |
| 1,98 | 0,07 | 0,13 |
| 2,32 | 0,09 | 0,44 |
| 5,76 | -0,12 | 0,34 |
| NAN | NAN | NAN |
| NAN | NAN | NAN |
| NAN | NAN | NAN |
| NAN | NAN | NAN |
| NAN | NAN | NAN |
| NAN | NAN | NAN |
| NAN | NAN | NAN |
| NAN | NAN | NAN |

| | | |
|---|---|---|
| NAN | NAN | NAN |
| NAN | NAN | NAN |
| NAN | NAN | NAN |
| NAN | NAN | NAN |
| NAN | NAN | NAN |
| 0,23 | -0,73 | 0,03 |
| NAN | NAN | NAN |
| -5,35 | 0,38 | -0,53 |
| 7,09 | 0,00 | 0,48 |
| NAN | NAN | NAN |
| 0,39 | -0,05 | 0,11 |
| NAN | NAN | NAN |
| NAN | NAN | NAN |
| 4,41 | -0,12 | 0,25 |
| NAN | NAN | NAN |
| -6,29 | -1,35 | -0,58 |
| -5,71 | 0,27 | -0,65 |
| NAN | NAN | NAN |
| NAN | NAN | NAN |
| NAN | NAN | NAN |
| NAN | NAN | NAN |
| 1,79 | -0,49 | 0,35 |
| -1,90 | -0,56 | -0,26 |
| -0,31 | -2,29 | -0,30 |
| -4,18 | 0,04 | -0,30 |
| -4,14 | 0,04 | -0,36 |
| -2,47 | -0,93 | -0,35 |
| 2,04 | 0,36 | 0,09 |
| -0,01 | -0,27 | 0,00 |
| -1,73 | -1,70 | -0,32 |
| -3,87 | -1,56 | -1,26 |
| 1,46 | -0,73 | 0,26 |
| -0,44 | -0,78 | -0,09 |
| -0,63 | -0,36 | -0,48 |
| -0,97 | 0,11 | -0,12 |
| -4,47 | -1,25 | -0,60 |
| 0,00 | -0,85 | 0,00 |
| -2,18 | -0,67 | -0,56 |
| -6,77 | -0,67 | -4,45 |
| 1,62 | 0,19 | 0,15 |
| 4,61 | 0,06 | 0,55 |
| 0,62 | -0,80 | 0,12 |
| NAN | NAN | NAN |
| 7,26 | -0,83 | 0,70 |
| 0,71 | -0,24 | 0,11 |
| -3,57 | -5,91 | -3,50 |
| -8,60 | -1,99 | -0,95 |
| -5,11 | -0,43 | -0,68 |
| 0,16 | -2,87 | 0,08 |
| NAN | NAN | NAN |

| | | |
|---|---|---|
| -5,71 | -2,63 | -2,62 |
| -8,03 | -2,58 | -2,02 |
| -3,75 | -0,95 | -0,40 |
| -4,47 | -3,62 | -3,06 |
| NAN | NAN | NAN |
| 1,57 | -1,52 | 0,49 |
| -10,67 | -0,39 | -3,82 |
| -1,44 | -2,44 | -2,18 |
| 0,64 | -0,82 | 0,22 |
| -21,25 | -1,64 | -2,35 |
| NAN | NAN | NAN |
| -8,86 | -2,34 | -2,37 |
| -1,76 | -6,57 | -1,45 |
| -4,95 | -2,74 | -2,65 |
| -0,74 | -1,84 | -0,27 |
| NAN | NAN | NAN |
| NAN | NAN | NAN |
| NAN | NAN | NAN |
| NAN | NAN | NAN |
| NAN | NAN | NAN |
| NAN | NAN | NAN |
| NAN | NAN | NAN |
| NAN | NAN | NAN |
| -5,09 | -0,71 | -0,40 |
| -4,85 | -0,75 | -0,93 |
| 2,85 | 0,29 | 0,25 |
| 4,17 | -0,04 | 0,17 |
| NAN | NAN | NAN |
| NAN | NAN | NAN |
| -9,93 | -1,36 | -0,87 |
| 1,82 | -1,54 | 0,54 |
| -0,04 | -0,33 | 0,00 |
| NAN | NAN | NAN |
| NAN | NAN | NAN |
| 5,29 | 0,08 | 0,52 |
| 5,86 | 0,44 | 0,61 |
| -6,20 | 0,68 | -0,81 |
| -2,01 | 0,24 | -1,10 |
| 1,12 | 0,53 | 0,07 |
| 0,31 | 0,49 | 0,02 |
| NAN | NAN | NAN |
| 7,66 | 0,59 | 0,61 |
| 6,30 | 0,01 | 0,45 |
| 1,83 | 0,23 | 0,23 |
| 8,82 | 0,34 | 0,73 |
| 1,39 | 0,07 | 0,47 |
| NAN | NAN | NAN |
| NAN | NAN | NAN |
| NAN | NAN | NAN |
| NAN | NAN | NAN |

| | | |
|---|---|---|
| 3,76 | 0,57 | 0,34 |
| -5,50 | 0,21 | -0,57 |
| 7,35 | 0,52 | 0,57 |
| -1,96 | -0,03 | -0,94 |
| -2,17 | -0,61 | -0,35 |
| 2,26 | 0,22 | 0,14 |
| 6,99 | -0,03 | 0,29 |
| -3,27 | 0,16 | -0,51 |
| -2,08 | 0,08 | -0,42 |
| 5,08 | -0,08 | 0,36 |
| 1,63 | 0,18 | 0,12 |
| -2,68 | -0,05 | -0,21 |
| NAN | NAN | NAN |
| NAN | NAN | NAN |
| NAN | NAN | NAN |
| NAN | NAN | NAN |

| TNF alpha (pg/mL) 24 gw | TNF alpha (pg/mL) 2 yrs PD | TNF alpha increase 24 gw | TNF alpha change 24 gw |
|---|---|---|---|
| 2,01 | 2,61 | 0,15 | 0,07 |
| 1,37 | 1,82 | 2,28 | 0,62 |
| 4,91 | 3,64 | -1,49 | -0,44 |
| 5,99 | 4,53 | -3,03 | -1,02 |
| 3,81 | 2,89 | -0,37 | -0,11 |
| 2,19 | 5,00 | -0,73 | -0,50 |
| 2,87 | 3,13 | -0,26 | -0,10 |
| 0,01 | 1,47 | 1,36 | 0,99 |
| 2,96 | 4,11 | 1,87 | 0,39 |
| 3,31 | 4,80 | -0,77 | -0,30 |
| 1,27 | 2,56 | -0,20 | -0,19 |
| 1,54 | 1,88 | 0,36 | 0,19 |
| 1,15 | 1,43 | -0,41 | -0,55 |
| 2,99 | 3,55 | -0,27 | -0,10 |
| 4,59 | 4,59 | -0,43 | -0,10 |
| 2,53 | 3,38 | -0,03 | -0,01 |
| 2,29 | 3,52 | 0,63 | 0,22 |
| 5,06 | 5,78 | -0,74 | -0,17 |
| 7,82 | 7,24 | 10,14 | 0,56 |
| 9,85 | 7,62 | 2,07 | 0,17 |
| 3,94 | 2,04 | 0,80 | 0,17 |
| 5,00 | 5,37 | 4,06 | 0,45 |
| 5,18 | 6,71 | -0,32 | -0,07 |
| 5,12 | 7,14 | 0,08 | 0,02 |
| 5,40 | 5,62 | -0,06 | -0,01 |
| 5,99 | 6,25 | -0,53 | -0,10 |
| 3,71 | 3,84 | 2,28 | 0,38 |
| 6,18 | 5,89 | 0,05 | 0,01 |
| 1,44 | 1,60 | -0,15 | -0,12 |
| 11,61 | 4,60 | 3,75 | 0,24 |
| 4,85 | 11,56 | 4,53 | 0,48 |
| 6,45 | 4,87 | 5,35 | 0,45 |
| 3,02 | 3,77 | 1,01 | 0,25 |
| 2,75 | 2,56 | 0,61 | 0,18 |
| 5,08 | 2,78 | -0,76 | -0,18 |
| 0,01 | 0,01 | 0,00 | 0,00 |
| 1,74 | 2,38 | 0,91 | 0,34 |
| 1,86 | 2,55 | -0,27 | -0,17 |
| 6,83 | 6,28 | -1,93 | -0,39 |
| 4,00 | 4,30 | -0,42 | -0,12 |
| 4,00 | 4,40 | 0,87 | 0,18 |
| 2,17 | 2,80 | 0,30 | 0,12 |
| 5,16 | 6,05 | 0,10 | 0,02 |
| 3,00 | 2,21 | 1,35 | 0,31 |
| 2,84 | 2,79 | -0,43 | -0,18 |
| 4,03 | 4,40 | -0,40 | -0,11 |
| 4,24 | 2,98 | 0,34 | 0,07 |

| | | | |
|---|---|---|---|
| 1,68 | 3,94 | 0,73 | 0,30 |
| 3,25 | 3,67 | -0,20 | -0,07 |
| 4,33 | 3,76 | -0,57 | -0,15 |
| 4,73 | 4,39 | -0,25 | -0,06 |
| 1,49 | 1,98 | 1,45 | 0,49 |
| 1,98 | 2,82 | 1,25 | 0,39 |
| 3,78 | 5,26 | -0,08 | -0,02 |
| NAN | NAN | NAN | NAN |
| 1,67 | 1,97 | 0,11 | 0,06 |
| 1,78 | 3,81 | 0,70 | 0,28 |
| 1,98 | 2,68 | 0,21 | 0,10 |
| 2,17 | 3,50 | -0,05 | -0,02 |
| 4,13 | 3,70 | -1,42 | -0,52 |
| 2,74 | 2,77 | 0,16 | 0,06 |
| 3,41 | 4,20 | 0,02 | 0,01 |
| NAN | NAN | NAN | NAN |
| 2,54 | 1,77 | -0,22 | -0,09 |
| 4,76 | 6,20 | -0,41 | -0,09 |
| 5,17 | 7,20 | -0,03 | -0,01 |
| 3,75 | 2,22 | 0,79 | 0,17 |
| 2,17 | 4,20 | 0,24 | 0,10 |
| 5,06 | 5,05 | -0,56 | -0,12 |
| 2,59 | 3,56 | 0,29 | 0,10 |
| 4,88 | 7,66 | -0,38 | -0,08 |
| 3,81 | 3,92 | 0,16 | 0,04 |
| 2,88 | 4,12 | -0,27 | -0,10 |
| 0,96 | 1,40 | 0,07 | 0,07 |
| 2,45 | 4,56 | 1,63 | 0,40 |
| 1,99 | 1,65 | 0,25 | 0,11 |
| 4,65 | 6,46 | -2,17 | -0,88 |
| 3,50 | 3,40 | -0,65 | -0,23 |
| 4,28 | 4,17 | -1,06 | -0,33 |
| 3,10 | 3,62 | -0,43 | -0,16 |
| 2,49 | 2,41 | -0,48 | -0,24 |
| 6,35 | 5,36 | -1,72 | -0,37 |
| 2,47 | 2,43 | 0,48 | 0,16 |
| 2,01 | 2,26 | -0,23 | -0,13 |
| 3,93 | 3,25 | -1,06 | -0,37 |
| 3,75 | 3,43 | -0,48 | -0,15 |
| 1,88 | 2,75 | -0,17 | -0,10 |
| 0,70 | 0,01 | 0,01 | 0,01 |
| 2,66 | 2,85 | -0,24 | -0,10 |
| 5,00 | 5,08 | -0,39 | -0,08 |
| 2,23 | 2,88 | -0,11 | -0,05 |
| 1,41 | 0,81 | -0,48 | -0,52 |
| NAN | NAN | NAN | NAN |
| 0,70 | 3,46 | 0,03 | 0,04 |
| 1,81 | 1,67 | -0,14 | -0,08 |
| 2,94 | 2,12 | -0,05 | -0,02 |
| 2,37 | 2,12 | -0,17 | -0,08 |

| | | | |
|---|---|---|---|
| 1,54 | 2,85 | 1,39 | 0,47 |
| 1,02 | 2,05 | 0,12 | 0,11 |
| 1,85 | 1,63 | 0,36 | 0,16 |
| 3,37 | 2,08 | -1,54 | -0,84 |
| 1,97 | 2,49 | 0,11 | 0,05 |
| 0,01 | 0,82 | 0,76 | 0,99 |
| 1,67 | 1,76 | 0,42 | 0,20 |
| 1,94 | 2,25 | 0,27 | 0,12 |
| 3,58 | 4,28 | -0,29 | -0,09 |
| NAN | NAN | NAN | NAN |
| 1,21 | 2,03 | 0,53 | 0,30 |
| 1,67 | 2,68 | -0,22 | -0,15 |
| 0,01 | 0,01 | 0,76 | 0,99 |
| 283,00 | 1,92 | -280,43 | -109,12 |
| 1,77 | 2,57 | 0,57 | 0,24 |
| 1,76 | 1,60 | 0,75 | 0,30 |
| NAN | NAN | NAN | NAN |
| 6,28 | 6,88 | 10,33 | 0,62 |
| 3,44 | 3,47 | 0,92 | 0,21 |
| 0,81 | 0,83 | 1,06 | 0,57 |
| 35,81 | 59,20 | -3,08 | -0,09 |
| 2,83 | 1,64 | 4,07 | 0,59 |
| 2,57 | 2,39 | 0,54 | 0,17 |
| 4,31 | 4,28 | -0,29 | -0,07 |
| 4,63 | 4,68 | 0,68 | 0,13 |
| 9,79 | 8,15 | -0,88 | -0,10 |
| 0,90 | 1,76 | 1,04 | 0,54 |
| 2,29 | 2,59 | -0,17 | -0,08 |
| 4,47 | 5,46 | -0,19 | -0,04 |
| 7,22 | 7,44 | -1,45 | -0,25 |
| 6,41 | 6,22 | 0,41 | 0,06 |
| 7,95 | 8,77 | 0,55 | 0,06 |
| 5,18 | 6,11 | -0,75 | -0,17 |
| 3,29 | 3,66 | -0,56 | -0,21 |
| 7,31 | 6,03 | -1,65 | -0,29 |
| 2,02 | 2,92 | -0,24 | -0,13 |
| 6,25 | 5,66 | -0,76 | -0,14 |
| 4,52 | 3,66 | -0,71 | -0,19 |
| 1,64 | 1,66 | 0,19 | 0,10 |
| 5,52 | 4,95 | 0,03 | 0,01 |
| 4,23 | 4,02 | 0,55 | 0,12 |
| 2,49 | 2,05 | -0,70 | -0,39 |
| 2,86 | 2,54 | 0,50 | 0,15 |
| 2,67 | 1,88 | -1,06 | -0,66 |
| 2,07 | 1,83 | -0,33 | -0,19 |
| 4,37 | 4,46 | -1,04 | -0,31 |
| 1,92 | 4,26 | 0,70 | 0,27 |
| 1,91 | 3,13 | -0,80 | -0,72 |
| 3,32 | 3,52 | -1,14 | -0,52 |
| 3,46 | 3,78 | 0,70 | 0,17 |

| | | | |
|---|---|---|---|
| 1,66 | 1,70 | 0,17 | 0,09 |
| 4,81 | 1,83 | -0,11 | -0,02 |
| 2,77 | 2,72 | -1,16 | -0,72 |
| 2,72 | 2,33 | -1,49 | -1,21 |
| 3,03 | 3,30 | 2,54 | 0,46 |
| 3,32 | 4,22 | 3,86 | 0,54 |
| 3,67 | 2,64 | -0,03 | -0,01 |
| 0,76 | 1,98 | 0,84 | 0,53 |
| 2,30 | 2,62 | -0,25 | -0,12 |
| 0,81 | 1,34 | 0,15 | 0,16 |
| 1,22 | 1,71 | -0,60 | -0,97 |
| 1,07 | 1,51 | 0,31 | 0,22 |
| 1,22 | 1,28 | -0,11 | -0,10 |
| 1,55 | 18,68 | 0,33 | 0,18 |
| 0,84 | 1,08 | -0,26 | -0,45 |
| 0,84 | 1,41 | -0,30 | -0,56 |
| 2,79 | 2,53 | -0,24 | -0,09 |
| 1,26 | 1,69 | -0,09 | -0,08 |
| 3,14 | 3,63 | 1,18 | 0,27 |
| 3,52 | 5,23 | -0,38 | -0,12 |
| 2,28 | 2,43 | -0,06 | -0,03 |
| 2,90 | 3,48 | -0,47 | -0,19 |
| 1,16 | 1,84 | 0,72 | 0,38 |
| 2,55 | 3,08 | -0,75 | -0,42 |
| 3,90 | 3,34 | -0,64 | -0,20 |
| 1,20 | 1,78 | 0,34 | 0,22 |
| 1,20 | 2,26 | 0,39 | 0,25 |
| 2,71 | 3,59 | 0,51 | 0,16 |
| 1,50 | 1,33 | -0,30 | -0,25 |
| 3,14 | 2,46 | 0,16 | 0,05 |
| 2,30 | 2,91 | 0,32 | 0,12 |
| 3,79 | 4,51 | 0,23 | 0,06 |
| 3,73 | 5,11 | -0,24 | -0,07 |
| 0,01 | 0,01 | 0,00 | 0,00 |
| 2,88 | 3,09 | 2,72 | 0,49 |
| 3,37 | 3,81 | -0,19 | -0,06 |
| 3,88 | 3,83 | -2,28 | -1,43 |
| 0,01 | 0,01 | 0,00 | 0,00 |
| 3,33 | 2,84 | -0,95 | -0,40 |
| 2,99 | 6,12 | 3,44 | 0,53 |
| 0,80 | 0,83 | 0,80 | 0,50 |
| 2,33 | 2,62 | 0,02 | 0,01 |
| NAN | NAN | NAN | NAN |
| NAN | NAN | NAN | NAN |
| NAN | NAN | NAN | NAN |
| NAN | NAN | NAN | NAN |
| NAN | NAN | NAN | NAN |
| NAN | NAN | NAN | NAN |
| NAN | NAN | NAN | NAN |
| NAN | NAN | NAN | NAN |

| | | | |
|---|---|---|---|
| NAN | NAN | NAN | NAN |
| NAN | NAN | NAN | NAN |
| NAN | NAN | NAN | NAN |
| NAN | NAN | NAN | NAN |
| NAN | NAN | NAN | NAN |
| 2,82 | 2,08 | -0,39 | -0,16 |
| NAN | NAN | NAN | NAN |
| 1,37 | 2,36 | -0,08 | -0,06 |
| 4,79 | 4,47 | -1,00 | -0,26 |
| NAN | NAN | NAN | NAN |
| 2,19 | 1,26 | -0,74 | -0,51 |
| NAN | NAN | NAN | NAN |
| NAN | NAN | NAN | NAN |
| 1,79 | 1,99 | 0,06 | 0,03 |
| NAN | NAN | NAN | NAN |
| 7,90 | 9,09 | 0,54 | 0,06 |
| 1,56 | 2,00 | 0,16 | 0,09 |
| NAN | NAN | NAN | NAN |
| NAN | NAN | NAN | NAN |
| NAN | NAN | NAN | NAN |
| NAN | NAN | NAN | NAN |
| 2,93 | 3,77 | -0,41 | -0,16 |
| 2,67 | 2,31 | -0,75 | -0,39 |
| 2,74 | 2,75 | 0,15 | 0,05 |
| 2,03 | 5,22 | -0,04 | -0,02 |
| 1,33 | 1,49 | -0,20 | -0,18 |
| 4,06 | 4,64 | -0,03 | -0,01 |
| 3,60 | 4,62 | 0,20 | 0,05 |
| 3,44 | 4,16 | -1,01 | -0,42 |
| 4,26 | 3,34 | -0,26 | -0,06 |
| 5,52 | 5,81 | 0,69 | 0,11 |
| 4,96 | 4,14 | -1,77 | -0,55 |
| 3,43 | 4,72 | -0,44 | -0,15 |
| 3,10 | 3,66 | -1,16 | -0,60 |
| 3,07 | 2,43 | -0,13 | -0,04 |
| 2,27 | 2,29 | 0,02 | 0,01 |
| 2,76 | 2,89 | -0,56 | -0,25 |
| 2,04 | 2,20 | -0,05 | -0,03 |
| 1,90 | 1,64 | 0,19 | 0,09 |
| 4,10 | 5,17 | -0,04 | -0,01 |
| 4,14 | 4,03 | -0,81 | -0,24 |
| 3,52 | 4,05 | -0,43 | -0,14 |
| NAN | NAN | NAN | NAN |
| 1,31 | 1,73 | 0,26 | 0,17 |
| 2,21 | 2,17 | 0,06 | 0,03 |
| 2,50 | 3,20 | -0,20 | -0,09 |
| 2,41 | 1,72 | -1,23 | -1,04 |
| 4,39 | 4,88 | -1,08 | -0,33 |
| 4,79 | 3,69 | -2,29 | -0,92 |
| NAN | NAN | NAN | NAN |

| | | | |
|---|---|---|---|
| 3,54 | 8,36 | -1,54 | -0,77 |
| 3,11 | 4,13 | -0,90 | -0,41 |
| 2,65 | 3,50 | -0,57 | -0,27 |
| 1,91 | 2,00 | -0,07 | -0,04 |
| NAN | NAN | NAN | NAN |
| 1,91 | 1,69 | -0,51 | -0,36 |
| 3,61 | 1,58 | -1,37 | -0,61 |
| 1,30 | 1,07 | -0,12 | -0,10 |
| 3,79 | 6,39 | -1,06 | -0,39 |
| 0,22 | 0,22 | 0,00 | 0,00 |
| NAN | NAN | NAN | NAN |
| 0,92 | 1,24 | -0,70 | -3,18 |
| 2,46 | 4,44 | -2,45 | -245,00 |
| 1,12 | 1,42 | -0,90 | -4,09 |
| 4,13 | 2,81 | -3,05 | -2,82 |
| NAN | NAN | NAN | NAN |
| NAN | NAN | NAN | NAN |
| NAN | NAN | NAN | NAN |
| NAN | NAN | NAN | NAN |
| NAN | NAN | NAN | NAN |
| NAN | NAN | NAN | NAN |
| NAN | NAN | NAN | NAN |
| NAN | NAN | NAN | NAN |
| 2,66 | 2,31 | -0,83 | -0,45 |
| 3,18 | 7,59 | 0,19 | 0,06 |
| 5,10 | 4,02 | -0,54 | -0,12 |
| 7,60 | 8,62 | -0,10 | -0,01 |
| NAN | NAN | NAN | NAN |
| NAN | NAN | NAN | NAN |
| 3,22 | 3,36 | -0,11 | -0,04 |
| 1,97 | 269,00 | 0,28 | 0,12 |
| 4,37 | 4,31 | -0,53 | -0,14 |
| NAN | NAN | NAN | NAN |
| NAN | NAN | NAN | NAN |
| 2,20 | 2,77 | -0,02 | -0,01 |
| 1,41 | 1,64 | 0,27 | 0,16 |
| 2,30 | 2,52 | 2,93 | 0,56 |
| 2,16 | 2,83 | 0,36 | 0,14 |
| 1,84 | 1,11 | 0,44 | 0,19 |
| 114,00 | 2,28 | -112,28 | -65,28 |
| NAN | NAN | NAN | NAN |
| 1,63 | 1,53 | 0,06 | 0,04 |
| 1,58 | 1,96 | 0,36 | 0,19 |
| 1,16 | 1,98 | 0,20 | 0,15 |
| 1,87 | 2,16 | 0,12 | 0,06 |
| 2,28 | 3,52 | 0,22 | 0,09 |
| NAN | NAN | NAN | NAN |
| NAN | NAN | NAN | NAN |
| NAN | NAN | NAN | NAN |
| NAN | NAN | NAN | NAN |

| | | | |
|---|---|---|---|
| 1,80 | 2,66 | 0,27 | 0,13 |
| 2,66 | 5,09 | 0,59 | 0,18 |
| 0,93 | 1,57 | 0,18 | 0,16 |
| 0,83 | 1,59 | 0,94 | 0,53 |
| 1,55 | 2,36 | 0,48 | 0,24 |
| 2,19 | 2,64 | -0,05 | -0,02 |
| 2,69 | 0,96 | -0,46 | -0,21 |
| 1,30 | 1,79 | 0,07 | 0,05 |
| 2,34 | 2,99 | 0,13 | 0,05 |
| 2,68 | 3,58 | 0,40 | 0,13 |
| 2,10 | 2,40 | 0,02 | 0,01 |
| 2,68 | 3,33 | 0,70 | 0,21 |
| NAN | NAN | NAN | NAN |
| NAN | NAN | NAN | NAN |
| NAN | NAN | NAN | NAN |
| NAN | NAN | NAN | NAN |

| TNF alpha increase 2 yrs PD | TNF alpha change 2 yrs PD | BMI (Kg/m2) 2 yrs PD | WC (cm) 2 yrs PD |
|---|---|---|---|
| -0,45 | -0,21 | 19,80 | 65,00 |
| 1,83 | 0,50 | 22,68 | 82,00 |
| -0,22 | -0,06 | 22,85 | 79,00 |
| -1,57 | -0,53 | 31,60 | 99,00 |
| 0,55 | 0,16 | 22,57 | 80,00 |
| -3,54 | -2,42 | 17,80 | 67,50 |
| -0,52 | -0,20 | NAN | NAN |
| -0,10 | -0,07 | 30,03 | NAN |
| 0,72 | 0,15 | 26,23 | 80,00 |
| -2,26 | -0,89 | 38,60 | 105,00 |
| -1,49 | -1,39 | 19,60 | 66,00 |
| 0,02 | 0,01 | 30,60 | 92,00 |
| -0,69 | -0,93 | 21,79 | 76,50 |
| -0,83 | -0,31 | 35,70 | 117,00 |
| -0,43 | -0,10 | 18,49 | 61,00 |
| -0,88 | -0,35 | 21,04 | 69,00 |
| -0,60 | -0,21 | 19,60 | 63,00 |
| -1,46 | -0,34 | 21,30 | 69,00 |
| 10,72 | 0,60 | 24,34 | 85,00 |
| 4,30 | 0,36 | 23,20 | 90,00 |
| 2,70 | 0,57 | 20,43 | 66,00 |
| 3,69 | 0,41 | 30,00 | 103,00 |
| -1,85 | -0,38 | 31,30 | 89,00 |
| -1,94 | -0,37 | 20,21 | 77,00 |
| -0,28 | -0,05 | 31,62 | 100,00 |
| -0,79 | -0,14 | NAN | NAN |
| 2,15 | 0,36 | 23,50 | 80,00 |
| 0,34 | 0,05 | 28,51 | 91,00 |
| -0,31 | -0,24 | 18,70 | 63,00 |
| 10,76 | 0,70 | 21,66 | 83,00 |
| -2,18 | -0,23 | 27,57 | 98,00 |
| 6,93 | 0,59 | 20,34 | NAN |
| 0,26 | 0,06 | 26,80 | 84,00 |
| 0,80 | 0,24 | 27,92 | 86,00 |
| 1,54 | 0,36 | 22,00 | 72,00 |
| 0,00 | 0,00 | 24,04 | 81,00 |
| 0,27 | 0,10 | 19,60 | 68,00 |
| -0,96 | -0,60 | 32,10 | 93,00 |
| -1,38 | -0,28 | NAN | NAN |
| -0,72 | -0,20 | 25,01 | 82,00 |
| 0,47 | 0,10 | 25,51 | 78,00 |
| -0,33 | -0,13 | 26,61 | 91,00 |
| -0,79 | -0,15 | 23,13 | 77,00 |
| 2,14 | 0,49 | 21,90 | 84,00 |
| -0,38 | -0,16 | 21,53 | 77,50 |
| -0,77 | -0,21 | 26,00 | 88,00 |
| 1,60 | 0,35 | 19,31 | 66,00 |

| | | | |
|---|---|---|---|
| -1,53 | -0,63 | 23,37 | 80,00 |
| -0,62 | -0,20 | 24,52 | 82,00 |
| 0,00 | 0,00 | 23,55 | 77,50 |
| 0,09 | 0,02 | 30,20 | 103,00 |
| 0,96 | 0,33 | 18,67 | 68,00 |
| 0,41 | 0,13 | 24,20 | 73,00 |
| -1,56 | -0,42 | 23,71 | 82,00 |
| NAN | NAN | NAN | 77,00 |
| -0,19 | -0,11 | 22,80 | 76,00 |
| -1,33 | -0,54 | 25,19 | 79,00 |
| -0,49 | -0,22 | 23,87 | 73,00 |
| -1,38 | -0,65 | 25,05 | 74,00 |
| -0,99 | -0,37 | 26,72 | 84,00 |
| 0,13 | 0,04 | 21,60 | 79,00 |
| -0,77 | -0,22 | 25,86 | 89,00 |
| NAN | NAN | 25,62 | 83,50 |
| 0,55 | 0,24 | 25,71 | 85,00 |
| -1,85 | -0,43 | 19,10 | 77,00 |
| -2,06 | -0,40 | 28,10 | 95,00 |
| 2,32 | 0,51 | 22,50 | 86,50 |
| -1,79 | -0,74 | 21,10 | 73,00 |
| -0,55 | -0,12 | 21,50 | 82,00 |
| -0,68 | -0,24 | 27,52 | 93,50 |
| -3,16 | -0,70 | 22,60 | 74,00 |
| 0,05 | 0,01 | 51,56 | 120,00 |
| -1,51 | -0,58 | 28,29 | 91,50 |
| -0,37 | -0,36 | 19,41 | 67,50 |
| -0,48 | -0,12 | NAN | NAN |
| 0,59 | 0,26 | 24,13 | 75,00 |
| -3,98 | -1,60 | 25,50 | 83,50 |
| -0,55 | -0,19 | 21,19 | 71,00 |
| -0,95 | -0,30 | 27,50 | 94,00 |
| -0,95 | -0,36 | 24,30 | 97,80 |
| -0,40 | -0,20 | 25,36 | 96,00 |
| -0,73 | -0,16 | NAN | NAN |
| 0,52 | 0,18 | 19,18 | 68,00 |
| -0,48 | -0,27 | 22,10 | 75,00 |
| -0,38 | -0,13 | 25,00 | 84,00 |
| -0,16 | -0,05 | 32,42 | 103,00 |
| -1,04 | -0,61 | 19,76 | 73,00 |
| 0,70 | 0,99 | 18,80 | 71,00 |
| -0,43 | -0,18 | 29,49 | 92,00 |
| -0,47 | -0,10 | 33,33 | 98,50 |
| -0,76 | -0,36 | 21,00 | 73,00 |
| 0,12 | 0,13 | 24,00 | 73,00 |
| NAN | NAN | NAN | NAN |
| -2,73 | -3,74 | 18,38 | 68,00 |
| 0,00 | 0,00 | 20,42 | 73,00 |
| 0,77 | 0,27 | 25,40 | 84,50 |
| 0,08 | 0,04 | NAN | NAN |

| | | | |
|---|---|---|---|
| 0,08 | 0,03 | 19,19 | 73,00 |
| -0,91 | -0,80 | 14,37 | 65,00 |
| 0,58 | 0,26 | 22,36 | 79,00 |
| -0,25 | -0,14 | 18,24 | 72,00 |
| -0,41 | -0,20 | 22,80 | 78,50 |
| -0,05 | -0,06 | 21,80 | 77,00 |
| 0,33 | 0,16 | 21,37 | 66,00 |
| -0,04 | -0,02 | 23,00 | 83,00 |
| -0,99 | -0,30 | 27,16 | 91,50 |
| NAN | NAN | 23,90 | 72,00 |
| -0,29 | -0,17 | 27,69 | 80,00 |
| -1,23 | -0,85 | 26,60 | 84,00 |
| 0,76 | 0,99 | 22,60 | 69,00 |
| 0,65 | 0,25 | 21,40 | 81,50 |
| -0,23 | -0,10 | 29,40 | 90,00 |
| 0,91 | 0,36 | 25,15 | 81,00 |
| NAN | NAN | 18,70 | 68,50 |
| 9,73 | 0,59 | 17,30 | 65,00 |
| 0,89 | 0,20 | 18,30 | 66,00 |
| 1,04 | 0,56 | 21,30 | 79,00 |
| -26,47 | -0,81 | 21,19 | 70,00 |
| 5,26 | 0,76 | 27,70 | 86,00 |
| 0,72 | 0,23 | 25,11 | 76,00 |
| -0,26 | -0,06 | NAN | NAN |
| 0,63 | 0,12 | 26,94 | 83,00 |
| 0,76 | 0,09 | NAN | 63,50 |
| 0,18 | 0,09 | 16,56 | 62,00 |
| -0,47 | -0,22 | 23,40 | 84,00 |
| -1,18 | -0,28 | 34,91 | 113,00 |
| -1,67 | -0,29 | 16,23 | 68,00 |
| 0,60 | 0,09 | 28,70 | 109,00 |
| -0,27 | -0,03 | 21,29 | 93,00 |
| -1,68 | -0,38 | 19,80 | 72,00 |
| -0,93 | -0,34 | 21,00 | 73,00 |
| -0,37 | -0,07 | 28,20 | 88,00 |
| -1,14 | -0,64 | 21,38 | 74,50 |
| -0,17 | -0,03 | 32,37 | 102,00 |
| 0,15 | 0,04 | 25,00 | NAN |
| 0,17 | 0,09 | 23,00 | 73,00 |
| 0,60 | 0,11 | 26,85 | 90,00 |
| 0,76 | 0,16 | NAN | 88,00 |
| -0,26 | -0,15 | 33,50 | 98,50 |
| 0,82 | 0,24 | 25,00 | 97,00 |
| -0,27 | -0,17 | NAN | NAN |
| -0,09 | -0,05 | 29,27 | 89,50 |
| -1,13 | -0,34 | 24,33 | 89,00 |
| -1,64 | -0,63 | 22,37 | 81,00 |
| -2,02 | -1,82 | 24,70 | 88,50 |
| -1,34 | -0,61 | 23,63 | 89,50 |
| 0,38 | 0,09 | 29,79 | 100,00 |

| | | | |
|---|---|---|---|
| 0,13 | 0,07 | 20,44 | 71,00 |
| 2,87 | 0,61 | 21,10 | 78,00 |
| -1,11 | -0,69 | 20,60 | 67,00 |
| -1,10 | -0,89 | 19,00 | 73,00 |
| 2,27 | 0,41 | 27,98 | 91,00 |
| 2,96 | 0,41 | 22,43 | 73,00 |
| 1,00 | 0,27 | 18,42 | 63,00 |
| -0,38 | -0,24 | 19,58 | 71,00 |
| -0,57 | -0,28 | 26,00 | 89,00 |
| -0,38 | -0,40 | 22,70 | 76,00 |
| -1,09 | -1,76 | NAN | NAN |
| -0,13 | -0,09 | 21,60 | 71,00 |
| -0,17 | -0,15 | 20,22 | 77,00 |
| -16,80 | -8,94 | 27,60 | 88,50 |
| -0,50 | -0,86 | NAN | 62,00 |
| -0,87 | -1,61 | 37,30 | 114,00 |
| 0,02 | 0,01 | 25,80 | 86,50 |
| -0,52 | -0,44 | 25,58 | 83,00 |
| 0,69 | 0,16 | 28,90 | 96,00 |
| -2,09 | -0,67 | 25,50 | 86,00 |
| -0,21 | -0,09 | 26,26 | 98,00 |
| -1,05 | -0,43 | 23,20 | 84,00 |
| 0,04 | 0,02 | NAN | NAN |
| -1,28 | -0,71 | 23,70 | 76,00 |
| -0,08 | -0,02 | 24,98 | 94,00 |
| -0,24 | -0,16 | 20,10 | 91,00 |
| -0,67 | -0,42 | 18,70 | 66,50 |
| -0,37 | -0,11 | 19,50 | 71,50 |
| -0,13 | -0,11 | 27,30 | 91,00 |
| 0,84 | 0,25 | 24,89 | 82,00 |
| -0,29 | -0,11 | NAN | NAN |
| -0,49 | -0,12 | 22,74 | 95,00 |
| -1,62 | -0,46 | 21,80 | 79,00 |
| 0,00 | 0,00 | 20,31 | 84,00 |
| 2,51 | 0,45 | 25,40 | 76,50 |
| -0,63 | -0,20 | 21,70 | 70,50 |
| -2,23 | -1,39 | 25,00 | 97,00 |
| 0,00 | 0,00 | 28,60 | 92,00 |
| -0,46 | -0,19 | 24,12 | 81,00 |
| 0,31 | 0,05 | NAN | NAN |
| 0,77 | 0,48 | 18,96 | 74,00 |
| -0,27 | -0,11 | 27,10 | 98,00 |
| NAN | NAN | 20,39 | 76,00 |
| NAN | NAN | 20,08 | 79,00 |
| NAN | NAN | 25,70 | 84,00 |
| NAN | NAN | NAN | 85,00 |
| NAN | NAN | 28,00 | 87,00 |
| NAN | NAN | 20,70 | 70,00 |
| NAN | NAN | 20,00 | 64,50 |
| NAN | NAN | 22,46 | 79,00 |

| | | | |
|---|---|---|---|
| NAN | NAN | 34,62 | 109,00 |
| NAN | NAN | 21,05 | 77,00 |
| NAN | NAN | 26,17 | 92,00 |
| NAN | NAN | 25,28 | 85,00 |
| NAN | NAN | 23,80 | 87,50 |
| 0,35 | 0,14 | 26,60 | 86,00 |
| NAN | NAN | 26,44 | 106,00 |
| -1,07 | -0,83 | 33,72 | 107,00 |
| -0,68 | -0,18 | 21,33 | 72,50 |
| NAN | NAN | 21,79 | 76,50 |
| 0,19 | 0,13 | 22,10 | 69,00 |
| NAN | NAN | 26,91 | 98,00 |
| NAN | NAN | 23,27 | 77,50 |
| -0,14 | -0,08 | NAN | NAN |
| NAN | NAN | 18,59 | 64,00 |
| -0,65 | -0,08 | NAN | NAN |
| -0,28 | -0,16 | 28,40 | 97,00 |
| NAN | NAN | 20,20 | 68,00 |
| NAN | NAN | NAN | NAN |
| NAN | NAN | 29,40 | 87,50 |
| NAN | NAN | 30,60 | 96,00 |
| -1,25 | -0,50 | 18,50 | 68,00 |
| -0,39 | -0,20 | 25,75 | 98,00 |
| 0,14 | 0,05 | 18,90 | NAN |
| -3,23 | -1,62 | 32,50 | 96,00 |
| -0,36 | -0,32 | 28,00 | 96,00 |
| -0,61 | -0,15 | 23,80 | 81,50 |
| -0,82 | -0,22 | 38,40 | 115,00 |
| -1,73 | -0,71 | 26,60 | 85,00 |
| 0,66 | 0,17 | NAN | NAN |
| 0,40 | 0,06 | 21,69 | 72,00 |
| -0,95 | -0,30 | 21,30 | 82,00 |
| -1,73 | -0,58 | 19,10 | 77,00 |
| -1,72 | -0,89 | NAN | 77,50 |
| 0,51 | 0,17 | 21,80 | 82,00 |
| 0,00 | 0,00 | 25,30 | 86,50 |
| -0,69 | -0,31 | 19,44 | 85,00 |
| -0,21 | -0,11 | 21,10 | 76,50 |
| 0,45 | 0,22 | NAN | NAN |
| -1,11 | -0,27 | 22,25 | 86,50 |
| -0,70 | -0,21 | 21,50 | 75,00 |
| -0,96 | -0,31 | 25,10 | 86,50 |
| NAN | NAN | 25,30 | 86,50 |
| -0,16 | -0,10 | 21,06 | 74,00 |
| 0,10 | 0,04 | 24,04 | 81,00 |
| -0,90 | -0,39 | NAN | NAN |
| -0,54 | -0,46 | NAN | 104,00 |
| -1,57 | -0,47 | 25,30 | 91,00 |
| -1,19 | -0,48 | NAN | NAN |
| NAN | NAN | 29,30 | 100,00 |

| | | | |
|---|---|---|---|
| -6,36 | -3,18 | 24,60 | 82,60 |
| -1,92 | -0,87 | 25,70 | 84,00 |
| -1,42 | -0,68 | 32,20 | 107,50 |
| -0,16 | -0,09 | 25,45 | NAN |
| NAN | NAN | NAN | NAN |
| -0,29 | -0,21 | 21,70 | 70,00 |
| 0,66 | 0,29 | 21,40 | 72,00 |
| 0,11 | 0,09 | 22,18 | 82,00 |
| -3,66 | -1,34 | 21,80 | 75,00 |
| 0,00 | 0,00 | 29,74 | NAN |
| NAN | NAN | 29,30 | 85,00 |
| -1,02 | -4,64 | NAN | NAN |
| -4,43 | -443,00 | 20,12 | 75,00 |
| -1,20 | -5,45 | 22,30 | 79,50 |
| -1,73 | -1,60 | NAN | NAN |
| NAN | NAN | NAN | NAN |
| NAN | NAN | NAN | NAN |
| NAN | NAN | NAN | NAN |
| NAN | NAN | 22,30 | 78,50 |
| NAN | NAN | NAN | NAN |
| NAN | NAN | NAN | NAN |
| NAN | NAN | 23,80 | 81,00 |
| NAN | NAN | 27,10 | 76,50 |
| -0,48 | -0,26 | 31,80 | 99,50 |
| -4,22 | -1,25 | 21,80 | NAN |
| 0,54 | 0,12 | NAN | NAN |
| -1,12 | -0,15 | 36,73 | NAN |
| NAN | NAN | 21,04 | 73,50 |
| NAN | NAN | 22,60 | 73,50 |
| -0,25 | -0,08 | 28,90 | 90,00 |
| -266,75 | -118,56 | 22,40 | 69,00 |
| -0,47 | -0,12 | NAN | NAN |
| NAN | NAN | 25,50 | 89,50 |
| NAN | NAN | 25,60 | 86,00 |
| -0,59 | -0,27 | NAN | NAN |
| 0,04 | 0,02 | NAN | NAN |
| 2,71 | 0,52 | NAN | NAN |
| -0,31 | -0,12 | 23,70 | 76,00 |
| 1,17 | 0,51 | 29,49 | 93,50 |
| -0,56 | -0,33 | NAN | NAN |
| NAN | NAN | NAN | NAN |
| 0,16 | 0,09 | 21,70 | 74,50 |
| -0,02 | -0,01 | NAN | NAN |
| -0,62 | -0,46 | 23,70 | 80,00 |
| -0,17 | -0,09 | NAN | NAN |
| -1,02 | -0,41 | NAN | NAN |
| NAN | NAN | 18,90 | 65,00 |
| NAN | NAN | NAN | NAN |
| NAN | NAN | 18,10 | 65,00 |
| NAN | NAN | NAN | NAN |

| | | | |
|---|---|---|---|
| -0,59 | -0,29 | 26,50 | 81,50 |
| -1,84 | -0,57 | 24,00 | 82,50 |
| -0,46 | -0,41 | 24,43 | NAN |
| 0,18 | 0,10 | 21,00 | 75,00 |
| -0,33 | -0,16 | NAN | NAN |
| -0,50 | -0,23 | 25,30 | 80,00 |
| 1,27 | 0,57 | 33,70 | 91,00 |
| -0,42 | -0,31 | 23,50 | 76,00 |
| -0,52 | -0,21 | 25,38 | NAN |
| -0,50 | -0,16 | 29,13 | 87,00 |
| -0,28 | -0,13 | NAN | NAN |
| 0,05 | 0,01 | NAN | NAN |
| NAN | NAN | NAN | NAN |
| NAN | NAN | 33,74 | NAN |
| NAN | NAN | 20,35 | 80,00 |
| NAN | NAN | 35,80 | 96,00 |

| Resting Energy (Kcal/day) 2 Yrs PD | Total Energy (Kcal/day) 2 Yrs PD | Fat mass ( Kg) 2 Yrs PD |
|---|---|---|
| 1273 | 2291 | 12,35 |
| 1341 | 2146 | 21,86 |
| 1382 | 2488 | 21,44 |
| 1570 | 2513 | 32,34 |
| 1345 | 2421 | 19,56 |
| 1195 | 2155 | 8,16 |
| NAN | NAN | NAN |
| 1526 | 2442 | 36,89 |
| 1371 | 2468 | 24,78 |
| 1590 | 2861 | 41,10 |
| 1171 | 1873 | 14,44 |
| 1555 | 2488 | 38,44 |
| 1271 | 2034 | 14,83 |
| 1733 | 2773 | 48,78 |
| 1289 | 2321 | 8,86 |
| 1267 | 2281 | 13,77 |
| 1284 | 2312 | 16,63 |
| 1305 | 2349 | 14,28 |
| 1404 | 2246 | 24,28 |
| 1412 | 2259 | 23,05 |
| NAN | NAN | NAN |
| 1439 | 2591 | 29,29 |
| NAN | NAN | 34,37 |
| 1320 | 2376 | 14,60 |
| 1512 | 2116 | 34,65 |
| NAN | NAN | NAN |
| 1397 | 2514 | 23,74 |
| 1555 | 2799 | 36,34 |
| 1237 | 2227 | 14,30 |
| 1356 | 2169 | 21,77 |
| 1412 | 2542 | 28,25 |
| 1318 | 1845 | 18,73 |
| 1383 | 2490 | 25,20 |
| 1501 | 2702 | 31,88 |
| 1338 | 2140 | 19,87 |
| 1327 | 2389 | 17,48 |
| 1254 | 2023 | 12,01 |
| 1599 | 2878 | 38,75 |
| NAN | NAN | NAN |
| 1399 | 2239 | 27,17 |
| 1370 | 1918 | 3,40 |
| 1372 | 2469 | 25,11 |
| NAN | NAN | NAN |
| 1296 | 2333 | 17,44 |
| 1369 | 2191 | 22,08 |
| 1386 | 2496 | 27,10 |
| NAN | NAN | NAN |

| | | |
|---|---|---|
| 1314 | 2365 | 16,22 |
| 1388 | 2221 | 21,83 |
| 1380 | 2484 | 18,99 |
| 1481 | 2073 | 33,88 |
| 1224 | 1959 | 12,20 |
| 1345 | 2422 | 21,34 |
| 1358 | 2444 | 21,05 |
| NAN | NAN | NAN |
| 1381 | 2210 | 21,18 |
| 1329 | 2376 | 13,79 |
| 1374 | 2198 | 20,24 |
| 1303 | 2085 | 18,71 |
| 1423 | 2561 | 27,33 |
| 1274 | 2293 | 13,50 |
| 1419 | 2555 | 21,14 |
| 1359 | 2446 | 24,62 |
| 1414 | 2263 | 26,21 |
| 1310 | 2357 | 16,99 |
| 1395 | 2231 | 28,67 |
| NAN | NAN | 16,32 |
| 1314 | 2365 | 21,17 |
| 1321 | 2378 | 19,07 |
| 1418 | 2269 | 28,55 |
| 1288 | 2318 | 14,73 |
| 2826 | 5088 | 84,22 |
| 1525 | 2746 | 32,39 |
| 1234 | 2222 | 11,10 |
| NAN | NAN | NAN |
| 1379 | 2483 | 19,88 |
| 1419 | 2255 | 22,05 |
| NAN | NAN | NAN |
| 1372 | 2470 | 24,44 |
| 1395 | 2515 | 23,95 |
| 1441 | 2594 | 26,26 |
| NAN | NAN | NAN |
| 1260 | 2267 | 13,95 |
| 1244 | 1996 | NAN |
| 1327 | 2389 | 19,44 |
| 1454 | 2618 | 32,11 |
| NAN | NAN | NAN |
| 1236 | 2226 | 13,17 |
| 1459 | 2335 | 33,41 |
| 1488 | 2678 | 35,19 |
| 1283 | 2050 | 13,39 |
| NAN | NAN | 19,00 |
| NAN | NAN | NAN |
| 1282 | 2052 | 10,25 |
| 1297 | 2335 | 16,90 |
| 1361 | 2178 | 21,76 |
| NAN | NAN | NAN |

| | | |
|---|---|---|
| 1281 | 2050 | 13,33 |
| 1139 | 2277 | 6,43 |
| 1378 | 2480 | 17,56 |
| 1205 | 2168 | 10,84 |
| 1319 | 2374 | 18,25 |
| 1234 | 1975 | 16,74 |
| 1314 | 2366 | 14,86 |
| 1318 | 2109 | 20,83 |
| 1316 | 2369 | 19,95 |
| 1343 | 2419 | 19,79 |
| 1524 | 2743 | 24,51 |
| 1413 | 2544 | 28,41 |
| 1484 | 2671 | 15,95 |
| 1331 | 2129 | 14,95 |
| 1426 | 2567 | 12,40 |
| 1327 | 2389 | 21,85 |
| 1249 | 1999 | 12,76 |
| NAN | NAN | NAN |
| 1237 | 1979 | 9,14 |
| 1313 | 2364 | 9,52 |
| 1303 | 2346 | 17,05 |
| 1512 | 2721 | 31,00 |
| 1401 | 2242 | 23,20 |
| NAN | NAN | NAN |
| 1385 | 2493 | 26,64 |
| 1213 | 2183 | 10,01 |
| 1234 | 2222 | 9,17 |
| 1352 | 2433 | 20,86 |
| 1484 | 2671 | 32,01 |
| 1264 | 2275 | 12,21 |
| 1536 | 2457 | 35,63 |
| NAN | NAN | NAN |
| 1317 | 2370 | 12,99 |
| 1307 | 2357 | 15,06 |
| 1362 | 2451 | 24,48 |
| 1307 | 2091 | 16,80 |
| 1502 | 2704 | 35,90 |
| 1314 | 2365 | 19,72 |
| 1368 | 2189 | 22,85 |
| 1601 | 2561 | NAN |
| NAN | NAN | NAN |
| 1822 | 3279 | 39,83 |
| 1406 | 2249 | 23,06 |
| NAN | NAN | NAN |
| 1501 | 2402 | 33,55 |
| 1339 | 2143 | 23,54 |
| 1346 | 2423 | 20,10 |
| 1310 | 2357 | 20,40 |
| 1427 | 2569 | 21,38 |
| 1465 | 2637 | 28,10 |

| | | |
|---|---|---|
| 1219 | 2195 | 13,69 |
| 1371 | 2194 | 19,95 |
| 1312 | 2362 | 13,08 |
| 1246 | 1994 | 14,22 |
| 1457 | 2623 | 31,58 |
| 1272 | 2544 | 13,43 |
| 1268 | 2283 | 12,04 |
| 1289 | 2063 | 13,50 |
| 1449 | 2319 | 21,60 |
| 1355 | 2438 | 19,54 |
| NAN | NAN | NAN |
| 1301 | 2082 | 14,82 |
| 1293 | 2327 | 16,81 |
| 1405 | 2530 | 24,65 |
| 1266 | 2279 | 12,24 |
| 1651 | 2641 | 37,50 |
| 1373 | 2471 | 25,60 |
| 1412 | 2259 | 24,59 |
| 1459 | 2645 | 30,59 |
| 1370 | 2192 | 29,44 |
| 1427 | 2568 | 26,50 |
| 1329 | 1861 | 16,14 |
| NAN | NAN | NAN |
| 1279 | 2047 | 18,52 |
| 1398 | NAN | 26,22 |
| 1316 | 1843 | 15,65 |
| 2313 | 1285 | 13,85 |
| 1293 | 2063 | 15,72 |
| 1450 | 2610 | 28,51 |
| 1524 | 2743 | 15,29 |
| NAN | NAN | NAN |
| 1295 | 2332 | 17,30 |
| 1369 | 2190 | 18,70 |
| 1273 | 2292 | 6,10 |
| 1382 | 2487 | 26,60 |
| 1316 | 2105 | 14,88 |
| 1392 | 2506 | 21,43 |
| 1440 | 2592 | 29,09 |
| 1360 | 2448 | 23,32 |
| NAN | NAN | NAN |
| 1260 | 2268 | 12,50 |
| 1520 | 2750 | 31,42 |
| 1319 | 2116 | 14,75 |
| 1284 | 2312 | 19,16 |
| 1441 | 2306 | 25,40 |
| 1299 | 2339 | NAN |
| 1357 | 2400 | 23,29 |
| 2077 | 1298 | 15,80 |
| 1255 | 2259 | 13,84 |
| 1313 | 1838 | 16,89 |

| | | |
|---|---|---|
| 1500 | 2700 | 38,84 |
| 1311 | 2098 | 17,86 |
| 1318 | 2108 | 20,90 |
| 1434 | 2580 | 22,49 |
| 1285 | 2313 | 17,00 |
| 1345 | 2421 | 21,45 |
| NAN | NAN | 25,98 |
| 2948 | 1638 | 44,80 |
| 1289 | 2321 | 17,38 |
| 1288 | 2060 | 15,05 |
| 1294 | 2330 | 17,29 |
| 1405 | 2530 | 27,49 |
| 1345 | 2152 | 22,43 |
| NAN | NAN | NAN |
| 1257 | 2011 | 14,10 |
| NAN | NAN | NAN |
| 1378 | 2480 | 25,30 |
| 1301 | 2343 | 14,20 |
| NAN | NAN | NAN |
| 1473 | 2651 | 28,54 |
| 1459 | 2626 | 32,76 |
| 1220 | 2196 | 6,20 |
| NAN | NAN | NAN |
| 1452 | 2614 | 6,12 |
| 1514 | 2422 | 35,97 |
| 1391 | 2504 | 24,20 |
| 1305 | 2088 | 19,79 |
| 1571 | 2826 | 43,60 |
| 1484 | 2375 | 31,06 |
| NAN | NAN | NAN |
| 1286 | 2057 | 15,99 |
| 1328 | 1860 | 15,27 |
| 1337 | 2139 | 20,50 |
| NAN | NAN | NAN |
| 1308 | 2354 | 15,90 |
| 1378 | 2480 | 24,90 |
| 1332 | 2397 | 14,33 |
| 1226 | 2207 | 14,20 |
| NAN | NAN | NAN |
| 1280 | 2304 | 16,45 |
| 1293 | 2327 | 17,80 |
| 1249 | 2338 | 17,10 |
| 1339 | 2410 | 21,80 |
| 1306 | 2351 | 15,62 |
| 1309 | 2503 | 24,11 |
| NAN | NAN | NAN |
| 1570 | 2826 | 32,80 |
| 1384 | 2215 | 24,00 |
| NAN | NAN | NAN |
| 1455 | 2619 | 29,73 |

| | | |
|---|---|---|
| 1308 | 2354 | 20,40 |
| 1581 | 2842 | 20,40 |
| 1520 | 2433 | 36,13 |
| NAN | NAN | NAN |
| NAN | NAN | NAN |
| 1360 | 2176 | 16,42 |
| 1308 | 2092 | 18,05 |
| 1251 | 2252 | 15,30 |
| 1419 | 2555 | 17,47 |
| NAN | NAN | NAN |
| 1417 | 2550 | 29,01 |
| NAN | NAN | NAN |
| 1280 | 2048 | 13,85 |
| 1287 | 2317 | 16,88 |
| NAN | NAN | NAN |
| NAN | NAN | NAN |
| NAN | NAN | NAN |
| NAN | NAN | NAN |
| 1369 | 2190 | 21,30 |
| NAN | NAN | NAN |
| NAN | NAN | NAN |
| 1365 | 2457 | 19,70 |
| 1446 | 2603 | 26,20 |
| 1528 | 2445 | 38,60 |
| 1362 | 2180 | 19,10 |
| NAN | NAN | NAN |
| NAN | NAN | NAN |
| 1225 | 2205 | 10,42 |
| 2106 | 1317 | 20,50 |
| 1389 | 2222 | 28,70 |
| 1296 | 2074 | 15,50 |
| NAN | NAN | NAN |
| 1375 | 2475 | 21,34 |
| 1461 | 2338 | 32,16 |
| NAN | NAN | NAN |
| NAN | NAN | NAN |
| NAN | NAN | NAN |
| 1543 | 2314 | 16,10 |
| 1506 | 2711 | 34,36 |
| NAN | NAN | NAN |
| NAN | NAN | NAN |
| 1351 | 2431 | 20,40 |
| NAN | NAN | NAN |
| 1332 | 2132 | 22,50 |
| NAN | NAN | NAN |
| NAN | NAN | NAN |
| 1259 | 2014 | 10,30 |
| NAN | NAN | NAN |
| 1244 | 1991 | 10,90 |
| NAN | NAN | NAN |

| | | |
|---|---|---|
| 1362 | 2452 | 19,60 |
| 1438 | 2301 | 27,50 |
| NAN | NAN | NAN |
| 1321 | 2114 | 15,08 |
| NAN | NAN | NAN |
| 1377 | 2204 | 21,10 |
| 1600 | 2880 | 41,50 |
| 1379 | 2206 | 19,90 |
| NAN | NAN | NAN |
| 1467 | 2640 | 26,00 |
| NAN | NAN | NAN |
| NAN | NAN | NAN |
| NAN | NAN | NAN |
| NAN | NAN | NAN |
| 1258 | 2265 | 10,01 |
| 1544 | 2779 | 38,38 |

| Lean body mass (Kg) 2 Yrs PD | Skeletal Muscle Mass (Kg) 2 Yrs PD | Body Water (L) 2 Yrs PD | BMI >25 (Kg/m2) 2 yrs PD |
|---|---|---|---|
| 40,25 | 18,40 | 29,70 | 0,00 |
| 39,14 | 16,50 | 28,70 | 0,00 |
| 44,61 | 80,20 | 32,80 | 0,00 |
| 53,63 | 27,00 | 42,20 | 1,00 |
| 41,89 | 19,70 | 31,20 | 0,00 |
| 39,64 | 17,50 | 28,70 | 0,00 |
| NAN | NAN | NAN | NAN |
| 46,86 | 21,70 | 34,60 | 1,00 |
| 39,87 | 17,90 | 29,60 | 1,00 |
| 50,45 | 23,20 | 37,60 | 1,00 |
| 25,56 | 8,60 | 18,20 | 0,00 |
| 17,10 | 22,40 | 36,30 | 1,00 |
| 37,72 | 16,70 | 27,30 | 0,00 |
| 60,42 | 29,70 | 45,10 | 1,00 |
| 45,74 | 20,70 | 33,50 | 0,00 |
| 38,08 | 15,70 | 27,50 | 0,00 |
| 37,37 | 15,40 | 26,10 | 0,00 |
| 42,24 | 18,80 | 30,80 | 0,00 |
| 44,92 | 19,40 | 32,40 | 0,00 |
| 46,65 | 22,10 | 35,10 | 0,00 |
| NAN | NAN | NAN | 0,00 |
| 43,76 | 18,00 | 32,50 | 1,00 |
| 46,98 | 22,10 | 34,80 | 1,00 |
| 42,27 | 18,30 | 31,21 | 0,00 |
| 47,30 | 22,50 | 35,50 | 1,00 |
| NAN | NAN | NAN | NAN |
| 44,06 | 20,70 | 33,10 | 0,00 |
| 50,96 | 23,50 | 37,60 | 1,00 |
| 33,90 | 14,50 | 24,90 | 0,00 |
| 41,58 | 19,10 | 30,70 | 0,00 |
| 41,45 | 18,70 | 30,60 | 1,00 |
| 39,37 | 16,80 | 47,90 | 0,00 |
| 40,95 | 17,80 | 30,40 | 1,00 |
| 48,82 | 22,70 | 36,40 | 1,00 |
| 40,68 | 18,20 | 30,10 | 0,00 |
| 41,77 | 17,60 | 30,40 | 0,00 |
| 39,49 | 16,00 | 28,50 | 0,00 |
| 53,95 | 24,90 | 40,00 | 1,00 |
| NAN | NAN | NAN | NAN |
| 40,98 | 18,60 | 30,50 | 1,00 |
| 17,10 | 19,90 | 32,00 | 1,00 |
| 39,64 | 18,00 | 29,60 | 1,00 |
| NAN | NAN | NAN | 0,00 |
| 38,01 | 16,70 | 27,90 | 0,00 |
| 42,37 | 19,00 | 31,40 | 0,00 |
| 39,45 | 17,20 | 43,80 | 1,00 |
| NAN | NAN | NAN | 0,00 |

| | | | |
|---|---|---|---|
| 41,38 | 16,30 | 30,30 | 0,00 |
| 44,92 | NAN | NAN | 0,00 |
| 41,31 | 18,50 | 30,10 | 0,00 |
| 17,10 | 20,80 | 32,70 | 1,00 |
| 34,40 | 14,70 | 25,50 | 0,00 |
| 40,16 | 17,50 | 47,90 | 0,00 |
| 41,95 | 19,10 | 30,80 | 0,00 |
| NAN | NAN | NAN | NAN |
| 44,72 | 20,60 | 33,30 | 0,00 |
| 44,60 | 19,70 | 55,60 | 1,00 |
| 44,76 | 20,00 | 33,30 | 0,00 |
| 37,59 | 16,40 | 27,90 | 1,00 |
| 43,67 | 18,90 | 32,20 | 1,00 |
| 39,20 | 16,30 | 29,00 | 0,00 |
| 46,46 | 21,90 | 34,30 | 1,00 |
| 38,53 | 16,60 | 28,00 | 1,00 |
| 43,79 | 20,10 | 32,30 | 1,00 |
| 40,11 | 17,60 | 29,40 | 0,00 |
| 38,88 | 17,80 | 29,10 | 1,00 |
| 35,03 | 13,10 | 24,70 | 0,00 |
| 36,48 | 15,40 | 26,00 | 0,00 |
| 39,43 | 16,90 | 48,40 | 0,00 |
| 41,90 | 18,40 | 30,80 | 1,00 |
| 39,67 | 17,10 | 29,10 | 0,00 |
| 73,68 | 38,00 | 56,00 | 1,00 |
| 51,26 | 23,20 | 37,90 | 1,00 |
| 36,75 | 15,30 | 27,20 | 0,00 |
| NAN | NAN | NAN | NAN |
| 45,82 | 20,00 | 33,00 | 0,00 |
| 47,35 | 22,30 | 35,00 | 1,00 |
| NAN | NAN | NAN | 0,00 |
| 40,36 | 18,70 | 30,10 | 1,00 |
| 43,70 | 19,40 | 32,10 | 0,00 |
| 47,04 | 22,20 | 35,00 | 1,00 |
| NAN | NAN | NAN | NAN |
| 37,00 | 15,80 | 26,80 | 0,00 |
| 34,98 | 13,90 | 24,70 | 0,00 |
| 39,86 | 18,80 | 29,40 | 1,00 |
| 42,79 | 19,20 | 31,70 | 1,00 |
| NAN | NAN | NAN | 0,00 |
| 34,93 | 14,60 | 25,70 | 0,00 |
| 42,09 | 19,40 | 31,20 | 1,00 |
| 43,86 | 20,10 | 32,90 | 1,00 |
| 40,41 | 17,60 | 30,00 | 0,00 |
| NAN | 20,17 | 29,00 | 0,00 |
| NAN | NAN | NAN | NAN |
| 43,50 | 18,40 | 31,80 | 0,00 |
| 38,69 | 15,80 | 28,30 | 0,00 |
| 41,69 | 18,80 | 30,40 | 1,00 |
| NAN | NAN | NAN | NAN |

| | | | |
|---|---|---|---|
| 40,27 | 16,70 | 29,20 | 0,00 |
| 37,57 | 15,30 | 26,80 | 0,00 |
| 42,59 | 19,70 | 30,80 | 0,00 |
| 37,61 | 15,50 | 27,30 | 0,00 |
| 40,00 | 17,70 | 29,50 | 0,00 |
| 33,71 | 13,00 | 24,20 | 0,00 |
| 42,84 | 19,40 | 31,80 | 0,00 |
| 37,32 | 16,60 | 27,90 | 0,00 |
| 37,95 | 16,10 | 28,20 | 1,00 |
| 41,36 | 18,50 | 30,98 | 0,00 |
| 45,49 | 20,80 | 34,10 | 1,00 |
| 41,44 | 19,00 | 30,80 | 1,00 |
| 44,85 | 19,90 | 32,90 | 0,00 |
| 44,72 | 19,70 | 32,80 | 0,00 |
| 41,30 | 18,50 | 30,60 | 1,00 |
| 37,40 | 16,10 | 27,30 | 1,00 |
| 36,94 | 16,50 | 27,30 | 0,00 |
| NAN | NAN | NAN | 0,00 |
| 39,01 | 17,40 | 28,90 | 0,00 |
| 48,03 | 21,70 | 35,20 | 0,00 |
| 39,25 | 17,50 | 28,70 | 0,00 |
| 50,45 | 23,00 | 37,70 | 1,00 |
| 44,42 | 20,00 | 34,00 | 1,00 |
| NAN | NAN | NAN | NAN |
| 41,76 | 18,60 | 30,80 | 1,00 |
| 33,19 | 14,40 | 24,50 | NAN |
| 36,68 | 16,80 | 28,20 | 0,00 |
| 41,44 | 17,80 | 31,00 | 0,00 |
| 46,54 | 21,60 | 34,80 | 1,00 |
| 39,24 | 16,60 | 28,50 | 0,00 |
| 49,32 | 23,00 | 36,50 | 1,00 |
| NAN | NAN | NAN | 0,00 |
| 45,01 | 19,80 | NAN | 0,00 |
| 41,69 | 16,90 | 29,90 | 0,00 |
| 39,02 | 16,30 | 28,20 | 1,00 |
| 40,00 | 17,10 | 29,10 | 0,00 |
| 44,87 | 20,90 | 33,50 | 1,00 |
| 37,93 | 17,10 | 28,10 | 1,00 |
| 41,45 | 18,50 | 30,00 | 0,00 |
| NAN | NAN | NAN | 1,00 |
| NAN | NAN | NAN | NAN |
| 50,27 | 24,10 | 37,00 | 1,00 |
| 45,89 | 20,80 | 33,90 | 1,00 |
| NAN | NAN | NAN | NAN |
| 47,10 | 21,20 | 34,20 | 1,00 |
| 37,21 | 14,80 | 26,90 | 0,00 |
| 41,50 | 17,70 | 49,40 | 0,00 |
| 36,70 | 15,70 | 27,00 | 0,00 |
| 50,17 | 23,10 | 36,60 | 0,00 |
| 46,15 | 21,60 | 35,10 | 1,00 |

| | | | |
|---|---|---|---|
| 32,31 | 13,10 | 23,50 | 0,00 |
| 44,70 | 20,50 | 32,70 | 0,00 |
| 44,37 | 19,40 | 32,10 | 0,00 |
| 35,08 | 15,60 | 25,50 | 0,00 |
| 43,67 | 20,20 | 32,70 | 1,00 |
| 39,07 | 15,80 | 54,00 | 0,00 |
| 39,96 | NAN | 29,10 | 0,00 |
| 41,10 | 18,00 | 30,40 | 0,00 |
| 43,35 | 19,70 | 31,80 | 1,00 |
| 43,10 | 19,90 | 32,40 | 0,00 |
| NAN | NAN | NAN | NAN |
| 41,28 | 18,40 | 30,60 | 0,00 |
| 38,24 | 16,30 | 27,90 | 0,00 |
| 44,25 | 19,80 | 33,00 | 1,00 |
| 40,36 | 18,60 | 29,00 | NAN |
| 61,60 | 29,50 | 46,20 | 1,00 |
| 39,30 | 16,60 | 28,70 | 1,00 |
| 45,06 | 21,60 | 33,40 | 1,00 |
| 46,16 | 22,00 | 34,50 | 1,00 |
| 35,11 | 15,90 | 26,70 | 1,00 |
| 44,59 | 19,50 | 33,30 | 1,00 |
| 43,36 | 18,90 | 31,70 | 0,00 |
| NAN | NAN | NAN | NAN |
| 34,83 | 14,90 | 25,80 | 0,00 |
| 41,78 | 19,00 | 30,90 | 0,00 |
| 42,25 | NAN | 31,20 | 0,00 |
| 40,20 | 16,70 | 29,20 | 0,00 |
| 39,28 | 17,10 | 18,50 | 0,00 |
| 45,80 | 20,40 | 33,80 | 1,00 |
| 41,46 | 17,10 | 29,20 | 0,00 |
| NAN | NAN | NAN | NAN |
| 38,05 | 15,80 | 27,60 | 0,00 |
| 45,66 | 21,20 | 33,60 | 0,00 |
| 36,93 | 16,30 | 27,21 | 0,00 |
| 39,50 | 16,90 | 28,80 | 1,00 |
| 42,97 | 18,90 | 31,00 | 0,00 |
| 45,82 | 21,10 | 34,10 | 1,00 |
| 44,06 | 21,50 | 32,09 | 1,00 |
| 39,98 | 17,90 | 29,60 | 0,00 |
| NAN | NAN | NAN | NAN |
| 38,50 | 15,90 | 27,70 | 0,00 |
| 52,53 | 25,20 | 39,40 | 1,00 |
| 43,50 | 17,70 | 31,90 | 0,00 |
| 34,84 | 14,70 | 25,40 | 0,00 |
| 47,87 | 22,50 | 35,70 | 1,00 |
| 38,36 | 16,50 | 27,90 | NAN |
| 39,61 | 17,10 | 29,30 | 1,00 |
| 39,80 | 16,80 | 29,40 | 0,00 |
| 36,60 | 15,80 | 26,90 | 0,00 |
| 15,90 | 17,90 | 29,70 | 0,00 |

| | | | |
|---|---|---|---|
| 41,66 | 19,50 | 31,20 | 1,00 |
| 39,44 | 16,50 | 28,70 | 0,00 |
| 37,20 | 15,60 | 27,40 | 1,00 |
| 41,41 | 18,30 | 30,50 | 1,00 |
| 37,10 | 15,90 | 27,40 | 0,00 |
| 40,00 | 18,30 | 29,70 | 1,00 |
| 47,77 | 20,20 | 34,40 | 1,00 |
| 52,60 | 24,60 | 37,40 | 1,00 |
| 37,22 | 15,00 | 27,00 | 0,00 |
| 39,35 | 17,90 | 29,00 | 0,00 |
| 37,90 | 16,30 | 27,90 | 0,00 |
| NAN | 18,90 | 30,60 | 1,00 |
| 39,02 | 16,60 | 28,90 | 0,00 |
| NAN | NAN | NAN | NAN |
| 36,50 | 15,40 | 26,40 | 0,00 |
| NAN | NAN | NAN | NAN |
| 40,23 | 17,90 | 29,40 | 1,00 |
| 41,90 | 18,60 | 31,20 | 0,00 |
| NAN | NAN | NAN | NAN |
| 48,70 | 20,70 | 35,40 | 1,00 |
| 42,70 | 17,60 | 31,30 | 1,00 |
| 39,90 | 17,50 | 29,30 | 0,00 |
| NAN | NAN | NAN | 1,00 |
| 44,40 | 19,00 | 31,60 | 0,00 |
| 46,28 | 21,50 | 34,30 | 1,00 |
| 42,96 | 18,80 | 31,40 | 1,00 |
| 36,70 | 15,30 | 27,10 | 0,00 |
| 45,70 | 22,30 | 34,30 | 1,00 |
| 47,54 | 21,60 | 35,10 | 1,00 |
| NAN | NAN | NAN | NAN |
| 38,16 | 16,70 | 28,00 | 0,00 |
| 44,13 | NAN | 31,30 | 0,00 |
| 39,95 | 17,00 | 29,00 | 0,00 |
| NAN | NAN | NAN | NAN |
| 40,97 | 18,10 | 30,10 | 0,00 |
| 40,60 | 17,90 | 29,90 | 1,00 |
| 45,47 | 20,70 | 33,40 | 0,00 |
| 32,70 | 12,30 | 23,20 | 0,00 |
| NAN | NAN | NAN | NAN |
| 37,00 | 17,40 | 27,60 | 0,00 |
| 37,20 | 15,40 | 26,90 | 0,00 |
| 38,70 | 16,70 | 28,50 | 1,00 |
| 38,90 | 16,90 | 27,90 | 1,00 |
| 41,03 | 18,90 | 29,90 | 0,00 |
| 42,90 | 18,80 | 31,80 | 0,00 |
| NAN | NAN | NAN | NAN |
| 56,35 | 25,70 | 41,50 | NAN |
| 42,30 | 20,00 | 31,50 | 1,00 |
| NAN | NAN | NAN | NAN |
| 45,27 | 21,60 | 33,70 | 1,00 |

| | | | |
|---|---|---|---|
| 36,40 | 15,70 | 27,00 | 0,00 |
| 41,28 | 18,80 | 29,40 | 1,00 |
| 46,90 | 22,50 | 35,20 | 1,00 |
| NAN | NAN | NAN | 1,00 |
| NAN | NAN | NAN | NAN |
| 46,90 | 20,00 | 34,60 | 0,00 |
| 38,20 | NAN | 28,10 | 0,00 |
| 34,60 | 16,30 | 25,80 | 0,00 |
| 25,48 | 21,30 | 33,70 | 0,00 |
| NAN | NAN | NAN | 1,00 |
| 41,30 | 19,10 | 30,90 | 1,00 |
| NAN | NAN | NAN | NAN |
| 39,60 | 17,00 | 29,70 | 0,00 |
| 37,50 | 16,80 | 27,80 | 0,00 |
| NAN | NAN | NAN | NAN |
| NAN | NAN | NAN | NAN |
| NAN | NAN | NAN | NAN |
| NAN | NAN | NAN | NAN |
| 43,10 | 19,40 | 31,90 | 0,00 |
| NAN | NAN | NAN | NAN |
| NAN | NAN | NAN | NAN |
| 44,30 | NAN | 33,30 | 0,00 |
| 47,70 | 21,40 | 35,60 | 1,00 |
| 45,40 | 20,80 | 37,70 | 1,00 |
| 44,50 | 21,30 | 32,90 | 0,00 |
| NAN | NAN | NAN | NAN |
| NAN | NAN | NAN | 1,00 |
| 36,30 | 14,90 | 26,50 | 0,00 |
| 37,40 | 15,40 | 27,80 | 0,00 |
| 38,20 | 16,90 | 28,10 | 1,00 |
| 39,90 | 18,20 | 29,90 | 0,00 |
| NAN | NAN | NAN | NAN |
| 43,80 | 19,50 | 32,60 | 1,00 |
| 43,60 | 20,10 | 32,60 | 1,00 |
| NAN | NAN | NAN | NAN |
| NAN | NAN | NAN | NAN |
| NAN | NAN | NAN | NAN |
| 46,10 | NAN | 34,30 | 0,00 |
| 46,89 | 21,00 | 34,80 | 1,00 |
| NAN | NAN | NAN | NAN |
| NAN | NAN | NAN | NAN |
| 41,80 | 19,20 | 31,50 | 0,00 |
| NAN | NAN | NAN | NAN |
| 37,40 | 16,00 | 27,40 | 0,00 |
| NAN | NAN | NAN | NAN |
| NAN | NAN | NAN | NAN |
| 40,60 | 17,80 | 29,90 | 0,00 |
| NAN | NAN | NAN | NAN |
| 38,70 | 15,70 | 27,80 | 0,00 |
| NAN | NAN | NAN | NAN |

| | | | |
|---|---|---|---|
| 44,00 | 19,10 | 32,50 | 1,00 |
| 45,40 | 21,10 | 34,40 | 0,00 |
| NAN | NAN | NAN | 0,00 |
| 43,50 | 18,40 | 21,80 | 0,00 |
| NAN | NAN | NAN | NAN |
| 44,20 | 21,00 | 33,20 | 1,00 |
| 41,50 | 23,80 | 37,90 | 1,00 |
| 45,60 | 19,50 | 33,40 | 0,00 |
| NAN | NAN | NAN | 1,00 |
| 50,45 | 23,10 | NAN | 1,00 |
| NAN | NAN | NAN | NAN |
| NAN | NAN | NAN | NAN |
| NAN | NAN | NAN | NAN |
| NAN | NAN | NAN | 1,00 |
| 40,79 | NAN | NAN | 0,00 |
| 47,60 | 22,10 | 35,20 | 1,00 |

| WC >89.5 cm 2 Yrs PD | Abnormal Glucose Regulation 2 yrs PD | IFG 2 yrs PD | IGT 2 yrs PD |
|---|---|---|---|
| 0,00 | 0,00 | 0,00 | 0,00 |
| 0,00 | 0,00 | 0,00 | 0,00 |
| 0,00 | 0,00 | 0,00 | 0,00 |
| 1,00 | 1,00 | 1,00 | 0,00 |
| 0,00 | 1,00 | 1,00 | 0,00 |
| 0,00 | 0,00 | 0,00 | 0,00 |
| NAN | 0,00 | 0,00 | NAN |
| NAN | 0,00 | 0,00 | 0,00 |
| 0,00 | 0,00 | 0,00 | NAN |
| 1,00 | 1,00 | 1,00 | 0,00 |
| 0,00 | 0,00 | 0,00 | NAN |
| 1,00 | 1,00 | 1,00 | NAN |
| 0,00 | 0,00 | 0,00 | NAN |
| 1,00 | 0,00 | 0,00 | 0,00 |
| 0,00 | 0,00 | 0,00 | NAN |
| 0,00 | 0,00 | 0,00 | NAN |
| 0,00 | 0,00 | 0,00 | NAN |
| 0,00 | 0,00 | 0,00 | 0,00 |
| 0,00 | 0,00 | 0,00 | 0,00 |
| 1,00 | 1,00 | 0,00 | NAN |
| 0,00 | 0,00 | 0,00 | 0,00 |
| 1,00 | 0,00 | 0,00 | 0,00 |
| 1,00 | 1,00 | 1,00 | NAN |
| 0,00 | 0,00 | 0,00 | NAN |
| 1,00 | 0,00 | 0,00 | NAN |
| NAN | 0,00 | 0,00 | NAN |
| 0,00 | 0,00 | 0,00 | NAN |
| 1,00 | 1,00 | 0,00 | NAN |
| 0,00 | 1,00 | 1,00 | 0,00 |
| 0,00 | 0,00 | 0,00 | 0,00 |
| 1,00 | 1,00 | 1,00 | NAN |
| NAN | 1,00 | 1,00 | 0,00 |
| 0,00 | 0,00 | 0,00 | NAN |
| 0,00 | 0,00 | 0,00 | NAN |
| 0,00 | 0,00 | 0,00 | NAN |
| 0,00 | 0,00 | 0,00 | 0,00 |
| 0,00 | 0,00 | 0,00 | 0,00 |
| 1,00 | 0,00 | 0,00 | NAN |
| NAN | 0,00 | 0,00 | NAN |
| 0,00 | 0,00 | 0,00 | NAN |
| 0,00 | 0,00 | 0,00 | NAN |
| 1,00 | 1,00 | 1,00 | 0,00 |
| 0,00 | 0,00 | 0,00 | 0,00 |
| 0,00 | 0,00 | 0,00 | 0,00 |
| 0,00 | 0,00 | 0,00 | NAN |
| 0,00 | 0,00 | 0,00 | 0,00 |
| 0,00 | 0,00 | 0,00 | NAN |

| | | | |
|---|---|---|---|
| 0,00 | 0,00 | 0,00 | 0,00 |
| 0,00 | 0,00 | 0,00 | NAN |
| 0,00 | 0,00 | 0,00 | NAN |
| 1,00 | 0,00 | 0,00 | NAN |
| 0,00 | 0,00 | 0,00 | NAN |
| 0,00 | 0,00 | 0,00 | 1,00 |
| 0,00 | 0,00 | 0,00 | 0,00 |
| 0,00 | 0,00 | 0,00 | NAN |
| 0,00 | 0,00 | 0,00 | NAN |
| 0,00 | 0,00 | 0,00 | NAN |
| 0,00 | 0,00 | 0,00 | 0,00 |
| 0,00 | 0,00 | 0,00 | 0,00 |
| 0,00 | 0,00 | 0,00 | 0,00 |
| 0,00 | 0,00 | 0,00 | NAN |
| 1,00 | 0,00 | 0,00 | 0,00 |
| 0,00 | 0,00 | 0,00 | 0,00 |
| 0,00 | 1,00 | 1,00 | NAN |
| 0,00 | 0,00 | 0,00 | 0,00 |
| 1,00 | 0,00 | 0,00 | NAN |
| 0,00 | 0,00 | 0,00 | 0,00 |
| 0,00 | 0,00 | 0,00 | NAN |
| 0,00 | 0,00 | 0,00 | 0,00 |
| 1,00 | 0,00 | 0,00 | NAN |
| 0,00 | 0,00 | 0,00 | NAN |
| 1,00 | 0,00 | 0,00 | 0,00 |
| 1,00 | 1,00 | 1,00 | NAN |
| 0,00 | 0,00 | 0,00 | 0,00 |
| NAN | 1,00 | 1,00 | NAN |
| 0,00 | 0,00 | 0,00 | 0,00 |
| 0,00 | 0,00 | 0,00 | NAN |
| 0,00 | 0,00 | 0,00 | NAN |
| 1,00 | 0,00 | 0,00 | 1,00 |
| 1,00 | 1,00 | 1,00 | 0,00 |
| 1,00 | 0,00 | 0,00 | 0,00 |
| NAN | 0,00 | 0,00 | 0,00 |
| 0,00 | 0,00 | 0,00 | 0,00 |
| 0,00 | 0,00 | 0,00 | 0,00 |
| 0,00 | 1,00 | 1,00 | NAN |
| 1,00 | 0,00 | 0,00 | NAN |
| 0,00 | 1,00 | 0,00 | 0,00 |
| 0,00 | 0,00 | 0,00 | 1,00 |
| 1,00 | 1,00 | 1,00 | 1,00 |
| 1,00 | 0,00 | 0,00 | NAN |
| 0,00 | 1,00 | 1,00 | NAN |
| 0,00 | 0,00 | 0,00 | NAN |
| NAN | 0,00 | 0,00 | 0,00 |
| 0,00 | 0,00 | 0,00 | NAN |
| 0,00 | 0,00 | 0,00 | 0,00 |
| 0,00 | 0,00 | 0,00 | NAN |
| NAN | 0,00 | 0,00 | 0,00 |

| | | | |
|---|---|---|---|
| 0,00 | 0,00 | 0,00 | 0,00 |
| 0,00 | 0,00 | 0,00 | 0,00 |
| 0,00 | 0,00 | 0,00 | 0,00 |
| 0,00 | 0,00 | 0,00 | 0,00 |
| 0,00 | 0,00 | 0,00 | 0,00 |
| 0,00 | 0,00 | 0,00 | 0,00 |
| 0,00 | 0,00 | 0,00 | NAN |
| 0,00 | 0,00 | 0,00 | NAN |
| 1,00 | 1,00 | 1,00 | NAN |
| 0,00 | 0,00 | 0,00 | NAN |
| 0,00 | 0,00 | 0,00 | NAN |
| 0,00 | 1,00 | 1,00 | NAN |
| 0,00 | 0,00 | 0,00 | 0,00 |
| 0,00 | 0,00 | 0,00 | NAN |
| 1,00 | 0,00 | 0,00 | 0,00 |
| 0,00 | 0,00 | 0,00 | 0,00 |
| 0,00 | 0,00 | 0,00 | NAN |
| 0,00 | 0,00 | 0,00 | NAN |
| 0,00 | 0,00 | 0,00 | 0,00 |
| 0,00 | 0,00 | 0,00 | 0,00 |
| 0,00 | 0,00 | 0,00 | 0,00 |
| 0,00 | 0,00 | 0,00 | NAN |
| 0,00 | 0,00 | 0,00 | 0,00 |
| NAN | 0,00 | 0,00 | 0,00 |
| 0,00 | 0,00 | 0,00 | 0,00 |
| 0,00 | 0,00 | 0,00 | NAN |
| 0,00 | 0,00 | 0,00 | NAN |
| 0,00 | 0,00 | 0,00 | NAN |
| 1,00 | 0,00 | 0,00 | 0,00 |
| 0,00 | 0,00 | 0,00 | 0,00 |
| 1,00 | 0,00 | 0,00 | NAN |
| 1,00 | 1,00 | 1,00 | NAN |
| 0,00 | 0,00 | 0,00 | 0,00 |
| 0,00 | 0,00 | 0,00 | NAN |
| 0,00 | 1,00 | 1,00 | NAN |
| 0,00 | 0,00 | 0,00 | NAN |
| 1,00 | 1,00 | 1,00 | NAN |
| NAN | 1,00 | 1,00 | 0,00 |
| 0,00 | 0,00 | 0,00 | NAN |
| 1,00 | 0,00 | 0,00 | NAN |
| 0,00 | 0,00 | 0,00 | 0,00 |
| 1,00 | 1,00 | 1,00 | NAN |
| 1,00 | 0,00 | 0,00 | 0,00 |
| NAN | 0,00 | 0,00 | 0,00 |
| 1,00 | 0,00 | 0,00 | 0,00 |
| 1,00 | 0,00 | 0,00 | NAN |
| 0,00 | 0,00 | 0,00 | NAN |
| 1,00 | 0,00 | 0,00 | NAN |
| 1,00 | 0,00 | 0,00 | 0,00 |
| 1,00 | 0,00 | 0,00 | 0,00 |

| | | | |
|---|---|---|---|
| 0,00 | 0,00 | 0,00 | 0,00 |
| 0,00 | 0,00 | 0,00 | NAN |
| 0,00 | 0,00 | 0,00 | 0,00 |
| 0,00 | 0,00 | 0,00 | NAN |
| 1,00 | 0,00 | 0,00 | 0,00 |
| 0,00 | 0,00 | 0,00 | NAN |
| 0,00 | 0,00 | 0,00 | 0,00 |
| 0,00 | 0,00 | 0,00 | NAN |
| 1,00 | 0,00 | 0,00 | 0,00 |
| 0,00 | 1,00 | 1,00 | NAN |
| NAN | 0,00 | 0,00 | NAN |
| 0,00 | 0,00 | 0,00 | NAN |
| 0,00 | 0,00 | 0,00 | NAN |
| 1,00 | 1,00 | 0,00 | NAN |
| 0,00 | 0,00 | 0,00 | 0,00 |
| 1,00 | 0,00 | 0,00 | NAN |
| 0,00 | 0,00 | 0,00 | 0,00 |
| 0,00 | 0,00 | 0,00 | NAN |
| 1,00 | 0,00 | 0,00 | 0,00 |
| 0,00 | 0,00 | 0,00 | NAN |
| 1,00 | 0,00 | 0,00 | NAN |
| 0,00 | 0,00 | 0,00 | 0,00 |
| NAN | 0,00 | 0,00 | 0,00 |
| 0,00 | 0,00 | 0,00 | NAN |
| 1,00 | 1,00 | 1,00 | 0,00 |
| 1,00 | 0,00 | 0,00 | NAN |
| 0,00 | 0,00 | 0,00 | NAN |
| 0,00 | 0,00 | 0,00 | NAN |
| 1,00 | 0,00 | 0,00 | 0,00 |
| 0,00 | 0,00 | 0,00 | 0,00 |
| NAN | 0,00 | 0,00 | 0,00 |
| 1,00 | 0,00 | 0,00 | NAN |
| 0,00 | 0,00 | 0,00 | NAN |
| 0,00 | 1,00 | 1,00 | NAN |
| 0,00 | 0,00 | 0,00 | 1,00 |
| 0,00 | 0,00 | 0,00 | 0,00 |
| 1,00 | 0,00 | 0,00 | NAN |
| 1,00 | 0,00 | 0,00 | NAN |
| 0,00 | 0,00 | 0,00 | NAN |
| NAN | 0,00 | 0,00 | 0,00 |
| 0,00 | 0,00 | 0,00 | 1,00 |
| 1,00 | 0,00 | 0,00 | NAN |
| 0,00 | 0,00 | 0,00 | 0,00 |
| 0,00 | 0,00 | 0,00 | 0,00 |
| 0,00 | 0,00 | 0,00 | NAN |
| 0,00 | 0,00 | 0,00 | NAN |
| 0,00 | 0,00 | 0,00 | NAN |
| 0,00 | 0,00 | 0,00 | NAN |
| 0,00 | 0,00 | 0,00 | NAN |
| 0,00 | 0,00 | 0,00 | 0,00 |

| | | | |
|---|---|---|---|
| 1,00 | 0,00 | 0,00 | 0,00 |
| 0,00 | 0,00 | 0,00 | NAN |
| 1,00 | 1,00 | 0,00 | 0,00 |
| 0,00 | 0,00 | 0,00 | NAN |
| 0,00 | 0,00 | 0,00 | 0,00 |
| 0,00 | 1,00 | 1,00 | NAN |
| 1,00 | 0,00 | 0,00 | 0,00 |
| 1,00 | 1,00 | 0,00 | 0,00 |
| 0,00 | 0,00 | 0,00 | 0,00 |
| 0,00 | 0,00 | 0,00 | NAN |
| 0,00 | 0,00 | 0,00 | 0,00 |
| 1,00 | 0,00 | 0,00 | NAN |
| 0,00 | 0,00 | 0,00 | NAN |
| NAN | 0,00 | 0,00 | 0,00 |
| 0,00 | 0,00 | 0,00 | NAN |
| NAN | 1,00 | 1,00 | 0,00 |
| 1,00 | 0,00 | 0,00 | 0,00 |
| 0,00 | 0,00 | 0,00 | NAN |
| NAN | 0,00 | 0,00 | 0,00 |
| 0,00 | 0,00 | 0,00 | 0,00 |
| 1,00 | 0,00 | 0,00 | 0,00 |
| 0,00 | 0,00 | 0,00 | NAN |
| 1,00 | 0,00 | 0,00 | NAN |
| NAN | 0,00 | 0,00 | NAN |
| 1,00 | 0,00 | 0,00 | NAN |
| 1,00 | 0,00 | 0,00 | 0,00 |
| 0,00 | 0,00 | 0,00 | 0,00 |
| 1,00 | 0,00 | 0,00 | 0,00 |
| 0,00 | 0,00 | 0,00 | 0,00 |
| NAN | 0,00 | 0,00 | NAN |
| 0,00 | 0,00 | 0,00 | 0,00 |
| 0,00 | 1,00 | 0,00 | 0,00 |
| 0,00 | 0,00 | 0,00 | NAN |
| 0,00 | 1,00 | 1,00 | 0,00 |
| 0,00 | 0,00 | 0,00 | NAN |
| 0,00 | 0,00 | 0,00 | 0,00 |
| 0,00 | 0,00 | 0,00 | NAN |
| 0,00 | 0,00 | 0,00 | 0,00 |
| NAN | 0,00 | 0,00 | 0,00 |
| 0,00 | 0,00 | 0,00 | 0,00 |
| 0,00 | 1,00 | 1,00 | 0,00 |
| 0,00 | 1,00 | 1,00 | 0,00 |
| 0,00 | 0,00 | 0,00 | 0,00 |
| 0,00 | 0,00 | 0,00 | NAN |
| 0,00 | 0,00 | 0,00 | NAN |
| NAN | 1,00 | 1,00 | NAN |
| 1,00 | 0,00 | 0,00 | 0,00 |
| 1,00 | 0,00 | 0,00 | NAN |
| NAN | 0,00 | 0,00 | NAN |
| 1,00 | 0,00 | 0,00 | 0,00 |

| | | | |
|---|---|---|---|
| 0,00 | 0,00 | 0,00 | NAN |
| 0,00 | 0,00 | 0,00 | 0,00 |
| 1,00 | 1,00 | 1,00 | 1,00 |
| NAN | 1,00 | 1,00 | 0,00 |
| NAN | 0,00 | 0,00 | 0,00 |
| 0,00 | 0,00 | 0,00 | NAN |
| 0,00 | 0,00 | 0,00 | 0,00 |
| 0,00 | 0,00 | 0,00 | NAN |
| 0,00 | 0,00 | 0,00 | 0,00 |
| NAN | 0,00 | 0,00 | 0,00 |
| 0,00 | 1,00 | 1,00 | NAN |
| NAN | 0,00 | 0,00 | 0,00 |
| 0,00 | 0,00 | 0,00 | NAN |
| 0,00 | 1,00 | 1,00 | 0,00 |
| NAN | 0,00 | 0,00 | 0,00 |
| NAN | 0,00 | 0,00 | 0,00 |
| NAN | 0,00 | 0,00 | NAN |
| NAN | 0,00 | 0,00 | 0,00 |
| 0,00 | 1,00 | 0,00 | 0,00 |
| NAN | 0,00 | 0,00 | 0,00 |
| NAN | 0,00 | 0,00 | NAN |
| 0,00 | 0,00 | 0,00 | NAN |
| 0,00 | 0,00 | 0,00 | 0,00 |
| 1,00 | 0,00 | 0,00 | NAN |
| NAN | 0,00 | 0,00 | 0,00 |
| NAN | 0,00 | 0,00 | NAN |
| NAN | 0,00 | 0,00 | 0,00 |
| 0,00 | 0,00 | 0,00 | 0,00 |
| 0,00 | 0,00 | 0,00 | NAN |
| 1,00 | 0,00 | 0,00 | 0,00 |
| 0,00 | 0,00 | 0,00 | 0,00 |
| NAN | 1,00 | 1,00 | 1,00 |
| 1,00 | 1,00 | 1,00 | 0,00 |
| 0,00 | 0,00 | 0,00 | 0,00 |
| NAN | 0,00 | 0,00 | 0,00 |
| NAN | 0,00 | 0,00 | NAN |
| NAN | 0,00 | 0,00 | NAN |
| 0,00 | 0,00 | 0,00 | 0,00 |
| 1,00 | 0,00 | 0,00 | 0,00 |
| NAN | 0,00 | 0,00 | 0,00 |
| NAN | 0,00 | 0,00 | 0,00 |
| 0,00 | 0,00 | 0,00 | 0,00 |
| NAN | 0,00 | 0,00 | 0,00 |
| 0,00 | 0,00 | 0,00 | 0,00 |
| NAN | 0,00 | 0,00 | NAN |
| NAN | 0,00 | 0,00 | 0,00 |
| 0,00 | 1,00 | 1,00 | NAN |
| NAN | 0,00 | 0,00 | NAN |
| 0,00 | 0,00 | 0,00 | 0,00 |
| NAN | 1,00 | 1,00 | 0,00 |

| | | | |
|---|---|---|---|
| 0,00 | 0,00 | 0,00 | 0,00 |
| 0,00 | 0,00 | 0,00 | 1,00 |
| NAN | 0,00 | 0,00 | NAN |
| 0,00 | 0,00 | 0,00 | 0,00 |
| NAN | 0,00 | 0,00 | 0,00 |
| 0,00 | 0,00 | 0,00 | 0,00 |
| 1,00 | 0,00 | 0,00 | 0,00 |
| 0,00 | 0,00 | 0,00 | 0,00 |
| NAN | 0,00 | 0,00 | 0,00 |
| 0,00 | 1,00 | 1,00 | NAN |
| NAN | 1,00 | 1,00 | 0,00 |
| NAN | 1,00 | 1,00 | NAN |
| NAN | 0,00 | 0,00 | 0,00 |
| NAN | 1,00 | 1,00 | NAN |
| 0,00 | 0,00 | 0,00 | 0,00 |
| 1,00 | 1,00 | 1,00 | 0,00 |

| Prediabetes (A1c>5.7%) 2 yrs PD | sBP>140 mm Hg 2 Yrs PD | dBP >80 mm Hg 2 Yrs PD | TG > 150 mg/dl 2 yrs PD |
|---|---|---|---|
| 0,00 | 0,00 | 0,00 | 0,00 |
| 0,00 | 0,00 | 0,00 | 0,00 |
| 0,00 | NAN | NAN | 0,00 |
| 0,00 | NAN | NAN | 1,00 |
| 0,00 | 0,00 | 0,00 | 0,00 |
| 0,00 | 0,00 | 0,00 | 0,00 |
| 0,00 | NAN | NAN | 0,00 |
| 0,00 | 0,00 | 0,00 | 0,00 |
| NAN | 0,00 | 0,00 | 0,00 |
| 1,00 | NAN | NAN | 1,00 |
| 0,00 | 0,00 | 0,00 | 0,00 |
| 0,00 | 0,00 | 0,00 | 0,00 |
| 0,00 | NAN | NAN | 0,00 |
| 0,00 | 0,00 | 0,00 | 0,00 |
| 0,00 | 0,00 | 0,00 | 0,00 |
| 0,00 | 0,00 | 0,00 | 0,00 |
| 0,00 | NAN | NAN | 0,00 |
| 0,00 | 0,00 | 0,00 | 0,00 |
| 0,00 | 0,00 | 0,00 | 0,00 |
| 1,00 | 0,00 | 0,00 | 0,00 |
| 0,00 | 0,00 | 0,00 | 0,00 |
| 0,00 | 0,00 | 0,00 | 0,00 |
| NAN | NAN | NAN | 1,00 |
| 0,00 | 0,00 | 0,00 | 0,00 |
| 0,00 | 0,00 | 0,00 | 0,00 |
| 0,00 | NAN | NAN | 0,00 |
| 0,00 | 0,00 | 0,00 | 1,00 |
| 0,00 | 0,00 | 0,00 | 1,00 |
| 0,00 | 0,00 | 0,00 | 0,00 |
| 0,00 | 0,00 | 0,00 | 0,00 |
| 0,00 | 0,00 | 0,00 | 0,00 |
| 0,00 | 0,00 | 0,00 | 0,00 |
| 1,00 | 0,00 | 0,00 | 0,00 |
| 0,00 | 0,00 | 0,00 | 0,00 |
| 0,00 | NAN | NAN | 0,00 |
| 0,00 | 0,00 | 0,00 | 0,00 |
| 0,00 | 0,00 | 0,00 | 0,00 |
| 0,00 | 0,00 | 0,00 | 0,00 |
| 0,00 | NAN | NAN | 0,00 |
| 0,00 | NAN | NAN | 0,00 |
| 0,00 | NAN | NAN | 0,00 |
| 0,00 | 0,00 | 0,00 | 1,00 |
| 0,00 | NAN | NAN | 0,00 |
| 0,00 | 0,00 | 0,00 | 0,00 |
| 0,00 | NAN | NAN | 0,00 |
| 0,00 | 0,00 | 0,00 | 0,00 |
| 0,00 | 0,00 | 0,00 | 0,00 |

| | | | |
|---|---|---|---|
| 1,00 | 0,00 | 0,00 | 0,00 |
| 0,00 | 0,00 | 0,00 | 0,00 |
| 0,00 | 0,00 | 1,00 | 0,00 |
| 0,00 | 0,00 | 1,00 | 0,00 |
| 0,00 | 0,00 | 0,00 | 0,00 |
| 0,00 | 0,00 | 1,00 | 0,00 |
| 0,00 | 0,00 | 0,00 | 0,00 |
| 0,00 | NAN | NAN | 0,00 |
| 0,00 | 0,00 | 0,00 | 0,00 |
| NAN | 0,00 | 0,00 | 0,00 |
| 0,00 | 0,00 | 0,00 | 0,00 |
| 0,00 | NAN | NAN | 0,00 |
| 0,00 | 0,00 | 0,00 | 0,00 |
| 0,00 | 0,00 | 0,00 | 0,00 |
| 0,00 | 0,00 | 0,00 | 1,00 |
| 0,00 | NAN | NAN | 0,00 |
| 0,00 | 0,00 | 0,00 | 0,00 |
| 0,00 | 0,00 | 0,00 | 0,00 |
| 0,00 | 0,00 | 1,00 | 0,00 |
| 0,00 | 0,00 | 0,00 | 0,00 |
| 0,00 | 0,00 | 0,00 | 0,00 |
| 0,00 | 0,00 | 0,00 | 0,00 |
| 1,00 | 0,00 | 0,00 | 1,00 |
| 0,00 | 0,00 | 1,00 | 0,00 |
| 0,00 | NAN | NAN | 0,00 |
| 0,00 | NAN | NAN | 0,00 |
| 0,00 | 0,00 | 0,00 | 0,00 |
| 0,00 | NAN | NAN | 0,00 |
| 0,00 | 0,00 | 0,00 | 0,00 |
| 0,00 | 0,00 | 0,00 | 0,00 |
| 0,00 | 0,00 | 0,00 | 0,00 |
| 0,00 | 0,00 | 0,00 | 0,00 |
| 0,00 | 0,00 | 0,00 | 0,00 |
| 0,00 | NAN | NAN | 0,00 |
| 0,00 | 0,00 | 0,00 | 0,00 |
| 0,00 | 0,00 | 0,00 | 0,00 |
| 0,00 | 0,00 | 0,00 | 0,00 |
| 1,00 | 0,00 | 0,00 | 0,00 |
| 0,00 | 0,00 | 0,00 | 0,00 |
| 0,00 | 0,00 | 0,00 | 0,00 |
| 0,00 | 0,00 | 0,00 | 0,00 |
| 0,00 | 0,00 | 0,00 | 0,00 |
| 0,00 | 0,00 | 1,00 | 1,00 |
| 0,00 | 0,00 | 0,00 | 0,00 |
| 0,00 | 0,00 | 0,00 | 0,00 |
| 0,00 | NAN | NAN | 0,00 |
| 0,00 | 0,00 | 0,00 | 0,00 |
| 0,00 | 0,00 | 1,00 | 0,00 |
| 0,00 | NAN | NAN | 0,00 |
| 0,00 | 0,00 | 0,00 | 0,00 |

| | | | |
|---|---|---|---|
| NAN | 0,00 | 0,00 | 0,00 |
| 0,00 | 0,00 | 0,00 | 1,00 |
| 0,00 | 0,00 | 1,00 | 0,00 |
| 0,00 | 0,00 | 0,00 | 0,00 |
| 0,00 | 0,00 | 0,00 | 0,00 |
| 0,00 | 0,00 | 0,00 | 0,00 |
| 0,00 | NAN | NAN | 0,00 |
| 0,00 | 0,00 | 0,00 | 0,00 |
| 0,00 | NAN | NAN | 1,00 |
| 0,00 | NAN | NAN | 0,00 |
| 0,00 | NAN | NAN | 0,00 |
| 0,00 | 0,00 | 0,00 | 0,00 |
| 0,00 | 0,00 | 0,00 | 0,00 |
| 0,00 | 0,00 | 0,00 | 0,00 |
| 0,00 | 0,00 | 0,00 | 0,00 |
| 0,00 | 0,00 | 0,00 | 0,00 |
| 0,00 | 0,00 | 0,00 | 0,00 |
| 0,00 | NAN | NAN | 0,00 |
| 0,00 | NAN | NAN | 0,00 |
| 0,00 | NAN | NAN | 0,00 |
| 0,00 | 0,00 | 0,00 | 0,00 |
| 0,00 | 0,00 | 0,00 | 0,00 |
| 0,00 | NAN | NAN | 0,00 |
| 0,00 | 0,00 | 0,00 | 0,00 |
| 0,00 | 0,00 | 0,00 | 1,00 |
| 0,00 | 0,00 | 0,00 | 0,00 |
| 0,00 | 0,00 | 0,00 | 0,00 |
| 0,00 | NAN | NAN | 0,00 |
| 1,00 | 0,00 | 0,00 | 0,00 |
| 0,00 | 0,00 | 0,00 | 0,00 |
| 0,00 | 0,00 | 0,00 | 0,00 |
| 1,00 | NAN | NAN | 0,00 |
| 0,00 | 0,00 | 0,00 | 0,00 |
| 0,00 | NAN | NAN | 0,00 |
| 1,00 | NAN | NAN | 1,00 |
| 0,00 | NAN | NAN | 0,00 |
| 0,00 | 0,00 | 1,00 | 0,00 |
| 0,00 | NAN | NAN | 0,00 |
| 0,00 | 0,00 | 1,00 | 0,00 |
| 0,00 | NAN | NAN | 0,00 |
| 0,00 | NAN | NAN | 0,00 |
| 1,00 | 0,00 | 1,00 | 1,00 |
| 0,00 | 0,00 | 1,00 | 0,00 |
| 0,00 | NAN | NAN | 0,00 |
| 0,00 | 0,00 | 1,00 | 0,00 |
| 0,00 | 0,00 | 0,00 | 0,00 |
| 0,00 | 0,00 | 0,00 | 0,00 |
| 0,00 | 0,00 | 0,00 | 0,00 |
| 0,00 | 0,00 | 0,00 | 0,00 |
| 0,00 | 0,00 | 0,00 | 0,00 |

| | | | |
|---|---|---|---|
| 0,00 | NAN | NAN | 0,00 |
| 0,00 | 0,00 | 0,00 | 0,00 |
| 0,00 | 0,00 | 1,00 | 0,00 |
| 0,00 | 0,00 | 0,00 | 0,00 |
| 1,00 | 0,00 | 0,00 | 0,00 |
| 0,00 | 0,00 | 0,00 | 0,00 |
| 0,00 | 0,00 | 0,00 | 0,00 |
| 0,00 | 0,00 | 0,00 | 0,00 |
| 0,00 | 0,00 | 0,00 | 0,00 |
| 0,00 | 0,00 | 0,00 | 0,00 |
| 0,00 | NAN | NAN | 0,00 |
| 0,00 | 0,00 | 0,00 | 0,00 |
| 0,00 | 0,00 | 0,00 | 0,00 |
| 0,00 | 0,00 | 0,00 | 0,00 |
| 0,00 | 0,00 | 1,00 | 0,00 |
| 0,00 | NAN | NAN | 0,00 |
| 0,00 | 0,00 | 0,00 | 0,00 |
| 0,00 | 0,00 | 0,00 | 0,00 |
| 0,00 | 0,00 | 0,00 | 0,00 |
| 0,00 | 0,00 | 0,00 | 0,00 |
| 0,00 | 0,00 | 0,00 | 0,00 |
| 0,00 | 0,00 | 0,00 | 0,00 |
| 0,00 | NAN | NAN | 1,00 |
| 1,00 | 0,00 | 0,00 | 0,00 |
| 0,00 | NAN | NAN | 0,00 |
| 1,00 | 0,00 | 0,00 | 0,00 |
| 1,00 | 0,00 | 0,00 | 0,00 |
| 0,00 | 0,00 | 1,00 | 0,00 |
| 0,00 | 0,00 | 1,00 | 0,00 |
| 0,00 | NAN | NAN | 0,00 |
| NAN | NAN | NAN | 0,00 |
| 0,00 | 0,00 | 0,00 | 0,00 |
| 0,00 | 0,00 | 0,00 | 0,00 |
| 0,00 | 0,00 | 0,00 | 0,00 |
| 0,00 | 0,00 | 0,00 | 0,00 |
| 0,00 | 0,00 | 0,00 | 0,00 |
| 0,00 | 0,00 | 0,00 | 0,00 |
| 0,00 | NAN | NAN | 0,00 |
| 0,00 | 0,00 | 0,00 | 0,00 |
| 0,00 | NAN | NAN | 0,00 |
| 0,00 | NAN | NAN | 0,00 |
| 0,00 | 0,00 | 0,00 | 0,00 |
| 1,00 | NAN | NAN | 0,00 |
| 1,00 | 0,00 | 0,00 | 0,00 |
| 0,00 | 1,00 | 0,00 | 0,00 |
| 0,00 | 0,00 | 0,00 | 0,00 |
| 0,00 | NAN | NAN | 0,00 |
| 0,00 | 0,00 | 0,00 | 0,00 |
| 0,00 | 0,00 | 0,00 | 0,00 |
| 0,00 | NAN | NAN | 0,00 |

| | | | |
|---|---|---|---|
| 0,00 | 0,00 | 0,00 | 0,00 |
| 0,00 | 0,00 | 1,00 | 0,00 |
| 0,00 | 0,00 | 0,00 | 0,00 |
| 0,00 | 0,00 | 0,00 | 0,00 |
| 0,00 | 0,00 | 0,00 | 0,00 |
| 0,00 | 0,00 | 0,00 | 0,00 |
| 0,00 | 0,00 | 0,00 | 0,00 |
| 1,00 | 0,00 | 0,00 | 0,00 |
| 0,00 | 0,00 | 0,00 | 0,00 |
| 0,00 | 0,00 | 0,00 | 0,00 |
| 0,00 | NAN | NAN | 0,00 |
| 0,00 | 0,00 | 0,00 | 0,00 |
| 0,00 | 0,00 | 0,00 | 0,00 |
| 0,00 | NAN | NAN | 0,00 |
| 0,00 | 0,00 | 1,00 | 0,00 |
| 0,00 | NAN | NAN | 0,00 |
| 0,00 | 0,00 | 0,00 | 0,00 |
| 0,00 | NAN | NAN | 0,00 |
| 0,00 | NAN | NAN | 0,00 |
| 0,00 | 0,00 | 0,00 | 0,00 |
| 0,00 | 0,00 | 0,00 | 0,00 |
| 0,00 | 0,00 | 0,00 | 0,00 |
| 0,00 | 0,00 | 0,00 | 0,00 |
| NAN | 0,00 | 0,00 | 0,00 |
| 0,00 | NAN | NAN | 0,00 |
| 0,00 | 0,00 | 0,00 | 0,00 |
| 0,00 | 0,00 | 0,00 | 0,00 |
| 0,00 | 0,00 | 0,00 | 0,00 |
| 0,00 | 0,00 | 1,00 | 0,00 |
| 0,00 | NAN | NAN | 0,00 |
| 0,00 | 0,00 | 1,00 | 0,00 |
| 0,00 | 0,00 | 0,00 | 0,00 |
| 0,00 | 0,00 | 0,00 | 0,00 |
| 0,00 | 0,00 | 0,00 | 0,00 |
| 0,00 | 0,00 | 0,00 | 0,00 |
| 0,00 | 0,00 | 0,00 | 0,00 |
| 0,00 | 0,00 | 0,00 | 0,00 |
| 0,00 | 0,00 | 0,00 | 0,00 |
| 0,00 | 0,00 | 0,00 | 0,00 |
| 0,00 | 0,00 | 1,00 | 0,00 |
| 0,00 | 0,00 | 0,00 | 0,00 |
| 0,00 | 0,00 | 0,00 | 0,00 |
| 0,00 | 0,00 | 0,00 | 0,00 |
| 0,00 | 0,00 | 0,00 | 0,00 |
| 1,00 | 0,00 | 0,00 | 0,00 |
| 0,00 | NAN | NAN | 0,00 |
| 0,00 | 0,00 | 0,00 | 0,00 |
| 0,00 | NAN | NAN | 1,00 |
| 0,00 | NAN | NAN | 0,00 |
| 1,00 | 0,00 | 0,00 | 0,00 |

| | | | |
|---|---|---|---|
| 0,00 | 0,00 | 0,00 | 0,00 |
| 0,00 | 0,00 | 0,00 | 0,00 |
| 1,00 | NAN | NAN | 0,00 |
| 0,00 | NAN | NAN | 0,00 |
| 0,00 | 0,00 | 0,00 | 0,00 |
| 0,00 | 0,00 | 0,00 | 0,00 |
| 0,00 | 0,00 | 0,00 | 0,00 |
| 0,00 | 0,00 | 0,00 | 0,00 |
| 0,00 | 0,00 | 0,00 | 0,00 |
| 0,00 | 0,00 | 1,00 | 0,00 |
| 0,00 | 0,00 | 0,00 | 0,00 |
| 0,00 | 0,00 | 0,00 | 0,00 |
| 0,00 | 0,00 | 0,00 | 0,00 |
| 0,00 | 0,00 | 0,00 | 0,00 |
| 0,00 | 0,00 | 1,00 | 0,00 |
| 0,00 | 0,00 | 0,00 | 1,00 |
| 0,00 | NAN | NAN | 0,00 |
| 1,00 | 0,00 | 0,00 | 0,00 |
| 0,00 | NAN | NAN | 0,00 |
| 0,00 | 0,00 | 0,00 | 0,00 |
| 0,00 | 0,00 | 0,00 | 0,00 |
| 0,00 | 0,00 | 0,00 | 0,00 |
| 0,00 | 0,00 | 0,00 | 0,00 |
| 0,00 | 0,00 | 0,00 | 0,00 |
| 0,00 | NAN | NAN | 0,00 |
| 0,00 | NAN | NAN | 0,00 |
| 0,00 | 0,00 | 1,00 | 0,00 |
| 0,00 | 0,00 | 0,00 | 0,00 |
| 0,00 | 0,00 | 0,00 | 0,00 |
| 0,00 | 0,00 | 0,00 | 0,00 |
| 0,00 | 0,00 | 1,00 | 0,00 |
| 1,00 | NAN | NAN | 1,00 |
| 0,00 | 0,00 | 0,00 | 0,00 |
| 0,00 | 0,00 | 0,00 | 0,00 |
| 0,00 | NAN | NAN | 0,00 |
| 0,00 | NAN | NAN | 0,00 |
| 0,00 | NAN | NAN | 0,00 |
| 0,00 | 0,00 | 0,00 | 0,00 |
| 0,00 | 0,00 | 0,00 | 0,00 |
| 0,00 | NAN | NAN | 0,00 |
| 0,00 | 0,00 | 0,00 | 0,00 |
| 1,00 | 0,00 | 0,00 | 0,00 |
| 0,00 | NAN | NAN | 0,00 |
| 0,00 | 0,00 | 0,00 | 0,00 |
| 0,00 | NAN | NAN | 0,00 |
| 0,00 | NAN | NAN | 0,00 |
| 0,00 | 0,00 | 0,00 | 0,00 |
| 0,00 | NAN | NAN | 0,00 |
| 0,00 | 0,00 | 0,00 | 0,00 |
| 0,00 | NAN | NAN | 0,00 |

| | | | |
|---|---|---|---|
| 0,00 | 0,00 | 0,00 | 0,00 |
| 0,00 | 0,00 | 0,00 | 1,00 |
| 0,00 | 0,00 | 1,00 | 0,00 |
| 1,00 | 0,00 | 0,00 | 0,00 |
| 1,00 | NAN | NAN | 0,00 |
| 0,00 | NAN | NAN | 0,00 |
| 0,00 | 0,00 | 1,00 | 0,00 |
| 0,00 | 0,00 | 0,00 | 0,00 |
| 0,00 | 0,00 | 0,00 | 0,00 |
| 0,00 | 1,00 | 1,00 | 1,00 |
| 1,00 | NAN | NAN | 1,00 |
| 0,00 | 0,00 | 0,00 | 0,00 |
| 0,00 | NAN | NAN | 0,00 |
| 0,00 | NAN | NAN | 0,00 |
| 0,00 | 0,00 | 0,00 | 0,00 |
| 0,00 | NAN | NAN | 0,00 |

| HDL <45 mg/dl 2 Yrs PD | Metabolic Syndrome 2 yrs PD | Age (years) | Ethnicity | Pre-pregnancy self-reported body Weight (kg) |
|---|---|---|---|---|
| 0,00 | NAN | 39 | 1 | 53,00 |
| 0,00 | NAN | 38 | 1 | 57,00 |
| 0,00 | NAN | 44 | 1 | 67,00 |
| 1,00 | NAN | 33 | 1 | 75,00 |
| 0,00 | NAN | 31 | 1 | 58,50 |
| 1,00 | NAN | 20 | 2 | 43,00 |
| 0,00 | NAN | 40 | 1 | 52,00 |
| 0,00 | NAN | 30 | 1 | 70,00 |
| 0,00 | 0,00 | 35 | 1 | 58,00 |
| 1,00 | NAN | 31 | 2 | 78,00 |
| 0,00 | 0,00 | 39 | 2 | 39,00 |
| 0,00 | 1,00 | 35 | 1 | 80,00 |
| 0,00 | NAN | 28 | 1 | 50,00 |
| 0,00 | NAN | 30 | 2 | 96,00 |
| 0,00 | NAN | 34 | 1 | 54,00 |
| 0,00 | NAN | 33 | 1 | 51,00 |
| 1,00 | NAN | 31 | 1 | 54,00 |
| 1,00 | 0,00 | 30 | 1 | 54,00 |
| 0,00 | 0,00 | 38 | 1 | 63,00 |
| NAN | NAN | 39 | 1 | 71,00 |
| 0,00 | NAN | 24 | 1 | 46,10 |
| 1,00 | NAN | 39 | 2 | 56,00 |
| 0,00 | NAN | 37 | 2 | 72,00 |
| 0,00 | 0,00 | 37 | 1 | 59,00 |
| 1,00 | 1,00 | 40 | 1 | 74,00 |
| 0,00 | NAN | 29 | 3 | 58,00 |
| 0,00 | 0,00 | 31 | 1 | 55,00 |
| 0,00 | NAN | 28 | 1 | 70,00 |
| 0,00 | 0,00 | 35 | 1 | 47,00 |
| 0,00 | NAN | 35 | 1 | 59,00 |
| 0,00 | 1,00 | 31 | 1 | 71,00 |
| 0,00 | NAN | 31 | 1 | 58,00 |
| 0,00 | 0,00 | 33 | 2 | 52,00 |
| 1,00 | 0,00 | 32 | 1 | 76,00 |
| 0,00 | NAN | 38 | 1 | 55,00 |
| 0,00 | 0,00 | 27 | 2 | 57,00 |
| 0,00 | NAN | 35 | 1 | 48,00 |
| 1,00 | 1,00 | 28 | 2 | 80,00 |
| 1,00 | NAN | 37 | 2 | 64,00 |
| 0,00 | NAN | 33 | 1 | 74,00 |
| 1,00 | NAN | 30 | 1 | 70,00 |
| 0,00 | 1,00 | 36 | 2 | 60,00 |
| 0,00 | NAN | 30 | 2 | 63,00 |
| 0,00 | 0,00 | 34 | 1 | 43,00 |
| 0,00 | NAN | 36 | 1 | 61,50 |
| 0,00 | 0,00 | 39 | 1 | 62,00 |
| 0,00 | 0,00 | 25 | 1 | 42,00 |

| | | | | |
|---|---|---|---|---|
| 0,00 | NAN | 30 | 2 | 56,00 |
| 0,00 | 0,00 | 39 | 1 | 66,00 |
| 1,00 | 0,00 | 25 | 2 | 56,50 |
| 0,00 | NAN | 30 | 1 | 69,50 |
| 0,00 | NAN | 37 | 1 | 48,00 |
| 0,00 | 0,00 | 34 | 2 | 60,00 |
| 1,00 | 0,00 | 41 | 2 | 61,00 |
| 0,00 | NAN | 42 | 1 | 53,50 |
| 0,00 | NAN | 29 | 1 | 69,00 |
| 0,00 | NAN | 26 | 1 | 60,00 |
| 0,00 | 0,00 | 34 | 2 | 69,50 |
| 0,00 | NAN | 35 | 1 | 53,50 |
| 0,00 | NAN | 32 | 2 | 75,00 |
| 0,00 | NAN | 34 | 1 | 51,00 |
| 1,00 | 1,00 | 30 | 2 | 62,00 |
| 0,00 | NAN | 34 | 2 | 64,00 |
| 0,00 | NAN | 32 | 1 | 69,00 |
| 0,00 | NAN | 38 | 1 | 56,00 |
| 1,00 | 1,00 | 33 | 1 | 59,00 |
| 0,00 | 0,00 | 24 | 2 | 48,00 |
| 1,00 | NAN | 37 | 2 | 55,00 |
| 0,00 | NAN | 37 | 1 | 55,00 |
| 1,00 | NAN | 43 | 2 | 60,00 |
| 0,00 | NAN | 31 | 1 | 50,50 |
| 0,00 | NAN | 24 | 2 | 117,00 |
| 0,00 | NAN | 27 | 2 | 70,00 |
| 0,00 | NAN | 35 | 1 | 53,50 |
| 0,00 | NAN | 35 | 1 | 78,00 |
| 0,00 | NAN | 26 | 1 | 58,00 |
| 0,00 | NAN | 28 | 2 | 63,00 |
| 0,00 | NAN | 38 | 1 | 60,00 |
| 0,00 | 0,00 | 41 | 2 | 62,00 |
| 0,00 | 0,00 | 38 | 1 | 64,00 |
| 0,00 | NAN | 36 | 1 | 70,00 |
| 0,00 | NAN | 30 | 2 | 55,00 |
| 0,00 | NAN | 34 | 1 | 48,50 |
| 0,00 | 0,00 | 30 | 2 | 53,00 |
| 0,00 | NAN | 33 | 2 | 50,00 |
| 0,00 | 0,00 | 40 | 2 | 68,00 |
| 0,00 | NAN | 30 | 3 | 49,50 |
| 0,00 | NAN | 33 | 1 | 50,00 |
| 0,00 | 1,00 | 37 | 2 | 63,00 |
| 0,00 | NAN | 39 | 3 | 74,00 |
| 0,00 | NAN | 39 | 1 | 52,50 |
| 0,00 | NAN | 31 | 1 | 54,00 |
| 0,00 | NAN | 38 | 1 | 61,00 |
| 0,00 | 0,00 | 33 | 1 | 60,00 |
| 0,00 | NAN | 42 | 2 | 55,50 |
| 0,00 | NAN | 35 | 1 | 60,50 |
| 0,00 | NAN | 28 | 3 | 46,00 |

| | | | | |
|---|---|---|---|---|
| 0,00 | 0,00 | 34 | 1 | 52,00 |
| 0,00 | NAN | 21 | 2 | 43,00 |
| 0,00 | NAN | 25 | 3 | 58,00 |
| 1,00 | 0,00 | 26 | 2 | 49,00 |
| 0,00 | 0,00 | 33 | 2 | 60,00 |
| 0,00 | 0,00 | 22 | 2 | 50,00 |
| 0,00 | NAN | 30 | 1 | 63,00 |
| 0,00 | NAN | 40 | 1 | 55,00 |
| 0,00 | NAN | 30 | 2 | 50,00 |
| 0,00 | NAN | 36 | 1 | 63,50 |
| 0,00 | NAN | 26 | 2 | 56,00 |
| 0,00 | NAN | 36 | 3 | 67,00 |
| 0,00 | NAN | 35 | 1 | 55,00 |
| 0,00 | 0,00 | 38 | 1 | 57,00 |
| 0,00 | 0,00 | 35 | 1 | 65,00 |
| 0,00 | NAN | 35 | 2 | 62,00 |
| 0,00 | 0,00 | 35 | 1 | 50,00 |
| 0,00 | NAN | 37 | 1 | 45,00 |
| 0,00 | NAN | 35 | 1 | 51,00 |
| 0,00 | NAN | 35 | 1 | 55,00 |
| 0,00 | NAN | 40 | 1 | 52,00 |
| 0,00 | 0,00 | 39 | 1 | 77,00 |
| 0,00 | NAN | 36 | 1 | 58,00 |
| 0,00 | NAN | 26 | 2 | 56,00 |
| 1,00 | 1,00 | 38 | 2 | 68,00 |
| NAN | NAN | 37 | 1 | 44,50 |
| 0,00 | 0,00 | 36 | 1 | 49,00 |
| 0,00 | NAN | 41 | 2 | 58,00 |
| 1,00 | 1,00 | 34 | 2 | 65,00 |
| 0,00 | 0,00 | 32 | 1 | 53,00 |
| 0,00 | 0,00 | 43 | 1 | 85,00 |
| 0,00 | NAN | 40 | 2 | 60,00 |
| 0,00 | 0,00 | 40 | 1 | 56,00 |
| 0,00 | NAN | 39 | 1 | 55,00 |
| 0,00 | NAN | 34 | 2 | 58,00 |
| 0,00 | NAN | 36 | 1 | 54,00 |
| 0,00 | 1,00 | 37 | 2 | 76,00 |
| 0,00 | NAN | 31 | 2 | 57,00 |
| 0,00 | NAN | 35 | 1 | 60,00 |
| 0,00 | NAN | 32 | 1 | 85,00 |
| 0,00 | NAN | 36 | 1 | 67,00 |
| 0,00 | 1,00 | 25 | 1 | 73,00 |
| 0,00 | 0,00 | 35 | 1 | 67,00 |
| 0,00 | NAN | 32 | 1 | 54,00 |
| 0,00 | NAN | 36 | 1 | 80,00 |
| 0,00 | 0,00 | 41 | 1 | 58,00 |
| 0,00 | NAN | 36 | 1 | 60,00 |
| 0,00 | 0,00 | 34 | 2 | 55,00 |
| 0,00 | 0,00 | 31 | 1 | 67,00 |
| 0,00 | 0,00 | 32 | 1 | 75,00 |

| | | | | |
|---|---|---|---|---|
| 0,00 | NAN | 38 | 3 | 46,00 |
| 0,00 | 0,00 | 32 | 1 | 62,00 |
| 0,00 | 0,00 | 37 | 1 | 58,00 |
| 0,00 | 0,00 | 36 | 1 | 50,00 |
| 0,00 | 0,00 | 44 | 1 | 63,50 |
| 0,00 | 0,00 | 31 | 1 | 60,00 |
| 0,00 | 0,00 | 37 | 1 | 54,00 |
| 0,00 | NAN | 33 | 1 | 55,00 |
| 0,00 | NAN | 25 | 2 | 60,00 |
| 0,00 | 0,00 | 36 | 1 | 60,00 |
| 1,00 | NAN | 36 | 2 | 72,00 |
| 0,00 | 0,00 | 34 | 1 | 56,00 |
| 0,00 | 0,00 | 28 | 1 | 53,00 |
| 0,00 | 1,00 | 30 | 1 | 54,00 |
| 0,00 | 0,00 | 23 | 1 | 49,50 |
| 0,00 | NAN | 42 | 1 | 95,00 |
| 0,00 | 0,00 | 37 | 2 | 62,60 |
| 0,00 | NAN | 36 | 1 | 62,00 |
| 0,00 | 0,00 | 40 | 2 | 70,00 |
| 0,00 | NAN | 40 | 1 | 61,00 |
| 0,00 | 0,00 | 31 | 2 | 66,00 |
| 0,00 | NAN | 39 | 2 | 60,00 |
| 0,00 | NAN | 21 | 2 | 57,00 |
| 0,00 | NAN | 41 | 1 | 49,00 |
| 0,00 | NAN | 35 | 2 | 67,00 |
| 0,00 | NAN | 35 | 1 | 57,00 |
| 1,00 | NAN | 37 | 1 | 55,00 |
| 0,00 | 0,00 | 28 | 1 | 50,00 |
| 0,00 | 1,00 | 33 | 2 | 71,00 |
| 0,00 | NAN | 35 | 2 | 58,40 |
| 0,00 | NAN | 35 | 2 | 47,00 |
| 0,00 | 0,00 | 33 | 2 | 49,50 |
| 0,00 | NAN | 34 | 1 | 62,50 |
| 0,00 | 0,00 | 33 | 1 | 52,00 |
| 0,00 | NAN | 39 | 1 | 63,50 |
| 0,00 | 0,00 | 27 | 1 | 59,00 |
| 0,00 | 0,00 | 35 | 1 | 58,00 |
| 1,00 | NAN | 34 | 3 | 75,00 |
| 1,00 | NAN | 30 | 1 | 65,00 |
| 0,00 | NAN | 31 | 1 | 57,00 |
| 0,00 | NAN | 39 | 1 | 51,50 |
| 0,00 | 0,00 | 38 | 1 | 75,00 |
| 0,00 | NAN | 43 | 1 | 55,00 |
| 0,00 | 0,00 | 38 | 1 | 58,00 |
| 0,00 | 0,00 | 34 | 1 | 71,00 |
| 0,00 | 0,00 | 26 | 2 | 54,00 |
| 0,00 | NAN | 34 | 2 | 63,00 |
| 0,00 | 0,00 | 36 | 1 | 53,00 |
| 0,00 | NAN | 35 | 1 | 49,50 |
| 0,00 | NAN | 35 | 1 | 56,00 |

| | | | | |
|---|---|---|---|---|
| 0,00 | 0,00 | 42 | 1 | 79,00 |
| 0,00 | 0,00 | 36 | 1 | 58,00 |
| 0,00 | NAN | 39 | 2 | 54,50 |
| 0,00 | 0,00 | 26 | 2 | 65,00 |
| 0,00 | NAN | 43 | 2 | 50,50 |
| 0,00 | NAN | 32 | 2 | 60,00 |
| 1,00 | 1,00 | 33 | 1 | 83,00 |
| 0,00 | 1,00 | 32 | 2 | 81,00 |
| 0,00 | 0,00 | 37 | 1 | 59,50 |
| 0,00 | 0,00 | 37 | 1 | 54,00 |
| 0,00 | NAN | 38 | 1 | 57,00 |
| 0,00 | NAN | 27 | 1 | 56,50 |
| 0,00 | NAN | 40 | 1 | 56,00 |
| 0,00 | NAN | 29 | 2 | 79,50 |
| 0,00 | NAN | 33 | 1 | 48,50 |
| 0,00 | NAN | 29 | 1 | 65,00 |
| 0,00 | NAN | 28 | 2 | 56,00 |
| 0,00 | NAN | 20 | 1 | 54,50 |
| 0,00 | NAN | 41 | 1 | 55,50 |
| 0,00 | NAN | 37 | 2 | 83,00 |
| 0,00 | 0,00 | 38 | 2 | 70,00 |
| 0,00 | NAN | 38 | 1 | 48,00 |
| 0,00 | 0,00 | 38 | 1 | 65,50 |
| 0,00 | NAN | 35 | 1 | 54,50 |
| 1,00 | NAN | 32 | 1 | 64,00 |
| 1,00 | 1,00 | 33 | 2 | 62,00 |
| 0,00 | NAN | 42 | 1 | 54,70 |
| 1,00 | NAN | 29 | 1 | 81,50 |
| 0,00 | 0,00 | 33 | 1 | 75,00 |
| 0,00 | NAN | 30 | 1 | 52,00 |
| 0,00 | NAN | 33 | 1 | 51,00 |
| 0,00 | 0,00 | 35 | 1 | 57,00 |
| 0,00 | 0,00 | 37 | 1 | 62,50 |
| 0,00 | 0,00 | 29 | 2 | 58,00 |
| 0,00 | NAN | 35 | 1 | 59,00 |
| 0,00 | NAN | 29 | 2 | 63,50 |
| 0,00 | NAN | 32 | 1 | 59,50 |
| 0,00 | 0,00 | 31 | 2 | 47,00 |
| 0,00 | NAN | 29 | 2 | 64,00 |
| 0,00 | 0,00 | 27 | 2 | 45,00 |
| 0,00 | 0,00 | 34 | 1 | 53,50 |
| 0,00 | 0,00 | 39 | 2 | 55,50 |
| 0,00 | 0,00 | 31 | 2 | 58,00 |
| 0,00 | 0,00 | 32 | 2 | 57,00 |
| 0,00 | NAN | 37 | 1 | 62,50 |
| 0,00 | NAN | 33 | 1 | 57,00 |
| 0,00 | 0,00 | 27 | 2 | 84,00 |
| 1,00 | NAN | 40 | 1 | 59,20 |
| 0,00 | NAN | 39 | 1 | 57,00 |
| 0,00 | 0,00 | 31 | 1 | 66,00 |

| | | | | |
|---|---|---|---|---|
| 0,00 | NAN | 39 | 2 | 54,00 |
| 0,00 | NAN | 38 | 2 | 52,50 |
| 0,00 | NAN | 37 | 1 | 75,80 |
| 0,00 | NAN | 37 | 1 | 72,00 |
| 0,00 | NAN | 29 | 1 | 57,00 |
| 0,00 | 0,00 | 41 | 1 | 60,50 |
| 0,00 | 0,00 | 35 | 1 | 57,00 |
| 1,00 | NAN | 40 | 3 | 44,00 |
| 0,00 | 0,00 | 26 | 1 | 58,00 |
| 0,00 | NAN | 36 | 2 | 67,00 |
| 0,00 | 0,00 | 37 | 1 | 66,00 |
| 0,00 | NAN | 39 | 2 | 59,00 |
| 0,00 | 0,00 | 39 | 1 | 53,00 |
| 0,00 | 0,00 | 43 | 1 | 48,50 |
| 0,00 | NAN | 37 | 2 | 51,50 |
| 0,00 | NAN | 27 | 2 | 67,00 |
| 0,00 | NAN | 30 | 2 | 51,00 |
| 0,00 | NAN | 40 | 2 | 57,00 |
| 0,00 | NAN | 40 | 1 | 60,00 |
| 0,00 | NAN | 34 | 2 | 60,00 |
| 0,00 | NAN | 37 | 1 | 55,00 |
| 0,00 | NAN | 29 | 1 | 57,00 |
| 0,00 | 0,00 | 37 | 1 | 87,00 |
| 0,00 | 0,00 | 32 | 2 | 83,00 |
| 0,00 | NAN | 39 | 1 | 62,50 |
| 0,00 | NAN | 33 | 1 | 60,00 |
| 0,00 | NAN | 28 | 2 | 76,00 |
| 0,00 | NAN | 31 | 2 | 45,00 |
| 0,00 | NAN | 41 | 1 | 55,00 |
| 0,00 | 0,00 | 34 | 2 | 60,00 |
| 0,00 | NAN | 37 | 1 | 53,00 |
| 0,00 | NAN | 33 | 1 | 73,50 |
| 0,00 | 1,00 | 31 | 2 | 61,00 |
| 0,00 | 0,00 | 33 | 1 | 69,50 |
| 0,00 | NAN | 36 | 1 | 60,00 |
| 0,00 | NAN | 35 | 1 | 62,00 |
| 0,00 | NAN | 34 | 1 | 51,00 |
| 0,00 | 0,00 | 35 | 1 | 61,00 |
| 0,00 | 0,00 | 37 | 1 | 77,00 |
| 0,00 | NAN | 39 | 1 | 70,00 |
| 0,00 | NAN | 37 | 2 | 68,00 |
| 0,00 | 0,00 | 34 | 1 | 67,00 |
| 0,00 | NAN | 35 | 1 | 66,00 |
| 0,00 | NAN | 34 | 1 | 58,00 |
| 0,00 | NAN | 36 | 2 | 57,50 |
| 0,00 | NAN | 40 | 1 | 53,00 |
| 0,00 | NAN | 36 | 1 | 51,00 |
| 0,00 | NAN | 36 | 1 | 70,00 |
| 0,00 | NAN | 34 | 1 | 53,00 |
| 0,00 | NAN | 23 | 2 | 81,00 |

| | | | | |
|---|---|---|---|---|
| 0,00 | 0,00 | 30 | 2 | 66,00 |
| 0,00 | 0,00 | 43 | 1 | 69,00 |
| 0,00 | NAN | 30 | 1 | 70,00 |
| 0,00 | NAN | 38 | 1 | 58,00 |
| 0,00 | NAN | 35 | 2 | 65,00 |
| 0,00 | NAN | 35 | 1 | 64,00 |
| 0,00 | NAN | 33 | 2 | 89,30 |
| 0,00 | NAN | 36 | 1 | 64,00 |
| 0,00 | NAN | 39 | 1 | 65,00 |
| 1,00 | NAN | 30 | 1 | 72,00 |
| 1,00 | NAN | 35 | 2 | 63,00 |
| 0,00 | NAN | 35 | 2 | 75,00 |
| 0,00 | NAN | 22 | 2 | 51,00 |
| 0,00 | NAN | 33 | 1 | 95,00 |
| 0,00 | 0,00 | 31 | 1 | 52,00 |
| 1,00 | NAN | 39 | 2 | 84,00 |

| Pregnancy Body Weight at entry, 8-12 GW (kg) | Weight gain at 8-12 GW | Pre-pregnancy BMI (kg/m2) | HOMA-IR 12 gw | Fasting Plasma Insulin [illegible] | TSH mcUI/mL 12 gw |
|---|---|---|---|---|---|
| 56,00 | 3,00 | 18,78 | 0,6 | 1,2 | 4,88 |
| 56,00 | -1,00 | 21,19 | 1,5 | 2,9 | 2,25 |
| 71,00 | 4,00 | 23,18 | 1,2 | 2,4 | 1,63 |
| 77,00 | 2,00 | 26,26 | 1,9 | 3,5 | NAN |
| 58,50 | 0,00 | 21,49 | 6,0 | 12,7 | 1,43 |
| 46,00 | 3,00 | 15,79 | 22,7 | 42,8 | 3,10 |
| 50,00 | -2,00 | 21,93 | 0,7 | 1,2 | 1,62 |
| 73,00 | 3,00 | 25,71 | 1,5 | 2,8 | 4,39 |
| 63,50 | 5,50 | 23,53 | 3,6 | 6,6 | 1,51 |
| 83,00 | 5,00 | 32,05 | 5,1 | 9,6 | 0,96 |
| 39,50 | 0,50 | 16,88 | 1,4 | 2,8 | 1,43 |
| 86,00 | 6,00 | 27,04 | 3,9 | 8,2 | NAN |
| 51,00 | 1,00 | 20,28 | 0,7 | 1,2 | 1,04 |
| 97,00 | 1,00 | 31,35 | 6,2 | 11,6 | NAN |
| 56,00 | 2,00 | 18,25 | 1,5 | 3,1 | 2,06 |
| 53,00 | 2,00 | 20,69 | 0,9 | 1,6 | 0,57 |
| 52,00 | -2,00 | 19,83 | 2,6 | 5,1 | 0,44 |
| 55,00 | 1,00 | 20,32 | 2,7 | 5,2 | 1,02 |
| 62,50 | -0,50 | 22,32 | 0,7 | 1,4 | 0,14 |
| 73,00 | 2,00 | 26,72 | 2,3 | 4,3 | 2,55 |
| 48,70 | 2,60 | 17,35 | 1,0 | 2,4 | 0,90 |
| 65,00 | 9,00 | 24,89 | 1,7 | 3,2 | 0,15 |
| 71,00 | -1,00 | 27,78 | 1,7 | 3,2 | 1,08 |
| 62,00 | 3,00 | 20,42 | 0,8 | 1,6 | 0,68 |
| 78,00 | 4,00 | 28,55 | 1,8 | 3,4 | 2,00 |
| 58,00 | 0,00 | 21,56 | 0,6 | 1,2 | 3,02 |
| 62,00 | 7,00 | 19,03 | 0,7 | 1,4 | 3,48 |
| 74,00 | 4,00 | 22,86 | 2,0 | 4,4 | NAN |
| 50,00 | 3,00 | 18,36 | 1,8 | 3,4 | 1,46 |
| 61,00 | 2,00 | 20,42 | 1,9 | 4,4 | NAN |
| 69,30 | -1,70 | 28,80 | 2,0 | 3,4 | 0,95 |
| 58,00 | 0,00 | 20,31 | 1,3 | 2,4 | 1,41 |
| 57,00 | 5,00 | 21,10 | 0,6 | 1,4 | 0,31 |
| 78,00 | 2,00 | 26,30 | 1,2 | 2,4 | 2,89 |
| 58,00 | 3,00 | 20,20 | 3,7 | 6,8 | 0,09 |
| 58,80 | 1,80 | 22,55 | 2,4 | 4,8 | 1,90 |
| 49,00 | 1,00 | 18,75 | 0,6 | 1,2 | 1,18 |
| 85,00 | 5,00 | 27,68 | 2,9 | 6,3 | 3,15 |
| 69,00 | 5,00 | 25,64 | 2,0 | 4,2 | 0,43 |
| 67,00 | -7,00 | 27,18 | 10,8 | 26,0 | NAN |
| 71,00 | 1,00 | 28,04 | 2,1 | 4,8 | NAN |
| 62,00 | 2,00 | 25,97 | 1,8 | 3,2 | 1,57 |
| 65,00 | 2,00 | 21,80 | 1,7 | 3,2 | 4,85 |
| 55,00 | 12,00 | 16,80 | 3,4 | 7,3 | 1,28 |
| 68,00 | 6,50 | 20,55 | 1,0 | 1,8 | 1,80 |
| 63,00 | 1,00 | 24,22 | 4,3 | 8,7 | 0,83 |
| 42,00 | 0,00 | 17,48 | 2,5 | 4,8 | 2,95 |

| | | | | | |
|---|---|---|---|---|---|
| 63,00 | 7,00 | 22,72 | 0,7 | 1,4 | 3,38 |
| 66,70 | 0,70 | 24,24 | 6,0 | 12,9 | 3,34 |
| 58,00 | 1,50 | 22,07 | 2,5 | 4,6 | 0,75 |
| 72,00 | 2,50 | 26,16 | 2,4 | 5,2 | 0,04 |
| 50,20 | 2,20 | 19,23 | 1,5 | 3,0 | 0,16 |
| 63,00 | 3,00 | 23,44 | 1,0 | 2,0 | NAN |
| 61,00 | 0,00 | 22,41 | 5,5 | 11,0 | 0,04 |
| 54,00 | 0,50 | 20,39 | 1,1 | 2,4 | NAN |
| 70,20 | 1,20 | 23,88 | 2,1 | 4,1 | 1,92 |
| 62,50 | 2,50 | 24,65 | 7,2 | 17,0 | 1,36 |
| 72,00 | 2,50 | 25,53 | 12,5 | 24,8 | 2,62 |
| 55,00 | 1,50 | 23,78 | 8,3 | 16,8 | 0,58 |
| 75,00 | 0,00 | 28,23 | 1,4 | 2,8 | 3,11 |
| 54,00 | 3,00 | 20,43 | 3,4 | 6,8 | 3,46 |
| 64,00 | 2,00 | 23,34 | 21,6 | 42,1 | 1,00 |
| 64,00 | 0,00 | 25,96 | 15,0 | 31,1 | 2,69 |
| 69,00 | 0,00 | 25,34 | 1,0 | 2,0 | 1,80 |
| 57,00 | 1,00 | 18,71 | 7,5 | 15,7 | 0,94 |
| 63,00 | 4,00 | 24,56 | 7,2 | 16,8 | 0,84 |
| 49,00 | 1,00 | 20,50 | 1,6 | 3,0 | 1,15 |
| 52,00 | -3,00 | 20,45 | 3,5 | 7,8 | NAN |
| 56,00 | 1,00 | 20,20 | 1,1 | 2,1 | 2,16 |
| 62,50 | 2,50 | 23,44 | 1,3 | 2,7 | 1,98 |
| 53,10 | 2,60 | 21,02 | 0,6 | 1,2 | 0,66 |
| 135,90 | 18,90 | 38,64 | 14,5 | 27,4 | NAN |
| 73,60 | 3,60 | 24,22 | 0,6 | 1,4 | 4,74 |
| 57,00 | 3,50 | 21,70 | 7,8 | 14,4 | 1,36 |
| 80,00 | 2,00 | 29,00 | 3,4 | 6,3 | 3,79 |
| 59,50 | 1,50 | 20,55 | 1,4 | 3,3 | 1,04 |
| 63,00 | 0,00 | 23,71 | 0,8 | 1,6 | 0,49 |
| 61,00 | 1,00 | 21,77 | 1,1 | 2,4 | 3,47 |
| 62,00 | 0,00 | 24,84 | 0,6 | 1,2 | 2,41 |
| 65,00 | 1,00 | 22,95 | 1,1 | 2,4 | NAN |
| 74,00 | 4,00 | 24,22 | 0,8 | 1,8 | 1,27 |
| 58,00 | 3,00 | 24,12 | 0,5 | 1,2 | 3,11 |
| 51,00 | 2,50 | 18,48 | 6,4 | 13,7 | 1,87 |
| 52,80 | -0,20 | 22,64 | 2,9 | 7,1 | 2,21 |
| 54,70 | 4,70 | 21,36 | 1,9 | 4,0 | NAN |
| 72,00 | 4,00 | 29,43 | 3,5 | 7,6 | 3,04 |
| 48,00 | -1,50 | 19,58 | 0,5 | 1,2 | NAN |
| 54,50 | 4,50 | 19,53 | 8,3 | 15,4 | NAN |
| 63,00 | 0,00 | 24,92 | 2,1 | 4,0 | 2,34 |
| 79,10 | 5,10 | 31,20 | 2,7 | 6,3 | NAN |
| 55,60 | 3,10 | 20,51 | 0,6 | 1,2 | 1,71 |
| 56,60 | 2,60 | 21,63 | 1,2 | 2,2 | 3,45 |
| 64,60 | 3,60 | 21,11 | 7,7 | 14,6 | 0,47 |
| 64,20 | 4,20 | 20,52 | 0,5 | 1,2 | 1,32 |
| 55,50 | 0,00 | 20,39 | 1,1 | 2,1 | NAN |
| 64,00 | 3,50 | 23,05 | 6,4 | 12,8 | 1,25 |
| 41,70 | -4,30 | 19,91 | 1,4 | 3,0 | 1,12 |

| | | | | | |
|---|---|---|---|---|---|
| 53,30 | 1,30 | 19,10 | 0,7 | 1,4 | 1,26 |
| 46,00 | 3,00 | 14,37 | 1,3 | 2,8 | 3,32 |
| 59,70 | 1,70 | 21,30 | 6,1 | 11,7 | NAN |
| 48,50 | -0,50 | 19,14 | 2,1 | 3,7 | 1,56 |
| 61,50 | 1,50 | 23,44 | 6,8 | 13,0 | 4,54 |
| 50,80 | 0,80 | 22,22 | 0,7 | 1,3 | NAN |
| 65,10 | 2,10 | 23,14 | 3,9 | 7,9 | 2,20 |
| 58,10 | 3,10 | 21,76 | 7,3 | 14,6 | 1,90 |
| 57,40 | 7,40 | NAN | 11,6 | 21,3 | 1,93 |
| 65,60 | 2,10 | 24,80 | 3,8 | 7,0 | 2,68 |
| 60,00 | 4,00 | 21,88 | 1,1 | 2,3 | 1,31 |
| 59,60 | -7,40 | 26,17 | 1,5 | 3,1 | NAN |
| 58,50 | 3,50 | 20,70 | 0,6 | 1,2 | 1,28 |
| 60,00 | 3,00 | 20,94 | 1,1 | 2,0 | 0,15 |
| 66,00 | 1,00 | 27,06 | 2,3 | 4,3 | 1,03 |
| 65,10 | 3,10 | 22,77 | 1,4 | 2,5 | 1,37 |
| 51,50 | 1,50 | 18,82 | 1,7 | 3,5 | 1,21 |
| 46,50 | 1,50 | 19,22 | 2,7 | 5,3 | 0,97 |
| 54,60 | 3,60 | 19,92 | 0,7 | 1,7 | 2,04 |
| 60,00 | 5,00 | 20,20 | 0,8 | 1,8 | 2,22 |
| 55,50 | 3,50 | 19,57 | 4,8 | 10,6 | 1,09 |
| 92,30 | 15,30 | 26,03 | 4,7 | 8,9 | 1,89 |
| 65,30 | 7,30 | 21,30 | 18,9 | 34,1 | 1,83 |
| 57,50 | 1,50 | 23,01 | 1,1 | 1,9 | 2,80 |
| 67,90 | -0,10 | 26,90 | 2,3 | 4,1 | 1,31 |
| 47,50 | 3,00 | 17,83 | 1,1 | 2,4 | 1,15 |
| 53,60 | 4,60 | 17,36 | 12,2 | 27,2 | 2,85 |
| 63,10 | 5,10 | 21,30 | 0,9 | 1,9 | NAN |
| 65,60 | 0,60 | 29,28 | 3,5 | 7,8 | 0,79 |
| 53,90 | 0,90 | 19,95 | 2,7 | 5,2 | 2,28 |
| 85,10 | 0,10 | 28,73 | 4,7 | 9,0 | 3,03 |
| 60,80 | 0,80 | 27,39 | NAN | NAN | 2,77 |
| 59,00 | 3,00 | 18,93 | 0,6 | 1,2 | NAN |
| 55,00 | 0,00 | 22,03 | 1,4 | 3,0 | 3,56 |
| 59,10 | 1,10 | 24,78 | 17,2 | 34,0 | 1,92 |
| 55,60 | 1,60 | 19,83 | 6,2 | 11,8 | 1,66 |
| 75,40 | -0,60 | 30,06 | 1,2 | 2,6 | 0,17 |
| 56,20 | -0,80 | 25,00 | 1,3 | 2,4 | 1,97 |
| 65,80 | 5,80 | 21,26 | 1,3 | 2,5 | 1,06 |
| NAN | NAN | 24,57 | 1,5 | 3,1 | 2,41 |
| 71,10 | 4,10 | 22,91 | 0,7 | 1,4 | 2,33 |
| 78,50 | 5,50 | 27,48 | 1,7 | 3,4 | 1,46 |
| 71,00 | 4,00 | 24,31 | 12,4 | 23,6 | 3,55 |
| 56,00 | 2,00 | 19,36 | 0,6 | 1,2 | 3,27 |
| 83,40 | 3,40 | 28,01 | 8,8 | 19,1 | 1,39 |
| 58,90 | 0,90 | 22,94 | 0,6 | 1,2 | 1,96 |
| 61,50 | 1,50 | 21,77 | 0,6 | 1,2 | 3,35 |
| 54,50 | -0,50 | 22,31 | 0,8 | 1,6 | 4,40 |
| 71,40 | 4,40 | 22,13 | 1,4 | 3,2 | 1,40 |
| 79,60 | 4,60 | 29,30 | 2,0 | 4,2 | 3,23 |

| | | | | | |
|---|---|---|---|---|---|
| 46,30 | 0,30 | 20,44 | 2,7 | 5,6 | 0,10 |
| 61,70 | -0,30 | 20,24 | 2,7 | 6,4 | NAN |
| 56,90 | -1,10 | 20,80 | 0,6 | 1,4 | 1,65 |
| 50,00 | 0,00 | 19,78 | 3,8 | 8,2 | 0,84 |
| 69,40 | 5,90 | 23,61 | 4,6 | 8,4 | 1,74 |
| 59,20 | -0,80 | 24,97 | 1,2 | 2,5 | 0,18 |
| 55,40 | 1,40 | 18,69 | 13,3 | 27,5 | 2,26 |
| 55,00 | 0,00 | 19,72 | 3,4 | 6,6 | NAN |
| 60,00 | 0,00 | 24,03 | 4,8 | 10,1 | NAN |
| 58,10 | -1,90 | 21,77 | 0,7 | 1,4 | 3,34 |
| 74,50 | 2,50 | 26,45 | 0,6 | 1,2 | 2,03 |
| 55,00 | -1,00 | 21,60 | 4,7 | 9,3 | 0,08 |
| 54,70 | 1,70 | 23,56 | 0,8 | 1,8 | NAN |
| 59,40 | 5,40 | 22,19 | 3,7 | 7,7 | 1,61 |
| 48,40 | -1,10 | 18,63 | 1,0 | 1,9 | 0,74 |
| 95,50 | 0,50 | 35,76 | 2,4 | 4,5 | 1,52 |
| 63,30 | 0,70 | 24,76 | 1,6 | 3,3 | 1,13 |
| 66,30 | 4,30 | 22,77 | 0,8 | 1,6 | 2,06 |
| 73,00 | 3,00 | 25,71 | 6,8 | 14,2 | NAN |
| 60,00 | -1,00 | 24,13 | 1,0 | 1,8 | 0,98 |
| 66,70 | 0,70 | 24,24 | 4,3 | 9,3 | NAN |
| 63,50 | 3,50 | 23,44 | 8,5 | 20,0 | 6,48 |
| 59,70 | 2,70 | 20,94 | 2,7 | 6,1 | NAN |
| 50,00 | 1,00 | 20,93 | 10,2 | 20,4 | 1,78 |
| 67,70 | 0,70 | 24,61 | 0,7 | 1,6 | NAN |
| 59,60 | 2,60 | 19,72 | 0,7 | 1,5 | 2,58 |
| 57,00 | 2,00 | 19,03 | 4,0 | 9,2 | NAN |
| 52,00 | 2,00 | 17,72 | 1,1 | 2,6 | NAN |
| 70,50 | -0,50 | 25,46 | 2,0 | 3,9 | 1,15 |
| 48,30 | -10,10 | 25,28 | 3,7 | 7,5 | 2,47 |
| 47,20 | 0,20 | 19,82 | 0,6 | 1,2 | 2,63 |
| NAN | NAN | 20,60 | 1,9 | 4,0 | NAN |
| 64,40 | 1,90 | 21,37 | 1,2 | 2,3 | NAN |
| 52,50 | 0,50 | 20,06 | 1,1 | 2,2 | NAN |
| 65,60 | 2,10 | 24,50 | 7,5 | 14,3 | 1,20 |
| 61,30 | 2,30 | 22,48 | 1,2 | 2,4 | 1,73 |
| 64,60 | 6,60 | 22,38 | 1,0 | 2,0 | 1,47 |
| 67,40 | -7,60 | 28,93 | 1,4 | 3,1 | 2,24 |
| 69,80 | 4,80 | 24,17 | 11,4 | 22,8 | NAN |
| 56,50 | -0,50 | 22,83 | 1,8 | 3,4 | NAN |
| 54,20 | 2,70 | 19,15 | 8,3 | 16,5 | 2,36 |
| 78,00 | 3,00 | 24,21 | 0,8 | 1,7 | 2,84 |
| 57,90 | 2,90 | 19,49 | 0,6 | 1,3 | 0,54 |
| 57,30 | -0,70 | 21,56 | 2,0 | 4,3 | 5,42 |
| 73,50 | 2,50 | 24,86 | 9,9 | 20,0 | 0,79 |
| 53,20 | -0,80 | 21,91 | 11,2 | 26,2 | NAN |
| 58,60 | -4,40 | 26,22 | 1,1 | 2,4 | NAN |
| 52,60 | -0,40 | 19,71 | 0,9 | 1,7 | NAN |
| 51,30 | 1,80 | 19,83 | 3,3 | 8,0 | 2,00 |
| NAN | NAN | 21,88 | 3,9 | 7,3 | 0,93 |

| | | | | | |
|---|---|---|---|---|---|
| 77,70 | -1,30 | 30,86 | 1,5 | 3,0 | 1,38 |
| 62,50 | 4,50 | 21,30 | 6,7 | 15,6 | 0,99 |
| 57,20 | 2,70 | 24,88 | 3,4 | 5,8 | 1,72 |
| 69,80 | 4,80 | 28,13 | 1,9 | 4,0 | 2,71 |
| 51,70 | 1,20 | 23,69 | 5,6 | 10,0 | 1,18 |
| 59,80 | -0,20 | 25,30 | 1,2 | 2,3 | 3,58 |
| 83,20 | 0,20 | 29,76 | 0,5 | 1,2 | 5,83 |
| 83,00 | 2,00 | 28,03 | 1,5 | 3,4 | 1,06 |
| 61,40 | 1,90 | 23,24 | 1,1 | 2,4 | 2,47 |
| 55,50 | 1,50 | 21,63 | 1,1 | 2,5 | 0,81 |
| 57,40 | 0,40 | 22,83 | 1,1 | 2,2 | NAN |
| 59,60 | 3,10 | 21,80 | 2,3 | 4,6 | 1,09 |
| 58,20 | 2,20 | 21,08 | 1,8 | 3,4 | 0,46 |
| 80,50 | 1,00 | 31,45 | 2,8 | 5,4 | 2,08 |
| 48,70 | 0,20 | 18,48 | 11,0 | 21,7 | 2,47 |
| 68,60 | 3,60 | 26,71 | 0,7 | 1,3 | 3,64 |
| 59,50 | 3,50 | 23,61 | 4,1 | 9,3 | 11,50 |
| 56,70 | 2,20 | 20,26 | 0,9 | 2,0 | NAN |
| 58,80 | 3,30 | 19,90 | 3,7 | 6,6 | 1,37 |
| 83,40 | 0,40 | 31,24 | 2,5 | 4,8 | 3,51 |
| 68,40 | -1,60 | 28,40 | 1,4 | 2,9 | 0,63 |
| 51,90 | 3,90 | 19,23 | 7,9 | 16,4 | 0,62 |
| 69,50 | 4,00 | 24,35 | 0,7 | 1,3 | 4,24 |
| 54,30 | -0,20 | 19,08 | 10,2 | 21,8 | NAN |
| 70,40 | 6,40 | 25,32 | 1,9 | 3,4 | 1,91 |
| 67,00 | 5,00 | 25,48 | 1,3 | 2,8 | 0,57 |
| 55,10 | 0,40 | 22,19 | 1,1 | 2,4 | 1,72 |
| 82,10 | 0,60 | 33,49 | 1,5 | 2,8 | 1,39 |
| 78,20 | 3,20 | 25,35 | 0,9 | 1,7 | 2,16 |
| 53,10 | 1,10 | 19,57 | 4,0 | 7,7 | 0,57 |
| 54,00 | 3,00 | 19,43 | 0,6 | 1,2 | 0,14 |
| 59,40 | 2,40 | 19,96 | 1,0 | 1,8 | 3,93 |
| 62,00 | -0,50 | 19,73 | 1,0 | 1,9 | 1,48 |
| 53,10 | -4,90 | 22,66 | 3,4 | 7,3 | 0,79 |
| 61,60 | 2,60 | 22,21 | 6,7 | 13,6 | 1,06 |
| 63,50 | 0,00 | 24,80 | 1,6 | 3,0 | 2,19 |
| 60,40 | 0,90 | 19,43 | 1,2 | 2,3 | 0,36 |
| 47,10 | 0,10 | 20,89 | 6,7 | 13,6 | 2,10 |
| 56,40 | -7,60 | 24,39 | 2,0 | 4,4 | 1,31 |
| 49,70 | 4,70 | 20,00 | 2,2 | 4,3 | 0,08 |
| 56,30 | 2,80 | 20,90 | 0,9 | 2,0 | NAN |
| 56,50 | 1,00 | 24,02 | 1,0 | 1,8 | 0,54 |
| 59,60 | 1,60 | 24,14 | 0,8 | 1,6 | 1,59 |
| 59,20 | 2,20 | 21,45 | 1,7 | 3,2 | 1,40 |
| 68,30 | 5,80 | 22,41 | 0,8 | 1,6 | 0,93 |
| 57,80 | 0,80 | 19,96 | 0,6 | 1,3 | 3,65 |
| 88,50 | 4,50 | 27,43 | 0,9 | 1,7 | 1,80 |
| 62,10 | 2,90 | 22,84 | 4,7 | 9,2 | 1,82 |
| 57,90 | 0,90 | 17,99 | 6,5 | 11,8 | 0,33 |
| 71,60 | 5,60 | 25,78 | 2,4 | 4,6 | 6,63 |

| | | | | | |
|---|---|---|---|---|---|
| 55,00 | 1,00 | 23,07 | 0,8 | 1,8 | 2,36 |
| 57,50 | 5,00 | 21,85 | 3,7 | 7,0 | 2,55 |
| 75,30 | -0,50 | 28,88 | 1,7 | 3,5 | 0,77 |
| 72,00 | 0,00 | 25,82 | 4,8 | 9,8 | 2,27 |
| 60,30 | 3,30 | 23,12 | 2,4 | 4,8 | 2,75 |
| 64,90 | 4,40 | 20,93 | 5,1 | 11,0 | NAN |
| 56,30 | -0,70 | 21,45 | 1,0 | 2,1 | 1,68 |
| NAN | NAN | 19,82 | 7,0 | 14,5 | NAN |
| 61,10 | 3,10 | 20,31 | 2,7 | 5,0 | 2,14 |
| 74,70 | 7,70 | 24,02 | 0,6 | 1,4 | 0,21 |
| 61,60 | -4,40 | 27,47 | 1,4 | 2,8 | 3,46 |
| 56,50 | -2,50 | 24,24 | 0,9 | 1,7 | NAN |
| 55,70 | 2,70 | 19,95 | 0,7 | 1,3 | 1,79 |
| 52,20 | 3,70 | 19,43 | 8,5 | 15,2 | NAN |
| 64,90 | 13,40 | 20,63 | 2,2 | 4,6 | 1,27 |
| 67,80 | 0,80 | 26,84 | 3,0 | 5,5 | NAN |
| 53,90 | 2,90 | 20,43 | 2,1 | 3,8 | 2,13 |
| 61,90 | 4,90 | 20,44 | 4,4 | 9,2 | 3,58 |
| 59,00 | -1,00 | 20,76 | 0,8 | 1,4 | 1,11 |
| 54,20 | -5,80 | 23,44 | 7,1 | 13,0 | NAN |
| 57,30 | 2,30 | 19,72 | 0,6 | 1,3 | NAN |
| 59,40 | 2,40 | 21,72 | 2,0 | 3,9 | 0,89 |
| 90,00 | 3,00 | 32,35 | 0,8 | 1,5 | 2,35 |
| 81,00 | -2,00 | 31,63 | 3,2 | 6,6 | 2,23 |
| 65,00 | 2,50 | 21,13 | 4,4 | 9,7 | NAN |
| 61,70 | 1,70 | 21,26 | 2,6 | 5,4 | 0,99 |
| 77,90 | 1,90 | 32,47 | 2,6 | 5,4 | 0,98 |
| NAN | NAN | 19,74 | 1,2 | 3,0 | NAN |
| 63,20 | 8,20 | 20,70 | 0,8 | 1,5 | 1,01 |
| 59,30 | -0,70 | 25,63 | 1,1 | 2,2 | NAN |
| 54,90 | 1,90 | 20,96 | 9,0 | 16,2 | NAN |
| 76,30 | 2,80 | 24,84 | 1,5 | 2,9 | 1,14 |
| 63,00 | 2,00 | 23,24 | 0,9 | 1,8 | 0,39 |
| 71,20 | 1,70 | 23,49 | 2,9 | 5,6 | 2,09 |
| 63,60 | 3,60 | 22,86 | 5,7 | 10,5 | NAN |
| 66,40 | 4,40 | 22,50 | 1,9 | 3,7 | 2,34 |
| 52,40 | 1,40 | 18,96 | 0,6 | 1,2 | 0,67 |
| 61,90 | 0,90 | 23,24 | 3,7 | 8,2 | 3,57 |
| 82,30 | 5,30 | 27,94 | 6,6 | 12,2 | 0,30 |
| 75,90 | 5,90 | 26,67 | 1,0 | 1,8 | 2,33 |
| 69,60 | 1,60 | 24,09 | 0,9 | 2,1 | 3,32 |
| 69,70 | 2,70 | 23,46 | 10,2 | 18,8 | 2,08 |
| 73,30 | 7,30 | 24,84 | 4,2 | 8,5 | 2,01 |
| 61,10 | 3,10 | 22,94 | 1,0 | 2,3 | NAN |
| 60,00 | 2,50 | 21,12 | 4,5 | 8,1 | 3,16 |
| 57,20 | 4,20 | 22,35 | 0,6 | 1,4 | 3,49 |
| 51,00 | 0,00 | 18,96 | 1,3 | 2,6 | 3,15 |
| 71,20 | 1,20 | 25,10 | 1,1 | 2,3 | 3,20 |
| 55,30 | 2,30 | 17,92 | 2,6 | 5,1 | 3,24 |
| 78,70 | -2,30 | 30,12 | 15,6 | 30,0 | 2,55 |

| | | | | | |
|---|---|---|---|---|---|
| 69,00 | 3,00 | 27,47 | 12,7 | 25,4 | 1,57 |
| 69,20 | 0,20 | 23,32 | 1,8 | 3,3 | 0,63 |
| 73,10 | 3,10 | 28,04 | 7,7 | 13,9 | 2,57 |
| 57,90 | -0,10 | 22,10 | 7,2 | 13,3 | 1,85 |
| 66,10 | 1,10 | 25,39 | 4,6 | 8,8 | 1,51 |
| 64,50 | 0,50 | 25,64 | 1,7 | 3,3 | 1,11 |
| 87,50 | -1,80 | 32,80 | 2,1 | 3,8 | 1,50 |
| 66,20 | 2,20 | 22,95 | 1,2 | 2,4 | 1,15 |
| 64,80 | -0,20 | 25,08 | 1,0 | 2,1 | NAN |
| 76,30 | 4,30 | 27,78 | 8,8 | 16,0 | 0,45 |
| 60,80 | -2,20 | 28,00 | 3,3 | 6,0 | 1,92 |
| 66,20 | -8,80 | 27,55 | 1,2 | 2,4 | 1,91 |
| 52,60 | 1,60 | 21,23 | 1,6 | 3,5 | 3,31 |
| 98,00 | 3,00 | 32,11 | 2,4 | 4,3 | 2,64 |
| 52,60 | 0,60 | 21,10 | 0,8 | 1,8 | NAN |
| 79,60 | -4,40 | 32,81 | 1,1 | 2,0 | 0,95 |

| FT4 (ng/dl) 12 gw | Adiponectin (mcg/mL) 12 gw | IL6 (pg/mL) 12 gw | Leptin (ng/ml) 12 gw | TNF alpha (pg/mL) 12 gw | Systolic BP (mmHg) 12 gw |
|---|---|---|---|---|---|
| 8,83 | 23,40 | 3,04 | 2,85 | 2,16 | NAN |
| 8,68 | 5,15 | 4,99 | 29,18 | 3,65 | NAN |
| 8,59 | 20,37 | 5,19 | 14,34 | 3,42 | NAN |
| NAN | 26,43 | 7,19 | 20,16 | 2,96 | NAN |
| 8,08 | 24,15 | 4,72 | 7,72 | 3,44 | NAN |
| 9,76 | 19,10 | 3,38 | 1,65 | 1,46 | NAN |
| 8,25 | 12,01 | 1,57 | 1,59 | 2,61 | NAN |
| 8,46 | 7,56 | 2,17 | 28,81 | 1,37 | NAN |
| 9,63 | 19,39 | 1,43 | 11,57 | 4,83 | NAN |
| 6,43 | 20,72 | 2,78 | 19,65 | 2,54 | NAN |
| 8,73 | 26,73 | 1,97 | 5,22 | 1,07 | NAN |
| NAN | 33,53 | 1,29 | 22,16 | 1,90 | NAN |
| 9,21 | 14,81 | 4,05 | 2,35 | 0,74 | NAN |
| NAN | 14,82 | 1,63 | 14,86 | 2,72 | NAN |
| 8,20 | 28,41 | 2,71 | 3,60 | 4,16 | NAN |
| 10,77 | 35,05 | 25,51 | 4,83 | 2,50 | NAN |
| 10,27 | 25,95 | 10,12 | 4,53 | 2,92 | NAN |
| 8,43 | 14,11 | 6,62 | 5,19 | 4,32 | NAN |
| 15,90 | 34,94 | 3,31 | 3,76 | 17,96 | NAN |
| 7,17 | 6,42 | 2,52 | 13,66 | 11,92 | NAN |
| 7,31 | 29,08 | 9,52 | 2,92 | 4,74 | NAN |
| 9,72 | 26,43 | 18,39 | 8,08 | 9,06 | NAN |
| 9,45 | 21,89 | 2,81 | 24,19 | 4,86 | NAN |
| 8,50 | 30,73 | 6,31 | 7,29 | 5,20 | NAN |
| 8,97 | 28,09 | 3,71 | 26,54 | 5,34 | NAN |
| 8,44 | 22,17 | 9,42 | 3,39 | 5,46 | NAN |
| 6,19 | 27,35 | 1,51 | 8,07 | 5,99 | NAN |
| NAN | 46,77 | 3,31 | 16,15 | 6,23 | NAN |
| 9,97 | 43,07 | 2,27 | 3,80 | 1,29 | NAN |
| NAN | 27,38 | 9,14 | 6,17 | 15,36 | NAN |
| 7,20 | 9,07 | 18,55 | 14,56 | 9,38 | NAN |
| 9,02 | 20,69 | 6,50 | 6,34 | 11,80 | NAN |
| 7,77 | 26,84 | 0,01 | 3,69 | 4,03 | NAN |
| 8,08 | 18,24 | 15,01 | 8,38 | 3,36 | NAN |
| 9,43 | 16,80 | 2,31 | 19,63 | 4,32 | NAN |
| 9,55 | 28,90 | 2,31 | 4,78 | 0,01 | NAN |
| 10,33 | 35,22 | 8,16 | 1,87 | 2,65 | NAN |
| 9,61 | 11,32 | 5,41 | 25,41 | 1,59 | NAN |
| 6,71 | 7,17 | 3,69 | 19,12 | 4,90 | NAN |
| NAN | 16,21 | 4,10 | 13,09 | 3,58 | NAN |
| NAN | 6,14 | 1,99 | 18,46 | 4,87 | NAN |
| 6,85 | 18,15 | 13,83 | 13,83 | 2,47 | NAN |
| 7,97 | 19,41 | 0,01 | 20,85 | 5,26 | NAN |
| 7,53 | 14,19 | 11,67 | 4,80 | 4,35 | NAN |
| 9,34 | 34,22 | 0,01 | 6,18 | 2,41 | NAN |
| 9,13 | 17,10 | 1,54 | 17,95 | 3,63 | NAN |
| 8,70 | 31,04 | 54,39 | 2,00 | 4,58 | NAN |

| | | | | | |
|---|---|---|---|---|---|
| 8,41 | 30,45 | 2,85 | 5,02 | 2,41 | NAN |
| 8,80 | 31,01 | 2,39 | 9,38 | 3,05 | NAN |
| 9,10 | 20,43 | 17,77 | 14,05 | 3,76 | NAN |
| 10,03 | 27,69 | 3,75 | 27,64 | 4,48 | NAN |
| 12,87 | 13,19 | 2,87 | 31,20 | 2,94 | NAN |
| NAN | 21,60 | 0,87 | 2,84 | 3,23 | NAN |
| 16,84 | 37,60 | 2,39 | 6,05 | 3,70 | NAN |
| NAN | NAN | NAN | NAN | NAN | NAN |
| 7,54 | 20,43 | 10,07 | 5,79 | 1,78 | NAN |
| 6,74 | 26,27 | 1,92 | 5,86 | 2,48 | NAN |
| 9,16 | 21,30 | 1,29 | 7,58 | 2,19 | NAN |
| 8,57 | 38,12 | 1,77 | 4,24 | 2,12 | NAN |
| 7,94 | NAN | 2,08 | 17,91 | 2,71 | NAN |
| 8,54 | 13,18 | 5,90 | 5,46 | 2,90 | NAN |
| 8,02 | 18,28 | 0,87 | 7,69 | 3,43 | NAN |
| 9,42 | NAN | NAN | NAN | NAN | NAN |
| 8,32 | 11,72 | 5,38 | 11,28 | 2,32 | NAN |
| 11,43 | 25,97 | 2,05 | 6,15 | 4,35 | NAN |
| 9,89 | 10,54 | 5,96 | 14,09 | 5,14 | NAN |
| 8,50 | 20,99 | 7,58 | 7,90 | 4,54 | NAN |
| NAN | 11,50 | 8,77 | 11,27 | 2,41 | NAN |
| 10,64 | 37,91 | 0,01 | 6,82 | 4,50 | NAN |
| 8,19 | 11,85 | 1,09 | 13,46 | 2,88 | NAN |
| 8,83 | 27,12 | 7,95 | 2,35 | 4,50 | NAN |
| NAN | 12,11 | 2,56 | 29,14 | 3,97 | NAN |
| 8,30 | 26,47 | 0,01 | 19,64 | 2,61 | 106 |
| 7,88 | 19,76 | 0,01 | 12,04 | 1,03 | NAN |
| 8,59 | 31,43 | 12,18 | 20,67 | 4,08 | NAN |
| 8,33 | 18,13 | 2,86 | 2,87 | 2,24 | 99 |
| 8,77 | 33,73 | 0,01 | 13,41 | 2,48 | 93 |
| 7,13 | 25,50 | 0,01 | 2,38 | 2,85 | 100 |
| 7,09 | 26,46 | 0,01 | 5,93 | 3,22 | NAN |
| NAN | 21,59 | 2,61 | 11,01 | 2,67 | NAN |
| 8,09 | 22,83 | 13,47 | 9,90 | 2,01 | NAN |
| 7,21 | 33,57 | 1,86 | 8,87 | 4,63 | NAN |
| 8,39 | 19,37 | 14,13 | 3,69 | 2,95 | 94 |
| 7,23 | 33,89 | 2,21 | 4,92 | 1,78 | 93 |
| NAN | 5,42 | 0,01 | 6,81 | 2,87 | 108 |
| 8,76 | 9,62 | 0,01 | 6,12 | 3,27 | 106 |
| NAN | 14,10 | 0,95 | 2,73 | 1,71 | NAN |
| NAN | 28,84 | 6,03 | 4,76 | 0,71 | 101 |
| 9,99 | 18,30 | 3,58 | 6,91 | 2,42 | 102 |
| NAN | 10,67 | 0,01 | 13,65 | 4,61 | 120 |
| 9,79 | 31,23 | 1,23 | 1,97 | 2,12 | 96 |
| 9,33 | 29,16 | 3,33 | 2,47 | 0,93 | 115 |
| 7,15 | 10,28 | NAN | NAN | NAN | 100 |
| 7,21 | 19,48 | 6,90 | 8,48 | 0,73 | 102 |
| NAN | 27,79 | 0,84 | 5,82 | 1,67 | 104 |
| 8,91 | 13,23 | 12,61 | 9,49 | 2,89 | 107 |
| 8,07 | 19,70 | 0,01 | 10,38 | 2,20 | 105 |

| | | | | | |
|---|---|---|---|---|---|
| 8,14 | 23,22 | 7,85 | 3,97 | 2,93 | 101 |
| 7,33 | 20,76 | 2,33 | 2,90 | 1,14 | 97 |
| NAN | 22,80 | 48,00 | 1,58 | 2,21 | 104 |
| 8,52 | 29,15 | 3,32 | 4,78 | 1,83 | 104 |
| 7,63 | 17,88 | 2,69 | 8,75 | 2,08 | 111 |
| NAN | 19,63 | 7,29 | 10,78 | 0,77 | 102 |
| 8,05 | 21,58 | 11,75 | 6,47 | 2,09 | 101 |
| 8,74 | 14,97 | 0,01 | 6,13 | 2,21 | 110 |
| 6,18 | 16,48 | 2,33 | 7,30 | 3,29 | 104 |
| 8,88 | NAN | NAN | NAN | NAN | 111 |
| 7,58 | 25,21 | 1,59 | 6,35 | 1,74 | 104 |
| NAN | 7,13 | 1,03 | 2,40 | 1,45 | 117 |
| 8,22 | 18,73 | 20,64 | 1,31 | 0,77 | 103 |
| 11,65 | 33,18 | 8,90 | 3,24 | 2,57 | NAN |
| 7,94 | 11,54 | 15,44 | 12,59 | 2,34 | NAN |
| 7,96 | 26,34 | 1,67 | 3,65 | 2,51 | 84 |
| 7,51 | NAN | NAN | NAN | NAN | 118 |
| 7,97 | 28,95 | 2,46 | 5,44 | 16,61 | 83 |
| 7,21 | 11,30 | 8,38 | 10,79 | 4,36 | 106 |
| 7,45 | 16,64 | 0,84 | 0,45 | 1,87 | 95 |
| 8,86 | 21,40 | 53,97 | 7,55 | 32,73 | 107 |
| 9,85 | 17,13 | 3,48 | 11,93 | 6,90 | 107 |
| 8,50 | 29,85 | 3,48 | 6,42 | 3,11 | 97 |
| 6,80 | 46,29 | 3,87 | 15,20 | 4,02 | 90 |
| 7,82 | 9,53 | 28,60 | 9,27 | 5,31 | 117 |
| 9,74 | 18,75 | 24,43 | 5,91 | 8,91 | 91 |
| 8,92 | 16,41 | 6,19 | 1,45 | 1,94 | 115 |
| NAN | 21,68 | 0,96 | 15,51 | 2,12 | 142 |
| 8,52 | 14,62 | 13,66 | 13,60 | 4,28 | 95 |
| 7,67 | 22,28 | 4,44 | 2,72 | 5,77 | 104 |
| 8,65 | 17,84 | 18,01 | 25,26 | 6,82 | 127 |
| 7,41 | 42,40 | 23,25 | 7,21 | 8,50 | 113 |
| NAN | 14,81 | 5,34 | 6,37 | 4,43 | 126 |
| 9,04 | 14,41 | 1,09 | 4,96 | 2,73 | 100 |
| 7,25 | 11,06 | 3,62 | 13,14 | 5,66 | 113 |
| 8,27 | 12,90 | 0,01 | 4,27 | 1,78 | 100 |
| 11,07 | 10,20 | 7,73 | 20,87 | 5,49 | 105 |
| 8,29 | 7,11 | 0,01 | 8,74 | 3,81 | 112 |
| 9,19 | 14,64 | 3,63 | 12,14 | 1,83 | 99 |
| 6,76 | 15,85 | 8,96 | 11,36 | 5,55 | 124 |
| 9,04 | 20,21 | 0,97 | 9,91 | 4,78 | 100 |
| 6,61 | 8,67 | 4,39 | 12,67 | 1,79 | 133 |
| 10,16 | 7,43 | 33,36 | 10,18 | 3,36 | 120 |
| 8,95 | 15,24 | 8,02 | 6,35 | 1,61 | 105 |
| 7,72 | 10,34 | 0,01 | 4,57 | 1,74 | 100 |
| 9,47 | 20,72 | 6,76 | 7,06 | 3,33 | 105 |
| 8,71 | 24,39 | 0,01 | 5,47 | 2,62 | 110 |
| 7,89 | 11,65 | 33,90 | 1,67 | 1,11 | 92 |
| 8,17 | 15,90 | 4,26 | 6,32 | 2,18 | 120 |
| 6,47 | 11,37 | 0,98 | 7,98 | 4,16 | 113 |

| | | | | | |
|---|---|---|---|---|---|
| 12,66 | 15,32 | 2,30 | 6,95 | 1,83 | 103 |
| NAN | 14,10 | 11,88 | 10,05 | 4,70 | 106 |
| 8,13 | 12,41 | 0,01 | 2,86 | 1,61 | 108 |
| 9,16 | 23,49 | 1,47 | 2,17 | 1,23 | 103 |
| 10,00 | 27,68 | 8,46 | 7,80 | 5,57 | 101 |
| 12,92 | 14,20 | 3,26 | 4,47 | 7,18 | 99 |
| 8,52 | 27,94 | 0,01 | 2,60 | 3,64 | 115 |
| NAN | 8,48 | 0,01 | 1,02 | 1,60 | 102 |
| NAN | 20,77 | 0,01 | 7,77 | 2,05 | 106 |
| 9,06 | 25,59 | 0,01 | 1,24 | 0,96 | 113 |
| 9,68 | 18,02 | 0,01 | 2,67 | 0,62 | 109 |
| 12,01 | 20,79 | 5,01 | 2,06 | 1,38 | 91 |
| NAN | 43,93 | 0,01 | 1,87 | 1,11 | 107 |
| 8,18 | 13,44 | 2,23 | 4,76 | 1,88 | 109 |
| 11,67 | 22,40 | 0,01 | 1,70 | 0,58 | 111 |
| 6,14 | 14,23 | 1,57 | 4,32 | 0,54 | 123 |
| 7,69 | 18,78 | 0,01 | 3,58 | 2,55 | 129 |
| 8,53 | 16,41 | 0,01 | 7,63 | 1,17 | 107 |
| NAN | 12,56 | 8,38 | 15,51 | 4,32 | 108 |
| 10,63 | 31,57 | 2,04 | 6,41 | 3,14 | 93 |
| NAN | 16,95 | 0,81 | 4,93 | 2,22 | 102 |
| 8,88 | 24,19 | 0,83 | 4,04 | 2,43 | 110 |
| NAN | 14,22 | 1,59 | 7,39 | 1,88 | 96 |
| 8,46 | 17,73 | 1,19 | 6,33 | 1,80 | 89 |
| NAN | 17,77 | 1,44 | 16,40 | 3,26 | 90 |
| 7,14 | 9,67 | 0,81 | 5,18 | 1,54 | 110 |
| NAN | 31,40 | 0,01 | 2,58 | 1,59 | 110 |
| NAN | 18,65 | 1,88 | 1,11 | 3,22 | 118 |
| 8,09 | 12,85 | 0,01 | 12,75 | 1,20 | 91 |
| 6,94 | 16,00 | 1,03 | 7,24 | 3,30 | 104 |
| 8,45 | 41,14 | 2,49 | 5,56 | 2,62 | 97 |
| NAN | 17,90 | 0,01 | 5,52 | 4,02 | 85 |
| NAN | 22,58 | 75,01 | 8,27 | 3,49 | 119 |
| NAN | 12,54 | 5,10 | 5,59 | 0,01 | 91 |
| 8,64 | 15,80 | 16,41 | 15,90 | 5,60 | 111 |
| 8,19 | 24,87 | 6,67 | 8,76 | 3,18 | 91 |
| 7,76 | 18,94 | 0,01 | 6,40 | 1,60 | 90 |
| 9,59 | 11,45 | 1,95 | 5,73 | 0,01 | 104 |
| NAN | 12,84 | 3,41 | 25,09 | 2,38 | 116 |
| NAN | 15,50 | 4,64 | 15,27 | 6,43 | 116 |
| 9,94 | 24,94 | 0,01 | 5,33 | 1,60 | 90 |
| 8,06 | 6,87 | 4,64 | 17,13 | 2,35 | 114 |
| 10,45 | 21,25 | NAN | NAN | NAN | 87 |
| 9,66 | 16,85 | NAN | NAN | NAN | 113 |
| 9,14 | 11,59 | NAN | NAN | NAN | 129 |
| NAN | 12,04 | NAN | NAN | NAN | 103 |
| NAN | 16,65 | NAN | NAN | NAN | 100 |
| NAN | 11,77 | NAN | NAN | NAN | 101 |
| 9,61 | 22,37 | NAN | NAN | NAN | 94 |
| 12,15 | 18,86 | NAN | NAN | NAN | 101 |

| | | | | | |
|---|---|---|---|---|---|
| 6,98 | 13,74 | NAN | NAN | NAN | 124 |
| 8,11 | 11,26 | NAN | NAN | NAN | 122 |
| 8,11 | 8,64 | NAN | NAN | NAN | 107 |
| 9,41 | 14,58 | NAN | NAN | NAN | 105 |
| 7,26 | 15,98 | NAN | NAN | NAN | 109 |
| 7,93 | 20,78 | 0,01 | 8,50 | 2,43 | 110 |
| 10,19 | 6,77 | NAN | NAN | NAN | 107 |
| 9,26 | 14,69 | 0,01 | 10,01 | 1,29 | 114 |
| 9,84 | 13,15 | 0,01 | 14,82 | 3,79 | 134 |
| 9,57 | 16,01 | NAN | NAN | NAN | 106 |
| NAN | 16,28 | 26,79 | 3,55 | 1,45 | 99 |
| 10,79 | 22,83 | NAN | NAN | NAN | 112 |
| 10,48 | 21,04 | NAN | NAN | NAN | 107 |
| 9,52 | 15,06 | 0,01 | 17,82 | 1,85 | 91 |
| 7,70 | 20,53 | NAN | NAN | NAN | 110 |
| 7,79 | 42,34 | 45,01 | 10,84 | 8,44 | 110 |
| 6,87 | 21,13 | 29,72 | 8,85 | 1,72 | 103 |
| NAN | 35,04 | NAN | NAN | NAN | 101 |
| 8,95 | 27,00 | NAN | NAN | NAN | 98 |
| 6,30 | 33,50 | NAN | NAN | NAN | NAN |
| 8,11 | 25,76 | NAN | NAN | NAN | 92 |
| 7,80 | 15,17 | 2,30 | 5,10 | 2,52 | 94 |
| 8,76 | 12,40 | 0,01 | 7,30 | 1,92 | 108 |
| NAN | 30,35 | 89,77 | 1,04 | 2,89 | 109 |
| 8,42 | 15,86 | 0,01 | 14,04 | 1,99 | 95 |
| 7,60 | 12,94 | 0,01 | 11,41 | 1,13 | 104 |
| 8,99 | 12,45 | 5,47 | 7,04 | 4,03 | 115 |
| 8,08 | 7,79 | 0,87 | 22,89 | 3,80 | NAN |
| 8,83 | 15,09 | 2,30 | 9,95 | 2,43 | 113 |
| 8,65 | 21,30 | 1,49 | 5,48 | 4,00 | 92 |
| 9,05 | 29,11 | 1,38 | 3,07 | 6,21 | 98 |
| 8,87 | 18,93 | 38,94 | 5,62 | 3,19 | 95 |
| 9,39 | 10,62 | 0,78 | 5,15 | 2,99 | 105 |
| 9,89 | 30,98 | 0,01 | 1,31 | 1,94 | 98 |
| 12,09 | 35,03 | 0,01 | 7,89 | 2,94 | 94 |
| 8,68 | 11,26 | 25,89 | 7,46 | 2,29 | 124 |
| 8,50 | 30,98 | 0,01 | 2,42 | 2,20 | 112 |
| 10,57 | NAN | 0,01 | 3,90 | 1,99 | 94 |
| 7,77 | NAN | 20,76 | 1,52 | 2,09 | 89 |
| 10,99 | NAN | 2,45 | 10,80 | 4,06 | 109 |
| NAN | NAN | 0,01 | 8,34 | 3,33 | 116 |
| 9,81 | NAN | 0,80 | 5,29 | 3,09 | 110 |
| 7,09 | NAN | NAN | NAN | NAN | 100 |
| 9,86 | NAN | 0,01 | 10,41 | 1,57 | 102 |
| 7,86 | NAN | 0,84 | 6,48 | 2,27 | 102 |
| 9,58 | NAN | 0,62 | 1,02 | 2,30 | 86 |
| 8,97 | NAN | 0,01 | 9,04 | 1,18 | 113 |
| 9,23 | NAN | 10,96 | 7,48 | 3,31 | 112 |
| 9,25 | NAN | 0,01 | 2,04 | 2,50 | 96 |
| 7,63 | NAN | NAN | NAN | NAN | 105 |

| | | | | | |
|---|---|---|---|---|---|
| 8,04 | NAN | 0,01 | 2,18 | 2,00 | 94 |
| 8,60 | NAN | 7,71 | 3,97 | 2,21 | 110 |
| 9,20 | NAN | 4,87 | 9,42 | 2,08 | 112 |
| 9,49 | NAN | 0,01 | 1,46 | 1,84 | 124 |
| 8,07 | NAN | NAN | NAN | NAN | 123 |
| NAN | NAN | 6,03 | 3,19 | 1,40 | 108 |
| 9,38 | NAN | 51,62 | 2,79 | 2,24 | 105 |
| NAN | NAN | 0,01 | 0,66 | 1,18 | 100 |
| 10,60 | NAN | 0,01 | 2,85 | 2,73 | 108 |
| 9,46 | NAN | 0,01 | 9,03 | 0,22 | 119 |
| 9,55 | NAN | NAN | NAN | NAN | 102 |
| NAN | NAN | 0,01 | 3,74 | 0,22 | 90 |
| 7,93 | NAN | 4,68 | 1,21 | 0,01 | 104 |
| NAN | NAN | 4,58 | 1,87 | 0,22 | 107 |
| 5,99 | NAN | 0,01 | 2,73 | 1,08 | 114 |
| NAN | NAN | NAN | NAN | NAN | 106 |
| 7,83 | NAN | NAN | NAN | NAN | 101 |
| 6,30 | NAN | NAN | NAN | NAN | 101 |
| 8,23 | NAN | NAN | NAN | NAN | 102 |
| NAN | NAN | NAN | NAN | NAN | 102 |
| NAN | NAN | NAN | NAN | NAN | 99 |
| 8,82 | NAN | NAN | NAN | NAN | 106 |
| 7,22 | NAN | NAN | NAN | NAN | 107 |
| 6,89 | NAN | 40,80 | 12,67 | 1,83 | 117 |
| NAN | NAN | 0,01 | 5,24 | 3,37 | 117 |
| 10,39 | NAN | 32,74 | 11,56 | 4,56 | 103 |
| 7,88 | NAN | 0,01 | 24,28 | 7,50 | 128 |
| NAN | NAN | NAN | NAN | NAN | NAN |
| 9,30 | NAN | NAN | NAN | NAN | 99 |
| NAN | NAN | 5,70 | 11,35 | 3,11 | 117 |
| NAN | NAN | 0,01 | 3,38 | 2,25 | 112 |
| 11,46 | NAN | 0,01 | 18,02 | 3,84 | 112 |
| 8,04 | NAN | NAN | NAN | NAN | 94 |
| 7,54 | NAN | NAN | NAN | NAN | 115 |
| NAN | NAN | 0,01 | 10,13 | 2,18 | 117 |
| 7,60 | NAN | 0,01 | 9,67 | 1,68 | 102 |
| 10,25 | NAN | 0,82 | 7,67 | 5,23 | 112 |
| 9,00 | NAN | 2,58 | 1,83 | 2,52 | 103 |
| 14,21 | NAN | 2,10 | 16,75 | 2,28 | 116 |
| 7,80 | NAN | 0,01 | 16,14 | 1,72 | 95 |
| 7,26 | NAN | NAN | NAN | NAN | 112 |
| 8,53 | NAN | 3,74 | 12,61 | 1,69 | 119 |
| 8,12 | NAN | 0,01 | 13,92 | 1,94 | 104 |
| NAN | NAN | 0,01 | 7,95 | 1,36 | 107 |
| 9,20 | NAN | 0,01 | 12,10 | 1,99 | 104 |
| 6,84 | NAN | 0,82 | 2,98 | 2,50 | 110 |
| 9,33 | NAN | NAN | NAN | NAN | NAN |
| 9,29 | NAN | NAN | NAN | NAN | 108 |
| 9,08 | NAN | NAN | NAN | NAN | 100 |
| 8,13 | NAN | NAN | NAN | NAN | 108 |

| | | | | | |
|---|---|---|---|---|---|
| 8,45 | NAN | 3,90 | 11,12 | 2,07 | 123 |
| 9,97 | NAN | 0,01 | 9,73 | 3,25 | 126 |
| 7,74 | NAN | 8,62 | 12,97 | 1,11 | 110 |
| 8,90 | NAN | 0,01 | 2,09 | 1,77 | 91 |
| 7,78 | NAN | 0,01 | 6,16 | 2,03 | 127 |
| 9,04 | NAN | 1,35 | 16,31 | 2,14 | 101 |
| 7,23 | NAN | 0,01 | 24,12 | 2,23 | 114 |
| 6,98 | NAN | 0,01 | 6,46 | 1,37 | 103 |
| NAN | NAN | 0,01 | 5,01 | 2,47 | 110 |
| 9,42 | NAN | 4,85 | 14,29 | 3,08 | 143 |
| 6,91 | NAN | 3,17 | 13,24 | 2,12 | 114 |
| 7,92 | NAN | 0,01 | 12,57 | 3,38 | 114 |
| 7,81 | NAN | NAN | NAN | NAN | 99 |
| 8,01 | NAN | NAN | NAN | NAN | 113 |
| NAN | NAN | NAN | NAN | NAN | 100 |
| 8,70 | NAN | NAN | NAN | NAN | 110 |

| Diastolic BP (mm Hg) 12 gw | SMOKERs | Fasting Blood Glucose (mg/dl) 12 gw | Fasting Cholesterol (mg/dl) 12 gw | Fasting Tryglycerides (mg/dl) 12 gw | HbA1c (%) 12 gw |
|---|---|---|---|---|---|
| NAN | 1 | 77 | 170 | 61 | NAN |
| NAN | 1 | 82 | 191 | 57 | NAN |
| NAN | 2 | 82 | 217 | 95 | NAN |
| NAN | 2 | 89 | NAN | NAN | NAN |
| NAN | 1 | 76 | 161 | 43 | NAN |
| NAN | 0 | 86 | 113 | 74 | NAN |
| NAN | 1 | 90 | 196 | 75 | NAN |
| NAN | 1 | 85 | 190 | 84 | NAN |
| NAN | 0 | 89 | 173 | 101 | NAN |
| NAN | 0 | 86 | 157 | 95 | NAN |
| NAN | 0 | 84 | 229 | 94 | NAN |
| NAN | 1 | 77 | NAN | NAN | NAN |
| NAN | 2 | 90 | 167 | 54 | NAN |
| NAN | 0 | 87 | NAN | NAN | NAN |
| NAN | 3 | 81 | 124 | 51 | NAN |
| NAN | 0 | 89 | 183 | 82 | NAN |
| NAN | 0 | 81 | 161 | 107 | NAN |
| NAN | 1 | 84 | 113 | 50 | NAN |
| NAN | 0 | 82 | 142 | 60 | NAN |
| NAN | 2 | 87 | 122 | 137 | NAN |
| NAN | 1 | 70 | 185 | 66 | NAN |
| NAN | 0 | 86 | NAN | NAN | NAN |
| NAN | 0 | 88 | 176 | 63 | NAN |
| NAN | 1 | 86 | 198 | 74 | NAN |
| NAN | 2 | 84 | 183 | 99 | NAN |
| NAN | 0 | 85 | 153 | 104 | NAN |
| NAN | 0 | 85 | 244 | 104 | NAN |
| NAN | 1 | 74 | NAN | NAN | NAN |
| NAN | 1 | 87 | 202 | 44 | NAN |
| NAN | 2 | 69 | NAN | NAN | NAN |
| NAN | 2 | 92 | 213 | 171 | NAN |
| NAN | 0 | 85 | 163 | 54 | NAN |
| NAN | 0 | 72 | 161 | 49 | NAN |
| NAN | 0 | 78 | 137 | 96 | NAN |
| NAN | 1 | 88 | 154 | 100 | 5,0 |
| NAN | 0 | 82 | 174 | 95 | NAN |
| NAN | 0 | 81 | 184 | 56 | NAN |
| NAN | 0 | 75 | 155 | 114 | NAN |
| NAN | 0 | 76 | 199 | 243 | NAN |
| NAN | 1 | 67 | NAN | NAN | NAN |
| NAN | 1 | 72 | NAN | NAN | NAN |
| NAN | 0 | 91 | 234 | 112 | NAN |
| NAN | 0 | 88 | 141 | 66 | NAN |
| NAN | 2 | 75 | 216 | 111 | NAN |
| NAN | 1 | 86 | 208 | 45 | NAN |
| NAN | 0 | 80 | 201 | 69 | NAN |
| NAN | 0 | 84 | 227 | 68 | NAN |

| | | | | | |
|---|---|---|---|---|---|
| NAN | 0 | 77 | 173 | 50 | NAN |
| NAN | 1 | 76 | 205 | 70 | NAN |
| NAN | 0 | 89 | 150 | 142 | NAN |
| NAN | 1 | 73 | NAN | NAN | NAN |
| NAN | 9 | 78 | NAN | NAN | NAN |
| NAN | 0 | 77 | NAN | NAN | NAN |
| NAN | 0 | 82 | 134 | 86 | NAN |
| NAN | 0 | 74 | NAN | NAN | NAN |
| NAN | 0 | 83 | 150 | 41 | NAN |
| NAN | 0 | 69 | 222 | 97 | NAN |
| NAN | 0 | 82 | 174 | 97 | NAN |
| NAN | 0 | 80 | 182 | 67 | NAN |
| NAN | 0 | 82 | 123 | 69 | NAN |
| NAN | 3 | 81 | 188 | 68 | NAN |
| NAN | 0 | 83 | 169 | 90 | NAN |
| NAN | 0 | 78 | 219 | 94 | 5,1 |
| NAN | 0 | 85 | 295 | 156 | NAN |
| NAN | 0 | 78 | 195 | 56 | NAN |
| NAN | 0 | 69 | NAN | NAN | NAN |
| NAN | 1 | 90 | 214 | 80 | NAN |
| NAN | 1 | 73 | NAN | NAN | NAN |
| NAN | 0 | 82 | 163 | 62 | NAN |
| NAN | 0 | 79 | 176 | 169 | NAN |
| NAN | 3 | 84 | 182 | 70 | NAN |
| NAN | 0 | 86 | NAN | NAN | NAN |
| 54 | 0 | 75 | 128 | 65 | NAN |
| NAN | 2 | 88 | 180 | 70 | NAN |
| NAN | 3 | 86 | 158 | 83 | NAN |
| 57 | 1 | 68 | 159 | 54 | NAN |
| 60 | 1 | 88 | 159 | 48 | NAN |
| 65 | 0 | 72 | 136 | 61 | NAN |
| NAN | 1 | 84 | 149 | 88 | NAN |
| NAN | 1 | 74 | NAN | NAN | NAN |
| NAN | 2 | 68 | 186 | 74 | NAN |
| NAN | 0 | 65 | 172 | 73 | NAN |
| 69 | 3 | 76 | 169 | 75 | NAN |
| 60 | 0 | 67 | 165 | 86 | NAN |
| 73 | 0 | 77 | NAN | NAN | NAN |
| 51 | 0 | 75 | 186 | 161 | NAN |
| NAN | 0 | 69 | NAN | NAN | NAN |
| 64 | 1 | 88 | NAN | NAN | NAN |
| 39 | 0 | 87 | 207 | 117 | NAN |
| 78 | 0 | 69 | NAN | NAN | NAN |
| 57 | 1 | 83 | 169 | 77 | NAN |
| 71 | 1 | 87 | NAN | NAN | NAN |
| 58 | 2 | 86 | 214 | 157 | NAN |
| 58 | 2 | 72 | 162 | 37 | NAN |
| 69 | 0 | 88 | NAN | NAN | NAN |
| 67 | 0 | 81 | 168 | 81 | NAN |
| 53 | 0 | 77 | NAN | NAN | NAN |

| | | | | | |
|---|---|---|---|---|---|
| 61 | 0 | 85 | 173 | 56 | NAN |
| 58 | 2 | 74 | 207 | 87 | NAN |
| 57 | 0 | 84 | NAN | NAN | NAN |
| 67 | 0 | 91 | 120 | 59 | NAN |
| 68 | 1 | 85 | 140 | 102 | NAN |
| 58 | 0 | 83 | NAN | NAN | NAN |
| 63 | 2 | 81 | 167 | 41 | NAN |
| 59 | 0 | 81 | 217 | 115 | NAN |
| 55 | 0 | 88 | 187 | 160 | NAN |
| 62 | 0 | 87 | 168 | 70 | NAN |
| 63 | 1 | 78 | 190 | 69 | NAN |
| 60 | 0 | 77 | NAN | NAN | NAN |
| 67 | 0 | 82 | 178 | 80 | NAN |
| NAN | 1 | 91 | 150 | 50 | NAN |
| NAN | 1 | 85 | 161 | 68 | NAN |
| 56 | 0 | 91 | 173 | 88 | NAN |
| 65 | 1 | 76 | 190 | 85 | NAN |
| 58 | 2 | 83 | 180 | 66 | NAN |
| 75 | 3 | 68 | 170 | 72 | NAN |
| 59 | 0 | 75 | 132 | 50 | NAN |
| 71 | 3 | 74 | 184 | 91 | NAN |
| 68 | 1 | 86 | 182 | 43 | NAN |
| 54 | 3 | 90 | 187 | 61 | NAN |
| 55 | 0 | 90 | 184 | 111 | NAN |
| 68 | 0 | 91 | 209 | 204 | NAN |
| 51 | 3 | 75 | 147 | 44 | NAN |
| 57 | 1 | 73 | NAN | NAN | NAN |
| 92 | 0 | 78 | 179 | 98 | NAN |
| 57 | 0 | 73 | 113 | 72 | NAN |
| 68 | 2 | 85 | 195 | 66 | 5,0 |
| 76 | 2 | 85 | 198 | 132 | NAN |
| 66 | 0 | 83 | 221 | 136 | NAN |
| 76 | 1 | 75 | NAN | NAN | NAN |
| 65 | 1 | 78 | 169 | 43 | NAN |
| 80 | 0 | 82 | 196 | 164 | NAN |
| 60 | 3 | 85 | 170 | 100 | NAN |
| 60 | 0 | 77 | 189 | 97 | NAN |
| 73 | 0 | 89 | 157 | 82 | NAN |
| 63 | 1 | 82 | 182 | 73 | NAN |
| 85 | 1 | 79 | 111 | 63 | NAN |
| 63 | 1 | 79 | 159 | 77 | NAN |
| 68 | 0 | 78 | 234 | 201 | NAN |
| 71 | 0 | 85 | 118 | 39 | NAN |
| 67 | 0 | 82 | 128 | 70 | NAN |
| 63 | 3 | 75 | 190 | 80 | NAN |
| 61 | 0 | 87 | 212 | 56 | NAN |
| 73 | 0 | 79 | 155 | 47 | NAN |
| 51 | 0 | 84 | 206 | 70 | NAN |
| 68 | 1 | 70 | 159 | 29 | NAN |
| 68 | 0 | 79 | 245 | 198 | NAN |

| | | | | | |
|---|---|---|---|---|---|
| 66 | 0 | 78 | 193 | 110 | NAN |
| 66 | 1 | 69 | NAN | NAN | NAN |
| 75 | 0 | 72 | 195 | 89 | NAN |
| 69 | 0 | 74 | 171 | 59 | NAN |
| 68 | 1 | 89 | 174 | 48 | NAN |
| 66 | 1 | 78 | 210 | 123 | NAN |
| 74 | 1 | 78 | 190 | 83 | NAN |
| 64 | 0 | 82 | NAN | NAN | NAN |
| 77 | 0 | 77 | NAN | NAN | NAN |
| 56 | 1 | 78 | 180 | 45 | NAN |
| 77 | 1 | 76 | 96 | 63 | NAN |
| 66 | 0 | 82 | 172 | 122 | NAN |
| 61 | 0 | 72 | NAN | NAN | NAN |
| 60 | 2 | 77 | 192 | 44 | NAN |
| 76 | 2 | 83 | 221 | 89 | NAN |
| 69 | 3 | 85 | 166 | 72 | NAN |
| 85 | 0 | 79 | 187 | 148 | NAN |
| 75 | 1 | 83 | 164 | 76 | NAN |
| 66 | 2 | 77 | NAN | NAN | NAN |
| 68 | 0 | 90 | 206 | 148 | NAN |
| 68 | 1 | 75 | NAN | NAN | NAN |
| 72 | 0 | 69 | NAN | NAN | NAN |
| 64 | 0 | 71 | NAN | NAN | NAN |
| 57 | 1 | 81 | 232 | 125 | NAN |
| 57 | 9 | 73 | NAN | NAN | NAN |
| 73 | 1 | 71 | 206 | 83 | NAN |
| 68 | 1 | 70 | NAN | NAN | NAN |
| 83 | 0 | 70 | NAN | NAN | NAN |
| 57 | 0 | 85 | 182 | 92 | NAN |
| 53 | 0 | 79 | 194 | 86 | NAN |
| 59 | 0 | 77 | 132 | 82 | NAN |
| 51 | 2 | 76 | NAN | NAN | NAN |
| 72 | 0 | 83 | NAN | NAN | NAN |
| 62 | 3 | 83 | NAN | NAN | NAN |
| 63 | 0 | 85 | 208 | 94 | NAN |
| 69 | 1 | 83 | NAN | NAN | NAN |
| 62 | 0 | 78 | 159 | 79 | NAN |
| 63 | 0 | 76 | 129 | 82 | NAN |
| 67 | 2 | 81 | NAN | NAN | NAN |
| 70 | 0 | 85 | 203 | 139 | NAN |
| 56 | 0 | 81 | 162 | 49 | NAN |
| 66 | 2 | 80 | 137 | 55 | NAN |
| 59 | 0 | 78 | 139 | 40 | NAN |
| 73 | 2 | 74 | 190 | 49 | NAN |
| 67 | 1 | 80 | 123 | 47 | NAN |
| 63 | 0 | 69 | NAN | NAN | NAN |
| 69 | 0 | 71 | NAN | NAN | NAN |
| 69 | 0 | 86 | NAN | NAN | NAN |
| 61 | 0 | 68 | 179 | 53 | NAN |
| 62 | 0 | 87 | 197 | 53 | NAN |

| | | | | | |
|---|---|---|---|---|---|
| 75 | 0 | 80 | 179 | 100 | NAN |
| 76 | 0 | 70 | 161 | 134 | NAN |
| 67 | 0 | 93 | 201 | 147 | NAN |
| 65 | 0 | 75 | 206 | 131 | NAN |
| 67 | 1 | 91 | 170 | 84 | NAN |
| 71 | 0 | 83 | 163 | 86 | NAN |
| 58 | 3 | 72 | 169 | 81 | NAN |
| 78 | 0 | 74 | 170 | 78 | NAN |
| 74 | 0 | 76 | 171 | 71 | NAN |
| 78 | 2 | 75 | NAN | NAN | NAN |
| 64 | 0 | 79 | NAN | NAN | NAN |
| 63 | 0 | 79 | 185 | 42 | NAN |
| 73 | 0 | 87 | 162 | 71 | NAN |
| 57 | 0 | 86 | 163 | 101 | NAN |
| 76 | 2 | 82 | 223 | 136 | 5,2 |
| 62 | 0 | 89 | 194 | 61 | NAN |
| 58 | 0 | 72 | 218 | 152 | 5,3 |
| 68 | 0 | 75 | NAN | NAN | NAN |
| 61 | 2 | 91 | 150 | 55 | NAN |
| NAN | 2 | 84 | 185 | 143 | NAN |
| 50 | 9 | 77 | 203 | 91 | NAN |
| 67 | 2 | 78 | 157 | 101 | NAN |
| 70 | 2 | 89 | 198 | 86 | NAN |
| 64 | 0 | 76 | 185 | 62 | NAN |
| 62 | 2 | 89 | 119 | 67 | NAN |
| 87 | 0 | 77 | 140 | 113 | NAN |
| 67 | 0 | 75 | 178 | 63 | NAN |
| NAN | 0 | 85 | 182 | 88 | NAN |
| 74 | 0 | 87 | 174 | 46 | NAN |
| 56 | 3 | 83 | 164 | 76 | 5,2 |
| 57 | 1 | 87 | 162 | 101 | NAN |
| 53 | 0 | 86 | 144 | 59 | NAN |
| 79 | 0 | 87 | 148 | 65 | NAN |
| 65 | 0 | 76 | 193 | 133 | NAN |
| 57 | 1 | 80 | 163 | 56 | NAN |
| 75 | 9 | 88 | NAN | NAN | NAN |
| 73 | 2 | 86 | 163 | 64 | NAN |
| 54 | 0 | 80 | 131 | 67 | NAN |
| 53 | 0 | 74 | 150 | 117 | NAN |
| 67 | 0 | 82 | NAN | NAN | NAN |
| 83 | 1 | 74 | NAN | NAN | NAN |
| 47 | 0 | 91 | 153 | 183 | NAN |
| 50 | 0 | 78 | 194 | 91 | NAN |
| 55 | 0 | 88 | 171 | 108 | NAN |
| 54 | 2 | 80 | 155 | 72 | 5,5 |
| 50 | 0 | 75 | 155 | 48 | NAN |
| 60 | 0 | 85 | 154 | 52 | NAN |
| 66 | 3 | 83 | 215 | 228 | NAN |
| 68 | 2 | 89 | NAN | NAN | NAN |
| 67 | 1 | 87 | 166 | 103 | NAN |

| | | | | | |
|---|---|---|---|---|---|
| 63 | 0 | 71 | 225 | 127 | NAN |
| 58 | 0 | 86 | 160 | 96 | NAN |
| 64 | 3 | 79 | NAN | NAN | NAN |
| 74 | 1 | 80 | 187 | 65 | 5,0 |
| 66 | 0 | 81 | 173 | 61 | NAN |
| 60 | 2 | 75 | NAN | NAN | NAN |
| 74 | 1 | 73 | 136 | 82 | NAN |
| 60 | 1 | 78 | NAN | NAN | NAN |
| 61 | 0 | 87 | 196 | 61 | NAN |
| 73 | 0 | 77 | NAN | NAN | NAN |
| 64 | 0 | 83 | NAN | NAN | NAN |
| 56 | 0 | 86 | 176 | 68 | NAN |
| 55 | 1 | 90 | 205 | 124 | NAN |
| 68 | 1 | 91 | NAN | NAN | NAN |
| 76 | 0 | 78 | 199 | 99 | NAN |
| 68 | 0 | 88 | NAN | NAN | NAN |
| 66 | 0 | 88 | 188 | 109 | NAN |
| 50 | 0 | 77 | 175 | 87 | NAN |
| 67 | 1 | 91 | 147 | 42 | 5,2 |
| 51 | 0 | 88 | NAN | NAN | NAN |
| 58 | 0 | 74 | NAN | NAN | NAN |
| 69 | 0 | 82 | 194 | 77 | 4,6 |
| 75 | 3 | 84 | 172 | 56 | NAN |
| 72 | 1 | 78 | 194 | 76 | NAN |
| 74 | 1 | 73 | NAN | NAN | NAN |
| 65 | 1 | 77 | 156 | 56 | NAN |
| 82 | 0 | 78 | 165 | 69 | NAN |
| NAN | 1 | 67 | NAN | NAN | NAN |
| 52 | 0 | 82 | 152 | 60 | 5,1 |
| 48 | 0 | 80 | NAN | NAN | NAN |
| 69 | 1 | 90 | NAN | NAN | NAN |
| 53 | 3 | 85 | 193 | 54 | NAN |
| 57 | 0 | 86 | 148 | 32 | NAN |
| 69 | 0 | 82 | 191 | 67 | NAN |
| 72 | 0 | 88 | NAN | NAN | NAN |
| 65 | 0 | 85 | 166 | 74 | NAN |
| 69 | 2 | 78 | 161 | 63 | NAN |
| 59 | 0 | 74 | 159 | 92 | NAN |
| 76 | 0 | 88 | 200 | 61 | NAN |
| 56 | 2 | 84 | 164 | 44 | NAN |
| 58 | 0 | 72 | 175 | 162 | NAN |
| 78 | 2 | 88 | 143 | 45 | NAN |
| 69 | 3 | 81 | 132 | 62 | NAN |
| 73 | 1 | 73 | NAN | NAN | NAN |
| 69 | 0 | 89 | 182 | 80 | NAN |
| 74 | 3 | 75 | 175 | 58 | NAN |
| NAN | 0 | 85 | 203 | 58 | NAN |
| 64 | 3 | 79 | 186 | 59 | NAN |
| 65 | 0 | 83 | 170 | 140 | NAN |
| 72 | 1 | 84 | 108 | 71 | NAN |

| | | | | | |
|---|---|---|---|---|---|
| 75 | 0 | 81 | 177 | 124 | 5,4 |
| 77 | 0 | 89 | 203 | 168 | NAN |
| 72 | 1 | 90 | 183 | 108 | NAN |
| 57 | 1 | 88 | 149 | 68 | NAN |
| 74 | 0 | 84 | 275 | 94 | NAN |
| 68 | 1 | 82 | 197 | 48 | NAN |
| 80 | 0 | 91 | 146 | 65 | NAN |
| 60 | 2 | 85 | 185 | 69 | NAN |
| 71 | 0 | 75 | NAN | NAN | NAN |
| 93 | 0 | 89 | 225 | 564 | NAN |
| 50 | 0 | 89 | 129 | 234 | NAN |
| 67 | 0 | 81 | 162 | 93 | NAN |
| 49 | 1 | 74 | 159 | 98 | NAN |
| 51 | 3 | 90 | 175 | 82 | NAN |
| 65 | 1 | 76 | NAN | NAN | NAN |
| 57 | 0 | 91 | 132 | 91 | 5,2 |

| PREG_NUTRITIONSCORE | MEDAS Score 12 gw | PREG_LIVESTYLE_SCORE | PREG_EXERCISE_SCORE |
|---|---|---|---|
| 6,00 | 5,00 | 5,00 | -1,00 |
| 5,00 | 6,00 | 3,00 | -2,00 |
| 4,00 | 5,00 | 5,00 | 1,00 |
| -1,00 | 5,00 | -3,00 | -2,00 |
| -1,00 | 4,00 | -3,00 | -2,00 |
| -3,00 | 4,00 | -5,00 | -2,00 |
| 1,00 | 5,00 | 1,00 | 0,00 |
| 2,00 | 5,00 | -1,00 | -3,00 |
| 5,00 | 5,00 | 3,00 | -2,00 |
| -3,00 | 4,00 | -3,00 | 0,00 |
| 3,00 | 9,00 | 1,00 | -2,00 |
| -1,00 | 4,00 | -3,00 | -2,00 |
| -3,00 | 4,00 | -5,00 | -2,00 |
| -1,00 | 5,00 | -3,00 | -2,00 |
| 1,00 | 6,00 | 0,00 | -1,00 |
| 2,00 | 6,00 | -1,00 | -3,00 |
| 3,00 | 4,00 | 5,00 | 2,00 |
| 1,00 | 2,00 | 1,00 | 0,00 |
| 0,00 | 5,00 | -2,00 | -2,00 |
| 3,00 | 5,00 | 1,00 | -2,00 |
| -5,00 | 3,00 | -7,00 | -2,00 |
| -3,00 | 2,00 | -2,00 | 1,00 |
| 1,00 | 4,00 | 0,00 | -1,00 |
| 9,00 | 9,00 | 8,00 | -1,00 |
| 2,00 | 4,00 | 1,00 | -1,00 |
| 0,00 | 4,00 | -2,00 | -2,00 |
| 1,00 | 3,00 | -1,00 | -2,00 |
| -2,00 | 6,00 | -5,00 | -3,00 |
| 2,00 | 4,00 | -1,00 | -3,00 |
| -3,00 | 4,00 | -5,00 | -2,00 |
| 4,00 | 6,00 | 4,00 | 0,00 |
| 6,00 | 7,00 | 5,00 | -1,00 |
| 1,00 | 4,00 | 1,00 | 0,00 |
| 2,00 | 5,00 | -1,00 | -3,00 |
| 1,00 | 4,00 | 1,00 | 0,00 |
| 2,00 | 5,00 | 3,00 | 1,00 |
| 9,00 | 11,00 | 7,00 | -2,00 |
| 3,00 | 5,00 | 1,00 | -2,00 |
| -1,00 | 5,00 | -3,00 | -2,00 |
| -4,00 | 4,00 | -5,00 | -1,00 |
| -5,00 | 3,00 | -8,00 | -3,00 |
| 1,00 | 3,00 | 1,00 | 0,00 |
| 3,00 | 6,00 | 0,00 | -3,00 |
| -1,00 | 4,00 | -2,00 | -1,00 |
| 8,00 | 8,00 | 5,00 | -3,00 |
| -1,00 | 4,00 | -4,00 | -3,00 |
| -6,00 | 2,00 | -8,00 | -2,00 |

| | | | |
|---|---|---|---|
| -1,00 | 5,00 | -2,00 | -1,00 |
| 0,00 | 4,00 | -2,00 | -2,00 |
| -4,00 | 2,00 | -7,00 | -3,00 |
| 2,00 | 3,00 | 2,00 | 0,00 |
| 5,00 | 9,00 | 2,00 | -3,00 |
| 3,00 | 5,00 | 1,00 | -2,00 |
| -2,00 | 5,00 | -4,00 | -2,00 |
| 4,00 | 7,00 | 1,00 | -3,00 |
| 2,00 | 6,00 | 1,00 | -1,00 |
| -2,00 | 5,00 | -3,00 | -1,00 |
| 4,00 | 5,00 | 3,00 | -1,00 |
| -3,00 | 4,00 | -6,00 | -3,00 |
| -4,00 | 3,00 | -7,00 | -3,00 |
| -2,00 | 5,00 | -3,00 | -1,00 |
| -3,00 | 3,00 | -5,00 | -2,00 |
| -1,00 | 4,00 | -4,00 | -3,00 |
| 5,00 | 7,00 | 3,00 | -2,00 |
| 5,00 | 5,00 | 3,00 | -2,00 |
| 7,00 | 8,00 | 5,00 | -2,00 |
| -1,00 | 5,00 | -1,00 | 0,00 |
| -1,00 | 5,00 | -3,00 | -2,00 |
| 1,00 | 4,00 | -1,00 | -2,00 |
| -3,00 | 2,00 | -5,00 | -2,00 |
| -6,00 | 3,00 | -6,00 | 0,00 |
| 1,00 | 5,00 | -1,00 | -2,00 |
| 1,00 | 6,00 | -1,00 | -2,00 |
| -1,00 | 3,00 | -3,00 | -2,00 |
| 0,00 | 5,00 | -3,00 | -3,00 |
| 5,00 | 5,00 | 4,00 | -1,00 |
| 3,00 | 5,00 | 1,00 | -2,00 |
| 3,00 | 6,00 | 2,00 | -1,00 |
| 2,00 | 5,00 | -1,00 | -3,00 |
| 6,00 | 8,00 | 4,00 | -2,00 |
| -1,00 | 3,00 | -3,00 | -2,00 |
| -5,00 | 2,00 | -6,00 | -1,00 |
| 0,00 | 2,00 | -2,00 | -2,00 |
| 2,00 | 7,00 | 0,00 | -2,00 |
| -6,00 | 3,00 | -8,00 | -2,00 |
| -1,00 | 4,00 | -2,00 | -1,00 |
| -4,00 | 3,00 | -7,00 | -3,00 |
| -1,00 | 4,00 | -4,00 | -3,00 |
| 5,00 | 7,00 | 5,00 | 0,00 |
| 2,00 | 4,00 | -1,00 | -3,00 |
| 1,00 | 5,00 | 1,00 | 0,00 |
| 8,00 | 6,00 | 7,00 | -1,00 |
| -4,00 | 4,00 | -6,00 | -2,00 |
| 1,00 | 6,00 | 0,00 | -1,00 |
| 1,00 | 4,00 | -1,00 | -2,00 |
| -1,00 | 6,00 | -3,00 | -2,00 |
| 3,00 | 6,00 | 1,00 | -2,00 |

| | | | |
|---|---|---|---|
| 3,00 | 4,00 | 1,00 | -2,00 |
| 1,00 | 5,00 | 0,00 | -1,00 |
| 7,00 | 8,00 | 4,00 | -3,00 |
| -4,00 | 4,00 | -7,00 | -3,00 |
| 2,00 | 5,00 | 0,00 | -2,00 |
| 1,00 | 4,00 | 0,00 | -1,00 |
| 2,00 | 6,00 | 0,00 | -2,00 |
| 6,00 | 8,00 | 3,00 | -3,00 |
| -6,00 | 1,00 | -9,00 | -3,00 |
| 5,00 | 6,00 | 5,00 | 0,00 |
| 4,00 | 10,00 | 1,00 | -3,00 |
| 2,00 | 4,00 | 0,00 | -2,00 |
| 5,00 | 6,00 | 6,00 | 1,00 |
| 3,00 | 6,00 | 0,00 | -3,00 |
| -3,00 | 3,00 | -6,00 | -3,00 |
| 5,00 | 7,00 | 5,00 | 0,00 |
| 3,00 | 7,00 | 0,00 | -3,00 |
| -2,00 | 4,00 | -5,00 | -3,00 |
| -3,00 | 3,00 | -6,00 | -3,00 |
| 2,00 | 5,00 | -1,00 | -3,00 |
| 4,00 | 5,00 | 2,00 | -2,00 |
| 0,00 | 4,00 | -2,00 | -2,00 |
| 0,00 | 5,00 | -2,00 | -2,00 |
| 3,00 | 7,00 | 3,00 | 0,00 |
| 2,00 | 5,00 | 0,00 | -2,00 |
| 0,00 | 4,00 | -1,00 | -1,00 |
| 3,00 | 6,00 | 1,00 | -2,00 |
| -6,00 | 3,00 | -9,00 | -3,00 |
| -2,00 | 5,00 | -3,00 | -1,00 |
| -1,00 | 7,00 | -1,00 | 0,00 |
| 1,00 | 3,00 | 0,00 | -1,00 |
| 5,00 | 5,00 | 4,00 | -1,00 |
| 5,00 | 5,00 | 2,00 | -3,00 |
| NAN | 8,00 | NAN | -2,00 |
| 1,00 | 5,00 | -1,00 | -2,00 |
| 1,00 | 5,00 | -2,00 | -3,00 |
| 2,00 | 5,00 | -1,00 | -3,00 |
| -1,00 | 4,00 | -2,00 | -1,00 |
| 0,00 | 4,00 | -3,00 | -3,00 |
| -4,00 | 2,00 | -6,00 | -2,00 |
| 1,00 | 5,00 | -1,00 | -2,00 |
| -2,00 | 6,00 | -4,00 | -2,00 |
| 6,00 | 7,00 | 5,00 | -1,00 |
| 4,00 | 4,00 | 2,00 | -2,00 |
| 2,00 | 4,00 | 2,00 | 0,00 |
| 2,00 | 4,00 | 1,00 | -1,00 |
| -1,00 | 4,00 | -3,00 | -2,00 |
| -1,00 | 4,00 | -3,00 | -2,00 |
| -3,00 | 3,00 | -5,00 | -2,00 |
| 4,00 | 6,00 | 2,00 | -2,00 |

| | | | |
|---|---|---|---|
| -4,00 | 4,00 | -6,00 | -2,00 |
| -3,00 | 5,00 | -5,00 | -2,00 |
| 5,00 | 7,00 | 5,00 | 0,00 |
| -1,00 | 5,00 | -4,00 | -3,00 |
| 3,00 | 6,00 | 2,00 | -1,00 |
| -1,00 | 4,00 | -2,00 | -1,00 |
| 2,00 | 7,00 | -1,00 | -3,00 |
| 4,00 | 6,00 | 1,00 | -3,00 |
| -4,00 | 5,00 | -6,00 | -2,00 |
| 2,00 | 4,00 | 0,00 | -2,00 |
| 3,00 | 4,00 | 0,00 | -3,00 |
| -3,00 | 4,00 | -5,00 | -2,00 |
| -4,00 | 2,00 | -6,00 | -2,00 |
| 4,00 | 8,00 | 4,00 | 0,00 |
| -3,00 | 3,00 | -5,00 | -2,00 |
| -6,00 | 4,00 | -8,00 | -2,00 |
| -2,00 | 3,00 | -4,00 | -2,00 |
| -1,00 | 6,00 | -4,00 | -3,00 |
| 2,00 | 3,00 | 0,00 | -2,00 |
| 5,00 | 8,00 | 2,00 | -3,00 |
| 5,00 | 5,00 | 3,00 | -2,00 |
| 2,00 | 7,00 | 0,00 | -2,00 |
| -1,00 | 3,00 | -2,00 | -1,00 |
| 2,00 | 6,00 | 0,00 | -2,00 |
| 3,00 | 6,00 | 1,00 | -2,00 |
| 4,00 | 6,00 | 3,00 | -1,00 |
| 4,00 | 5,00 | 5,00 | 1,00 |
| 4,00 | 6,00 | 3,00 | -1,00 |
| 1,00 | 6,00 | 0,00 | -1,00 |
| 2,00 | 6,00 | 2,00 | 0,00 |
| 6,00 | 8,00 | 7,00 | 1,00 |
| 2,00 | 5,00 | 0,00 | -2,00 |
| 6,00 | 7,00 | 3,00 | -3,00 |
| -5,00 | 3,00 | -8,00 | -3,00 |
| 0,00 | 3,00 | -1,00 | -1,00 |
| 5,00 | 5,00 | 2,00 | -3,00 |
| 2,00 | 5,00 | 0,00 | -2,00 |
| -1,00 | 6,00 | -3,00 | -2,00 |
| -3,00 | 3,00 | -6,00 | -3,00 |
| 1,00 | 3,00 | -2,00 | -3,00 |
| 6,00 | 5,00 | 3,00 | -3,00 |
| 0,00 | 5,00 | -3,00 | -3,00 |
| -3,00 | 4,00 | -4,00 | -1,00 |
| 0,00 | 6,00 | -2,00 | -2,00 |
| -2,00 | 3,00 | -3,00 | -1,00 |
| -3,00 | 5,00 | -6,00 | -3,00 |
| -4,00 | 3,00 | -5,00 | -1,00 |
| 1,00 | 5,00 | 0,00 | -1,00 |
| 2,00 | 4,00 | -1,00 | -3,00 |
| -1,00 | 5,00 | -3,00 | -2,00 |

| | | | |
|---|---|---|---|
| 3,00 | 6,00 | 1,00 | -2,00 |
| 0,00 | 4,00 | -1,00 | -1,00 |
| -3,00 | 4,00 | -5,00 | -2,00 |
| 5,00 | 6,00 | 3,00 | -2,00 |
| 4,00 | 6,00 | 2,00 | -2,00 |
| 3,00 | 7,00 | 1,00 | -2,00 |
| 0,00 | 3,00 | -3,00 | -3,00 |
| 0,00 | 5,00 | -3,00 | -3,00 |
| 9,00 | 10,00 | 8,00 | -1,00 |
| 1,00 | 5,00 | 1,00 | 0,00 |
| 4,00 | 6,00 | 2,00 | -2,00 |
| 0,00 | 4,00 | -3,00 | -3,00 |
| -1,00 | 4,00 | -4,00 | -3,00 |
| -6,00 | 2,00 | -9,00 | -3,00 |
| 1,00 | 6,00 | -1,00 | -2,00 |
| -1,00 | 5,00 | -3,00 | -2,00 |
| 5,00 | 7,00 | 3,00 | -2,00 |
| 4,00 | 5,00 | 3,00 | -1,00 |
| -1,00 | 4,00 | -3,00 | -2,00 |
| -5,00 | 4,00 | -7,00 | -2,00 |
| 1,00 | 6,00 | 0,00 | -1,00 |
| -2,00 | 3,00 | -3,00 | -1,00 |
| 2,00 | 5,00 | 0,00 | -2,00 |
| -2,00 | 5,00 | -4,00 | -2,00 |
| -1,00 | 3,00 | -2,00 | -1,00 |
| -1,00 | 5,00 | -1,00 | 0,00 |
| 0,00 | 4,00 | -3,00 | -3,00 |
| -4,00 | 5,00 | -7,00 | -3,00 |
| -2,00 | 4,00 | -3,00 | -1,00 |
| -7,00 | 2,00 | -10,00 | -3,00 |
| 0,00 | 2,00 | -2,00 | -2,00 |
| 1,00 | 6,00 | -1,00 | -2,00 |
| 1,00 | 6,00 | -1,00 | -2,00 |
| -3,00 | 3,00 | -5,00 | -2,00 |
| -1,00 | 5,00 | -4,00 | -3,00 |
| -2,00 | 4,00 | -5,00 | -3,00 |
| -2,00 | 4,00 | -4,00 | -2,00 |
| 0,00 | 6,00 | -1,00 | -1,00 |
| 4,00 | 9,00 | 2,00 | -2,00 |
| -2,00 | 5,00 | -4,00 | -2,00 |
| 7,00 | 7,00 | 5,00 | -2,00 |
| -1,00 | 5,00 | -2,00 | -1,00 |
| 0,00 | 6,00 | -2,00 | -2,00 |
| 3,00 | 5,00 | 1,00 | -2,00 |
| -1,00 | 4,00 | -3,00 | -2,00 |
| 4,00 | 7,00 | 2,00 | -2,00 |
| 1,00 | 5,00 | 0,00 | -1,00 |
| 2,00 | 7,00 | -1,00 | -3,00 |
| 2,00 | 6,00 | 1,00 | -1,00 |
| 2,00 | 6,00 | -1,00 | -3,00 |

| | | | |
|---|---|---|---|
| -3,00 | 5,00 | -4,00 | -1,00 |
| -1,00 | 6,00 | -3,00 | -2,00 |
| -4,00 | 4,00 | -6,00 | -2,00 |
| -1,00 | 4,00 | -4,00 | -3,00 |
| -2,00 | 5,00 | -5,00 | -3,00 |
| -1,00 | 5,00 | -4,00 | -3,00 |
| 4,00 | 6,00 | 1,00 | -3,00 |
| -2,00 | 6,00 | -3,00 | -1,00 |
| -5,00 | 4,00 | -6,00 | -1,00 |
| 9,00 | 11,00 | 6,00 | -3,00 |
| -2,00 | 5,00 | -2,00 | 0,00 |
| -2,00 | 4,00 | -4,00 | -2,00 |
| 0,00 | 5,00 | -3,00 | -3,00 |
| 1,00 | 6,00 | -2,00 | -3,00 |
| 0,00 | 5,00 | -2,00 | -2,00 |
| -2,00 | 5,00 | -4,00 | -2,00 |
| -4,00 | 3,00 | -6,00 | -2,00 |
| -1,00 | 4,00 | 0,00 | 1,00 |
| 4,00 | 5,00 | 2,00 | -2,00 |
| 4,00 | 7,00 | 3,00 | -1,00 |
| -2,00 | 6,00 | -5,00 | -3,00 |
| 3,00 | 5,00 | 1,00 | -2,00 |
| -2,00 | 2,00 | -5,00 | -3,00 |
| -1,00 | 3,00 | -3,00 | -2,00 |
| 2,00 | 7,00 | 1,00 | -1,00 |
| 2,00 | 4,00 | -1,00 | -3,00 |
| -5,00 | 3,00 | -7,00 | -2,00 |
| -4,00 | 3,00 | -7,00 | -3,00 |
| 2,00 | 6,00 | -1,00 | -3,00 |
| 2,00 | 6,00 | 0,00 | -2,00 |
| 3,00 | 7,00 | 3,00 | 0,00 |
| 2,00 | 6,00 | 0,00 | -2,00 |
| 3,00 | 8,00 | 2,00 | -1,00 |
| 2,00 | 5,00 | -1,00 | -3,00 |
| 1,00 | 4,00 | -2,00 | -3,00 |
| 1,00 | 9,00 | -1,00 | -2,00 |
| -3,00 | NAN | -5,00 | -2,00 |
| 6,00 | 9,00 | 5,00 | -1,00 |
| 0,00 | 6,00 | -2,00 | -2,00 |
| 7,00 | 7,00 | 4,00 | -3,00 |
| 4,00 | 7,00 | 2,00 | -2,00 |
| 1,00 | 4,00 | -2,00 | -3,00 |
| 0,00 | 4,00 | -1,00 | -1,00 |
| 0,00 | 5,00 | -2,00 | -2,00 |
| 4,00 | 6,00 | 1,00 | -3,00 |
| 3,00 | 7,00 | 1,00 | -2,00 |
| -1,00 | 7,00 | -3,00 | -2,00 |
| -2,00 | 4,00 | -5,00 | -3,00 |
| -1,00 | 6,00 | -1,00 | 0,00 |
| -4,00 | 5,00 | -6,00 | -2,00 |

| | | | |
|---|---|---|---|
| 1,00 | 6,00 | -2,00 | -3,00 |
| 2,00 | 6,00 | 0,00 | -2,00 |
| 5,00 | 8,00 | 2,00 | -3,00 |
| 2,00 | 8,00 | 1,00 | -1,00 |
| 1,00 | 6,00 | 1,00 | 0,00 |
| 1,00 | 4,00 | -2,00 | -3,00 |
| 3,00 | 6,00 | 1,00 | -2,00 |
| -2,00 | 3,00 | -5,00 | -3,00 |
| -4,00 | 4,00 | -7,00 | -3,00 |
| 2,00 | 4,00 | 2,00 | 0,00 |
| 1,00 | 6,00 | -2,00 | -3,00 |
| 6,00 | NAN | 3,00 | -3,00 |
| 3,00 | NAN | 1,00 | -2,00 |
| 2,00 | 6,00 | -1,00 | -3,00 |
| -2,00 | 3,00 | -3,00 | -1,00 |
| 7,00 | 9,00 | 4,00 | -3,00 |

| SG24_NUTRITION_SCORE | SG24_LIVESTYLE_SCORE | SG24_MEDDIET_SCORE |
|---|---|---|
| 5,00 | 5,00 | 6,00 |
| 0,00 | -2,00 | 3,00 |
| 3,00 | 3,00 | 4,00 |
| -1,00 | -4,00 | 5,00 |
| -3,00 | -6,00 | 3,00 |
| -3,00 | -5,00 | 3,00 |
| 0,00 | -1,00 | 3,00 |
| 5,00 | 2,00 | 4,00 |
| 4,00 | 2,00 | 4,00 |
| -1,00 | -2,00 | 5,00 |
| 8,00 | 5,00 | 10,00 |
| 4,00 | 1,00 | 6,00 |
| 0,00 | -3,00 | 4,00 |
| 3,00 | 1,00 | 3,00 |
| 2,00 | 1,00 | 6,00 |
| 1,00 | -2,00 | 4,00 |
| 5,00 | 3,00 | 6,00 |
| 3,00 | 1,00 | 3,00 |
| -1,00 | -3,00 | 2,00 |
| 3,00 | 1,00 | 6,00 |
| 1,00 | 0,00 | 5,00 |
| -4,00 | -5,00 | 1,00 |
| NAN | NAN | NAN |
| 11,00 | 8,00 | 8,00 |
| 1,00 | -1,00 | 4,00 |
| 0,00 | -2,00 | 4,00 |
| -3,00 | -4,00 | 4,00 |
| -3,00 | -5,00 | 4,00 |
| -1,00 | -4,00 | 4,00 |
| -1,00 | -4,00 | 5,00 |
| 3,00 | 2,00 | 6,00 |
| 8,00 | 8,00 | 7,00 |
| 2,00 | -1,00 | 5,00 |
| 2,00 | 1,00 | 6,00 |
| 0,00 | -2,00 | 3,00 |
| 8,00 | 6,00 | 6,00 |
| 11,00 | 10,00 | 10,00 |
| 4,00 | 3,00 | 5,00 |
| 3,00 | 2,00 | 3,00 |
| 3,00 | 2,00 | 5,00 |
| -2,00 | -4,00 | 2,00 |
| -1,00 | -3,00 | 5,00 |
| -2,00 | -5,00 | 4,00 |
| -2,00 | -2,00 | 4,00 |
| 10,00 | 7,00 | 8,00 |
| -2,00 | -4,00 | 3,00 |
| 0,00 | 1,00 | 6,00 |

| | | |
|---|---|---|
| -2,00 | -2,00 | 4,00 |
| 0,00 | 0,00 | 3,00 |
| -3,00 | -5,00 | 2,00 |
| 5,00 | 3,00 | 4,00 |
| 5,00 | 5,00 | 5,00 |
| -1,00 | -3,00 | 3,00 |
| 2,00 | 0,00 | 3,00 |
| 7,00 | 5,00 | 5,00 |
| 0,00 | -1,00 | 4,00 |
| 1,00 | 0,00 | 6,00 |
| 6,00 | 5,00 | 7,00 |
| 0,00 | -2,00 | 4,00 |
| -3,00 | -5,00 | 3,00 |
| -2,00 | -4,00 | 6,00 |
| 2,00 | 0,00 | 4,00 |
| 7,00 | 4,00 | 7,00 |
| 3,00 | 1,00 | 5,00 |
| 3,00 | 1,00 | 3,00 |
| 4,00 | 2,00 | 6,00 |
| -2,00 | -1,00 | 5,00 |
| 0,00 | -1,00 | 6,00 |
| 1,00 | -1,00 | 5,00 |
| -1,00 | -1,00 | 2,00 |
| -4,00 | -6,00 | 2,00 |
| 1,00 | 1,00 | 5,00 |
| 1,00 | -1,00 | 3,00 |
| 5,00 | 4,00 | 5,00 |
| 2,00 | 0,00 | 8,00 |
| 1,00 | 2,00 | 4,00 |
| -1,00 | -3,00 | 4,00 |
| 6,00 | 5,00 | 6,00 |
| -3,00 | -4,00 | 5,00 |
| 6,00 | 5,00 | 4,00 |
| -3,00 | -5,00 | 2,00 |
| -1,00 | -2,00 | 6,00 |
| -1,00 | -4,00 | 2,00 |
| 4,00 | 2,00 | 7,00 |
| -6,00 | -7,00 | 2,00 |
| 1,00 | 0,00 | 4,00 |
| -3,00 | -6,00 | 5,00 |
| -3,00 | -6,00 | 2,00 |
| 6,00 | 4,00 | 5,00 |
| -4,00 | -6,00 | 2,00 |
| 0,00 | -1,00 | 7,00 |
| 7,00 | 6,00 | 6,00 |
| -3,00 | -5,00 | 2,00 |
| 0,00 | -3,00 | 5,00 |
| 5,00 | 2,00 | 6,00 |
| 3,00 | 0,00 | 7,00 |
| 2,00 | 0,00 | 4,00 |

| | | |
|---|---|---|
| 5,00 | 3,00 | 6,00 |
| 3,00 | 2,00 | 5,00 |
| 3,00 | 1,00 | 6,00 |
| -7,00 | -8,00 | 2,00 |
| -2,00 | -4,00 | 4,00 |
| -1,00 | -2,00 | 5,00 |
| 3,00 | 2,00 | 5,00 |
| 5,00 | 2,00 | 5,00 |
| -1,00 | -3,00 | 2,00 |
| 2,00 | -1,00 | 4,00 |
| -1,00 | -4,00 | 6,00 |
| 0,00 | -1,00 | 5,00 |
| 8,00 | 7,00 | 6,00 |
| 1,00 | -2,00 | 7,00 |
| -3,00 | -6,00 | 3,00 |
| 1,00 | 0,00 | 6,00 |
| 4,00 | 1,00 | 8,00 |
| 1,00 | -1,00 | 5,00 |
| 0,00 | -1,00 | 4,00 |
| 2,00 | -1,00 | 4,00 |
| 7,00 | 5,00 | 5,00 |
| -3,00 | -6,00 | 4,00 |
| 2,00 | 0,00 | 5,00 |
| 3,00 | 1,00 | 9,00 |
| 5,00 | 3,00 | 7,00 |
| 1,00 | -2,00 | 4,00 |
| 3,00 | 1,00 | 4,00 |
| 1,00 | -2,00 | 6,00 |
| 5,00 | 4,00 | 8,00 |
| 11,00 | 10,00 | 10,00 |
| 7,00 | 5,00 | 6,00 |
| 4,00 | 4,00 | 6,00 |
| 9,00 | 7,00 | 7,00 |
| 3,00 | 1,00 | 5,00 |
| 2,00 | -1,00 | 5,00 |
| 6,00 | 3,00 | 7,00 |
| 2,00 | 0,00 | 7,00 |
| 4,00 | 2,00 | 6,00 |
| -1,00 | -4,00 | 5,00 |
| 4,00 | 1,00 | 3,00 |
| 7,00 | 5,00 | 9,00 |
| 5,00 | 3,00 | 7,00 |
| 7,00 | 6,00 | 8,00 |
| 4,00 | 2,00 | 5,00 |
| 4,00 | 2,00 | 6,00 |
| 5,00 | 3,00 | 4,00 |
| 7,00 | 6,00 | 6,00 |
| 2,00 | 0,00 | 4,00 |
| 4,00 | 4,00 | 8,00 |
| 7,00 | 6,00 | 7,00 |

| | | |
|---|---|---|
| 2,00 | 1,00 | 7,00 |
| 1,00 | -1,00 | 8,00 |
| 7,00 | 8,00 | 6,00 |
| 0,00 | -3,00 | 3,00 |
| 9,00 | 6,00 | 10,00 |
| 2,00 | -1,00 | 4,00 |
| 8,00 | 7,00 | 8,00 |
| 9,00 | 8,00 | 8,00 |
| 4,00 | 2,00 | 6,00 |
| 11,00 | 11,00 | 10,00 |
| 6,00 | 3,00 | 6,00 |
| 0,00 | -2,00 | 5,00 |
| 5,00 | 3,00 | NAN |
| 5,00 | 2,00 | 8,00 |
| -1,00 | -2,00 | 6,00 |
| 1,00 | -1,00 | 6,00 |
| 3,00 | 1,00 | 5,00 |
| 3,00 | 0,00 | 7,00 |
| 5,00 | 5,00 | 5,00 |
| 8,00 | 5,00 | 11,00 |
| 3,00 | 2,00 | 6,00 |
| 8,00 | 7,00 | 8,00 |
| 5,00 | 2,00 | 6,00 |
| 5,00 | 2,00 | 6,00 |
| 3,00 | 1,00 | 7,00 |
| 8,00 | 8,00 | 7,00 |
| 11,00 | 10,00 | 11,00 |
| 8,00 | 7,00 | 9,00 |
| 0,00 | -1,00 | 4,00 |
| 5,00 | 3,00 | 7,00 |
| 8,00 | 7,00 | 7,00 |
| 7,00 | 4,00 | 7,00 |
| 7,00 | 5,00 | 8,00 |
| -3,00 | -5,00 | 4,00 |
| 8,00 | 6,00 | 8,00 |
| 5,00 | 2,00 | 8,00 |
| 4,00 | 2,00 | 4,00 |
| 2,00 | 1,00 | 8,00 |
| 4,00 | 1,00 | 6,00 |
| 4,00 | 1,00 | 5,00 |
| 9,00 | 7,00 | 8,00 |
| 4,00 | 2,00 | 5,00 |
| 6,00 | 4,00 | 7,00 |
| 3,00 | 1,00 | 9,00 |
| -2,00 | -4,00 | 3,00 |
| 12,00 | 9,00 | 11,00 |
| 0,00 | -2,00 | 4,00 |
| 2,00 | 0,00 | 4,00 |
| 2,00 | 0,00 | 5,00 |
| 2,00 | -1,00 | 4,00 |

| | | |
|---|---|---|
| 5,00 | 3,00 | 6,00 |
| 2,00 | 0,00 | 3,00 |
| 6,00 | 4,00 | 9,00 |
| 0,00 | -2,00 | 7,00 |
| 8,00 | 9,00 | 5,00 |
| 4,00 | 2,00 | 7,00 |
| 4,00 | 1,00 | 5,00 |
| -2,00 | -5,00 | 2,00 |
| 10,00 | 9,00 | 8,00 |
| 7,00 | 6,00 | 7,00 |
| 6,00 | 5,00 | 6,00 |
| 3,00 | 0,00 | 4,00 |
| 4,00 | 1,00 | 6,00 |
| 7,00 | 4,00 | 8,00 |
| 6,00 | 4,00 | 7,00 |
| 10,00 | 9,00 | 9,00 |
| 5,00 | 4,00 | 7,00 |
| 6,00 | 5,00 | 7,00 |
| 7,00 | 6,00 | 6,00 |
| 3,00 | 2,00 | 6,00 |
| 5,00 | 3,00 | 8,00 |
| 1,00 | -2,00 | 6,00 |
| 4,00 | 2,00 | 6,00 |
| 0,00 | -3,00 | 6,00 |
| 9,00 | 7,00 | 10,00 |
| -1,00 | -1,00 | 6,00 |
| 7,00 | 5,00 | 7,00 |
| 1,00 | 0,00 | 6,00 |
| 6,00 | 4,00 | 6,00 |
| 1,00 | -1,00 | 5,00 |
| 1,00 | 0,00 | 6,00 |
| 1,00 | 0,00 | 5,00 |
| 8,00 | 6,00 | 6,00 |
| 2,00 | 0,00 | 6,00 |
| 7,00 | 4,00 | 7,00 |
| 4,00 | 1,00 | 6,00 |
| 6,00 | 4,00 | 9,00 |
| 1,00 | -2,00 | 4,00 |
| 6,00 | 3,00 | 8,00 |
| 4,00 | 3,00 | 6,00 |
| 8,00 | 6,00 | 6,00 |
| 4,00 | 3,00 | 6,00 |
| -1,00 | -4,00 | 4,00 |
| 7,00 | 4,00 | 9,00 |
| 5,00 | 3,00 | 6,00 |
| 5,00 | 3,00 | 7,00 |
| 9,00 | 8,00 | 9,00 |
| 7,00 | 6,00 | 8,00 |
| 5,00 | 3,00 | 7,00 |
| 4,00 | 2,00 | 7,00 |

| | | |
|---|---|---|
| 5,00 | 3,00 | 9,00 |
| 6,00 | 4,00 | 8,00 |
| 3,00 | 1,00 | 5,00 |
| 2,00 | -1,00 | 3,00 |
| 4,00 | 2,00 | 6,00 |
| 1,00 | -2,00 | 4,00 |
| 3,00 | 1,00 | 6,00 |
| 2,00 | 0,00 | 7,00 |
| -2,00 | -3,00 | 3,00 |
| 10,00 | 8,00 | 11,00 |
| NAN | NAN | NAN |
| 3,00 | 1,00 | 5,00 |
| 1,00 | -1,00 | 5,00 |
| 6,00 | 4,00 | 8,00 |
| 5,00 | 3,00 | 7,00 |
| 4,00 | 2,00 | 8,00 |
| 5,00 | 4,00 | 5,00 |
| -1,00 | -2,00 | 4,00 |
| 9,00 | 9,00 | 7,00 |
| 4,00 | 1,00 | 5,00 |
| 5,00 | 3,00 | 7,00 |
| 3,00 | 2,00 | 6,00 |
| -2,00 | -4,00 | 4,00 |
| 0,00 | -3,00 | 7,00 |
| 6,00 | 4,00 | 9,00 |
| 6,00 | 3,00 | 7,00 |
| 2,00 | 0,00 | 4,00 |
| -2,00 | -4,00 | 6,00 |
| 10,00 | 8,00 | 8,00 |
| 8,00 | 6,00 | 10,00 |
| 4,00 | 2,00 | 7,00 |
| 5,00 | 3,00 | 7,00 |
| 2,00 | 2,00 | 9,00 |
| 5,00 | 2,00 | 7,00 |
| 4,00 | 2,00 | 6,00 |
| -2,00 | -5,00 | 4,00 |
| 2,00 | 0,00 | 4,00 |
| 8,00 | 6,00 | 10,00 |
| 5,00 | 4,00 | 8,00 |
| 4,00 | 1,00 | 7,00 |
| NAN | NAN | NAN |
| 2,00 | 1,00 | 5,00 |
| -2,00 | -4,00 | 4,00 |
| 0,00 | -2,00 | 7,00 |
| 5,00 | 2,00 | 8,00 |
| 8,00 | 6,00 | 8,00 |
| 9,00 | 8,00 | 8,00 |
| 7,00 | 4,00 | 6,00 |
| 8,00 | 7,00 | 9,00 |
| -6,00 | -7,00 | 4,00 |

| | | |
|---|---|---|
| 6,00 | 4,00 | 7,00 |
| 6,00 | 3,00 | 7,00 |
| 7,00 | 5,00 | 8,00 |
| 7,00 | 5,00 | 9,00 |
| 4,00 | 3,00 | 4,00 |
| 9,00 | 9,00 | 7,00 |
| 1,00 | -2,00 | 5,00 |
| 4,00 | 1,00 | 6,00 |
| 1,00 | -1,00 | NAN |
| 1,00 | 0,00 | 5,00 |
| 3,00 | 2,00 | 7,00 |
| 5,00 | 4,00 | NAN |
| 5,00 | 3,00 | NAN |
| 4,00 | 1,00 | 7,00 |
| 1,00 | -2,00 | 4,00 |
| 5,00 | 2,00 | 9,00 |

| MEDAS_SCORE_SG24 | SG24_EXERCISE_SCORE | NutritionScore_2años | EXERCISEScore_2años |
|---|---|---|---|
| 7,00 | 0,00 | 3,00 | 0,00 |
| 3,00 | -2,00 | 1,00 | -2,00 |
| 5,00 | 0,00 | 0,00 | -2,00 |
| 7,00 | -3,00 | 1,00 | -3,00 |
| 4,00 | -3,00 | -1,00 | -3,00 |
| 3,00 | -2,00 | NAN | -2,00 |
| 5,00 | -1,00 | 4,00 | -2,00 |
| 6,00 | -3,00 | 8,00 | -2,00 |
| 6,00 | -2,00 | 7,00 | -3,00 |
| 7,00 | -1,00 | -2,00 | -2,00 |
| 10,00 | -3,00 | 0,00 | -2,00 |
| 8,00 | -3,00 | 4,00 | -3,00 |
| 7,00 | -3,00 | 7,00 | -3,00 |
| 4,00 | -2,00 | -2,00 | -1,00 |
| 6,00 | -1,00 | 3,00 | 1,00 |
| 5,00 | -3,00 | 4,00 | -2,00 |
| 6,00 | -2,00 | 6,00 | 1,00 |
| 6,00 | -2,00 | 2,00 | -2,00 |
| 4,00 | -2,00 | 0,00 | -1,00 |
| 6,00 | -2,00 | 4,00 | -2,00 |
| 6,00 | -1,00 | 0,00 | -2,00 |
| 4,00 | -1,00 | -3,00 | 0,00 |
| NAN | NAN | 0,00 | -1,00 |
| 8,00 | -3,00 | 9,00 | -1,00 |
| 4,00 | -2,00 | 3,00 | -2,00 |
| 4,00 | -2,00 | 1,00 | -2,00 |
| 4,00 | -1,00 | -2,00 | -1,00 |
| 4,00 | -2,00 | -2,00 | -2,00 |
| 6,00 | -3,00 | 3,00 | -2,00 |
| 5,00 | -3,00 | -2,00 | -3,00 |
| 6,00 | -1,00 | 2,00 | 0,00 |
| 7,00 | 0,00 | 6,00 | -2,00 |
| 7,00 | -3,00 | 0,00 | -2,00 |
| 7,00 | -1,00 | 3,00 | -3,00 |
| 4,00 | -2,00 | 0,00 | -2,00 |
| 7,00 | -2,00 | 1,00 | -1,00 |
| 10,00 | -1,00 | 6,00 | -2,00 |
| 6,00 | -1,00 | 1,00 | -1,00 |
| 6,00 | -1,00 | 5,00 | -2,00 |
| 6,00 | -1,00 | 0,00 | -2,00 |
| 3,00 | -2,00 | 1,00 | -3,00 |
| 5,00 | -2,00 | 4,00 | -2,00 |
| 6,00 | -3,00 | 2,00 | -2,00 |
| 6,00 | 0,00 | 0,00 | 0,00 |
| 8,00 | -3,00 | 10,00 | -3,00 |
| 3,00 | -2,00 | 2,00 | -3,00 |
| 7,00 | 1,00 | -2,00 | -1,00 |

| | | | |
|---|---|---|---|
| 6,00 | 0,00 | -1,00 | -2,00 |
| 6,00 | 0,00 | 5,00 | -3,00 |
| 3,00 | -2,00 | NAN | -2,00 |
| 5,00 | -2,00 | 4,00 | 1,00 |
| 6,00 | 0,00 | NAN | -2,00 |
| 5,00 | -2,00 | 1,00 | -1,00 |
| 5,00 | -2,00 | -3,00 | -1,00 |
| 5,00 | -2,00 | 6,00 | -2,00 |
| 4,00 | -1,00 | -1,00 | -3,00 |
| 8,00 | -1,00 | 1,00 | -1,00 |
| 8,00 | -1,00 | 4,00 | -3,00 |
| 6,00 | -2,00 | -1,00 | -3,00 |
| 5,00 | -2,00 | -1,00 | -1,00 |
| 7,00 | -2,00 | 0,00 | 0,00 |
| 6,00 | -2,00 | 0,00 | -2,00 |
| 8,00 | -3,00 | 3,00 | -2,00 |
| 5,00 | -2,00 | 5,00 | -2,00 |
| 5,00 | -2,00 | 6,00 | -2,00 |
| 7,00 | -2,00 | 7,00 | -2,00 |
| 6,00 | 1,00 | -2,00 | -2,00 |
| 6,00 | -1,00 | -3,00 | -1,00 |
| 6,00 | -2,00 | 6,00 | -1,00 |
| 4,00 | 0,00 | 3,00 | -2,00 |
| 5,00 | -2,00 | 0,00 | -1,00 |
| 5,00 | 0,00 | 2,00 | 0,00 |
| 5,00 | -2,00 | 0,00 | -1,00 |
| 7,00 | -1,00 | NAN | NAN |
| 9,00 | -2,00 | NAN | NAN |
| 6,00 | 1,00 | 6,00 | -1,00 |
| 7,00 | -2,00 | 2,00 | -2,00 |
| 8,00 | -1,00 | 2,00 | -1,00 |
| 6,00 | -1,00 | -4,00 | -1,00 |
| 7,00 | -1,00 | 4,00 | 0,00 |
| 3,00 | -2,00 | 1,00 | -2,00 |
| 7,00 | -1,00 | 6,00 | -2,00 |
| 5,00 | -3,00 | -2,00 | -2,00 |
| 8,00 | -2,00 | 7,00 | -3,00 |
| 2,00 | -1,00 | 0,00 | -2,00 |
| 6,00 | -1,00 | -1,00 | -1,00 |
| 7,00 | -3,00 | -2,00 | -1,00 |
| 4,00 | -3,00 | 1,00 | -3,00 |
| 6,00 | -2,00 | 3,00 | -2,00 |
| 2,00 | -2,00 | -3,00 | -2,00 |
| 7,00 | -1,00 | -1,00 | -3,00 |
| 7,00 | -1,00 | 5,00 | -1,00 |
| 4,00 | -2,00 | NAN | NAN |
| 8,00 | -3,00 | 0,00 | -3,00 |
| 7,00 | -3,00 | 1,00 | -1,00 |
| 8,00 | -3,00 | 2,00 | -2,00 |
| 6,00 | -2,00 | 6,00 | -1,00 |

| | | | |
|---|---|---|---|
| 8,00 | -2,00 | 2,00 | -2,00 |
| 6,00 | -1,00 | 1,00 | -1,00 |
| 6,00 | -2,00 | 3,00 | 0,00 |
| 3,00 | -1,00 | -3,00 | 0,00 |
| 6,00 | -2,00 | 0,00 | -1,00 |
| 6,00 | -1,00 | -5,00 | -1,00 |
| 7,00 | -1,00 | NAN | NAN |
| 8,00 | -3,00 | 7,00 | -3,00 |
| 5,00 | -2,00 | -4,00 | -2,00 |
| 4,00 | -3,00 | 0,00 | -1,00 |
| 7,00 | -3,00 | 0,00 | -1,00 |
| 8,00 | -1,00 | 1,00 | -1,00 |
| 7,00 | -1,00 | 6,00 | -1,00 |
| 7,00 | -3,00 | 4,00 | -3,00 |
| 5,00 | -3,00 | 0,00 | -2,00 |
| 8,00 | -1,00 | -1,00 | -1,00 |
| 8,00 | -3,00 | 7,00 | -2,00 |
| 7,00 | -2,00 | -3,00 | -1,00 |
| 5,00 | -1,00 | -3,00 | -2,00 |
| 5,00 | -3,00 | 3,00 | -1,00 |
| 6,00 | -2,00 | 3,00 | -2,00 |
| 6,00 | -3,00 | 2,00 | -2,00 |
| 8,00 | -2,00 | 2,00 | 1,00 |
| 10,00 | -2,00 | 2,00 | -3,00 |
| 9,00 | -2,00 | 1,00 | -2,00 |
| 6,00 | -3,00 | 4,00 | -2,00 |
| 6,00 | -2,00 | 2,00 | -1,00 |
| 7,00 | -3,00 | 1,00 | -3,00 |
| 9,00 | -1,00 | 2,00 | -1,00 |
| 10,00 | -1,00 | 4,00 | -1,00 |
| 6,00 | -2,00 | 0,00 | -2,00 |
| 7,00 | 0,00 | 1,00 | -3,00 |
| 8,00 | -2,00 | 2,00 | -2,00 |
| 5,00 | -2,00 | 4,00 | -2,00 |
| 6,00 | -3,00 | 0,00 | -2,00 |
| 7,00 | -3,00 | 3,00 | -1,00 |
| 8,00 | -2,00 | 3,00 | -1,00 |
| 8,00 | -2,00 | 5,00 | -1,00 |
| 6,00 | -3,00 | 0,00 | -3,00 |
| 5,00 | -3,00 | -4,00 | -2,00 |
| 10,00 | -2,00 | 5,00 | -3,00 |
| 7,00 | -2,00 | 0,00 | -2,00 |
| 9,00 | -1,00 | 7,00 | -2,00 |
| 5,00 | -2,00 | 2,00 | -1,00 |
| 7,00 | -2,00 | 1,00 | -3,00 |
| 6,00 | -2,00 | 3,00 | -3,00 |
| 7,00 | -1,00 | 1,00 | -1,00 |
| 6,00 | -2,00 | -1,00 | -1,00 |
| 9,00 | 0,00 | 4,00 | -2,00 |
| 7,00 | -1,00 | 4,00 | -3,00 |

| | | | |
|---|---|---|---|
| 8,00 | -1,00 | 0,00 | -1,00 |
| 8,00 | -2,00 | -2,00 | -1,00 |
| 9,00 | 1,00 | 10,00 | -1,00 |
| 5,00 | -3,00 | 0,00 | -2,00 |
| 10,00 | -3,00 | 2,00 | -2,00 |
| 4,00 | -3,00 | 6,00 | -1,00 |
| 8,00 | -1,00 | 2,00 | -2,00 |
| 8,00 | -1,00 | NAN | NAN |
| 6,00 | -2,00 | -3,00 | -2,00 |
| 10,00 | 0,00 | 3,00 | 0,00 |
| 7,00 | -3,00 | NAN | NAN |
| 5,00 | -2,00 | 1,00 | -3,00 |
| NAN | -2,00 | 2,00 | -2,00 |
| 7,00 | -3,00 | 0,00 | -1,00 |
| 8,00 | -1,00 | -7,00 | -1,00 |
| 8,00 | -2,00 | -1,00 | -2,00 |
| 7,00 | -2,00 | 1,00 | -2,00 |
| 7,00 | -3,00 | -2,00 | -3,00 |
| 7,00 | 0,00 | 3,00 | -1,00 |
| 11,00 | -3,00 | 7,00 | -3,00 |
| 8,00 | -1,00 | 5,00 | -1,00 |
| 9,00 | -1,00 | 4,00 | -2,00 |
| 9,00 | -3,00 | NAN | NAN |
| 6,00 | -3,00 | 0,00 | -3,00 |
| 7,00 | -2,00 | -1,00 | 0,00 |
| 8,00 | 0,00 | 6,00 | -1,00 |
| 12,00 | -1,00 | 9,00 | -2,00 |
| 10,00 | -1,00 | 5,00 | -1,00 |
| 7,00 | -1,00 | 0,00 | -2,00 |
| 8,00 | -2,00 | 4,00 | 0,00 |
| 9,00 | -1,00 | 7,00 | 0,00 |
| 8,00 | -3,00 | 5,00 | 0,00 |
| 8,00 | -2,00 | 7,00 | -2,00 |
| 6,00 | -2,00 | 0,00 | -3,00 |
| 10,00 | -2,00 | 6,00 | -1,00 |
| 9,00 | -3,00 | 6,00 | -2,00 |
| 4,00 | -2,00 | 3,00 | -2,00 |
| 8,00 | -1,00 | 2,00 | 0,00 |
| 7,00 | -3,00 | 3,00 | -3,00 |
| 6,00 | -3,00 | 5,00 | -2,00 |
| 9,00 | -2,00 | 7,00 | -2,00 |
| 7,00 | -2,00 | -2,00 | -3,00 |
| 7,00 | -2,00 | -4,00 | -2,00 |
| 9,00 | -2,00 | 8,00 | -1,00 |
| 5,00 | -2,00 | 4,00 | -2,00 |
| 11,00 | -3,00 | 5,00 | -2,00 |
| 6,00 | -2,00 | -5,00 | 0,00 |
| 4,00 | -2,00 | 0,00 | -2,00 |
| 7,00 | -2,00 | -1,00 | -2,00 |
| 4,00 | -3,00 | 4,00 | -3,00 |

| | | | |
|---|---|---|---|
| 7,00 | -2,00 | 8,00 | -1,00 |
| 6,00 | -2,00 | 1,00 | -1,00 |
| 9,00 | -2,00 | 0,00 | -3,00 |
| 7,00 | -2,00 | 2,00 | -1,00 |
| 7,00 | 1,00 | 7,00 | -3,00 |
| 7,00 | -2,00 | 5,00 | -1,00 |
| 5,00 | -3,00 | 4,00 | -2,00 |
| 4,00 | -3,00 | -1,00 | -2,00 |
| 9,00 | -1,00 | 8,00 | -1,00 |
| 7,00 | -1,00 | 4,00 | -2,00 |
| 6,00 | -1,00 | 5,00 | -3,00 |
| 5,00 | -3,00 | 5,00 | -1,00 |
| 7,00 | -3,00 | 3,00 | -3,00 |
| 10,00 | -3,00 | NAN | NAN |
| 7,00 | -2,00 | 3,00 | -3,00 |
| 10,00 | -1,00 | 1,00 | -1,00 |
| 8,00 | -1,00 | 2,00 | -2,00 |
| 8,00 | -1,00 | 3,00 | -1,00 |
| 7,00 | -1,00 | 4,00 | -2,00 |
| 7,00 | -1,00 | 2,00 | 0,00 |
| 8,00 | -2,00 | -1,00 | -1,00 |
| 7,00 | -3,00 | 5,00 | -2,00 |
| 7,00 | -2,00 | 0,00 | -2,00 |
| 6,00 | -3,00 | -4,00 | -2,00 |
| 10,00 | -2,00 | 4,00 | -1,00 |
| 8,00 | 0,00 | 1,00 | -1,00 |
| 8,00 | -2,00 | 1,00 | -3,00 |
| 8,00 | -1,00 | -2,00 | -3,00 |
| 7,00 | -2,00 | 11,00 | -2,00 |
| 7,00 | -2,00 | NAN | NAN |
| 7,00 | -1,00 | 3,00 | -1,00 |
| 6,00 | -1,00 | 4,00 | -2,00 |
| 9,00 | -2,00 | 5,00 | -1,00 |
| 8,00 | -2,00 | 1,00 | -3,00 |
| 8,00 | -3,00 | 2,00 | -2,00 |
| 7,00 | -3,00 | -2,00 | -3,00 |
| 9,00 | -2,00 | 0,00 | -1,00 |
| 6,00 | -3,00 | -2,00 | -2,00 |
| 9,00 | -3,00 | 3,00 | -3,00 |
| 6,00 | -1,00 | -1,00 | 0,00 |
| 8,00 | -2,00 | 8,00 | -2,00 |
| 9,00 | -1,00 | 6,00 | 0,00 |
| 4,00 | -3,00 | -2,00 | 1,00 |
| 9,00 | -3,00 | 6,00 | -3,00 |
| 7,00 | -2,00 | 8,00 | -3,00 |
| 7,00 | -2,00 | NAN | NAN |
| 10,00 | -1,00 | 9,00 | -1,00 |
| 9,00 | -1,00 | -1,00 | -3,00 |
| 7,00 | -2,00 | 8,00 | -1,00 |
| 8,00 | -2,00 | 6,00 | -2,00 |

| | | | |
|---|---|---|---|
| 9,00 | -2,00 | 1,00 | -1,00 |
| 8,00 | -2,00 | 2,00 | -1,00 |
| 7,00 | -2,00 | -1,00 | -2,00 |
| 5,00 | -3,00 | 5,00 | -2,00 |
| 7,00 | -2,00 | 4,00 | -1,00 |
| 6,00 | -3,00 | 5,00 | -2,00 |
| 7,00 | -2,00 | 5,00 | -2,00 |
| 9,00 | -2,00 | 6,00 | 0,00 |
| 3,00 | -1,00 | 0,00 | -1,00 |
| 11,00 | -2,00 | 10,00 | -3,00 |
| NAN | NAN | 4,00 | -1,00 |
| 7,00 | -2,00 | 1,00 | -2,00 |
| 7,00 | -2,00 | 6,00 | -3,00 |
| 8,00 | -2,00 | 3,00 | -2,00 |
| 7,00 | -2,00 | 3,00 | 0,00 |
| 7,00 | -2,00 | 2,00 | -1,00 |
| 5,00 | -1,00 | -1,00 | -1,00 |
| 5,00 | -1,00 | 0,00 | -1,00 |
| 10,00 | 0,00 | 4,00 | -1,00 |
| 6,00 | -3,00 | 3,00 | -2,00 |
| 8,00 | -2,00 | 3,00 | -2,00 |
| 7,00 | -1,00 | 3,00 | -1,00 |
| 5,00 | -2,00 | 1,00 | -3,00 |
| 8,00 | -3,00 | 0,00 | -1,00 |
| 10,00 | -2,00 | 8,00 | -2,00 |
| 7,00 | -3,00 | NAN | NAN |
| 7,00 | -2,00 | NAN | NAN |
| 8,00 | -2,00 | -5,00 | -3,00 |
| 9,00 | -2,00 | 6,00 | -1,00 |
| 10,00 | -2,00 | 1,00 | 0,00 |
| 7,00 | -2,00 | 7,00 | 0,00 |
| 9,00 | -2,00 | -1,00 | -2,00 |
| 9,00 | 0,00 | 1,00 | -1,00 |
| 7,00 | -3,00 | 1,00 | -3,00 |
| 8,00 | -2,00 | 9,00 | -2,00 |
| 5,00 | -3,00 | 2,00 | -1,00 |
| 4,00 | -2,00 | -2,00 | -1,00 |
| 10,00 | -2,00 | 9,00 | -3,00 |
| 9,00 | -1,00 | 7,00 | -1,00 |
| 7,00 | -3,00 | 7,00 | -2,00 |
| NAN | NAN | 8,00 | -2,00 |
| 6,00 | -1,00 | 0,00 | -2,00 |
| 5,00 | -2,00 | NAN | NAN |
| 7,00 | -2,00 | 0,00 | -2,00 |
| 9,00 | -3,00 | 3,00 | -2,00 |
| 8,00 | -2,00 | 9,00 | -1,00 |
| 10,00 | -1,00 | 5,00 | -3,00 |
| 7,00 | -3,00 | -3,00 | -3,00 |
| 10,00 | -1,00 | 1,00 | -2,00 |
| 4,00 | -1,00 | -1,00 | -2,00 |

| | | | |
|---|---|---|---|
| 9,00 | -2,00 | 0,00 | -1,00 |
| 8,00 | -3,00 | 9,00 | -3,00 |
| 9,00 | -2,00 | 2,00 | -3,00 |
| 9,00 | -2,00 | 5,00 | -2,00 |
| 5,00 | -1,00 | NAN | NAN |
| 9,00 | 0,00 | 4,00 | -3,00 |
| 7,00 | -3,00 | -1,00 | -2,00 |
| 8,00 | -3,00 | 5,00 | -1,00 |
| NAN | -2,00 | 0,00 | -3,00 |
| 7,00 | -1,00 | -1,00 | -2,00 |
| 7,00 | -1,00 | 4,00 | -1,00 |
| NAN | -1,00 | 8,00 | 0,00 |
| NAN | -2,00 | 2,00 | -1,00 |
| 9,00 | -3,00 | 3,00 | -1,00 |
| 6,00 | -3,00 | 1,00 | -2,00 |
| 11,00 | -3,00 | -5,00 | -2,00 |

| identificacion_micros | IDENTIFICACTION | Log2mir222_2ddCt_g1_24s | Log2mir103_2ddCt_g1_24s |
|---|---|---|---|
| PDG0005 | PDG0005 | 1,13 | 0,92 |
| NAN | PDG0014 | NAN | NAN |
| PDG0021 | PDG0021 | 1,38 | 0,55 |
| PDG0022 | PDG0022 | 1,57 | 0,48 |
| PDG0023 | PDG0023 | 1,83 | 1,20 |
| PDG0028 | PDG0028 | 1,71 | 1,24 |
| NAN | PDG0039 | NAN | NAN |
| PDG0045 | PDG0045 | 1,93 | 1,17 |
| PDG0048 | PDG0048 | 1,53 | 1,58 |
| PDG0049 | PDG0049 | NAN | 1,91 |
| PDG0050 | PDG0050 | 1,76 | 1,99 |
| PDG0053 | PDG0053 | 2,12 | 1,38 |
| PDG0055 | PDG0055 | -2,10 | -1,02 |
| PDG0058 | PDG0058 | -1,59 | -1,37 |
| PDG0060 | PDG0060 | -1,86 | -2,13 |
| PDG0077 | PDG0077 | -3,41 | -2,29 |
| NAN | PDG0081 | NAN | NAN |
| PDG0083 | PDG0083 | -1,78 | -4,91 |
| PDG0095 | PDG0095 | -2,42 | -1,32 |
| PDG0096 | PDG0096 | NAN | -2,73 |
| PDG0097 | PDG0097 | NAN | -1,64 |
| PDG0104 | PDG0104 | -2,05 | -2,67 |
| PDG0105 | PDG0105 | -1,58 | -1,70 |
| NAN | PDG0110 | NAN | NAN |
| PDG0112 | PDG0112 | 1,83 | 0,21 |
| PDG0114 | PDG0114 | -0,47 | -0,33 |
| PDG0123 | PDG0123 | NAN | NAN |
| PDG0131 | PDG0131 | -1,52 | -2,48 |
| NAN | PDG0132 | NAN | NAN |
| NAN | PDG0133 | NAN | NAN |
| PDG0135 | PDG0135 | -0,88 | -1,94 |
| PDG0136 | PDG0136 | -0,88 | -1,79 |
| NAN | PDG0151 | NAN | NAN |
| NAN | PDG0152 | NAN | NAN |
| PDG0156 | PDG0156 | -1,32 | -1,49 |
| PDG0164 | PDG0164 | 0,97 | 0,84 |
| PDG0165 | PDG0165 | -0,57 | -0,85 |
| PDG0173 | PDG0173 | 0,80 | -0,94 |
| PDG0179 | PDG0179 | 1,09 | 0,58 |
| NAN | PDG0182 | NAN | NAN |
| NAN | PDG0189 | NAN | NAN |
| PDG0192 | PDG0192 | 1,20 | 0,73 |
| NAN | PDG0195 | NAN | NAN |
| NAN | PDG0197 | NAN | NAN |
| NAN | PDG0207 | NAN | NAN |
| NAN | PDG0217 | NAN | NAN |
| NAN | PDG0223 | NAN | NAN |

| | | | |
|---|---|---|---|
| PDG0224 | PDG0224 | NAN | 1,35 |
| NAN | PDG0230 | NAN | NAN |
| PDG0231 | PDG0231 | 1,27 | 0,73 |
| NAN | PDG0233 | NAN | NAN |
| NAN | PDG0243 | NAN | NAN |
| NAN | PDG0244 | NAN | NAN |
| NAN | PDG0251 | NAN | NAN |
| NAN | PDG0256 | NAN | NAN |
| NAN | PDG0260 | NAN | NAN |
| NAN | PDG0261 | NAN | NAN |
| NAN | PDG0262 | NAN | NAN |
| NAN | PDG0264 | NAN | NAN |
| PDG0265 | PDG0265 | 0,63 | -1,41 |
| PDG0266 | PDG0266 | 0,75 | -1,20 |
| PDG0271 | PDG0271 | 0,50 | -1,39 |
| NAN | PDG0276 | NAN | NAN |
| PDG0279 | PDG0279 | -0,70 | -1,48 |
| PDG0283 | PDG0283 | 0,14 | -0,94 |
| PDG0284 | PDG0284 | 1,37 | -2,19 |
| PDG0294 | PDG0294 | 0,07 | -1,14 |
| NAN | PDG0297 | NAN | NAN |
| PDG0298 | PDG0298 | 0,12 | -2,05 |
| PDG0299 | PDG0299 | -0,71 | -0,93 |
| PDG0301 | PDG0301 | -0,26 | -1,74 |
| PDG0306 | PDG0306 | -0,67 | -2,10 |
| NAN | PDG0311 | NAN | NAN |
| PDG0316 | PDG0316 | -0,37 | -2,19 |
| PDG0320 | PDG0320 | 1,28 | -0,28 |
| PDG0328 | PDG0328 | -0,55 | -1,26 |
| PDG0332 | PDG0332 | 1,24 | 0,46 |
| PDG0335 | PDG0335 | -0,03 | -0,15 |
| PDG0336 | PDG0336 | -0,06 | -1,09 |
| PDG0337 | PDG0337 | 1,17 | 0,34 |
| NAN | PDG0339 | NAN | NAN |
| PDG0341 | PDG0341 | 0,22 | -0,55 |
| NAN | PDG0344 | NAN | NAN |
| NAN | PDG0348 | NAN | NAN |
| PDG0351 | PDG0351 | 2,04 | -0,25 |
| PDG0352 | PDG0352 | 2,64 | 0,40 |
| PDG0355 | PDG0355 | 2,46 | 0,90 |
| PDG0358 | PDG0358 | 1,91 | -0,15 |
| PDG0363 | PDG0363 | 1,09 | -0,85 |
| PDG0364 | PDG0364 | 2,93 | 0,01 |
| PDG0370 | PDG0370 | 2,73 | 0,76 |
| PDG0371 | PDG0371 | 2,74 | 1,16 |
| NAN | PDG0376 | NAN | NAN |
| NAN | PDG0377 | NAN | NAN |
| NAN | PDG0381 | NAN | NAN |
| NAN | PDG0385 | NAN | NAN |
| PDG0390 | PDG0390 | 2,21 | -0,16 |

| | | | |
|---|---|---|---|
| PDG0394 | PDG0394 | 2,42 | -0,12 |
| PDG0396 | PDG0396 | -2,98 | -3,07 |
| PDG0401 | PDG0401 | -2,65 | -2,79 |
| PDG0404 | PDG0404 | -2,12 | -2,12 |
| PDG0406 | PDG0406 | -1,38 | -2,32 |
| NAN | PDG0411 | NAN | NAN |
| PDG0412 | PDG0412 | -2,08 | -2,86 |
| PDG0414 | PDG0414 | -1,90 | -2,53 |
| PDG0418 | PDG0418 | -1,23 | -1,31 |
| NAN | PDG0419 | NAN | NAN |
| PDG0426 | PDG0426 | 0,12 | NAN |
| PDG0430 | PDG0430 | -2,28 | -2,37 |
| PDG0437 | PDG0437 | 1,43 | -0,08 |
| PDG0442 | PDG0442 | NAN | -3,16 |
| PDG0444 | PDG0444 | 1,40 | 0,26 |
| PDG0448 | PDG0448 | 1,97 | 2,43 |
| NAN | PDG0456 | NAN | NAN |
| PDG0464 | PDG0464 | 1,38 | 0,79 |
| PDG0465 | PDG0465 | 2,34 | 1,31 |
| PDG0471 | PDG0471 | 2,29 | 0,99 |
| PDG0474 | PDG0474 | 1,65 | 0,50 |
| PDG0477 | PDG0477 | 2,36 | 2,10 |
| PDG0482 | PDG0482 | NAN | 0,88 |
| PDG0484 | PDG0484 | 0,35 | 0,74 |
| PDG0485 | PDG0485 | 1,98 | 0,13 |
| NAN | PDG0491 | NAN | NAN |
| PDG0492 | PDG0492 | 2,07 | 1,10 |
| PDG0496 | PDG0496 | 1,96 | 1,01 |
| PDG0501 | PDG0501 | 0,98 | -0,25 |
| PDG0505 | PDG0505 | NAN | 0,32 |
| PDG0506 | PDG0506 | 1,29 | 1,02 |
| PDG0509 | PDG0509 | 1,93 | 1,56 |
| PDG0512 | PDG0512 | -0,19 | -1,90 |
| PDG0515 | PDG0515 | 0,28 | 0,19 |
| PDG0524 | PDG0524 | 2,47 | 1,71 |
| PDG0527 | PDG0527 | -0,85 | -2,87 |
| NAN | PDG0528 | NAN | NAN |
| PDG0529 | PDG0529 | 2,36 | 1,50 |
| PDG0535 | PDG0535 | 0,12 | 1,12 |
| PDG0536 | PDG0536 | NAN | NAN |
| PDG0538 | PDG0538 | -1,73 | -2,10 |
| PDG0542 | PDG0542 | 0,28 | -2,07 |
| PDG0544 | PDG0544 | 0,01 | 1,00 |
| PDG0545 | PDG0545 | 0,23 | -3,08 |
| PDG0547 | PDG0547 | -1,07 | NAN |
| PDG0552 | PDG0552 | NAN | NAN |
| PDG0555 | PDG0555 | -1,20 | -2,18 |
| PDG0572 | PDG0572 | 1,00 | 1,36 |
| PDG0574 | PDG0574 | 5,02 | NAN |
| PDG0576 | PDG0576 | -0,13 | 1,12 |

| | | | |
|---|---|---|---|
| PDG0580 | PDG0580 | 0,14 | 0,55 |
| PDG0581 | PDG0581 | 0,71 | -1,27 |
| NAN | PDG0588 | NAN | NAN |
| PDG0590 | PDG0590 | 0,37 | -1,46 |
| NAN | PDG0591 | NAN | NAN |
| PDG0592 | PDG0592 | 1,16 | 1,40 |
| NAN | PDG0601 | NAN | NAN |
| PDG0608 | PDG0608 | 0,67 | -1,50 |
| PDG0611 | PDG0611 | 0,52 | -0,96 |
| PDG0612 | PDG0612 | 1,33 | 2,81 |
| PDG0613 | PDG0613 | 1,01 | -0,50 |
| PDG0615 | PDG0615 | 0,41 | -1,35 |
| PDG0616 | PDG0616 | 1,10 | 2,58 |
| PDG0622 | PDG0622 | 0,29 | -0,49 |
| PDG0623 | PDG0623 | 0,33 | -1,26 |
| PDG0624 | PDG0624 | 0,80 | -0,85 |
| PDG0626 | PDG0626 | 0,48 | -0,87 |
| PDG0632 | PDG0632 | 0,24 | -1,50 |
| PDG0640 | PDG0640 | 1,42 | 1,14 |
| PDG0642 | PDG0642 | 0,47 | 0,58 |
| PDG0644 | PDG0644 | 1,19 | 2,49 |
| PDG0647 | PDG0647 | 1,28 | 1,82 |
| PDG0649 | PDG0649 | 1,43 | 1,90 |
| PDG0660 | PDG0660 | 0,27 | -1,29 |
| PDG0664 | PDG0664 | 0,83 | 1,57 |
| PDG0666 | PDG0666 | 0,92 | 0,90 |
| PDG0673 | PDG0673 | 1,55 | 0,39 |
| PDG0676 | PDG0676 | 1,87 | 1,98 |
| PDG0679 | PDG0679 | 1,61 | 0,65 |
| PDG0680 | PDG0680 | 1,24 | 0,87 |
| PDG0683 | PDG0683 | 1,36 | 0,42 |
| PDG0684 | PDG0684 | 0,83 | -0,07 |
| PDG0685 | PDG0685 | 0,02 | -1,52 |
| PDG0687 | PDG0687 | 0,65 | 1,09 |
| PDG0688 | PDG0688 | 1,95 | 1,45 |
| PDG0691 | PDG0691 | 1,02 | 0,57 |
| NAN | PDG0692 | NAN | NAN |
| PDG0694 | PDG0694 | 1,45 | 0,33 |
| NAN | PDG0695 | NAN | NAN |
| NAN | PDG0696 | NAN | NAN |
| PDG0704 | PDG0704 | 0,42 | 1,78 |
| PDG0706 | PDG0706 | 0,06 | -2,17 |
| PDG0709 | PDG0709 | 0,07 | 0,48 |
| PDG0711 | PDG0711 | 1,79 | 1,22 |
| PDG0715 | PDG0715 | 1,48 | 3,26 |
| PDG0718 | PDG0718 | 3,97 | 3,05 |
| PDG0720 | PDG0720 | 0,84 | 1,86 |
| PDG0723 | PDG0723 | 1,82 | 2,47 |
| PDG0725 | PDG0725 | 1,44 | 1,98 |
| PDG0733 | PDG0733 | 1,32 | 1,97 |

| | | | |
|---|---|---|---|
| PDG0736 | PDG0736 | 2,14 | 2,92 |
| PDG0744 | PDG0744 | 1,10 | 2,37 |
| PDG0747 | PDG0747 | 2,54 | 2,45 |
| PDG0751 | PDG0751 | 2,30 | 2,72 |
| PDG0753 | PDG0753 | 1,89 | 2,10 |
| PDG0754 | PDG0754 | 1,81 | 2,43 |
| PDG0758 | PDG0758 | 1,44 | 1,47 |
| PDG0765 | PDG0765 | 1,21 | 3,97 |
| PDG0768 | PDG0768 | 1,51 | 1,32 |
| PDG0769 | PDG0769 | 0,98 | 2,27 |
| PDG0772 | PDG0772 | 1,96 | 2,34 |
| PDG0777 | PDG0777 | 1,16 | 1,42 |
| PDG0779 | PDG0779 | 1,15 | 1,91 |
| PDG0789 | PDG0789 | 1,28 | 1,64 |
| PDG0792 | PDG0792 | 1,20 | 2,73 |
| PDG0793 | PDG0793 | 1,12 | 1,39 |
| PDG0807 | PDG0807 | 1,53 | 2,23 |
| PDG0809 | PDG0809 | 2,02 | 2,99 |
| PDG0814 | PDG0814 | 1,37 | 2,24 |
| PDG0815 | PDG0815 | 1,87 | 2,24 |
| PDG0817 | PDG0817 | 0,29 | -0,47 |
| PDG0820 | PDG0820 | 1,28 | 1,85 |
| PDG0821 | PDG0821 | 2,36 | 3,27 |
| PDG0822 | PDG0822 | 0,93 | 0,81 |
| PDG0828 | PDG0828 | 1,80 | 4,19 |
| PDG0829 | PDG0829 | 1,81 | 2,84 |
| PDG0831 | PDG0831 | -0,07 | 0,44 |
| PDG0833 | PDG0833 | 2,05 | 2,32 |
| PDG0834 | PDG0834 | 1,20 | 1,74 |
| PDG0837 | PDG0837 | 0,29 | 0,53 |
| PDG0843 | PDG0843 | 0,54 | 1,85 |
| PDG0847 | PDG0847 | 1,34 | 2,82 |
| NAN | PDG0849 | NAN | NAN |
| PDG0851 | PDG0851 | 0,75 | 2,17 |
| PDG0852 | PDG0852 | 1,79 | 4,08 |
| PDG0857 | PDG0857 | 0,88 | 0,68 |
| PDG0860 | PDG0860 | 2,06 | 2,35 |
| PDG0868 | PDG0868 | 1,34 | 1,57 |
| PDG0869 | PDG0869 | 0,93 | 1,49 |
| PDG0871 | PDG0871 | 0,83 | -0,11 |
| PDG0874 | PDG0874 | 0,97 | 1,33 |
| PDG0877 | PDG0877 | -0,06 | 1,93 |
| PDG0878 | PDG0878 | 1,04 | 1,21 |
| PDG0885 | PDG0885 | 1,16 | -0,59 |
| PDG0886 | PDG0886 | 1,47 | 1,67 |
| PDG0896 | PDG0896 | 1,13 | 2,16 |
| PDG0899 | PDG0899 | 0,25 | 0,50 |
| PDG0900 | PDG0900 | -1,67 | -1,23 |
| PDG0901 | PDG0901 | -0,24 | -0,63 |
| PDG0902 | PDG0902 | 0,98 | 0,18 |

| | | | |
|---|---|---|---|
| PDG0907 | PDG0907 | 0,59 | -0,85 |
| PDG0909 | PDG0909 | 1,16 | 0,31 |
| PDG0910 | PDG0910 | 1,11 | -0,56 |
| PDG0911 | PDG0911 | 0,45 | -0,34 |
| PDG0912 | PDG0912 | -0,68 | -1,64 |
| PDG0915 | PDG0915 | -0,22 | -1,00 |
| PDG0922 | PDG0922 | 0,04 | 1,98 |
| PDG0923 | PDG0923 | 1,58 | 3,70 |
| PDG0929 | PDG0929 | 0,40 | 1,72 |
| PDG0930 | PDG0930 | -1,63 | -2,56 |
| PDG0932 | PDG0932 | NAN | NAN |
| PDG0933 | PDG0933 | 1,48 | 3,78 |
| PDG0940 | PDG0940 | 1,07 | 3,41 |
| PDG0941 | PDG0941 | 1,75 | 2,72 |
| PDG0944 | PDG0944 | 1,33 | 2,57 |
| PDG0945 | PDG0945 | 0,33 | 2,43 |
| PDG0946 | PDG0946 | 0,95 | 2,52 |
| PDG0952 | PDG0952 | 1,45 | 1,57 |
| PDG0956 | PDG0956 | 3,31 | 0,91 |
| PDG0963 | PDG0963 | 0,96 | 1,85 |
| PDG0971 | PDG0971 | 2,41 | 1,05 |
| PDG0974 | PDG0974 | 3,51 | 2,55 |
| PDG0975 | PDG0975 | 2,78 | 1,30 |
| PDG0977 | PDG0977 | 2,57 | 1,19 |
| PDG0979 | PDG0979 | 3,70 | 2,50 |
| PDG0984 | PDG0984 | 2,94 | 0,86 |
| PDG0985 | PDG0985 | 3,85 | 1,82 |
| PDG0988 | PDG0988 | 3,98 | 2,68 |
| PDG0992 | PDG0992 | 4,22 | 2,69 |
| PDG0993 | PDG0993 | 4,72 | 5,90 |
| PDG0998 | PDG0998 | 5,83 | 6,84 |
| PDG1001 | PDG1001 | 6,59 | 6,75 |
| PDG1002 | PDG1002 | 0,12 | 2,15 |
| PDG1034 | PDG1034 | 5,55 | 5,65 |
| PDG1043 | PDG1043 | 1,89 | 2,74 |
| PDG1045 | PDG1045 | 4,99 | 5,65 |
| PDG1050 | PDG1050 | 1,08 | 2,36 |
| PDG1051 | PDG1051 | 1,26 | 2,54 |
| PDG1053 | PDG1053 | 1,37 | 2,55 |
| NAN | PDG1054 | NAN | NAN |
| PDG1057 | PDG1057 | NAN | NAN |
| PDG1059 | PDG1059 | 1,33 | 1,70 |
| PDG1070 | PDG1070 | 4,89 | 5,54 |
| PDG1073 | PDG1073 | 2,28 | 2,89 |
| PDG1076 | PDG1076 | 4,16 | 3,62 |
| PDG1079 | PDG1079 | 5,63 | 5,54 |
| PDG1080 | PDG1080 | 4,81 | 4,61 |
| PDG1083 | PDG1083 | 4,41 | 4,53 |
| PDG1088 | PDG1088 | 5,88 | 5,91 |
| PDG1089 | PDG1089 | 5,80 | 5,96 |

| | | | |
|---|---|---|---|
| PDG1091 | PDG1091 | 5,46 | 5,60 |
| PDG1092 | PDG1092 | 5,54 | 5,21 |
| PDG1093 | PDG1093 | 5,32 | 4,86 |
| PDG1095 | PDG1095 | 1,80 | 2,25 |
| PDG1098 | PDG1098 | 2,33 | 2,47 |
| PDG1099 | PDG1099 | 1,42 | 1,32 |
| PDG1100 | PDG1100 | 1,23 | 2,26 |
| PDG1102 | PDG1102 | 2,00 | 1,69 |
| PDG1106 | PDG1106 | 1,30 | 0,75 |
| PDG1107 | PDG1107 | 1,14 | 3,28 |
| PDG1109 | PDG1109 | 2,50 | 1,39 |
| PDG1113 | PDG1113 | 2,53 | 1,40 |
| PDG1114 | PDG1114 | 2,77 | 2,27 |
| PDG1115 | PDG1115 | 1,35 | 0,56 |
| PDG1116 | PDG1116 | 1,71 | 1,37 |
| PDG1117 | PDG1117 | 1,42 | 0,78 |

| Log2mir29_2ddCt_g1_24s | Log2mir132_2ddct_g1_24s | Log2mir222_2ddCt_g1_2A |
|---|---|---|
| -0,37 | -0,45 | 1,57 |
| NAN | NAN | NAN |
| 0,32 | 0,02 | 1,51 |
| 0,09 | -0,20 | 1,68 |
| -0,05 | -0,40 | NAN |
| -0,14 | 0,66 | 2,29 |
| NAN | NAN | NAN |
| -0,58 | NAN | 2,80 |
| -0,05 | -0,58 | 0,69 |
| 0,36 | 0,89 | 1,48 |
| -0,34 | -0,06 | 1,08 |
| 0,44 | -0,02 | 1,48 |
| -1,80 | -0,11 | -1,65 |
| -2,88 | 0,36 | -0,11 |
| -1,88 | NAN | NAN |
| -2,54 | -0,06 | NAN |
| NAN | NAN | NAN |
| -2,62 | 0,58 | -1,39 |
| -2,52 | 1,70 | -1,36 |
| -3,07 | 1,42 | NAN |
| NAN | 0,82 | -2,81 |
| -2,78 | 1,59 | -2,68 |
| -4,21 | 0,63 | -1,87 |
| NAN | NAN | NAN |
| 1,09 | 1,51 | 1,45 |
| -0,95 | 0,91 | 0,39 |
| NAN | NAN | NAN |
| -1,06 | NAN | NAN |
| NAN | NAN | NAN |
| NAN | NAN | NAN |
| -1,25 | NAN | -0,48 |
| -0,61 | NAN | -0,03 |
| NAN | NAN | NAN |
| NAN | NAN | NAN |
| 0,08 | 0,00 | -1,41 |
| -0,07 | 1,85 | 0,33 |
| -0,78 | NAN | 0,19 |
| -1,49 | 1,84 | -0,30 |
| NAN | NAN | 1,26 |
| NAN | NAN | NAN |
| NAN | NAN | NAN |
| 2,12 | -0,19 | 1,34 |
| NAN | NAN | NAN |
| NAN | NAN | NAN |
| NAN | NAN | NAN |
| NAN | NAN | NAN |
| NAN | NAN | NAN |

| | | |
|---|---|---|
| NAN | NAN | NAN |
| NAN | NAN | NAN |
| 0,69 | NAN | -0,23 |
| NAN | NAN | NAN |
| NAN | NAN | NAN |
| NAN | NAN | NAN |
| NAN | NAN | NAN |
| NAN | NAN | NAN |
| NAN | NAN | NAN |
| NAN | NAN | NAN |
| NAN | NAN | NAN |
| NAN | NAN | NAN |
| 1,22 | -0,91 | 0,49 |
| 1,70 | -1,06 | 0,20 |
| -0,29 | -1,09 | 0,15 |
| NAN | NAN | NAN |
| -0,17 | -0,55 | -0,52 |
| 1,85 | -1,12 | 0,76 |
| 1,70 | -1,78 | 2,23 |
| 1,14 | -0,44 | 0,07 |
| NAN | NAN | NAN |
| 1,92 | -1,17 | 0,83 |
| -0,10 | 0,69 | -0,08 |
| 0,35 | -0,03 | -0,12 |
| -0,74 | -0,20 | -0,77 |
| NAN | NAN | NAN |
| -0,12 | 0,21 | 0,18 |
| 0,24 | 1,36 | 0,85 |
| 0,09 | 1,36 | 0,84 |
| -0,34 | NAN | 0,98 |
| 0,48 | 0,30 | 0,56 |
| 0,47 | 0,85 | 0,70 |
| 0,97 | 0,12 | 0,87 |
| NAN | NAN | NAN |
| -0,86 | NAN | 1,10 |
| NAN | NAN | NAN |
| NAN | NAN | NAN |
| 3,18 | NAN | 1,59 |
| 4,85 | NAN | 2,00 |
| 3,03 | NAN | 2,54 |
| 3,37 | 0,05 | 1,40 |
| 3,26 | NAN | 2,83 |
| 3,70 | 0,36 | 1,71 |
| 3,80 | NAN | 1,65 |
| 3,64 | 1,52 | 1,41 |
| NAN | NAN | NAN |
| NAN | NAN | NAN |
| NAN | NAN | NAN |
| NAN | NAN | NAN |
| 3,41 | 0,38 | 2,19 |

| | | |
|---|---|---|
| 3,81 | 1,28 | 3,11 |
| -0,08 | NAN | -2,42 |
| -0,08 | NAN | 0,29 |
| -0,58 | NAN | -0,58 |
| 1,64 | NAN | 0,25 |
| NAN | NAN | NAN |
| 0,26 | NAN | -1,69 |
| -0,95 | NAN | -1,55 |
| 0,00 | -0,06 | -2,67 |
| NAN | NAN | NAN |
| -0,64 | NAN | -1,27 |
| -1,66 | NAN | -2,94 |
| 0,53 | 0,73 | 2,34 |
| -2,20 | NAN | NAN |
| 0,74 | 0,15 | 1,50 |
| 1,02 | 0,48 | 1,91 |
| NAN | NAN | NAN |
| 0,03 | 0,65 | 0,42 |
| 1,12 | 0,90 | 2,53 |
| 1,39 | NAN | 1,06 |
| 0,13 | NAN | 1,95 |
| 1,21 | 0,36 | 2,22 |
| 1,43 | 0,91 | 2,48 |
| 0,01 | NAN | 1,98 |
| 0,10 | NAN | 1,18 |
| NAN | NAN | NAN |
| 0,93 | 0,51 | 1,62 |
| 0,76 | NAN | 0,46 |
| 0,22 | -0,71 | 1,53 |
| NAN | NAN | NAN |
| 1,90 | 0,35 | 0,67 |
| 2,00 | 0,53 | 2,65 |
| -1,34 | -0,17 | -0,54 |
| NAN | NAN | -0,14 |
| 1,44 | 1,12 | 1,64 |
| -1,15 | -0,31 | -1,06 |
| NAN | NAN | NAN |
| 1,26 | 0,34 | 2,09 |
| -2,40 | -0,98 | -0,67 |
| NAN | NAN | NAN |
| -1,84 | NAN | 0,98 |
| -1,21 | NAN | -0,35 |
| -2,00 | -1,71 | 2,53 |
| -1,41 | NAN | 0,34 |
| -2,73 | NAN | -0,27 |
| NAN | NAN | 5,20 |
| -1,27 | -0,89 | -1,01 |
| -2,14 | -0,19 | -0,29 |
| NAN | NAN | NAN |
| -2,83 | NAN | 0,81 |

| | | |
|---|---|---|
| 0,28 | -0,79 | NAN |
| -1,06 | 0,25 | -1,31 |
| NAN | NAN | NAN |
| -0,21 | NAN | -1,76 |
| NAN | NAN | NAN |
| 2,77 | NAN | 1,47 |
| NAN | NAN | NAN |
| -1,04 | 0,23 | -2,61 |
| -0,85 | 0,01 | 0,45 |
| -0,81 | 0,35 | 0,88 |
| 0,18 | 0,00 | -0,58 |
| 0,29 | -0,63 | 0,18 |
| -2,10 | -0,17 | 1,13 |
| -1,04 | -0,13 | 0,85 |
| -0,44 | -0,50 | 0,16 |
| 0,21 | -0,72 | -0,50 |
| 0,19 | -0,04 | -0,56 |
| 0,46 | 0,24 | -0,96 |
| 2,53 | NAN | 1,71 |
| -1,94 | -0,95 | 0,14 |
| -1,33 | 0,19 | 0,92 |
| 2,50 | NAN | 0,70 |
| -1,17 | 0,43 | 0,84 |
| 0,10 | -0,07 | -0,40 |
| -2,00 | -0,96 | 2,64 |
| 2,12 | 0,32 | 0,71 |
| -0,45 | 1,05 | 1,09 |
| 0,15 | 0,53 | 1,17 |
| -1,27 | -0,24 | 1,57 |
| -1,27 | 0,85 | 1,43 |
| -0,63 | 0,75 | 0,69 |
| -1,88 | 0,70 | 0,12 |
| 0,37 | -0,55 | -0,73 |
| 2,60 | 1,48 | 1,08 |
| -0,20 | 0,77 | 2,11 |
| -1,03 | 1,36 | 0,63 |
| NAN | NAN | NAN |
| -2,26 | 1,33 | 1,02 |
| NAN | NAN | NAN |
| NAN | NAN | NAN |
| -0,81 | NAN | 0,07 |
| -0,41 | -1,16 | 0,45 |
| 2,50 | 1,70 | 0,47 |
| -1,62 | 0,78 | 2,32 |
| -2,28 | NAN | 1,05 |
| -1,26 | 0,44 | 2,08 |
| 1,74 | 1,33 | 1,03 |
| -1,79 | 0,39 | NAN |
| -1,62 | -0,96 | 1,96 |
| -1,86 | -0,88 | 0,96 |

| | | |
|---|---|---|
| -1,48 | -0,21 | 2,37 |
| NAN | -0,36 | 1,50 |
| -1,01 | 0,13 | 1,36 |
| -0,93 | 0,94 | 1,53 |
| -1,74 | -0,60 | 2,44 |
| -2,11 | -0,58 | 1,04 |
| NAN | 0,00 | 1,38 |
| 0,12 | -1,20 | 1,03 |
| 3,02 | NAN | 1,18 |
| -0,82 | -0,85 | 0,31 |
| -1,28 | 0,20 | 1,99 |
| -1,58 | -0,52 | 0,93 |
| -1,56 | NAN | 0,86 |
| 2,76 | 0,85 | 1,55 |
| -2,96 | -0,96 | 0,75 |
| 2,44 | 0,91 | 1,49 |
| -1,79 | -0,07 | 1,53 |
| 1,30 | 0,37 | 1,63 |
| -1,16 | 0,27 | 1,32 |
| -2,51 | 0,51 | 2,04 |
| -0,13 | -0,26 | 0,24 |
| 0,48 | -0,72 | 0,79 |
| 0,40 | 0,15 | 1,19 |
| 1,04 | NAN | 1,80 |
| 1,26 | 0,18 | 1,00 |
| 1,06 | NAN | NAN |
| -0,15 | NAN | 0,76 |
| 0,33 | 0,24 | 2,19 |
| 0,07 | -0,09 | 2,64 |
| 0,01 | NAN | 0,84 |
| -0,37 | NAN | 1,51 |
| -0,25 | -0,41 | 1,96 |
| NAN | NAN | NAN |
| -1,99 | 1,02 | 1,34 |
| 0,44 | 0,58 | 0,66 |
| 1,45 | -0,55 | 2,18 |
| 0,98 | 0,89 | 0,95 |
| 1,49 | 0,01 | 1,31 |
| 1,15 | -0,50 | 0,87 |
| -0,47 | 0,56 | 2,92 |
| 1,04 | -1,02 | 1,57 |
| -0,44 | NAN | 1,21 |
| 1,27 | -0,20 | 2,14 |
| 2,88 | -0,88 | -0,11 |
| 1,52 | -0,04 | 0,86 |
| 0,71 | -0,22 | 0,61 |
| 1,25 | -1,53 | 1,11 |
| -2,34 | -1,76 | -0,06 |
| -1,51 | -1,26 | 0,12 |
| 0,09 | 0,31 | 0,75 |

| | | |
|---|---|---|
| -0,88 | -0,27 | 0,38 |
| -0,29 | 0,61 | 1,64 |
| -0,71 | 0,01 | 0,79 |
| -1,25 | -0,59 | 1,19 |
| -2,41 | -0,58 | 0,37 |
| -1,74 | -0,23 | 0,17 |
| 0,18 | -1,37 | 1,58 |
| 0,89 | -0,54 | 0,55 |
| 0,46 | 0,07 | 0,99 |
| 2,67 | -0,72 | 0,82 |
| NAN | NAN | 0,25 |
| 0,03 | 0,16 | 0,13 |
| 0,21 | -0,93 | 1,09 |
| 1,10 | 0,34 | 0,16 |
| 0,73 | 0,24 | 0,83 |
| 0,12 | -0,82 | 0,34 |
| 0,56 | -0,65 | 0,61 |
| 0,58 | NAN | 2,27 |
| 3,15 | 2,99 | 4,47 |
| 0,19 | NAN | 0,97 |
| 1,93 | 2,46 | 1,87 |
| 2,96 | 2,84 | 3,33 |
| 2,38 | 3,13 | 2,34 |
| 2,67 | 2,09 | 3,23 |
| 2,95 | 4,17 | 4,03 |
| 2,53 | 2,67 | NAN |
| 3,44 | NAN | 6,66 |
| 3,17 | 3,19 | 3,81 |
| 2,75 | NAN | 4,38 |
| 4,36 | 3,34 | 6,23 |
| 4,84 | 4,16 | 5,47 |
| 4,37 | 4,19 | 7,95 |
| 0,88 | NAN | 0,90 |
| 5,23 | 4,06 | 6,52 |
| 1,67 | NAN | 2,76 |
| 4,49 | 4,67 | 4,82 |
| 0,54 | NAN | 0,77 |
| 0,78 | NAN | 1,50 |
| 0,87 | NAN | NAN |
| NAN | NAN | NAN |
| NAN | NAN | -0,90 |
| 0,50 | NAN | 1,11 |
| 3,81 | 3,97 | 5,10 |
| 1,29 | NAN | 2,38 |
| 3,49 | 2,70 | 4,09 |
| 4,71 | 4,31 | 5,30 |
| 4,79 | 3,93 | 4,26 |
| 3,77 | 3,18 | 4,25 |
| 5,18 | 4,26 | 4,53 |
| 4,29 | 3,97 | 5,82 |

| | | |
|---|---|---|
| 4,86 | 4,04 | 5,46 |
| 5,25 | 4,12 | 6,66 |
| 3,15 | 3,66 | 5,16 |
| 3,56 | -0,23 | 1,52 |
| 1,05 | NAN | 1,68 |
| 3,45 | NAN | 1,13 |
| 2,70 | -0,07 | 1,06 |
| 3,46 | 0,21 | 1,04 |
| 3,09 | -1,06 | 2,33 |
| 0,24 | NAN | 0,97 |
| 2,60 | 0,29 | 2,38 |
| 3,19 | -0,08 | 1,51 |
| 2,78 | 0,65 | 1,41 |
| 2,63 | -0,52 | 1,19 |
| 2,69 | 0,00 | 1,15 |
| 0,99 | -0,46 | 2,70 |

| Log2mir103_2ddCt_g1_2A | Log2mir132_2ddct_g1_2A | Log2mir29_2ddCt_g1_2A |
|---|---|---|
| 0,68 | -0,12 | -0,28 |
| NAN | NAN | NAN |
| 1,99 | 0,52 | -0,26 |
| 2,40 | 0,27 | -0,45 |
| 3,00 | 0,81 | -0,21 |
| 3,20 | 0,61 | -0,52 |
| NAN | NAN | NAN |
| 3,07 | 1,38 | 0,20 |
| 2,01 | -0,64 | -0,67 |
| 2,14 | -0,02 | -0,51 |
| 1,81 | -0,60 | 0,07 |
| 2,14 | -0,26 | -0,11 |
| -1,74 | -0,10 | -2,06 |
| 0,60 | NAN | NAN |
| -1,88 | -0,69 | -2,72 |
| -1,32 | NAN | -1,47 |
| NAN | NAN | NAN |
| -1,30 | 0,83 | -2,58 |
| -0,47 | 1,49 | -2,97 |
| -2,43 | NAN | -3,03 |
| -3,46 | 2,55 | -0,76 |
| -0,92 | 0,62 | -2,76 |
| -2,11 | 0,60 | -3,48 |
| NAN | NAN | NAN |
| 2,06 | 1,04 | 0,84 |
| 0,57 | 0,28 | -0,61 |
| NAN | NAN | NAN |
| -2,07 | NAN | -0,92 |
| NAN | NAN | NAN |
| NAN | NAN | NAN |
| -1,30 | NAN | 0,15 |
| -0,78 | NAN | -1,79 |
| NAN | NAN | NAN |
| NAN | NAN | NAN |
| -1,07 | 0,37 | -1,38 |
| 0,87 | NAN | -0,41 |
| 0,34 | 0,63 | -0,52 |
| 0,60 | NAN | -1,13 |
| 1,08 | 1,61 | -0,96 |
| NAN | NAN | NAN |
| NAN | NAN | NAN |
| 0,32 | 0,00 | 1,96 |
| NAN | NAN | NAN |
| NAN | NAN | NAN |
| NAN | NAN | NAN |
| NAN | NAN | NAN |
| NAN | NAN | NAN |

| | | |
|---|---|---|
| NAN | NAN | NAN |
| NAN | NAN | NAN |
| -0,83 | NAN | 0,70 |
| NAN | NAN | NAN |
| NAN | NAN | NAN |
| NAN | NAN | NAN |
| NAN | NAN | NAN |
| NAN | NAN | NAN |
| NAN | NAN | NAN |
| NAN | NAN | NAN |
| NAN | NAN | NAN |
| NAN | NAN | NAN |
| -0,32 | -0,88 | 1,68 |
| NAN | -1,22 | 1,18 |
| -1,65 | -1,10 | 1,58 |
| NAN | NAN | NAN |
| -0,90 | 0,00 | -0,02 |
| -0,22 | -0,68 | 1,42 |
| -1,69 | -1,04 | 2,20 |
| -0,94 | -0,40 | 1,28 |
| NAN | NAN | NAN |
| -0,20 | -0,44 | 1,83 |
| -0,90 | 0,28 | 0,10 |
| -1,13 | 0,73 | 0,54 |
| -2,32 | 0,26 | 0,11 |
| NAN | NAN | NAN |
| 0,49 | 0,54 | 0,40 |
| 0,23 | 1,67 | 0,12 |
| 0,47 | 1,67 | 0,99 |
| 0,36 | NAN | -0,29 |
| 0,31 | 1,05 | 0,27 |
| -0,01 | 0,86 | 0,49 |
| -0,05 | 0,18 | 0,58 |
| NAN | NAN | NAN |
| 0,62 | NAN | -0,71 |
| NAN | NAN | NAN |
| NAN | NAN | NAN |
| -0,74 | -0,19 | 3,99 |
| 0,09 | NAN | 4,16 |
| 0,61 | NAN | 4,76 |
| -0,64 | 0,02 | 3,04 |
| 0,14 | NAN | 4,67 |
| 1,50 | NAN | 3,45 |
| 0,71 | NAN | 3,21 |
| -0,26 | NAN | 2,05 |
| NAN | NAN | NAN |
| NAN | NAN | NAN |
| NAN | NAN | NAN |
| NAN | NAN | NAN |
| 0,24 | 0,78 | 3,56 |

| | | |
|---|---|---|
| -0,02 | NAN | 2,97 |
| -0,94 | NAN | 0,61 |
| -1,69 | NAN | 1,42 |
| -0,91 | NAN | 0,30 |
| -0,17 | 0,75 | 1,40 |
| NAN | NAN | NAN |
| -0,95 | NAN | 0,39 |
| -1,40 | NAN | -0,79 |
| -2,12 | NAN | 0,15 |
| NAN | NAN | NAN |
| -1,56 | NAN | -2,54 |
| -2,12 | NAN | -2,24 |
| 2,11 | NAN | -1,41 |
| -1,47 | NAN | -2,12 |
| 1,08 | 0,31 | 0,94 |
| 2,91 | 0,46 | 0,55 |
| NAN | NAN | NAN |
| 0,33 | NAN | -0,06 |
| 2,72 | 0,77 | 1,21 |
| 1,37 | -0,83 | 0,64 |
| 1,69 | 0,63 | 1,04 |
| 2,79 | 0,67 | 0,92 |
| 2,00 | 0,61 | 1,23 |
| 0,38 | NAN | 0,80 |
| 0,51 | 0,80 | 0,60 |
| NAN | NAN | NAN |
| 1,75 | 0,86 | 0,84 |
| 0,28 | 0,94 | 0,74 |
| 0,56 | 0,70 | 1,77 |
| 2,41 | NAN | 0,22 |
| -0,94 | NAN | 1,49 |
| 1,06 | NAN | 1,57 |
| -1,48 | -1,64 | -2,20 |
| -0,10 | 0,10 | -2,08 |
| 1,78 | 1,65 | 1,25 |
| -1,64 | NAN | -0,99 |
| NAN | NAN | NAN |
| -0,14 | 1,00 | 1,92 |
| 1,08 | -1,30 | -3,09 |
| NAN | NAN | NAN |
| -1,70 | 1,00 | -1,42 |
| -2,31 | -0,08 | -0,44 |
| 3,02 | 0,52 | -1,78 |
| -0,13 | 0,09 | -1,56 |
| -2,18 | NAN | -1,14 |
| NAN | NAN | NAN |
| -1,32 | -2,02 | -2,67 |
| 1,12 | -0,78 | -2,95 |
| NAN | NAN | NAN |
| 2,28 | -0,10 | -1,91 |

| | | |
|---|---|---|
| 1,13 | NAN | 0,95 |
| -1,58 | -1,21 | -2,41 |
| NAN | NAN | NAN |
| -2,88 | -1,54 | -2,10 |
| NAN | NAN | NAN |
| 1,63 | NAN | 2,12 |
| NAN | NAN | NAN |
| -1,86 | NAN | -2,63 |
| 0,02 | NAN | -1,14 |
| 3,26 | -0,03 | -2,09 |
| -0,72 | NAN | -0,01 |
| -0,52 | NAN | -0,39 |
| 2,12 | 0,17 | -1,62 |
| 0,42 | -0,15 | -0,67 |
| -0,48 | -0,78 | -0,44 |
| -0,67 | -0,76 | 0,23 |
| -0,86 | -0,53 | -0,57 |
| -1,29 | NAN | 0,11 |
| 2,09 | 1,12 | 2,95 |
| 2,46 | -0,38 | -2,20 |
| 3,11 | 0,49 | -1,78 |
| 2,72 | NAN | 2,34 |
| 1,60 | -0,31 | -2,61 |
| -0,97 | NAN | 0,16 |
| 4,20 | 1,08 | -1,01 |
| 1,72 | 0,39 | 2,25 |
| 0,74 | NAN | -0,78 |
| 0,89 | 0,30 | -1,65 |
| 0,99 | -0,11 | -2,46 |
| 1,47 | 0,37 | -1,03 |
| 0,48 | NAN | -0,81 |
| 0,25 | 0,32 | -1,92 |
| -0,42 | -0,89 | -0,61 |
| -2,82 | NAN | -0,01 |
| 2,04 | NAN | -0,67 |
| 0,78 | 0,83 | -2,56 |
| NAN | NAN | NAN |
| 0,55 | NAN | -1,06 |
| NAN | NAN | NAN |
| NAN | NAN | NAN |
| 1,39 | NAN | -0,58 |
| -0,66 | -0,59 | 0,04 |
| 1,88 | 2,68 | 2,94 |
| 1,29 | NAN | -2,57 |
| 2,41 | -1,31 | -1,55 |
| 3,95 | -0,43 | -2,22 |
| 2,08 | NAN | 2,61 |
| NAN | NAN | NAN |
| 1,79 | -0,94 | -1,19 |
| 2,02 | 0,68 | NAN |

| | | |
|---|---|---|
| 3,30 | 0,45 | -1,67 |
| 3,12 | NAN | -2,25 |
| 2,20 | -0,63 | -2,02 |
| 2,63 | -0,70 | -2,03 |
| 4,07 | -0,23 | -2,38 |
| 2,87 | -1,34 | -2,33 |
| 3,10 | 0,43 | -2,85 |
| 3,92 | -0,81 | 0,57 |
| 1,88 | NAN | 2,37 |
| 2,39 | -0,50 | -2,07 |
| 2,97 | 1,02 | -2,01 |
| 2,02 | NAN | -1,74 |
| 2,22 | -0,66 | -1,55 |
| 1,40 | NAN | 2,68 |
| 2,40 | -0,92 | -1,44 |
| 1,91 | NAN | 1,82 |
| 2,70 | -0,09 | -0,77 |
| 3,65 | NAN | 0,37 |
| 1,43 | -0,16 | -2,78 |
| 2,57 | NAN | -1,15 |
| -0,15 | -0,22 | -0,02 |
| 3,68 | NAN | 0,09 |
| NAN | -0,47 | 0,34 |
| 0,91 | NAN | NAN |
| 3,89 | -0,49 | -0,71 |
| -0,20 | NAN | -0,42 |
| 1,78 | NAN | -0,38 |
| 3,02 | 0,45 | 0,04 |
| 4,08 | 1,54 | 1,04 |
| 1,42 | NAN | -0,12 |
| 2,60 | 1,72 | 0,63 |
| 3,72 | 0,35 | 0,25 |
| NAN | NAN | NAN |
| 3,25 | -0,81 | 0,02 |
| 3,63 | -0,41 | 0,25 |
| 3,51 | 0,67 | 1,65 |
| 1,48 | 0,13 | 0,85 |
| 2,08 | -0,55 | 1,58 |
| 1,39 | -0,74 | 1,96 |
| 0,37 | 1,42 | 0,90 |
| 2,58 | -0,17 | 0,82 |
| 2,53 | NAN | -0,18 |
| 1,19 | NAN | 1,87 |
| -0,46 | -1,31 | 2,25 |
| 1,61 | -0,84 | 0,97 |
| 2,38 | -1,13 | 0,84 |
| 1,73 | -0,27 | 0,40 |
| 0,72 | -0,16 | -0,68 |
| 0,19 | -0,05 | -1,09 |
| 0,57 | 0,68 | -1,55 |

| | | |
|---|---|---|
| 0,92 | -0,49 | -1,36 |
| -0,08 | 0,75 | -0,11 |
| -0,42 | 0,22 | -1,48 |
| -0,74 | 0,88 | -0,73 |
| -0,27 | 0,46 | -1,32 |
| -1,30 | 0,05 | -1,07 |
| 3,81 | -0,02 | 0,93 |
| 3,31 | -1,42 | -0,14 |
| 2,97 | -0,99 | 0,16 |
| -0,22 | -0,89 | 2,13 |
| 1,53 | NAN | 0,15 |
| 2,82 | -1,57 | 0,09 |
| 1,20 | NAN | -0,24 |
| 1,77 | -0,95 | -0,63 |
| 1,88 | NAN | -0,26 |
| 2,46 | -0,51 | 0,06 |
| 1,44 | -1,27 | 0,33 |
| 2,59 | -0,07 | 0,21 |
| 3,19 | 3,71 | 3,56 |
| 2,23 | NAN | 0,53 |
| 2,57 | 1,45 | 1,18 |
| 2,77 | 4,05 | 3,66 |
| 1,27 | 1,79 | 2,01 |
| 2,81 | 2,68 | 2,62 |
| 2,99 | NAN | 2,60 |
| 1,93 | 2,44 | 2,21 |
| 3,96 | 5,70 | 5,06 |
| 3,11 | NAN | 3,35 |
| 3,57 | NAN | 3,73 |
| 7,08 | 4,43 | 5,34 |
| 6,17 | 3,71 | 4,59 |
| 8,05 | NAN | 6,17 |
| 3,06 | NAN | 1,48 |
| 7,24 | 4,99 | 5,23 |
| 2,44 | NAN | 1,69 |
| 6,36 | NAN | 3,85 |
| 2,15 | NAN | 0,01 |
| 2,59 | NAN | 0,49 |
| 2,02 | NAN | NAN |
| NAN | NAN | NAN |
| 1,46 | NAN | 1,04 |
| 2,50 | NAN | 0,77 |
| 5,81 | 3,63 | 4,56 |
| 2,47 | NAN | 1,44 |
| 4,96 | 3,08 | 3,36 |
| 5,93 | 3,76 | 5,11 |
| NAN | 2,46 | 4,04 |
| 5,53 | 3,01 | 3,97 |
| 4,84 | 2,96 | 3,71 |
| 6,10 | 3,41 | 4,75 |

| | | |
|---|---|---|
| 4,61 | 3,21 | 5,39 |
| 6,43 | 4,89 | 6,13 |
| 5,69 | 4,21 | 5,15 |
| 2,66 | 0,55 | 3,48 |
| 2,69 | NAN | 0,91 |
| 2,52 | NAN | 2,49 |
| 2,44 | NAN | 3,14 |
| 2,03 | -1,64 | 2,48 |
| 0,98 | -0,34 | 2,68 |
| 2,47 | NAN | 0,00 |
| 2,07 | 0,14 | 2,45 |
| 1,74 | 0,09 | 2,15 |
| 2,21 | 0,39 | -0,03 |
| 1,74 | -1,30 | 2,40 |
| 2,08 | -0,30 | 1,56 |
| 3,14 | 0,29 | 1,21 |

| Adiponectin_leptin ratio 12 GW | Adiponectin_leptin ratio 24 GW |
|---|---|
| 8,21 | 2,15 |
| 0,18 | 0,47 |
| 1,42 | 1,19 |
| 1,31 | 0,84 |
| 3,13 | 2,00 |
| 11,58 | 2,36 |
| 7,55 | 4,46 |
| 0,26 | 0,26 |
| 1,68 | 1,22 |
| 1,05 | 0,54 |
| 5,12 | 1,40 |
| 1,51 | 2,27 |
| 6,30 | 2,52 |
| 1,00 | 1,23 |
| 7,89 | 4,94 |
| 7,26 | 8,28 |
| 5,73 | 1,37 |
| 2,72 | 2,10 |
| 9,29 | 4,82 |
| 0,47 | 0,50 |
| 9,96 | 5,85 |
| 3,27 | 1,10 |
| 0,90 | 0,67 |
| 4,22 | 1,24 |
| 1,06 | 0,81 |
| 6,54 | 2,67 |
| 3,39 | 1,16 |
| 2,90 | 1,24 |
| 11,33 | 3,82 |
| 4,44 | 1,87 |
| 0,62 | 0,37 |
| 3,26 | 1,48 |
| 7,27 | 1,28 |
| 2,18 | 1,17 |
| 0,86 | 0,45 |
| 6,05 | 1,08 |
| 18,83 | 12,04 |
| 0,45 | 0,36 |
| 0,38 | 0,28 |
| 1,24 | 0,96 |
| 0,33 | 0,24 |
| 1,31 | 1,65 |
| 0,93 | 0,56 |
| 2,96 | 2,29 |
| 5,54 | 4,16 |
| 0,95 | 0,91 |
| 15,52 | 2,35 |

| | |
|---|---|
| 6,07 | 3,99 |
| 3,31 | 2,01 |
| 1,45 | 0,96 |
| 1,00 | 0,60 |
| 0,42 | 4,43 |
| 7,61 | 0,32 |
| 6,21 | 1,35 |
| NAN | NAN |
| 3,53 | 2,09 |
| 4,48 | 6,19 |
| 2,81 | 1,21 |
| 8,99 | 5,67 |
| NAN | 1,24 |
| 2,41 | 2,14 |
| 2,38 | 1,37 |
| NAN | NAN |
| 1,04 | 1,40 |
| 4,22 | 3,71 |
| 0,75 | 0,53 |
| 2,66 | 1,12 |
| 1,02 | 0,86 |
| 5,56 | 4,24 |
| 0,88 | 0,39 |
| 11,54 | 4,10 |
| 0,42 | 0,24 |
| 1,35 | 1,19 |
| 1,64 | 0,70 |
| 1,52 | 0,23 |
| 6,32 | 5,10 |
| 2,52 | 0,52 |
| 10,71 | 3,77 |
| 4,46 | 1,30 |
| 1,96 | 0,89 |
| 2,31 | 1,34 |
| 3,78 | 1,28 |
| 5,25 | 1,54 |
| 6,89 | 3,46 |
| 0,80 | 0,49 |
| 1,57 | 1,28 |
| 5,16 | 2,17 |
| 6,06 | 2,72 |
| 2,65 | 1,59 |
| 0,78 | 0,15 |
| 15,85 | 5,10 |
| 11,81 | 4,08 |
| NAN | NAN |
| 2,30 | 3,20 |
| 4,77 | 1,61 |
| 1,39 | 0,74 |
| 1,90 | 0,67 |

| | |
|---|---|
| 5,85 | 12,39 |
| 7,16 | 3,58 |
| 14,43 | 0,09 |
| 6,10 | 1,08 |
| 2,04 | 0,54 |
| 1,82 | 0,56 |
| 3,34 | 2,32 |
| 2,44 | 1,44 |
| 2,26 | 1,34 |
| NAN | NAN |
| 3,97 | 2,75 |
| 2,97 | 0,81 |
| 14,30 | 5,90 |
| 10,24 | 6,90 |
| 0,92 | 0,74 |
| 7,22 | 2,83 |
| NAN | NAN |
| 5,32 | 0,94 |
| 1,05 | 0,50 |
| 36,98 | 22,05 |
| 2,83 | 1,02 |
| 1,44 | 1,21 |
| 4,65 | 1,54 |
| 3,05 | 0,53 |
| 1,03 | 0,73 |
| 3,17 | 1,64 |
| 11,32 | 4,11 |
| 1,40 | 0,91 |
| 1,08 | 0,48 |
| 8,19 | 5,49 |
| 0,71 | 0,49 |
| 5,88 | 0,35 |
| 2,32 | 1,68 |
| 2,91 | 2,25 |
| 0,84 | 0,31 |
| 3,02 | 1,87 |
| 0,49 | 0,38 |
| 0,81 | 0,59 |
| 1,21 | 1,03 |
| 1,40 | 0,94 |
| 2,04 | 0,97 |
| 0,68 | 0,48 |
| 0,73 | 0,84 |
| 2,40 | 1,08 |
| 2,26 | NAN |
| 2,93 | 1,07 |
| 4,46 | 1,63 |
| 6,98 | 1,40 |
| 2,52 | 1,15 |
| 1,42 | 0,92 |

| | |
|---|---|
| 2,20 | 0,66 |
| 1,40 | 0,53 |
| 4,34 | 1,29 |
| 10,82 | 1,85 |
| 3,55 | 0,51 |
| 3,18 | 1,21 |
| 10,75 | 4,22 |
| 8,31 | 5,72 |
| 2,67 | 1,00 |
| 20,64 | 4,18 |
| 6,75 | 5,08 |
| 10,09 | 2,53 |
| 23,49 | 6,88 |
| 2,82 | 0,71 |
| 13,18 | 2,64 |
| 3,29 | 0,91 |
| 5,25 | 1,31 |
| 2,15 | 1,84 |
| 0,81 | 0,51 |
| 4,93 | 3,79 |
| 3,44 | 3,68 |
| 5,99 | 3,67 |
| 1,92 | 0,00 |
| 2,80 | 5,27 |
| 1,08 | 0,61 |
| 1,87 | 3,89 |
| 12,17 | 7,38 |
| 16,80 | 9,66 |
| 1,01 | 0,85 |
| 2,21 | 1,46 |
| 7,40 | 1,36 |
| 3,24 | 2,51 |
| 2,73 | 3,79 |
| 2,24 | 1,05 |
| 0,99 | 1,27 |
| 2,84 | 1,53 |
| 2,96 | 1,21 |
| 2,00 | 1,05 |
| 0,51 | 0,38 |
| 1,02 | 1,30 |
| 4,68 | 5,91 |
| 0,40 | 0,84 |
| NAN | NAN |
| NAN | NAN |
| NAN | NAN |
| NAN | NAN |
| NAN | NAN |
| NAN | NAN |
| NAN | NAN |
| NAN | NAN |

| | |
|---|---|
| NAN | NAN |
| NAN | NAN |
| NAN | NAN |
| NAN | NAN |
| NAN | NAN |
| 2,44 | 1,29 |
| NAN | NAN |
| 1,47 | 2,22 |
| 0,89 | 0,77 |
| NAN | NAN |
| 4,59 | 6,87 |
| NAN | NAN |
| NAN | NAN |
| 0,85 | 0,69 |
| NAN | NAN |
| 3,91 | 1,20 |
| 2,39 | 4,13 |
| NAN | NAN |
| NAN | NAN |
| NAN | NAN |
| NAN | NAN |
| 2,97 | 1,60 |
| 1,70 | 1,10 |
| 29,18 | 5,85 |
| 1,13 | 1,11 |
| 1,13 | 0,95 |
| 1,77 | 0,77 |
| 0,34 | 0,56 |
| 1,52 | 1,23 |
| 3,89 | 1,50 |
| 9,48 | 3,24 |
| 3,37 | 1,74 |
| 2,06 | 1,43 |
| 23,65 | 16,18 |
| 4,44 | 4,08 |
| 1,51 | 1,04 |
| 12,80 | 6,05 |
| NAN | NAN |
| NAN | NAN |
| NAN | NAN |
| NAN | NAN |
| NAN | NAN |
| NAN | NAN |
| NAN | NAN |
| NAN | NAN |
| NAN | NAN |
| NAN | NAN |
| NAN | NAN |
| NAN | NAN |
| NAN | NAN |

| | |
|---|---|
| NAN | NAN |
| NAN | NAN |
| NAN | NAN |
| NAN | NAN |
| NAN | NAN |
| NAN | NAN |
| NAN | NAN |
| NAN | NAN |
| NAN | NAN |
| NAN | NAN |
| NAN | NAN |
| NAN | NAN |
| NAN | NAN |
| NAN | NAN |
| NAN | NAN |
| NAN | NAN |
| NAN | NAN |
| NAN | NAN |
| NAN | NAN |
| NAN | NAN |
| NAN | NAN |
| NAN | NAN |
| NAN | NAN |
| NAN | NAN |
| NAN | NAN |
| NAN | NAN |
| NAN | NAN |
| NAN | NAN |
| NAN | NAN |
| NAN | NAN |
| NAN | NAN |
| NAN | NAN |
| NAN | NAN |
| NAN | NAN |
| NAN | NAN |
| NAN | NAN |
| NAN | NAN |
| NAN | NAN |
| NAN | NAN |
| NAN | NAN |
| NAN | NAN |
| NAN | NAN |
| NAN | NAN |
| NAN | NAN |
| NAN | NAN |
| NAN | NAN |
| NAN | NAN |
| NAN | NAN |
| NAN | NAN |
| NAN | NAN |

| | |
|---|---|
| NAN | NAN |
| NAN | NAN |
| NAN | NAN |
| NAN | NAN |
| NAN | NAN |
| NAN | NAN |
| NAN | NAN |
| NAN | NAN |
| NAN | NAN |
| NAN | NAN |
| NAN | NAN |
| NAN | NAN |
| NAN | NAN |
| NAN | NAN |
| NAN | NAN |
| NAN | NAN |

| Adiponectin_leptin ratio 2PD |
| --- |
| 6,93 |
| 0,82 |
| 1,64 |
| 1,06 |
| 0,93 |
| 2,77 |
| 4,65 |
| 0,21 |
| 1,27 |
| 0,34 |
| 7,42 |
| 0,91 |
| 3,11 |
| 0,65 |
| 10,31 |
| 5,43 |
| 8,98 |
| 2,62 |
| 2,15 |
| 0,35 |
| 5,70 |
| 1,01 |
| 0,81 |
| 6,44 |
| 0,94 |
| 2,35 |
| 1,87 |
| 0,87 |
| 24,22 |
| 2,00 |
| 0,41 |
| 1,35 |
| 1,28 |
| 2,16 |
| 1,21 |
| 3,63 |
| 18,41 |
| 0,40 |
| 0,26 |
| 0,42 |
| 1,04 |
| 1,44 |
| 0,72 |
| 1,89 |
| 4,44 |
| 1,73 |
| 4,78 |

| |
|---|
| 7,49 |
| 4,61 |
| 2,57 |
| 0,64 |
| 5,88 |
| 0,40 |
| 4,30 |
| NAN |
| 2,44 |
| 3,43 |
| 17,00 |
| 2,07 |
| 1,93 |
| 20,12 |
| 2,97 |
| NAN |
| 5,12 |
| 7,20 |
| 0,33 |
| 1,70 |
| 0,38 |
| 2,81 |
| 0,42 |
| 4,48 |
| 0,27 |
| 1,09 |
| 8,12 |
| 0,23 |
| 5,29 |
| 1,31 |
| 9,93 |
| 1,75 |
| 1,18 |
| 2,04 |
| 2,19 |
| 1,53 |
| 3,37 |
| 0,92 |
| 2,15 |
| 3,95 |
| 6,23 |
| 2,40 |
| 0,56 |
| 10,57 |
| 7,43 |
| NAN |
| 16,35 |
| 8,00 |
| 1,57 |
| 5,20 |

| |
|---|
| 8,76 |
| 15,80 |
| 0,38 |
| 3,03 |
| 1,96 |
| 3,19 |
| 5,76 |
| 3,09 |
| 2,75 |
| NAN |
| 2,93 |
| 0,70 |
| 11,71 |
| 26,89 |
| 1,29 |
| 8,77 |
| NAN |
| 15,88 |
| 3,35 |
| 30,72 |
| 1,72 |
| 2,44 |
| 6,30 |
| 6,42 |
| 1,29 |
| 4,08 |
| 21,44 |
| 2,93 |
| 0,35 |
| 15,36 |
| 0,85 |
| 0,45 |
| 2,52 |
| 1,92 |
| 0,50 |
| 3,84 |
| 0,39 |
| 0,56 |
| 1,59 |
| 1,32 |
| 1,50 |
| 0,52 |
| 0,42 |
| 1,49 |
| NAN |
| 1,42 |
| 2,51 |
| 3,61 |
| 1,21 |
| 1,12 |

| |
|---|
| 1,42 |
| 0,56 |
| 5,53 |
| 4,34 |
| 0,98 |
| 9,00 |
| 12,85 |
| 4,33 |
| 1,71 |
| 7,20 |
| 2,72 |
| 3,79 |
| 5,77 |
| 0,88 |
| 2,65 |
| 1,80 |
| 2,34 |
| 1,51 |
| 0,81 |
| 2,48 |
| 7,44 |
| 19,92 |
| 1,25 |
| 4,83 |
| 1,56 |
| 6,46 |
| 22,64 |
| 5,54 |
| 0,77 |
| 3,10 |
| 5,27 |
| 6,19 |
| 4,84 |
| 2,11 |
| 1,70 |
| 3,29 |
| 1,95 |
| 1,03 |
| 1,68 |
| 1,21 |
| 6,79 |
| 0,55 |
| NAN |
| NAN |
| NAN |
| NAN |
| NAN |
| NAN |
| NAN |
| NAN |

NAN
NAN
NAN
NAN
NAN
2,25
NAN
0,86
2,09
NAN
4,67
NAN
NAN
0,70
NAN
2,12
1,40
NAN
NAN
NAN
NAN
3,65
1,46
13,44
0,48
0,58
1,35
0,26
0,87
3,61
4,60
3,37
2,46
16,38
3,80
0,93
10,45
NAN
NAN
NAN
NAN
NAN
NAN
NAN
NAN
NAN
NAN
NAN
NAN
NAN

| |
|---|
| NAN |
| NAN |
| NAN |
| NAN |
| NAN |
| NAN |
| NAN |
| NAN |
| NAN |
| NAN |
| NAN |
| NAN |
| NAN |
| NAN |
| NAN |
| NAN |
| NAN |
| NAN |
| NAN |
| NAN |
| NAN |
| NAN |
| NAN |
| NAN |
| NAN |
| NAN |
| NAN |
| NAN |
| NAN |
| NAN |
| NAN |
| NAN |
| NAN |
| NAN |
| NAN |
| NAN |
| NAN |
| NAN |
| NAN |
| NAN |
| NAN |
| NAN |
| NAN |
| NAN |
| NAN |
| NAN |
| NAN |
| NAN |
| NAN |
| NAN |

NAN
NAN
NAN
NAN
NAN
NAN
NAN
NAN
NAN
NAN
NAN
NAN
NAN
NAN
NAN
NAN
